# TRIP13 promotes the expansion and immunosuppression of CD4^+^Foxp3^+^ regulatory T cells by sustaining HAT1 stability

**DOI:** 10.1038/s41419-025-08214-7

**Published:** 2026-01-14

**Authors:** Tianzhen He, Liwen Zhao, Chu-Ting Feng, Li-Ya Zhao, Shengnan Jing, Han Yang, Ke Wang, Siyu Ye, Yingchun Zhao, Ying Yu, Zhuting Fu, Chon-Kit Chou, Xin Chen, Yong-Jing Gao

**Affiliations:** 1 Institute of Pain Medicine and Special Environmental Medicine, Co-innovation Center of Neuroregeneration, Nantong University, Nantong, Jiangsu China; 2 Department of Urology, The Affiliated Hospital of Qingdao University, Qingdao University, Qingdao, Shangdong China; 3 Affiliated Hospital of Nantong University, Medical School of Nantong University, Nantong, China; 4 Analysis and Testing Center, Nantong University, Nantong, Jiangsu China; 5 State Key Laboratory of Quality Research in Chinese Medicine, Institute of Chinese Medical Sciences, University of Macau, Avenida da Universidade Taipa, Macau, SAR China

**Keywords:** Crohn's disease, Peripheral tolerance

## Abstract

There is compelling evidence that TNF preferentially activates and expands CD4^+^Foxp3^+^ regulatory T cells (Tregs) through TNFR2. However, the precise mechanisms underlying TNF-TNFR2 pathway-mediated Treg proliferation remain to be fully elucidated. In this study, using RNA-seq profiling of TNFR2^+^ and TNFR2-deficient Treg cells, we identified that *Trip13* is required for promoting TNF-TNFR2 pathway-mediated Treg expansion. Mechanistically, TRIP13 inhibited UBE4A-mediated ubiquitination degradation of HAT1 by directly binding to HAT1, thereby competing with UBE4A and promoting Treg expansion. In addition, TRIP13’s ATPase activity was essential for its binding to HAT1, which promoted Treg expansion by increasing Foxp3 expression. In a mouse colitis model, TRIP13 overexpression markedly alleviated colon inflammation by enhancing Treg expansion, an effect that was reversed by HAT1 knockdown. Conversely, genetic ablation of TRIP13 substantially reversed the effects induced by HAT1 overexpression, including enhanced Treg expansion and attenuation of colitis. These findings illustrate the TRIP13/HAT1 axis-mediated mechanism for TNF-TNFR2-induced Treg expansion and indicate that targeting TRIP13 may offer therapeutic potential for autoimmune and inflammatory diseases.

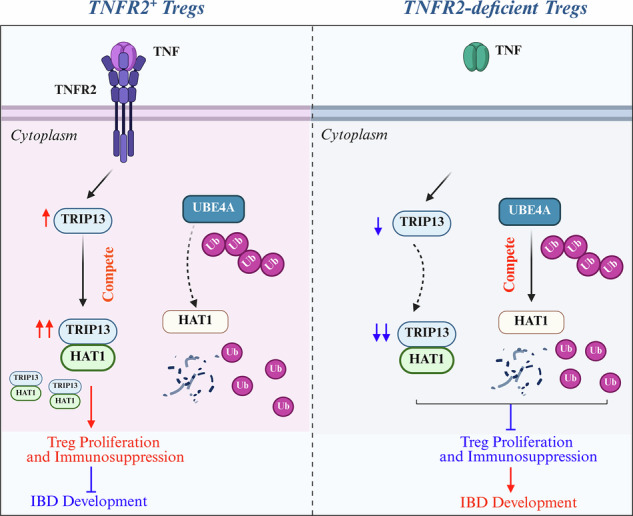

## Introduction

CD4^+^Foxp3^+^ regulatory T cells (Tregs) are crucial for maintaining immune homeostasis and preventing autoimmune responses [1]. They also play a major role in the immune evasion of cancer by suppressing immune responses against tumors [2]. Targeting Tregs has emerged as a strategy in treating major human diseases, including cancer, allergic and autoimmune diseases, transplantation rejection, and graft-versus-host disease (GvHD) [3, 4]. A thorough understanding of the biological pathways regulating Treg function is essential for developing therapeutic interventions.

Chen et al. were the first to report that tumor necrosis factor (TNF) activates Tregs through TNF receptor type II (TNFR2), one of TNF receptors preferentially expressed by Tregs [5]. Furthermore, the expression of TNFR2 identifies the most potent suppressive subset of human and mouse Tregs [6, 7]. In contrast, Tregs lacking TNFR2 exhibited minimal or no suppressive activity [6, 8, 9]. Moreover, TNF-TNFR2 signaling is important for maintaining the phenotypic stability of Tregs, including Foxp3 expression [5, 9–11], and plays a decisive role in the activation and expansion of Tregs [12–15]. However, the underlying mechanisms of Treg expansion by TNF-TNFR2 signaling remain incompletely understood.

Thyroid receptor-interacting protein 13 (TRIP13) is a member of the AAA+ ATPase family and is known for its ability to generate mechanical forces via ATP hydrolysis processes [16]. Multiple lines of evidence indicate that TRIP13 interacts with a diverse range of proteins and is involved in a variety of malignancies, including colorectal cancer [17], liver cancer [18], prostate cancer [19], and bladder cancer [20]. TRIP13 inhibitors have shown the potential to enhance cytotoxicity and promote tumor regression when combined with immune checkpoint inhibitors that target PD-1 and CTLA-4 [21, 22]. Inhibition of TRIP13 by small molecule improved immune responses in an immunocompetent syngeneic pancreatic ductal adenocarcinoma model by decreasing the expression of PD-1/PD-L1, increasing the expression of granzyme B/perforin, and promoting CD3^+^/CD4^+^ T cell infiltration [23]. However, the role of TRIP13 in immune cells and its impact on colitis development remain largely unknown.

Histone acetyltransferase 1 (HAT1) is a member of the Gcn5-related N-acetyltransferase family. HAT1’s primary role is to catalyze the transfer of an acetyl group from acetyl coenzyme A specifically to the ε-amino group of lysine (K) 5 and K12 in the amino-terminus of an H4 histone. This modification occurs immediately after the production of the H4 in the cytosol and before chromatin formation [24]. HAT1 has been linked to Foxp3 in stimulating the infiltration of CCR4^+^ Tregs into the tumor microenvironment [25]. In addition, HAT1 recruits the ubiquitin E3/E4 ligase UBE4A, triggering UBE4A-mediated polyubiquitination degradation of the viperin protein, thereby inhibiting host antiviral immunity [26]. However, the biological importance of HAT1 in Treg proliferation and its interaction with TRIP13 has not yet been explored.

In this study, we analyzed the RNA-seq profiles of TNFR2^+^ Treg cells and TNFR2-deficient Treg cells, identifying *Trip13* as the downstream gene in the TNF-TNFR2 signaling pathway in Tregs. Overexpression of TRIP13 promoted Treg expansion, thereby inhibiting the progression of colitis in a mouse T cell transfer model. TRIP13 knockout markedly reversed the effects of HAT1 overexpression on Treg expansion and the progression of colitis. Mechanistically, TRIP13 attenuated UBE4A-mediated ubiquitination degradation of HAT1 by directly binding to HAT1, competitively inhibiting its interaction with UBE4A. Our results indicate that targeting TRIP13 may represent a promising therapeutic strategy for the treatment of T cell-mediated inflammation.

## Results

### TRIP13 is required for TNF-mediated Treg expansion through TNFR2 in vitro

**Fig. 1:**
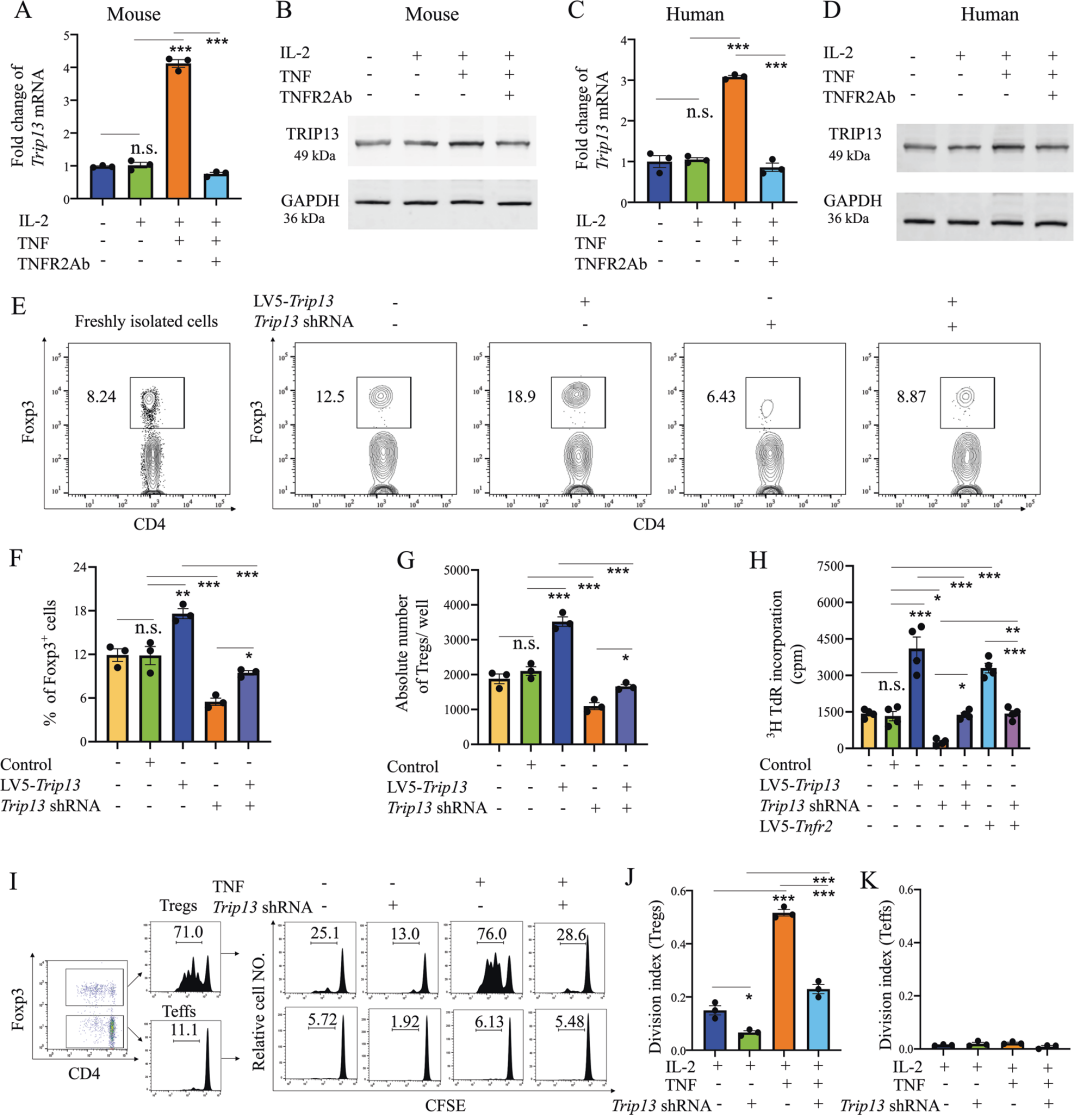
TRIP13 is required for TNF-mediated Treg expansion through TNFR2 in vitro. **A–D** TNF stimulation increased TRIP13 expression in mouse and human Treg cells, as measured by qRT-PCR and WB. CD4^+^CD25^+^ T cells were sorted by MACS and stimulated with or without IL-2 (50 pg/mL), recombinant TNF (10 ng/mL), or anti-TNFR2 antibody (160 μg/ml) for 72 h. **E–K** MACS-sorted CD4^+^ T cells were transfected with the LV5-*Trip13*, *Trip13* shRNA, LV5-*Tnfr2*, or TNF (10 ng/mL) in the presence of IL-2 (50 pg/mL). In some experiments, CD4^+^ T cells were labeled with CFSE. After 72 h, Treg proliferation was assessed by FCM or using a [^3^H] thymidine incorporation assay. Typical FCM histogram (**E**), percentage of Treg (**F**), absolute number of Tregs (**G**), representative cpm data of the [^3^H] thymidine incorporation assay (**H**), typical FCM histogram showing the proportion of replicating cells (**I**), division index of Tregs (**J**), and Division index of Teffs (**K**). “Control +” represents the group treated with vehicle control (e.g., LV5-NC). In contrast, “control –” indicates a baseline group without any viral transduction. Data were displayed as means ± SEM from three independent experiments. *P < 0.05, **P < 0.01, ***P < 0.001. n.s. no significant differences.

To identify the target(s) required for TNF-TNFR2-mediated Treg proliferation, we compared differentially expressed genes between TNFR2^+^ Treg cells and TNFR2-deficient Treg cells based on RNA-seq analysis (Fig. S1A). Among the genes upregulated in TNFR2^+^ Treg cells, we focused on those that had not been previously reported to play a role in Treg proliferation or colitis progression. Notably, TRIP13 expression was found to be approximate 5-fold higher in TNFR2^+^ Treg cells compared to TNFR2-deficient Treg cells (Fig. S1A). To further investigate whether TRIP13 is regulated by the TNF-TNFR2 signaling pathway in Tregs, CD4^+^ T cells were purified from the spleen and lymph nodes (LNs) of normal mice using magnetic antibody cell sorting (MACS). These cells were cultured with IL-2 to maintain viability [27]. The result showed that TRIP13 expression in mouse and human Tregs was upregulated following TNF stimulation, and this effect was completely inhibited by anti-TNFR2 antibody treatment (Fig. 1A–D). These data suggest that TRIP13 expression is regulated by the TNF-TNFR2 signaling pathway in Tregs.

To further determine whether TRIP13 overexpression could induce the proliferative expansion of Tregs, CD4^+^ T cells were purified from spleen and LNs of normal mice using MACS and transfected with either lentivirus-mediated overexpression of *Trip13* (LV5-*Trip13*) or *Trip13* shRNA (The efficiency of LV5-*Trip13*, *Trip13* shRNA1, and *Trip13* shRNA2 in the transfected CD4^+^ T cells is shown in Fig. S1B, C). LV5-*Trip13* stimulated Treg proliferation, resulting in a 49.2% increase in Treg numbers, approximately 1.5-fold greater than in the control groups (p < 0.01). In contrast, *Trip13* shRNA1 reduced the number of Tregs by approximately 1.5-fold compared to the control lentivirus (P < 0.01. Fig. 1E–G). Furthermore, *Trip13* shRNA1 completely blocked the proliferation of Tregs induced by LV5-*Tnfr2* (overexpression efficiency in transfected CD4^+^ T cells is shown in Fig. S1D) or TNF (p < 0.001, Fig. 1H–J). However, neither TRIP13 nor TNF stimulated the proliferation of mouse effector CD4^+^ T cells (Teffs, Fig. 1K). These results indicate that TRIP13 is crucial in TNF-TNFR2 signaling-mediated Treg proliferation in vitro.

### The TNFR2-TRIP13 axis promotes Treg expansion in vivo

**Fig. 2:**
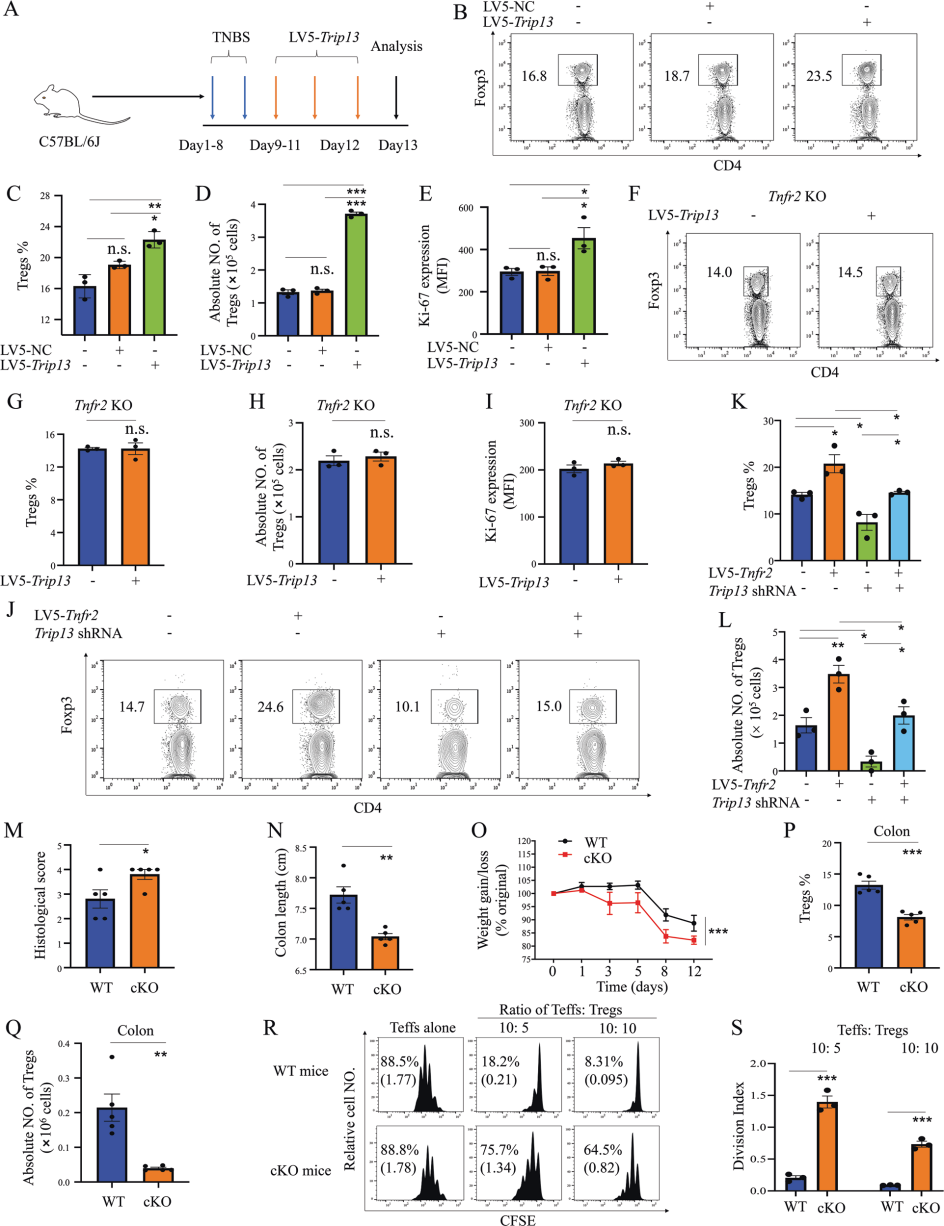
TRIP13 is crucial for TNFR2-mediated Treg proliferation in vivo in the inflammatory context. **A** Schematic diagram of experimental procedure. FCM analysis shows the proportion (**B**, **C**, **F**, **G**), number of colonic Tregs (**D** and **H**), and expression of Ki-67 (**E** and **I**) after LV5-NC or LV5-*Trip13* treatment in WT and TNFR2 KO mice. The proportion (**J** and **K**) and number of colonic Tregs (**L**) after i.p. injection with *Trip13* shRNA or LV5-*Tnfr2* or control for 3 days were shown. The development of colitis (**M**–**O**), proportion (**P**), and number of colonic Tregs (**Q**) after colitis induction by TNBS in *Foxp3*^YFP-cre^ and *Trip13* ^fl/fl^*Foxp3*^YFP-cre^ mice were shown. Typical FCM plots display the proportion of gated cells. **R**, **S** The immunosuppression of Tregs in *Trip13* ^fl/fl^*Foxp3*^YFP-cre^ mice was determined. Representative FCM histogram (**R**, the numbers indicate the proportion of replicating cells (%) and the division index), and the summary of the division index (**S**), were shown. Data are presented as means ± SEM from 3 mice per group and are representative of three independent experiments. *P < 0.05, **P < 0.01, ***P < 0.001. n.s. no significant differences.

We then investigated whether lentivirus-mediated overexpression of TRIP13 could stimulate the in vivo expansion of Tregs in the inflammatory context. C57BL/6J mice were intracolonally administered with TNBS on day 1 and day 8, and then injected intraperitoneally (i.p.) with LV5-*Trip13* for 3 days, resulting in a more than 2-fold increase in *Trip13* mRNA expression (Fig. S1E). On day 12, the mice were sacrificed, and the number, phenotype, and function of Tregs in the colon were analyzed. As shown in Fig. 2A–C, the proportion of Foxp3^+^ cells within colonic CD4^+^ T cells increased by 17.4% in LV5-*Trip13*-treated mice compared to the control lentivirus group (P < 0.05). The absolute number of Tregs in the colon also doubled following LV5-*Trip13* treatment (P < 0.001, Fig. 2D). Additionally, the expression of Ki-67, a proliferating marker co-expressed with TNFR2 in highly suppressive Tregs [8], was significantly increased in Tregs following LV5-*Trip13* treatment (P < 0.05, Fig. 2E). These results suggest that the increase in Tregs in LV5-*Trip13*-treated mice is due to the expansion of pre-existing naturally occurring Tregs (nTregs) rather than the conversion of naïve CD4^+^ T cells into induced Tregs (iTregs). Additionally, neither the proportion nor the absolute number of colonic Tregs, as well as Ki-67 expression in these Tregs, was altered in *Tnfr2* KO mice (Fig. 2F–I).

To further investigate the impact of TRIP13 knockdown on Treg proliferation, we injected mice with lentivirus expressing *Trip13* shRNAs (*Trip13* shRNA1 and *Trip13* shRNA2). Similarly, WT mice were infected with *Trip13* shRNAs or with a control lentivirus in the inflammatory context. Three days post-injection, the treatment effectively knocked down *Trip13* mRNA expression (Fig. S2A). Treatment with *Trip1*3 shRNA1 or shRNA2 resulted in 18% or 24.5% decrease, respectively, in the proportion of Tregs within CD4^+^ T cells, and a 36.8% or 34.6% decrease, respectively, in the number of Tregs in the colons of mice compared with control lentivirus-treated mice (p < 0.001, Fig. S2B–D). Additionally, the capacity of LV5-*Trip13* in promoting Treg expansion was abolished in mice infected with *Trip13* shRNA1 (Fig. S2F–H). Ki-67 expression in Tregs significantly decreased following treatment with *Trip13* shRNA1 or shRNA2 (P < 0.01, Fig. S2E and S2I). Furthermore, LV5-*Tnfr2* treatment resulted in a significant increase in both the proportion and number of Tregs (P < 0.01), but this effect was abolished by *Trip13* shRNA1 (P < 0.05, Fig. 2J–L). These data indicate that the TNF-TNFR2-TRIP13 signaling pathway plays a crucial role in the proliferation of Tregs in vivo in the inflammatory context.

### TRIP13 deficiency in Treg cells led to exacerbated colitis

We also investigated the role of TRIP13 in Treg cell biology using conditionally knockout mice (cKO mice) that conditionally deleted *Trip13* in Treg cells (*Trip13* ^fl/fl^*Foxp3*^YFP-cre^) (Fig. S3A). RT-qPCR analysis showed that the expression of *Trip13* was diminished at mRNA level in peripheral Treg cells, rather than Teffs, from *Trip13* ^fl/fl^*Foxp3*^YFP-cre^ mice (Fig. S3B, C). *Trip13* ^fl/fl^*Foxp3*^YFP-cre^ mice did not show any distinct abnormalities in thymic or peripheral Treg cell homeostasis. The percentage and number of Treg cells in lymphoid tissues remained unchanged (Fig. S3D–F). Consequently, TRIP13 is not required for the maintenance of Treg cell homeostasis in the steady state.

Compared with *Foxp3*^YFP-cre^ mice (Cre mice), *Trip13* ^fl/fl^*Foxp3*^YFP-cre^ mice (conditional *Trip13* knockout in Treg cells) promoted the progression of colitis (Fig. 2M–O) by day 12. This was not associated with increased total leukocyte infiltration in the colon (Fig. S3G). The proportion and number of Treg cells were reduced in the colon of cKO mice (Fig. 2P, Q). In addition, we generated *Trip13*^fl/fl^*Itgax*^cre^ mice (conditional *Trip13* knockout in dendritic cells, DCs) to investigate whether TRIP13 deficiency in DCs affects Treg proliferation and the progression of colitis. Our results showed that TRIP13 deletion in DCs had no remarkable effect on Treg proliferation or colitis development (Fig. S4A–K). Furthermore, we assessed whether TRIP13 influences Treg differentiation and found that TRIP13 deficiency did not alter the differentiation of Tregs (Fig. S4L–N).

We also examined the immunosuppressive function of Tregs isolated from *Foxp3*^YFP-cre^ and *Trip13* ^fl/fl^*Foxp3*^YFP-cre^ mice. As shown in Fig. 2R, S, Tregs from *Trip13* ^fl/fl^*Foxp3*^YFP-cre^ mice exhibited significantly reduced suppressive function, compared to those from control mice (P < 0.001). Hence, this suggests that TRIP13 is involved in the acquisition of an immunosuppressive state by Treg cells on day 12 after disease induction, and promoting Treg immunosuppressive activity.

### The TRIP13-HAT1 axis promotes Treg proliferation in vivo

**Fig. 3:**
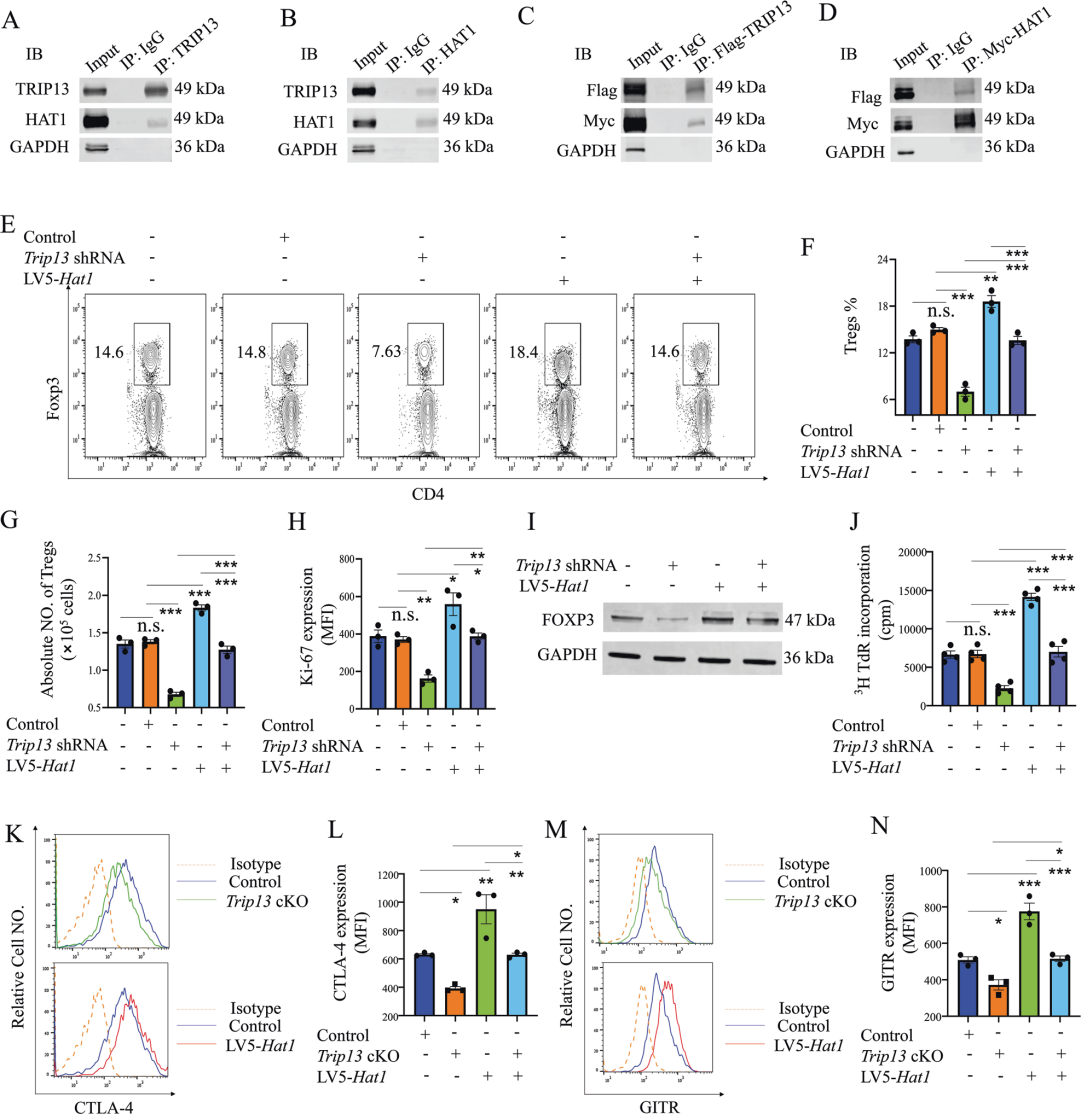
Knockdown of TRIP13 inhibits TNF/TNFR2-mediated Treg proliferation by down-regulating HAT1 expression. Co-IP assays demonstrated the specific interaction between TRIP13 and HAT1 in MACS-sorted Treg cells (**A**, **B**) and HEK-293T cells transfected with Flag-TRIP13 and Myc-HAT1 plasmids (**C**, **D**). Proportion (**E** and **F**), number of colonic Tregs (**G**) and expression of Ki-67 (**H**) by colonic Tregs after i.p. injection with *Trip13* shRNA or/and LV5-*Hat1* (i.p.) for 3 days were analyzed by FCM. **I**, **J** MACS-sorted human Treg cells were used to transfect with the *Trip13* shRNA or LV5-*Hat1* in the presence of recombinant human TNF, and TNFR2 agonistic antibody for 7 days. Then the expression of Foxp3 was determined by WB (**I**), and the proliferation was measured by [^3^H] TdR incorporation (**J**). Expression of CTLA-4 (**K**, **L**), and GITR (**M**, **N**) by Tregs after i.p. injection with LV5-*Hat1* in control or TRIP13 cKO mice for 3 days were analyzed by FCM. “Control +” represents the group treated with vehicle control (e.g., LV5-NC). In contrast, “control -” indicates a baseline group without any viral transduction. Data (means ± SEM, A-H: n = 3 mice, I-J: n = 3 or 4 healthy donors) were representative of three separate experiments. Compared with the indicated group, *P < 0.05, ** P < 0.01, ***P < 0.001, n.s. no significant differences.

To further elucidate the underlying mechanism of TRIP13-mediated Treg proliferation, we examined the binding partners of TRIP13 in Tregs using co-immunoprecipitation (co-IP) coupled with mass spectroscopy (Supplemental Dataset 1). Among the putative binding proteins, we identified and validated an interaction with HAT1 (Fig. 3A), a well-recognized marker associated with Treg infiltration [25]. Immunoprecipitating using anti-IgG, anti-HAT1, anti-TRIP13, anti-Flag, or anti-Myc antibodies confirmed the specific interaction between TRIP13 and HAT1 in Tregs and HEK-293T cells transfected with *Trip13* and *Hat1* plasmids (Fig. 3A–D).

We then investigated whether the TRIP13-HAT1 axis regulates Treg proliferation. WT mice were intracolonally administered with TNBS on day 1 and day 8, and then infected with *Trip13* shRNA1 or lentivirus encoding *Hat1* (LV5-*Hat1*). Three days after LV5*-Hat1* treatment, *Hat1* mRNA expression increased by more than 2-fold (Fig. S5A). In addition, LV5-*Hat1* treatment increased both the proportion (P < 0.01) and number of Tregs (P < 0.001, Fig. 3E–G), as well as Ki-67 expression in Tregs (P < 0.05, Fig. 3H). Furthermore, the ability of *Trip13* shRNA1 to downregulate mouse Treg expansion was attenuated by treatment with LV5-*Hat1* (P < 0.05, Fig. 3E–H). In line with the results seen in mouse Tregs, treatment with *Trip13* shRNA1 similarly decreased the Foxp3 expression and proliferation of MACS-sorted human Tregs (Fig. 3I, J). Additionally, LV5-*Hat1* down-regulated *Trip13* shRNA1’s ability to reduce the expression of FOXP3 and proliferation of human Tregs (Fig. 3I, J). In addition to assessing Foxp3 expression, we further examined the expression of key functional markers of Treg, including CTLA-4 and GITR. As a result, Tregs from *Trip13*^*fl/fl*^*Foxp3*^*YFP-cre*^ mice showed significantly reduced expression of both CTLA-4 and GITR compared to controls. In addition, overexpression of HAT1 via LV5-HAT1 lentivirus partially restored the expression of these markers (Fig. 3K–N), suggesting that the TRIP13-HAT1 axis modulates Treg expansion and function.

### TRIP13 interacts with HAT1 to inhibit HAT1 degradation

**Fig. 4:**
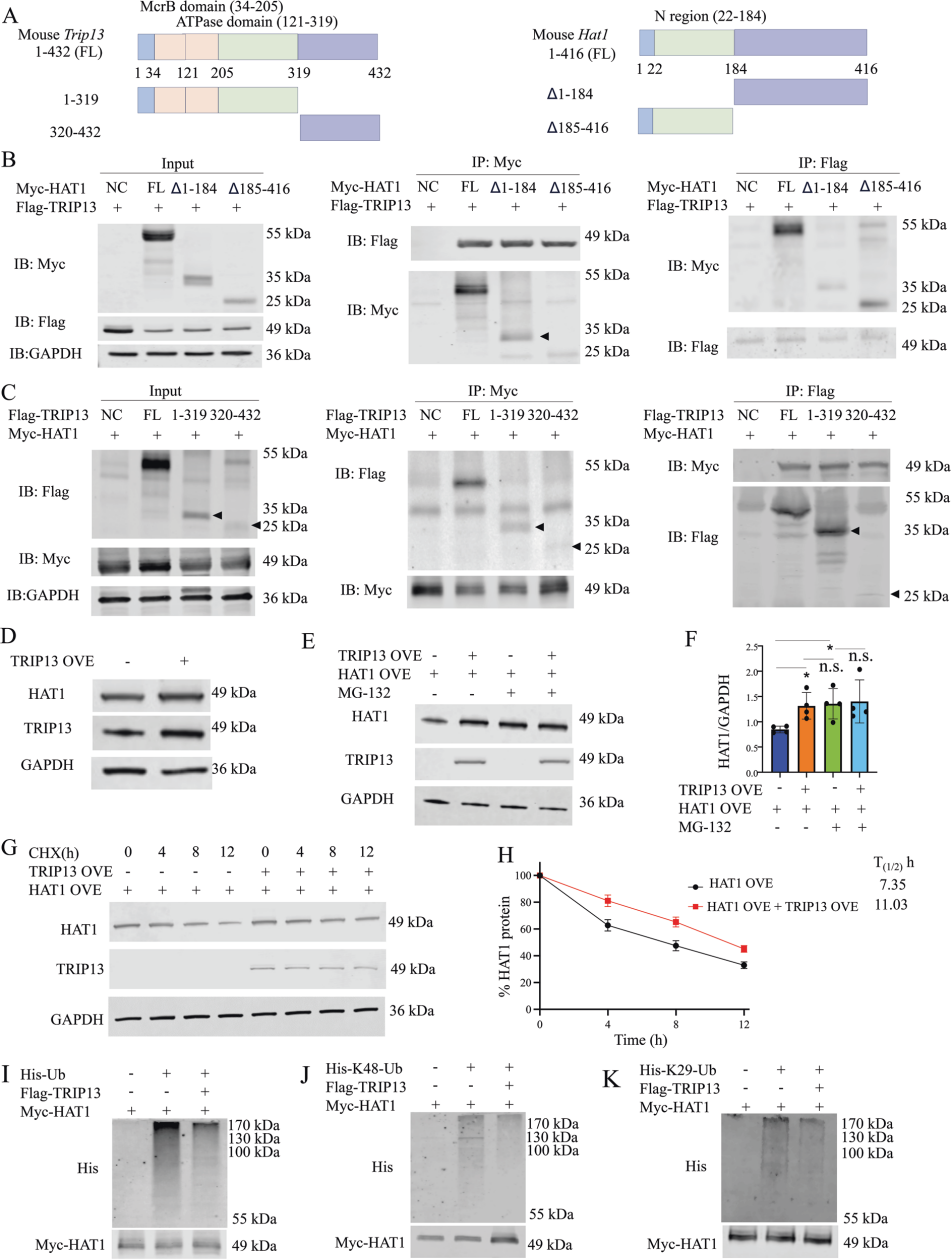
TRIP13 interacts with HAT1 protein and inhibits Ubiquitin-mediated HAT1 degradation. **A** Schematic diagram of the TRIP13 and HAT1 protein domains was shown. **B**, **C** co-IP assays showed the interaction between TRIP13 and different truncated mutants of HAT1 or between HAT1 and different truncated mutants of TRIP13. **D** MACS-sorted Tregs were transfected with the LV5-*Trip13*, then were subjected to determine the expression of HAT1 by WB. **E**, **F** HAT1 and TRIP13 expression in the HEK293T cells treated with or without MG-132 was analyzed by WB. **G**, **H** The effect of overexpressing TRIP13 on the half-life of HAT1 was evaluated in the HEK293T cells treated with CHX and collected at the indicated time points. **I–K** The effect of overexpressing TRIP13 on the levels of Ub or K48-, or K29-linked polyubiquitination of HAT1 was evaluated by immunoprecipitation of Myc-tagged HAT1 in HEK-293T cells. Data (means ± SEM, n = 3 independent experiments) were representative of three separate experiments. Compared with the indicated group, *P < 0.05, n.s. no significant differences.

We next identified the binding domains between TRIP13 and HAT1 using various truncated constructs of both proteins (Fig. 4A). Co-IP results showed that both the Δ1–184 amino acid (aa) (deletion of 1-184aa) and Δ185-416 aa segments (deletion of 185–416aa) of HAT1 interacted with TRIP13 (Fig. 4B). Similarly, all segments of TRIP13, including the 1–319 aa and 320-432 aa regions, interacted with HAT1 (Fig. 4C). These data reveal that all truncated segments of TRIP13 and HAT1 interact with each other.

Additionally, HAT1 protein levels, but not its mRNA levels, were markedly increased by LV5-*Tnfr2* and decreased by *Trip13* shRNA (Fig. S5B, C). Overexpression of TRIP13 markedly increased HAT1 protein levels, without affecting mRNA levels (Figs. 4D and S6A). Conversely, knocking down TRIP13 markedly reduced HAT1 protein levels but did not affect its mRNA levels (Fig. S6, B, C). To determine whether TRIP13 affects HAT1 degradation, we treated cells with the proteasome inhibitor MG-132. As shown in Fig. 4E, F, overexpressing TRIP13 had minimal effect on HAT1 protein levels when proteasome-mediated protein degradation was blocked by MG-132. Furthermore, overexpression of TRIP13 extended the protein half-life of HAT1 and significantly decreased its degradation (P < 0.05, Fig. 4G, H). These results indicate that TRIP13 overexpression inhibits the degradation of HAT1.

To determine whether TRIP13 interacts with HAT1 to decrease its ubiquitination in cells, we transiently transfected HEK-293T cells with Flag-tagged TRIP13, His-tagged ubiquitin (His-Ub), and Myc-tagged HAT1, or with empty vectors as controls. Co-IP of His-Ub revealed that overexpression of TRIP13 reduced the ubiquitination levels of HAT1 (Fig. 4I). Ubiquitin can link via one or more of its seven lysine residues (K6, K11, K27, K29, K33, K48, and K63), forming polyubiquitin chains that control protein fate, including degradation [28]. We further found that TRIP13’s interaction with HAT1 significantly suppressed K48-linked and K29-linked polyubiquitination (P < 0.05, Fig. 4J, K), but not K63-linked, K6-linked, K11-linked, K27-linked, or K33-linked ubiquitination (Fig. S7). These data suggest that TRIP13 binding to HAT1 abrogates K48/K29-linked polyubiquitination-mediated degradation of HAT1, thereby stabilizing HAT1 protein levels.

### UBE4A mediates HAT1 degradation and regulates Treg expansion

**Fig. 5:**
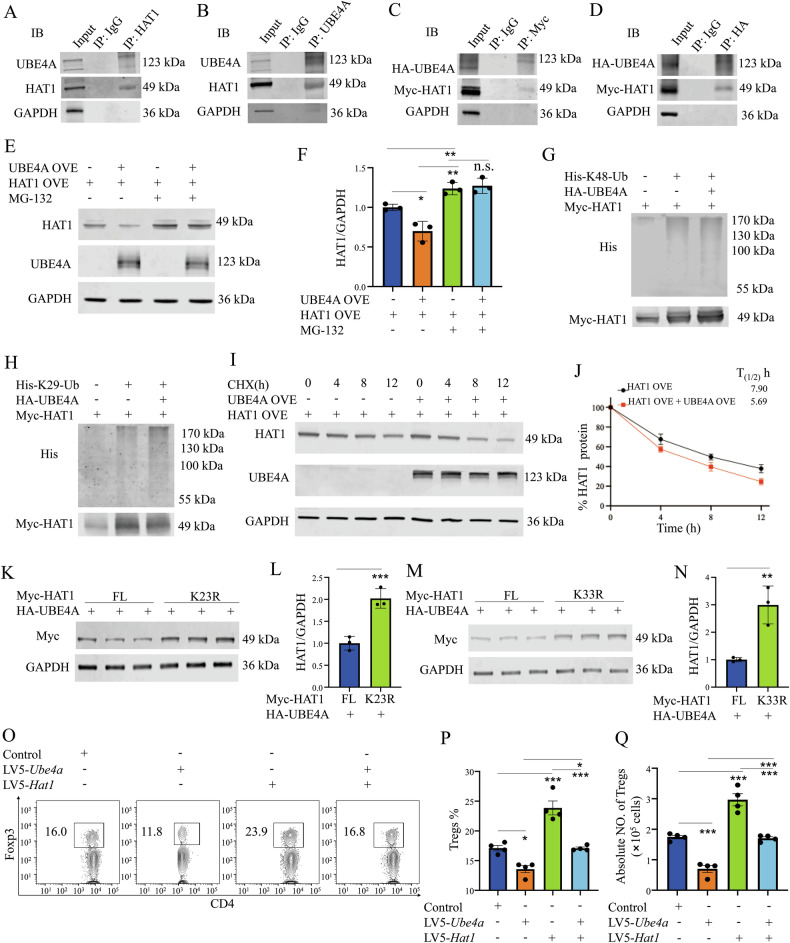
UBE4A induces the polyubiquitination-mediated degradation of HAT1 protein. Co-IP assays validated the specific interaction between UBE4A and HAT1 in MACS-sorted Treg cells (**A**, **B**) and HEK-293T cells transfected with HA-UBE4A and Myc-HAT1 plasmids (**C**, **D**). **E**, **F** WB analysis of HAT1 and UBE4A expression in the HEK293T cells treated with or without MG132. **G**, **H** The effect of overexpressing UBE4A on the levels of K48-, or K29-linked polyubiquitination of HAT1 was evaluated by immunoprecipitation of Myc-tagged HAT1. **I**, **J** The effect of overexpressing UBE4A on the half-life of HAT1 was evaluated in the HEK293T cells treated with CHX and collected at the indicated time points. **K–N** WB assays showed the specific ubiquitination sites of HAT1 for UBE4A-induced polyubiquitination degradation. Representative FCM plots (**O**), the proportion (**P**), and the number (**Q**) of Tregs after i.p. injection with LV5-*Ube4a*, or LV5-*Hat1*, or control lentivirus for 3 days were analyzed by FCM. Data (means ± SEM, n = 3 independent experiments) were representative of three separate experiments. Compared with the indicated group, **P < 0.01, ***P < 0.001.

Given that HAT1-mediated acetylation promotes the ubiquitin E3/E4 ligase activity of UBE4A [26], we investigated whether UBE4A contributes to HAT1 degradation and if TRIP13 can counteract this process. Co-IP confirmed the interaction between UBE4A and HAT1 (Fig. 5A–D). Overexpression of UBE4A hardly reduced HAT1 protein levels when proteasome-mediated protein degradation was blocked by MG-132 (Fig. 5E, F). Additionally, overexpression of UBE4A increased HAT1 polyubiquitination, specifically through K48- and K29-linked ubiquitin (Fig. 5G, H). Moreover, the HAT1 protein half-life was reduced to less than 6 h in UBE4A-overexpressing cells (Fig. 5I–J), indicating that UBE4A functions as an E3 ligase for HAT1.

We further investigated which specific ubiquitination site(s) of HAT1 are necessary for UBE4A-induced polyubiquitination and subsequent degradation. Using the Uniprot database (https://www.uniprot.org/), we identified six potential ubiquitination sites on HAT1 (K6, K12, K23, K33, K67, and K107). Mutating K23 and K33 to arginine (K23R and K33R) significantly stabilized HAT1 against UBE4A-mediated degradation (Fig. 5K–N), while mutations at other sites did not (Fig. S8). Further analysis revealed that UBE4A binds to HAT1, overlapping with the TRIP13-binding region (Fig. S9A). These results suggest that Lys-23 and Lys-33 are critical for UBE4A-directed HAT1 degradation.

We then evaluated the impact of UBE4A-mediated HAT1 protein degradation on Treg expansion by injecting mice with lentivirus expressing *Ube4a* (LV5-*Ube4a*) or *Hat1* after TNBS induction. Three days after LV5-*Ube4a* treatment, the mRNA expression of *Ube4a* was increased by more than 2-fold (Fig. S9B). Furthermore, LV5-*Ube4a* treatment completely blocked LV5-*Hat1*-induced Tregs expansion, even in the absence of LV5-*Hat1* (P < 0.05, Fig. 5O–Q), suggesting that UBE4A negatively regulates Treg homeostasis through HAT1 degradation.

### TRIP13 competitively disrupts UBE4A-mediated HAT1 degradation through ATPase-dependent binding

**Fig. 6:**
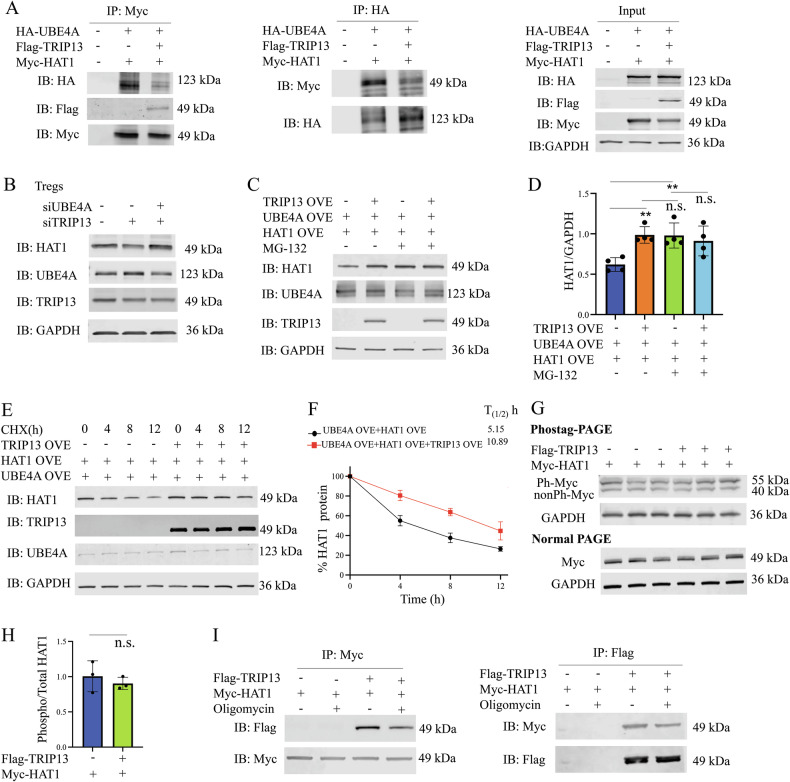
TRIP13 abrogates UBE4A-induced degradation of HAT1. **A** Co-IP assays showed TRIP13 abrogated the interaction between UBE4A and HAT1. **B** The effect of silencing TRIP13 or together with silencing UBE4A on the levels of HAT1 was assessed in Treg cells. **C**, **D** HAT1 expression in the HEK293T cells treated with or without MG-132 was analyzed by WB. **E**, **F** The effect of overexpressing TRIP13 on the half-life of HAT1 was evaluated in the HEK293T cells treated with CHX and collected at the indicated time points. **G**, **H** The effect of overexpressing TRIP13 on the phosphorylation of HAT1 was shown. **I** Co-IP assays showed the effect of overexpressing TRIP13 on HAT1 stability in the HEK293T cells treated with or without oligomycin. Data (means ± SEM, n = 3 independent experiments) were representative of three separate experiments. Compared with the indicated group, **P < 0.01, n.s. no significant differences.

We further examined whether TRIP13 affects the interaction of HAT1 and UBE4A. Co-IP results showed that TRIP13 decreased the binding of UBE4A to HAT1 (Fig. 6A). Moreover, when UBE4A was silenced, knocking down TRIP13 no longer led to HAT1 polyubiquitination or degradation (Fig. 6B). We also found that overexpression of TRIP13 effectively blocked UBE4A-induced HAT1 degradation. However, overexpression of TRIP13 hardly changed the protein levels of HAT1, even if overexpression of UBE4A when proteasome-mediated protein degradation was blocked by MG-132 (Fig. 6C, D). Additionally, as shown in Fig. 6E, F, overexpressing TRIP13 extended HAT1 protein half-life in the presence of UBE4A overexpression. These data indicate that TRIP13 competitively binds to HAT1, disrupting its interaction with UBE4A and preventing UBE4A-mediated degradation of HAT1.

To evaluate how TRIP13 regulates HAT1 stability, we first assessed HAT1 phosphorylation level but found no significant changes between TRIP13 overexpressing cells and the controls (Fig. 6G, H). We then investigated whether HAT1 stabilization is ATP-dependent, as reported for the TRIP13-p31^comet^/C-MAD2/CDC20 complex [29]. Treatment with oligomycin, an ATPase inhibitor [30], reduced the affinity between TRIP13 and HAT1 in co-IP analysis (Fig. 6I), suggesting that TRIP13’s ATPase activity is critical for maintaining the TRIP13-HAT1 interaction.

### TRIP13-HAT1 axis promotes Treg proliferation and inhibits colitis

**Fig. 7:**
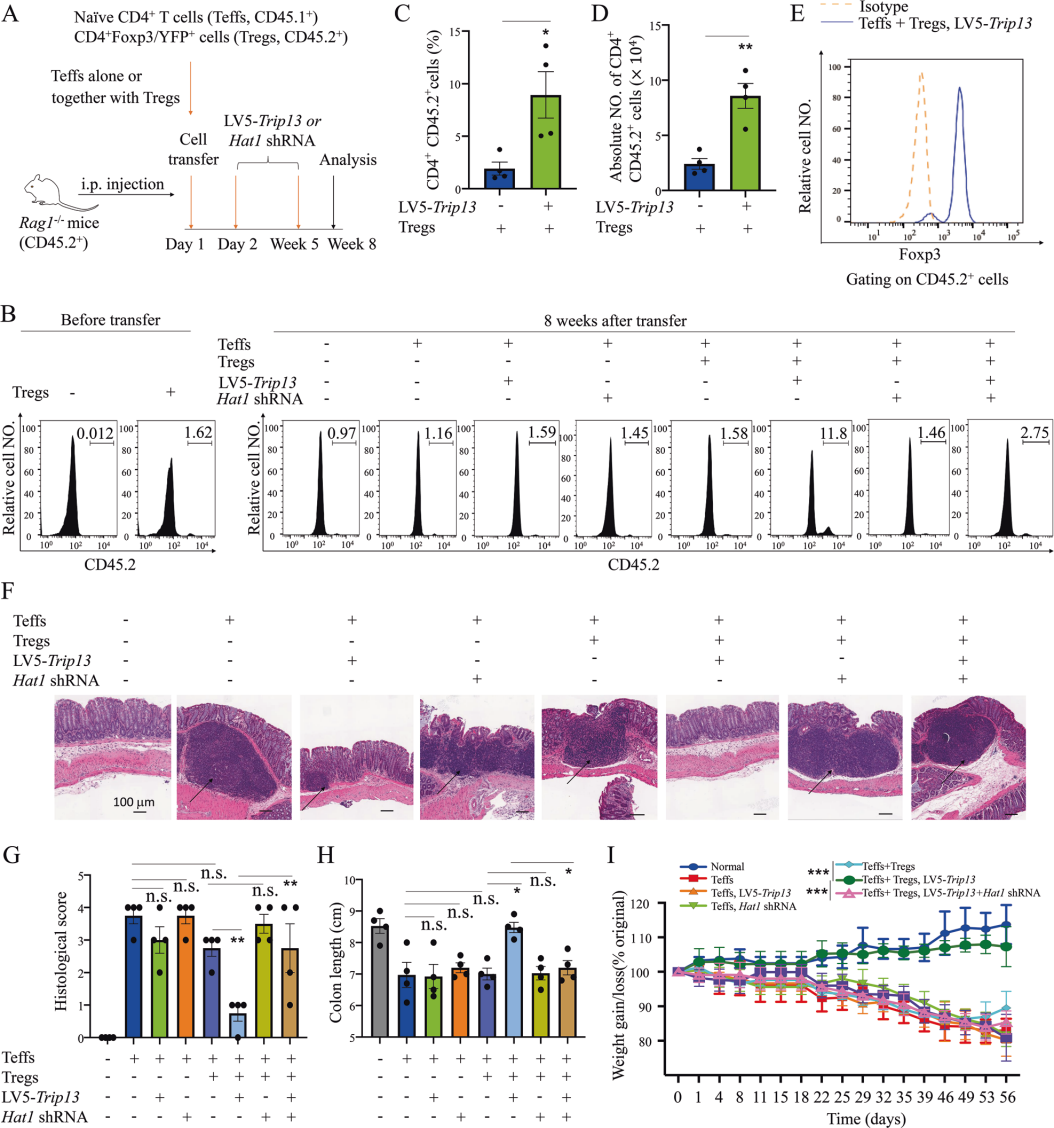
Blocking the degradation of HAT1 is crucial for promoting Treg proliferation and inhibiting the development of colitis. **A** Schematic diagram of experimental procedure. Naïve CD4^+^ T cells (Teffs, 4 × 10^5^ cells/mouse) from Ly5.1 B6 mice (CD45.1^+^) alone or together with CD4^+^Foxp3/YFP^+^ cells (Tregs, CD45.2^+^, 2 × 10^4^ cells/mouse) were injected intraperitoneally into Rag1^−/−^ mice. The mice were treated with LV5-*Trip13*, *Hat1* shRNA, or vehicle control (i.p.) once a week, starting from 2nd day after cell injection, for 5 weeks. The mouse colon and spleen were harvested on week 8 after cell transfer. The proportion of Tregs (CD45.2^+^) in colonic CD4^+^ T cells before (**B**, left panel) or 8 weeks after transfer (**B**, middle and right panel), number of splenic Tregs (**C**, **D**), Foxp3 expression on colonic Tregs **E** were analyzed by FCM. **F** H&E staining of the colon, **G** Histological score, **H** Length of the colon, and **I** Body weight gain/loss (% of initial) were shown. Arrows indicated inflammatory cell infiltrates. Data (means ± SEM) shown in (n = 4 mice) were representative of three separate experiments. Compared with the indicated group, *P < 0.05, **P < 0.01, ***P < 0.001, n.s. no significant differences.

We investigated whether TRIP13 exerts anti-inflammatory effect through Treg expansion in a mouse colitis model induced by transferring naïve CD4^+^ T cells into lymphopenic *Rag1*^−/−^ mice (CD45.2^+^). Naïve CD4^+^ T cells (flow-sorted CD4^+^CD25^-^ CD45RB^hi^ T cells, Teffs) from Ly5.1 B6 mice (CD45.1^+^) were transferred alone or co-transferred with CD4^+^ Foxp3/YFP^+^ cells (Tregs) from *Foxp3*^YFP-Cre^ mice (CD45.2^+^) to *Rag1*^−/−^ mice. Co-transfer of Tregs with a low Treg-to-Teff ratio (1.62%) was performed, followed by treatment with *Hat1* shRNA or LV5-*Trip13* (knockdown efficiency shown in Fig. S10A), starting one day after cell injection, once a week for 5 weeks (Fig. 7A). After 8 weeks, *Rag1*^−/−^ mice treated with LV5-*Trip13* exhibited a 7.5-fold increase in colonic Tregs (11.8%) compared to untreated mice (1.58%, P < 0.05, Fig. 7B, C). Tregs expansion was also observed in the spleen and mesenteric LNs (P < 0.01, Fig. S10B), indicating local proliferation rather than altered migration. Furthermore, the absolute number of colonic Tregs after the treatment with LV5-*Trip13* was markedly higher than that in untreated mice (P < 0.01, Fig. 7D). LV5-*Trip13* treatment had no effect on Foxp3 expression in the CD4^+^ CD45.1^+^ population (Fig. S10C) but significantly increased the Foxp3 expression of CD45.2^+^ cells in colonic tissue (P < 0.05, Fig. 7E), indicating that the increase in Tregs was due to the proliferative expansion of pre-existing nTregs, rather than from the conversion from induced Tregs. Furthermore, LV5-*Trip13* treatment decreased the histological scoring, increased the colon length, and increased the body weight, and this effect was reversed by *Hat1* shRNA (Fig. 7F–I).

**Fig. 8:**
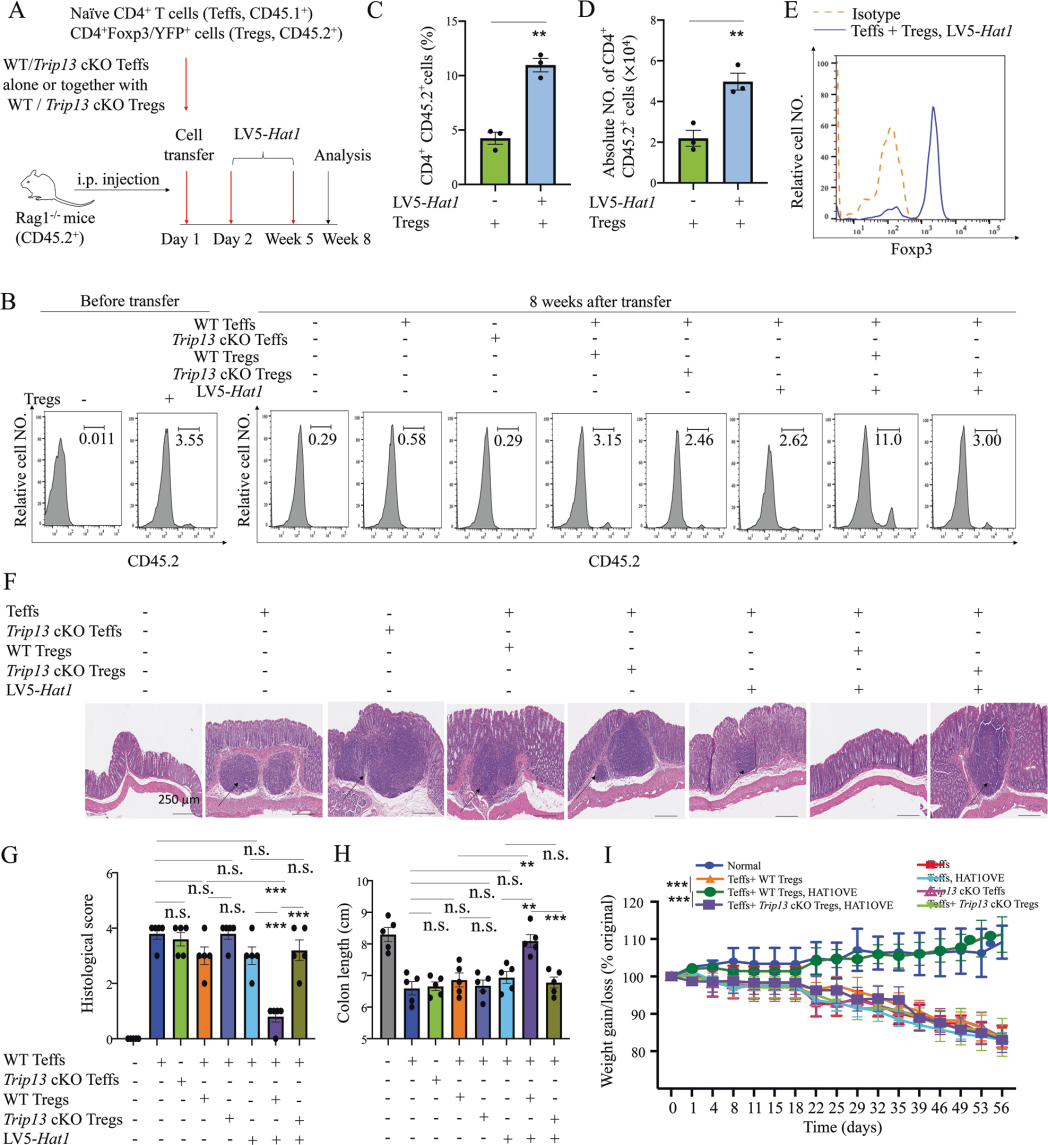
The axis of TRIP13-HAT1 plays an important role in promoting Treg proliferation and inhibiting the development of colitis. **A** Schematic diagram of experimental procedure. The proportion of Tregs (CD45.2^+^) in colonic CD4^+^ T cells before (**B**, left panel) or 8 weeks after transfer (**B**, middle and right panel), number of splenic Tregs (**C**, **D**), Foxp3 expression on colonic Tregs (**E**) were analyzed by FCM. **F** H&E staining of the colon, **G** Histological score, **H** Length of the colon, and **I** Body weight gain/loss (% of initial) were shown. Arrows indicated inflammatory cell infiltrates. Data (means ± SEM) shown in (n = 5 mice) were representative of three separate experiments. Compared with the indicated group, **P < 0.01, ***P < 0.001, n.s., no significant differences.

We then transferred Teffs derived from WT or *Trip13* cKO (*Trip13* ^fl/fl^CD4^cre^ mice) mice, alone or in combination with Tregs from either WT or *Trip13* cKO (*Trip13* ^fl/fl^*Foxp3* ^YFP-cre^ mice) mice, into *Rag1*^−/−^ mice. Tregs were co-transferred at a low Treg-to-Teff ratio (3.55%), followed by weekly intraperitoneal administration of LV5-*Hat1* starting one day after cell transfer for a total of 5 weeks (Fig. 8A). After 8 weeks, *Rag1*^−/−^ mice treated with LV5-*Hat1* exhibited a threefold increase in colonic Tregs (11.0%) compared to untreated mice (3.15%, P < 0.01, Fig. 8B, C). Moreover, the absolute number of colonic Tregs after the treatment with LV5-*Hat1* was significantly higher than in untreated mice (P < 0.01, Fig. 8D). Additionally, LV5-*Hat1* treatment alleviated colonic inflammation, as evidenced by reduced histological scores, increased colon length, and elevated body weight. Notably, these therapeutic effects were abrogated when TRIP13-deficient Tregs were transferred (Fig. 8F, I). Collectively, these findings suggest that targeting TRIP13 may represent a promising strategy for preventing colitis by promoting Treg expansion and function.

## Discussion

TNF-TNFR2 signaling is crucial for Treg activation, expansion, and phenotypical stability [5, 9, 10, 31]. Our previous research demonstrated that inhibiting two-pore channels markedly expanded Tregs in a TNF/TNFR2-dependent manner [32]. In this study, we identified *Trip13* as a potent regulator of Treg proliferation. Mechanistically, TNFR2 signaling elevated TRIP13 expression, which stabilized HAT1 protein by preventing UBE4A-mediated polyubiquitination degradation of HAT1, thus promoting Treg proliferation and protecting against colitis. In addition, TRIP13’ ATPase activity is essential for its interaction with HAT1 (Fig. S11).

Previous studies have identified the crucial role of TRIP13 in tumor immunity, where it supports tumor survival by modulating the recruitment of immune cells, including CD3^+^, CD4^+^, and CD8^+^ T cells, into the tumor microenvironment [23]. Depletion of TRIP13 enhances immune activation within the tumor microenvironment, ultimately slowing tumor progression [33]. Additionally, TRIP13 has been implicated in activating TGF-β signaling [34], a pathway that regulates Treg generation and homeostasis [35]. Consistent with these studies, our findings showed that *Trip13* is highly expressed in TNFR2^+^ Tregs, and TNF induced TRIP13 upregulation through TNFR2. Furthermore, lentivirus-mediated TRIP13 overexpression promoted Tregs expansion, while lentivirus-expressing *Trip13* shRNAs reduced Treg numbers in a TNFR2-dependent manner both in vitro and in vivo. Overexpression of TRIP13 also promoted immunosuppressive activity of Treg cells in vivo. We also generated cKO mice and found that colitis exacerbation following TRIP13 ablation in Treg cells was obtained after TNBS induction. Additionally, the proportion and number of Treg cells were unchanged in the steady state. However, the proportion and number of Treg cells were reduced at day 12 in the colon of cKO mice after TNBS induction. Consequently, the control of colitis progression by TRIP13-expressing Tregs is regulated in the inflammatory condition. Our work collectively provided substantial evidence that TRIP13 suppressed the inflammatory response by regulating the number and activity of Tregs.

Using co-IP coupled with mass spectroscopy, we identified HAT1 as a binding partner of TRIP13 in Tregs. Our findings suggest that TRIP13 positively regulates HAT1 expression, and the ability of lentivirus encoding *Hat1* to promote Treg expansion was abolished in mice infected with lentivirus expressing *Trip13* shRNA. This is significant as HAT1 was previously shown to interact with Foxp3, playing a crucial regulatory role in Tregs [25]. On a molecular level, Foxp3, when bound to the CCR4 promoter, recruits HAT1, leading to acetylation of the promoter. This upregulates the CCR4 receptor, enhancing Treg infiltration [25]. Collectively, these findings indicate that HAT1 exerts immunomodulatory function by controlling Treg infiltration in cancer. In our study, HAT1 was shown to promote Treg expansion by increasing Foxp3 expression. Further investigation is needed to elucidate the underlying mechanism of HAT1 stability-mediated Treg proliferation.

Previous studies have established a correlation between HAT1 expression and function in immunoinflammatory diseases [36–39], including chronic obstructive pulmonary disease (COPD) and atherosclerosis, where the immune response plays an important role in pathogenesis and outcomes. In COPD, HAT1 has been shown to potentially downregulate TLR4 expression, inhibiting the inflammatory response and thereby alleviating disease progression [39]. In atherosclerosis, HAT1 appears to play a dual role. On one hand, TLR4-triggered inflammation activates HATs in macrophages, promoting chromatin remodeling and increasing Nox5 expression, which leads to elevated ROS production [40]. However, HAT1 also seems to act as an anti-agent in atherosclerosis by facilitating the transformation of macrophages into foam cells, which reduces cholesterol accumulation and helps inhibit atherosclerosis development [41]. In our study, we further elucidated the anti-inflammatory action of HAT1 in Tregs, suggesting that this characteristic of HAT1 could be therapeutically exploited for treating inflammatory disorders. Moreover, TRIP13 overexpression may have a broader impact on Treg proliferation, beyond its interaction with HAT1 to inhibit HAT1 degradation, a hypothesis that warrants further investigation.

At the molecular level, TRIP13 overexpression increased protein levels without affecting mRNA levels, suggesting that HAT1 expression is regulated through post-translational modifications. HAT1 was previously reported to interact with HIF2A, promoting its stability by acetylation in cancer cells [42]. Similarly, HAT1 inhibited the degradation of EZH2 by disrupting the interaction between ubiquitin ligase UBR4 and EZH2 [43], thereby preserving EZH2 stability and enhancing its protein levels [43]. In our study, UBE4A bound to HAT1, promoting K48- and K29-linked polyubiquitination that led to proteasome-mediated degradation of HAT1. UBE4A has previously been shown to promote colorectal cancer cell proliferation [44], though its role in Treg cells remains unexplored. K48-linked and K29-linked polyubiquitination were previously reported to be required for protein degradation [45]. We identified two critical ubiquitination sites of the HAT1 protein at K23 and K33 and demonstrated that UBE4A induces K48-linked and K29-linked polyubiquitination at these sites, resulting in HAT1 protein degradation. Furthermore, the U-box domain and Ufd2P core domain of UBE4A (located 330–933aa and 952–1021aa) are essential for its ubiquitin ligase activity [46]. Our study confirmed that UBE4A binds to HAT1, triggering its degradation and inhibiting Treg proliferation. Further investigation is required to determine whether UBE4A and other members of the U-box protein family play distinct or overlapping roles in regulating Treg proliferation and their immunosuppressive function.

The ATPase activity of TRIP13 plays a critical role in stabilizing TRIP13-HAT1 interaction, consistent with previous findings showing that the stability of TRIP13-p31^comet^/C-MAD2/CDC20 complex also depends on TRIP13’s ATPase activity [29]. However, the interaction between TRIP13 and HAT1 may be influenced by other proteins or factors, as indicated by the reduced affinity between TRIP13 and HAT1 following oligomycin treatment, rather than a complete loss of binding. This raises important questions about potential additional regulators of the TRIP13-HAT1 axis.

Tregs are essential for the maintenance of immune homeostasis and self-tolerance, as they regulate immune responses [47]. Expanding Tregs in vivo offers a promising strategy for treating various inflammatory diseases, including autoimmune diseases, allergies, allograft rejection, and GvHD [47]. In our study, we demonstrate that TRIP13 overexpression promotes Treg expansion and consequently inhibits the development of colitis in a mouse model induced by T cell transfer. Conversely, genetic ablation of TRIP13 substantially reversed the effects induced by HAT1 overexpression, including enhanced Treg expansion and attenuation of colitis, suggesting that TRIP13 is required for HAT1-mediated functions. However, systemic overexpressing TRIP13/HAT1 by lentivirus could cause unwanted side effects. These off-target effects might be mitigated by engineering delivery method to specifically target Tregs. Therefore, overexpression of TRIP13 or knockdown of UBE4A may represent a promisinig strategy to induce immune tolerance. Further investigation is required to determine whether the effect of TRIP13 or UBE4A on Treg proliferation can be applied to treat other inflammatory diseases. Although the precise mechanism by which the TRIP13-HAT1 axis confers specificity in gene regulation in Tregs remains unclear, the observed biological outcomes suggest that the TRIP13-HAT1 pathway may represent a promising therapeutic avenue, pending further mechanistic validation.

In conclusion, our findings demonstrate that TRIP13 overexpression enhances the proliferation of immunosuppressive Tregs by inhibiting HAT1 degradation through its interaction with this protein. This elucidates the cellular and molecular mechanism underlying the anti-inflammatory impact of the TRIP13-HAT1 axis. These findings pave the way for the development of novel TRIP13 agonists as potential treatments for inflammatory conditions, including autoimmune diseases, allergies, allograft rejection, and GvHD.

## Materials and methods

### Reagents

Antibodies purchased from BD Pharmingen (San Diego, CA) include: PerCP-Cy5.5 anti-mouse CD3 (145-2C11), PE anti-mouse CD4 (GK1.5), PerCP-Cy5.5 anti-mouse CD25 (PC61), PerCP-Cy™5.5 anti-mouse TCRβ Chain (H57-597), and PE anti-mouse CD25 (PC61). Antibodies purchased from eBioscience include: PE-Cy7 anti-mouse CD4 (GK1.5), APC anti-mouse/rat Foxp3 staining set (FJK-16s), FITC anti-human Ki-67 Monoclonal Antibody (20Raj1), and FITC anti-mouse CD45RB (C363.16A). Antibodies purchased from Biolegend include: FITC anti-mouse CD45.1 (A20), PerCP-Cyanine5.5 anti-mouse CD45.2 (104), purified anti-human CD3ε (clone: OKT3). Antibodies purchased from Proteintech include: TRIP13 polyclonal antibody (19602-1-AP), GAPDH (human specific) recombinant antibody (80570-1-RR), and FOXP3 polyclonal antibody (22228-1-AP). Antibodies purchased from Abcam include: anti-KAT1/HAT1 antibody[EPR18775] (AB194296), anti-DDDDK tag (Binds to FLAG® tag sequence) [EPR20018-251] (ab205606), anti-6X His tag® antibody [HIS.H8] (ab18184) and goat anti-rabbit IgG H&L (Alexa Fluor® 488) (ab150077). Antibody purchased from Cell Signaling Technology includes: GAPDH (D4C6R) mouse monoclonal antibody (97166). Antibody purchased from Sigma-Aldrich includes: monoclonal anti-HA antibody produced in mouse (H9658). Antibody purchased from Abclonal includes: rabbit anti Myc-Tag polyclonal antibody (AE009). *InVivo*MAb anti-mouse TNFR2 (CD120b) (TR75-54.7) was purchased from Bioxcell. Human TNFR2 neutralizing antibody was purchased from SinoBiological (10417-R00N6). Recombinant mouse IL-2 (550069), human IL-2 (554603), human TNF (554618), and mouse TNF (554589) were obtained from BD Biosciences. Anti-PE microbeads (130-048-80) and mouse CD4^+^ T cell isolation kit (130-104-454) were obtained from Miltenyi Biotec.

### RNA extraction and whole transcriptome sequencing

Total RNA was extracted from TNFR2-deficient Tregs and TNFR2^+^ Tregs, RNA integrity was detected by the Agilent2100, and RNA concentration was accurately determined using NanoDrop. Magnetic beads with oligo-dT were used to isolate mRNA from total RNA, and the captured mRNA was fragmented. The first and second strands of cDNA were then synthesized using reverse transcriptase, and the reverse transcription product was end-repaired, followed by the addition of a base A at the 3’ end. The fragment is then connected to the sequencing adapter. The ligation product is purified, the product with incomplete ligation and the empty linker self-ligation product are removed, and PCR amplification is performed using primers that complement the linker sequence. Finally, the sequencing library was purified by magnetic beads. The library concentration was detected by Qubit, and the length of the library fragments was detected by Agilent fragment analyzer. PE150 sequencing of libraries using the Illumina Novaseq 6000 sequencing platform.

### In vitro cell culture and transfection

MACS-sorted 6-week-old female C57BL/6J mouse CD4^+^ T cells were transfected with the following lentivirus: LV5-*Trip13*, *Trip13* shRNA, or LV5-*Tnfr2*. All transfections were performed in the presence of IL-2 (50 pg/mL). For proliferation assays, in some experiments, MACS-sorted CD4^+^ T cells were labeled with CFSE. After 72 h, Treg proliferation was assessed by FCM or using a [^3^H] thymidine incorporation assay. For Treg differentiation assays, MACS-sorted naïve CD4^+^ T cells were transfected with LV5-*Trip13* or vehicle control lentivirus. Transfections were performed with or without murine IL-2 (50 pg/mL) or TNF (10 ng/mL), following previously established protocols [48, 49]. For protein interaction studies, MACS-sorted Treg cells or HEK-293T cells were transfected with Flag-TRIP13, Myc-HAT1, or HA-UBE4A plasmids. In some experiments, cells were then treated with 10 μM MG-132 (Sigma-Aldrich, M8699) for 8 h or treated with 50 μg/ml CHX (MCE, HY-12320) and harvested at the indicated time points. Cells were lysed and subjected to Western blot with the indicated antibodies.

For human Treg cell culture, peripheral blood mononuclear cells (PBMCs) from healthy human blood donors were isolated by gradient centrifugation over Lymphoprep (STEMCELL, #07851) and rinsed with Hanks’ balanced salt solution or phosphate-buffered saline. Erythrocytes were lysed in 10 mm Tris (pH 7.4) and 150 mm NH_4_Cl at 37 °C for 10 min. The cells were then pelleted, resuspended in RPMI medium (Gibco) supplemented with 10% fetal calf serum (Gibco), and plated. CD4^+^CD25^+^ T cells were purified from PBMCs using CD4^+^CD25^+^ regulatory T cell isolation kit (Miltenyi Biotec, Germany), following the manufacturer’s instructions. The purified cells were seeded in 96-well plates and transfected with the *Trip13* shRNA or LV5-*Hat1* in the presence of recombinant human IL-2 (300 IU/mL), anti-human CD3 mAb (OKT-3; BioLegend, San Diego, CA), and TNFR2 agonistic antibody (clone MR2-1, Hycult Biotech) for 7 days, following previous study [50].

### Plasmids and transfection, and lentivirus particle generation

HEK293T cells were transfected with plasmids at 80% confluence using Lipo8000™ Transfection Reagent (Beyotime, C0533). Transfected cells were cultured for 48 h before harvesting for western blot analysis or immunoprecipitation. The following plasmid constructs were generated: TRIP13 full-length and deletion mutants were constructed by PCR amplification of mouse TRIP13 full-length or partial coding sequences and cloned into mammalian expression vectors with Flag tag. HAT1 full-length and deletion mutants were generated by PCR amplification of HAT1 and cloned into mammalian expression vectors with Myc-tag. UBE4A full-length and deletion mutants were generated by PCR amplification of UBE4A and cloned into mammalian expression vectors with HA-tag. Ubiquitylation and its mutant constructs were generated using the site-directed mutagenesis kit (Agilent) as described previously [51] and cloned into mammalian expression vectors with His-tag. For some experiments, MACS-sorted Tregs were transfected with lentivirus particles. The lentivirus particles (LV5-*Trip13*, LV5-*Hat1*, LV5-*Tnfr2*, *Trip13* shRNAs, *Hat1* shRNA, LV5-*Ube4a*, and negative controls) were generated by Nan Jing Banma Yu, Co., Ltd. (Nan Jing, China). *Trip13* shRNA target sequences: *Trip13* shRNA1. GACACAGAACTAAAGGCTAAA; *Trip13* shRNA2. CCGAGTAGTCAATGCTGTGTT. *Hat1* shRNA target sequence: GAAGCTACAGACTGGATATTA. *Ube4a* shRNA target sequence: GCAGTCCGGTAGTGTATTTGG.

### In vivo administration of lentivirus particles

6-week-old female C57BL/6J mice were intraperitoneally (i.p.) injected with 100 µl lentivirus encoding either *Trip13*, *Trip13* shRNAs, *Hat1* shRNA, *Hat1*, or *Ube4a* (1 × 10^8^ TU ml^-1^) or with a control virus. Injections were given for 3 days. In some experiments, *Tnfr2* KO mice were i.p. injected with 100 μL lentivirus encoding *Trip13*. On day 4, the mice were sacrificed, and lymphoid tissues, including the spleen, axillary lymph nodes, inguinal lymph nodes, and mesenteric lymph nodes, were harvested to assess the number and phenotype of Tregs.

For the Treg functional assay, CFSE-labeled responder CD4^+^Foxp3^-^ cells (5 × 10^4^ cells/well), sorted from *Foxp3*^YFP-cre^ mice, were cultured either alone or co-cultured with flow-sorted CD4^+^Foxp3/YFP^+^ Tregs from and *Foxp3*^YFP-Cre^ or *Trip13* ^fl/fl^*Foxp3*^YFP-cre^ mice at responder-to-Treg ratios of 10:0, 10: 5, and 10:10. The cells were stimulated with 3000-rad irradiated APCs (CD4-depleted splenocytes, 2 × 10^5^ cells/well) and soluble anti-CD3 Ab (BD, Clone 145-2C11, 1 µg/ml). After 72 h of incubation, responder cell proliferation was analyzed by FCM based on CFSE dilution.

### TNBS induced colitis model

TNBS-induced colitis model was conducted according to previous protocol. In detail, to induce TNBS-induced colitis, mice were presensitized with 1% (w/v) TNBS (Sigma, St. Louis, MO, USA, P2297) on day 1 to induce acute TNBS-induced colitis. On day 8, they were intracolonally administered with 150 μL of TNBS solution (2.5% TNBS in 50% ethanol). On the 12th day following euthanasia, colon tissues were collected for pathological analysis, HE staining, and flow cytometry detection.

### T cell transfer model of colitis

Naïve CD4^+^ CD25^−^ CD45RB^hi^ T cells (Teffs) were flow-sorted from WT congenic B6 mice (Ly5.1 mice, also known as CD45.1^+^ mice, 4 × 10^5^ cells/mouse) and injected i.p. into *Rag1*^−/−^ (CD45.2^+^) recipient mice, either alone or in combination with CD4^+^ Foxp3/YFP^+^ Treg cells (CD45.2^+^, 2 × 10^4^ cells/mouse). In some experiments, Teff cells derived from WT or *Trip13* cKO mice were transferred alone or co-injected with Tregs from either WT or *Trip13* cKO mice. In experiments involving co-transfer Teffs and Tregs, cells were sorted from WT congenic B6 mice (CD45.1^+^) and CD4^+^ Foxp3/YFP^+^ mice (CD45.2^+^), mixed at 20: 1 ratio, and i.p. injected into *Rag1*^−/−^ mice. Mice were then treated with 100 μl of lentivirus particles (1 × 10^8^ TU ml^−1^) or vehicle control, starting on the 2nd day after cell transfer. The lentivirus was administered once a week for 5 weeks. The clinical symptoms of colitis, such as rectal bleeding, diarrhea, rough or hunched posture, and body weight changes, were monitored weekly. Mice that lost more than 20% of their initial body weight or showed severe signs of disease were euthanized. All mice were sacrificed at week 8 post-transfer, and the colon and lymphoid tissues were harvested for FCM analysis. For histopathological analysis, colon tissues were fixed in 10% neutral buffered formalin, embedded in paraffin, and stained with hematoxylin and eosin. The histopathological scoring was assessed based on previously described grading scales [52].

### Quantitative reverse transcription PCR (qRT-PCR)

To determine the fold change of *Tnfr2*, *Trip13*, *Hat1*, and *Ube4a* mRNA expression, total RNA was extracted and purified using Qiagen RNA following the manufacturer’s protocol. qRT-PCR was conducted using the Qiagen RT-PCR kit with SYBR Green dye and gene-specific primers (Qiagen). Mouse *Gapdh* was used as the internal control for normalization. The qRT-PCR amplifications were set at 95 °C for 3 min, then 40 cycles of thermal cycling at 95 °C for 5 s and 60 °C for 30 s. Quantification was performed by normalizing Ct (cycle threshold) values with *Gapdh* Ct (mRNA) and analyzed with the 2^−ΔΔCT^ method. The following primers were used in this experiment. Mouse *Tnfr2*: ACACCCTACAAACCGGAACC (forward primer) and AGCCTTCCTGTCATAGTATTCCT (reverse primer). Mouse *Trip13*: TCATATATCCTCGTCAGCAGC (forward primer) and GAGGCCCTCACTCTTCCTTG (reverse primer). Mouse *Hat1*: TATGGCAATACAGGCACAGC (forward primer) and TCAGCATCGCTCATGTCAG (reverse primer). Mouse *Ube4a*: CCTTTCAGGCTCCGGTACTT (forward primer) and TTCTTGGTGTGTCCGGAAGG (reverse primer). Mouse *Gapdh*: ACCCTTAAGAGGGATGCTGC (forward primer) and CCCAATACGGCCAAATCCGT (reverse primer).

### Flow cytometry

For flow cytometry analysis, cells were first blocked to prevent Fc receptor (FcR) binding and then incubated with appropriately diluted antibodies targeting the specific cell markers of interest. After antibody incubation, cells were suspended in FCM buffer for analysis. Data acquisition was performed by BD FACSAria III flow cytometer or BD LSRFortessa flow cytometer. Collected data were analyzed with FlowJo software (Tree Star Inc., Ashland, OR).

### Co-Immunoprecipitation (co-IP) and mass spectrometry analyses

Co-IP analysis was performed using the Co-Immunoprecipitation kit (Thermo Scientific) following the manufacturer’s instructions. Antibodies used for Co-IP include: Anti-TRIP13 (Proteintech, 19602-1-AP), Anti-HAT1 (Abcam, ab194296), Anti-HA tag (CST, #3724), Anti-DYKDDDDK tag (CST, #2368), Anti-myc (ABclonal, #AE070), Anti-6X His tag (Abcam, ab18184), and Anti-IgG (Abcam, ab6709). The antibodies were immobilized using amino link plus coupling resin. Cell lysates were first pre-cleared using the control agarose resin. Pre-cleared lysates were then added to spin columns containing antibody-coupled resin and incubated overnight at 4 °C to allow for protein-antibody binding. After incubation, bound proteins were eluted using the provided elution buffer. Mass spectrometry was conducted in collaboration with Shanghai Shenggong BioEngineering Co., LTD. (Shanghai, China) to identify the interacting proteins.

### Western blotting

Protein samples were subjected to separation on 10% SDS-PAGE gels or supersep^TM^ phos-tag^TM^ precast gels (Wako, Japan). Following electrophoresis, proteins were transferred onto PVDF membranes. The membranes were blocked with either 5% nonfat milk or 5% BSA for nonspecific binding reduction. After blocking, membranes were probed with primary antibodies overnight at 4 °C. The next day, the membranes were incubated with anti-rabbit IgG (H + L) (DyLight™ 800 4X PEG) conjugated (CST, #5151) or anti-mouse IgG (H + L) (DyLight 800 4X PEG) conjugated (CST, #5257) secondary antibodies (1: 10,000) for 2 h at room temperature. Protein bands were visualized using the Odyssey CLx LI-COR imaging system.

### Study approval

Six-week-old female C57BL/6 mice were purchased from the Laboratory Animal Center of Nantong University (Jiangsu, China). Foxp3^YFP-Cre^ (Cat. NO. C001467), CD4^Cre^ (Cat. NO. C001351), and CD45.1 mice were purchased from Cyagen Biosciences Inc. (Guangzhou, China). *Tnfr2* KO, *Rag1*^−/−^, *Itgax*^Cre^, and *Trip13*^flox/flox^ (*Trip13fl/fl*, Cat. NO. NM-CKO-226225) mice were purchased from the Shanghai Research Center for Model Organisms (Shanghai, China). The *Foxp3*^YFP-Cre^ littermate mice were used as the controls. The animal study was conducted in accordance with institutional ethical regulations, and all experimental procedures were approved by the Ethics Committee of Nantong University (No. S20221101-099). Mice were randomly assigned to treatment groups, and group allocation was double-blinded.

Peripheral blood from healthy human blood donors was obtained from the Affiliated Hospital of Qingdao University, China. This study adhered to the principles outlined in the Declaration of Helsinki and was approved by the Ethics Committee of the Affiliated Hospital of Qingdao University (NO. QYFYWZLL28514). All patients provided written informed consent after being fully informed about the study’s purpose, potential risks, and the use of their specimens.

### Statistical analysis

Comparisons between the two groups were analyzed by two-tailed Student’s *t*-test with GraphPad Prism 7.0. (GraphPad, San Diego, CA). Student’s *t*-test (2-tailed) was used when only two sets of data were processed. For comparisons involving more than two groups, one-way ANOVA was performed. In some experiments, two-way ANOVA was performed. A *p* value less than 0.05 was considered statistically significant. In the in vivo studies, *n* represents the number of individual mice used in the experiments. In the in vitro cell culture studies, *n* (represented as dots in bar charts) refers to the number of independent experiments conducted.

## Supplementary information

Supplementary Information

Supplemental data set 1

Raw data of Western Blot

## Data Availability

All data necessary to evaluate the conclusions presented in the paper are included in the main text and/or the Supplementary Materials. Additional data related to this study can be requested from the corresponding authors.
