# Supplementary material for: TRIP13 promotes the expansion and immunosuppression of CD4+Foxp3+ regulatory T cells by sustaining HAT1 stability
Source: Cell Death Dis. 2026 Jan 14;17(1):32. doi: 10.1038/s41419-025-08214-7 (PMC12804696; doi:10.1038/s41419-025-08214-7)
Supplement: Supplementary file 1 — Supplementary Information [file 41419_2025_8214_MOESM1_ESM.pdf]

Supplemental Materials

TRIP13 Promotes the Expansion and Immunosuppression of CD4$^+$Foxp3$^+$ Regulatory T Cells by Sustaining HAT1 Stability

Tianzhen He[1*†], Liwen Zhao[1†], Chu-Ting Feng[1], Li-Ya Zhao[1], Shengnan Jing[1], Han Yang[2], Ke Wang[2], Siyu Ye[1], Yingchun Zhao[3], Ying Yu[3], Zhuting Fu[4], Chon-Kit Chou[5], Xin Chen[5], Yong-Jing Gao[1*]

Fig. S1. Validation of the efficiency of knockdown and overexpression of *Trip13* and *Tnfr2*.

Fig. S2. *Trip13* shRNA reduces overexpressing TRIP13-mediated Tregs proliferation.

Fig. S3. The proportion and number of Tregs in cKO mice in the steady state and total leukocyte infiltration in the inflamed colon remain unchanged.

Fig. S4. TRIP13 in dendritic cells is dispensable for Treg proliferation and function and its overexpression does not influence Treg differentiation.

Fig. S5. Overexpressing TNFR2 or TRIP13 regulates HAT1 protein expression in Tregs.

Fig. S6. TRIP13 increases HAT1 protein but not its mRNA.

Fig. S7. The effect of overexpressing TRIP13 on the polyubiquitination of HAT1.

Fig. S8. The specific ubiquitination sites of HAT1 for UBE4A-induced polyubiquitination degradation.

Fig. S9. The identification of the UBE4A binding domains of HAT1 and validation of UBE4A overexpressing efficiency.

Fig. S10. Validation of the knockdown efficiency of *Hat1* and the effect of overexpressing TRIP13 on the proportion of CD4$^+$CD45.2$^+$ T cells in lymphoid tissues and Foxp3 expression in CD45.1$^+$ cells.

Fig. S11. Schematic model of the TNFR2-TRIP13-HAT1 axis in regulating Treg proliferation and colitis development.

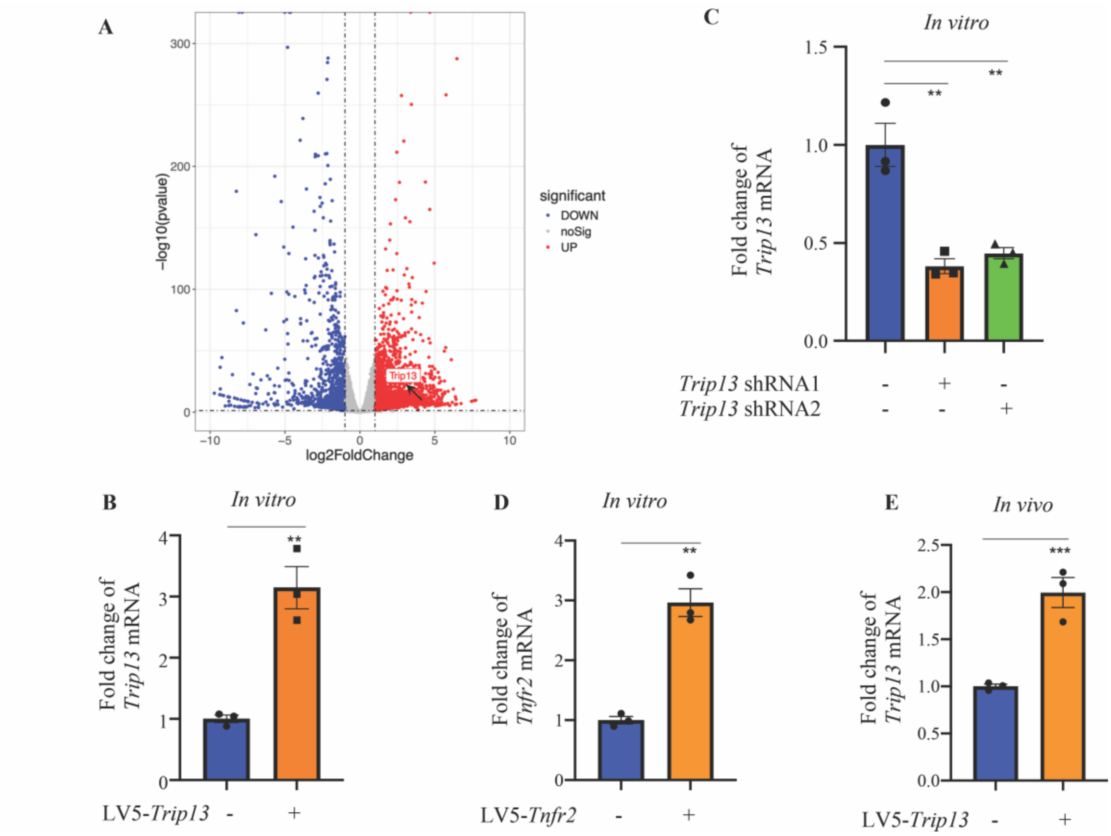

Fig. S1. Analysis of RNA-seq, validation of the efficiency of knockdown and overexpression of *Trip13* and *Tnfr2*. (A) Volcano plot shows significantly different genes in TNFR2^{+} Treg cells compared to TNFR2 -deficient Treg cells, based on RNA-seq analysis. **(B-D)** CD4^{+} T cells were MACS-sorted from the lymph nodes and spleen of C57BL/6J mice, then transfected with LV5-*Trip13*, *Trip13* shRNA1, *Trip13* shRNA2, or LV5-*Tnfr2*. The mRNA expression of *Trip13* (B-C) and *Tnfr2* (D) were analyzed by qRT-PCR. (**E**) C57BL/6J mice were intracolonally administered with TNBS on day 1 and day 8, and then injected intraperitoneally (i.p.) with LV5-*Trip13* or negative control for 3 days. CD4^{+} CD25^{+} T cells were sorted from the lymph nodes and spleen 24 h after the last injection, and the mRNA expression of *Trip13* was determined by qRT-PCR (E). Data are presented as means ± SEM (A-C: n=3 independent experiments; D: n=3 mice) and are representative of three separate experiments. ** $P < 0.01$, *** $P < 0.001$.

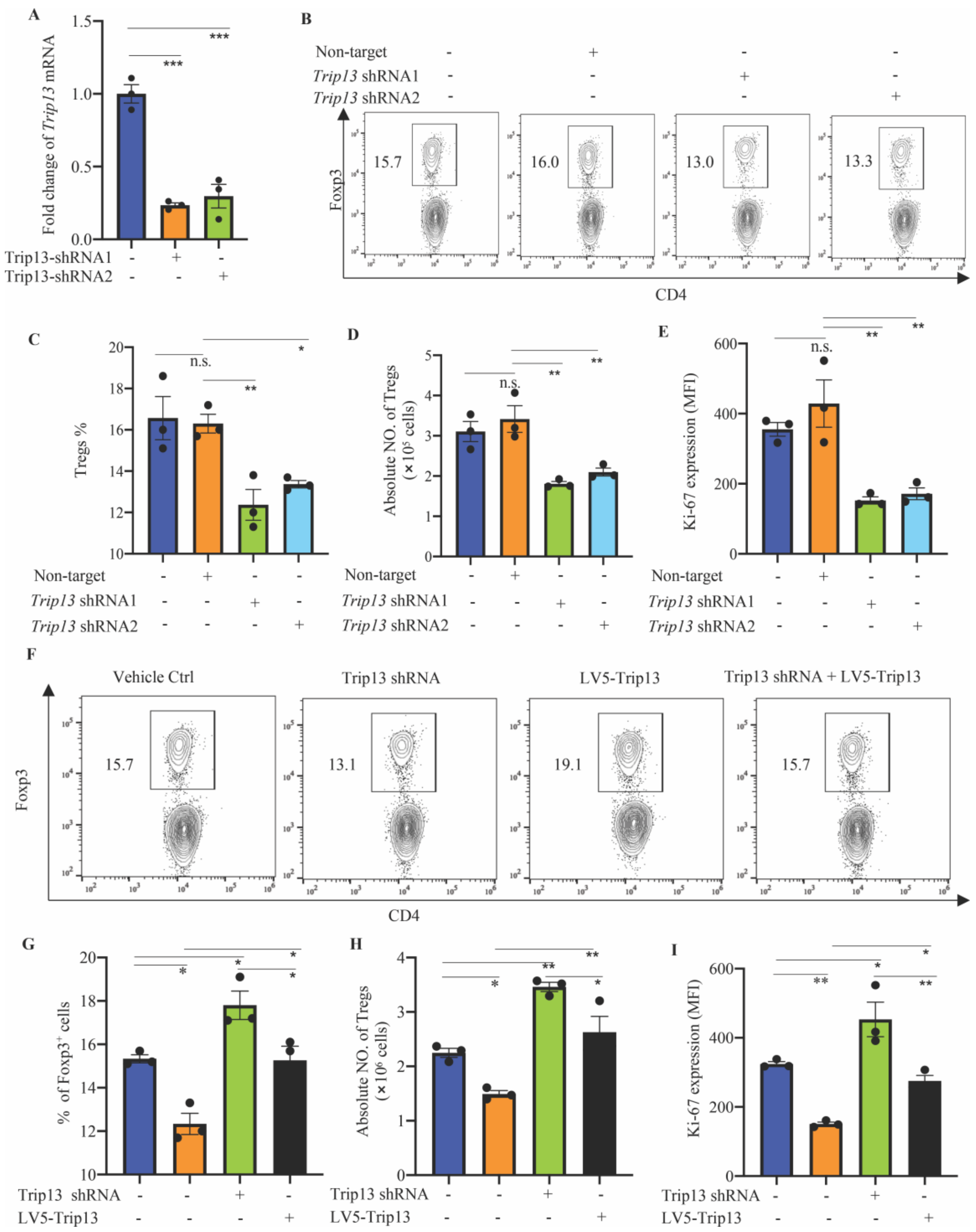

Fig. S2. *Trip13* shRNA reduces overexpressing TRIP13-mediated Tregs proliferation. (A) WT mice were intracolonally administered with TNBS on day 1 and day 8, and then i.p. injected with *Trip13* shRNA1, *Trip13* shRNA2 or non-targeting shRNA for 3 days. $CD4^{+}CD25^{+}$ T cells were sorted by MACS 24 h after last injection, and *Trip13* mRNA expression (A) was analyzed by qRT-PCR. **(B-E)** The proportion (B and C), number of colonic Tregs (D), and Ki-67 expression (E) after

i.p. injection with *Trip13* shRNAs (*Trip13* shRNA1 and *Trip13* shRNA2) in WT mice. Typical FCM plots show the proportion of gated cells. **(F-I)** WT mice were intracolonally administered with TNBS on day 1 and day 8, and then injected with *Trip13 shRNA*1 *or/and* LV5-*Trip13* (i.p.) for 3 days, 24 h after last injection, representative FCM plots (F), the proportion (G), and the number (H) of colonic Tregs and expression of Ki-67 (I) were analyzed by FCM. For representative FCM plots, the number indicated the proportion of gated cells. Data are presented as means ± SEM (n=3 mice) and are representative of three separate experiments. * $P < 0.05$, ** $P < 0.01$, *** $P < 0.001$.

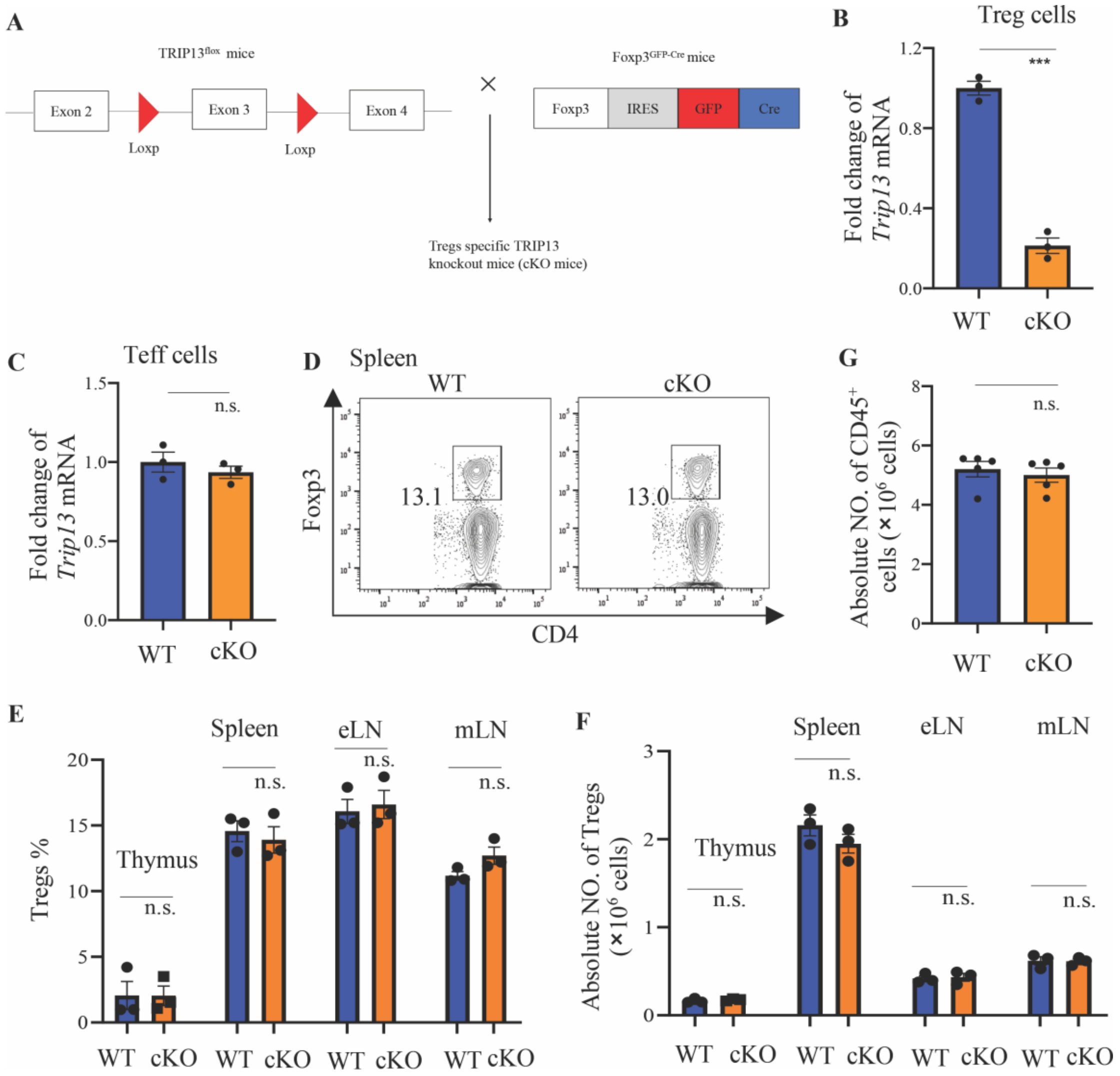

Fig. S3. The proportion and number of Tregs in cKO mice in the steady state and total leukocyte infiltration in the inflamed colon remain unchanged. (A) A strategy for generating cKO mice was shown. **(B-C)** The efficiency of the knockout of *Trip13* in Tregs and Teffs of cKO mice was validated. **(D-F)** The percentage and number of Treg cells in lymphoid tissues were shown. **(G)** Total leukocyte infiltration ($CD45^+$) in the inflamed colon of cKO mice after TNBS induction was shown. Data are presented as means ± SEM (n=3 or 5 mice) and are representative of three separate experiments. *** $P < 0.001$, n.s.: no significant differences.

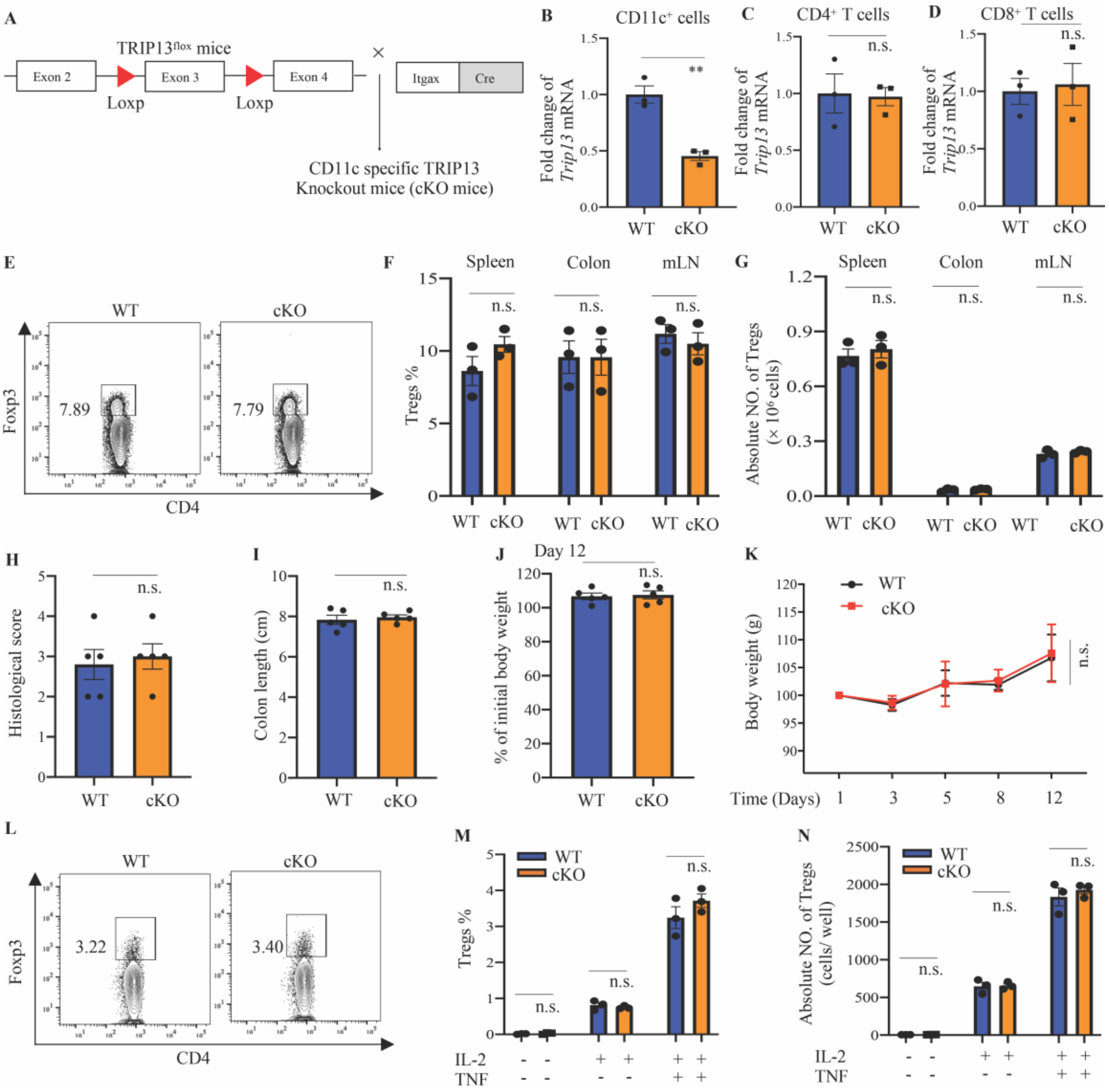

Fig. S4. TRIP13 in dendritic cells is dispensable for Treg proliferation and function and its overexpression does not influence Treg differentiation. (A) A strategy for generating cKO mice was shown. **(B-D)** The efficiency of knockout of *Trip13* in CD11c^{+} cells (B), CD4^{+} T cells (C) and CD8^{+} T cells (D) were validated. **(E-K)** The proportion (E-F), number of colonic Tregs (G), and development of colitis (H-K) after colitis induction by TNBS in *Foxp3*$^{\text{YFP-cre}}$ and *Trip13* $^{\text{fl/fl}}$*Cd11c* ^{cre} mice were shown. Typical FCM plots display the proportion of gated cells. The colon, spleen, and lymph nodes of *Foxp3*$^{\text{YFP-cre}}$ and *Trip13*$^{\text{fl/fl}}$*Cd11c*^{cre} mice were harvested. **(L-N)** Overexpression of TRIP13 displays no impact on Treg differentiation *in vitro*. Data are presented as means ± SEM (n=3 mice) and are representative of three separate experiments. ** $P < 0.01$.

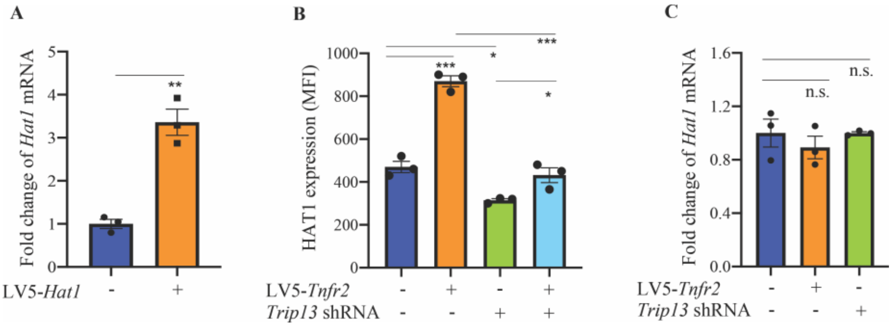

Fig. S5. Overexpressing TNFR2 or TRIP13 regulates HAT1 protein expression in Tregs. (A-C) WT mice were intracolonally administered with TNBS on day 1 and day 8, and then i.p. injected with LV5-*Hat1*, *Trip13* shRNA1, LV5-*Tnfr2,* or the control lentivirus for 3 days. $CD4^+$ $CD25^+$ T cells were MACS-sorted from the spleen and lymph nodes 24 h after the last injection. (A)The mRNA expression of *Hat1* was analyzed by qRT-PCR. (B) The protein expression of HAT1 in Tregs was analyzed by FCM. (C) The mRNA expression of *Hat1* was analyzed by qRT-PCR. Data are presented as means ± SEM (n=3 mice) and are representative of three separate experiments. * P < 0.05, ** P < 0.01, *** P < 0.001.

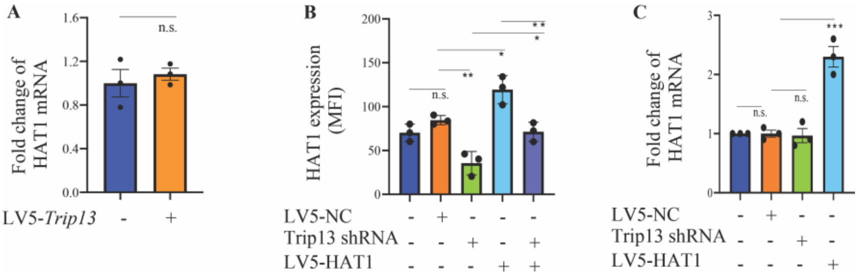

Fig. S6. TRIP13 increases HAT1 protein but not its mRNA. (**A**) MACS-sorted CD4$^+$CD25$^+$ T cells were transfected with LV5-*Trip13*, and the *Hat1* mRNA was subsequently determined by qRT-PCR. **(B-C)** WT mice were intracolonally administered with TNBS on day 1 and day 8, and then i.p. injected with *Trip13* shRNA or/and LV5-*Hat1* for 3 days. CD4$^+$ CD25$^+$ T cells were MACS-sorted from the spleen and lymph nodes one day after the last treatment. (B) The HAT1 protein level in Tregs was analyzed by FCM. (C) The *Hat1* mRNA level was detected by qRT-PCR. Data are presented as means ± SEM (n=3 mice) and are representative of three separate experiments. * P < 0.05, ** P < 0.01, *** P < 0.001, n.s.: no significant differences.

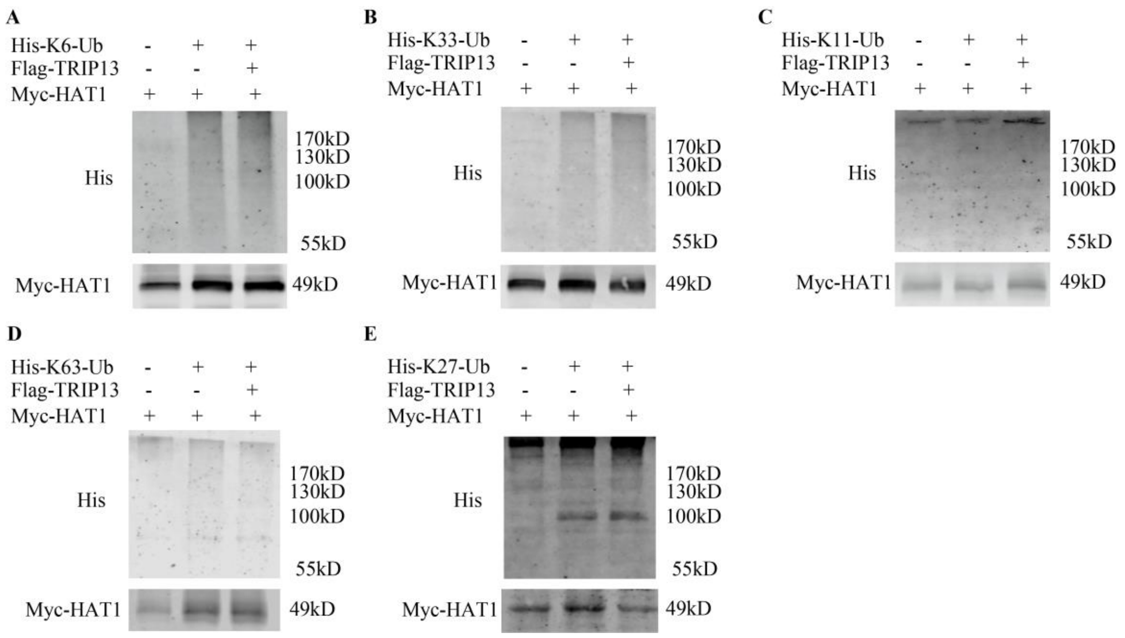

Fig. S7. The effect of overexpressing TRIP13 on the polyubiquitination of HAT1. (A-E) HEK293T cells were transfected with Flag-TRIP13, Myc-HAT1, and His-tagged ubiquitin mutants (K6/K33/K11/K63/K27). Forty-eight hours post-transfection, the cells were harvested, and the effect of overexpressing TRIP13 on the levels of (A) K6-, (B) K33-, (C) K11-, (D) K63-, and (E) K27-linked polyubiquitination of HAT1 was evaluated by immunoprecipitation of Myc-tagged HAT1. Data are presented as means ± SEM and are representative of three separate experiments.

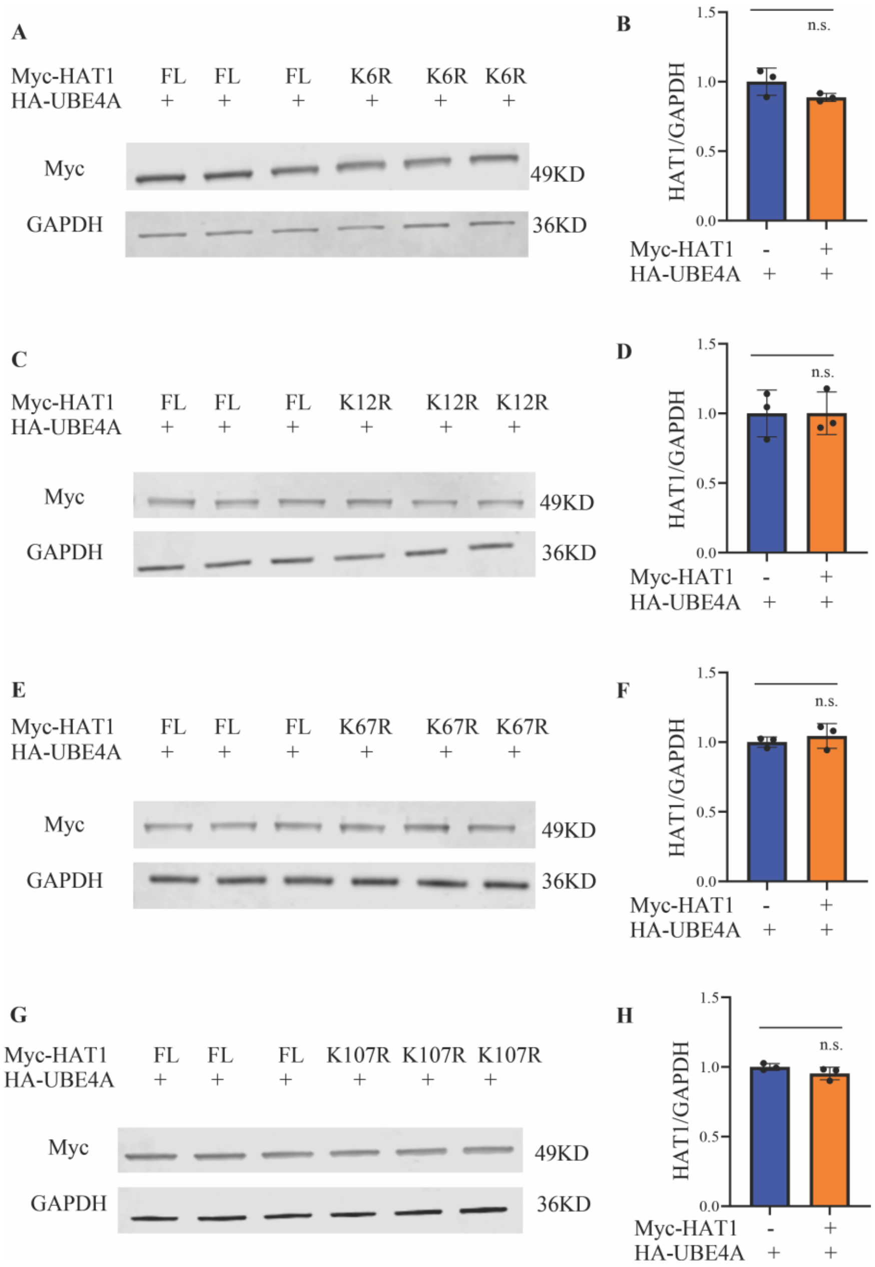

Fig. S8. The specific ubiquitination sites of HAT1 for UBE4A-induced polyubiquitination degradation. (A-H) HEK293T cells were transfected with HA-UBE4A, Myc-HAT1, or Myc-HAT1 mutants (K6R/K12R/K67R/K107R). Forty-eight hours post-transfection, the cells were harvested. WB assays showed that mutating K6 (A and B), K12 (C and D), K67 (E and F), or K107 (G and H) did not affect UBE4A-mediated HAT1 degradation. Data are presented as means ± SEM (n=3 mice) and are representative of three separate experiments. n.s.: no significant differences.

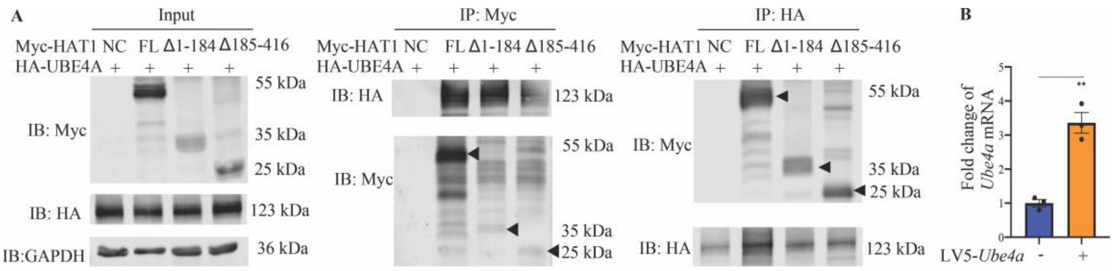

Fig. S9. The identification of the UBE4A binding domains of HAT1 and the validation of UBE4A overexpressing. (**A**) Co-IP assays show the interaction between UBE4A and different truncated mutants of HAT1. **(B)** WT mice were intracolonally administered with TNBS on day 1 and day 8, and then injected with LV5-*Ube4a* or control (i.p.) for 3 days. MACS-sorted $CD4^+$ $CD25^+$ T cells were obtained from mouse lymph nodes and spleen 24 h after last injection. The *Ube4a* mRNA expression was analyzed by qRT-PCR. Data are presented as means ± SEM (n=3 mice) and are representative of three separate experiments. ** P < 0.01.

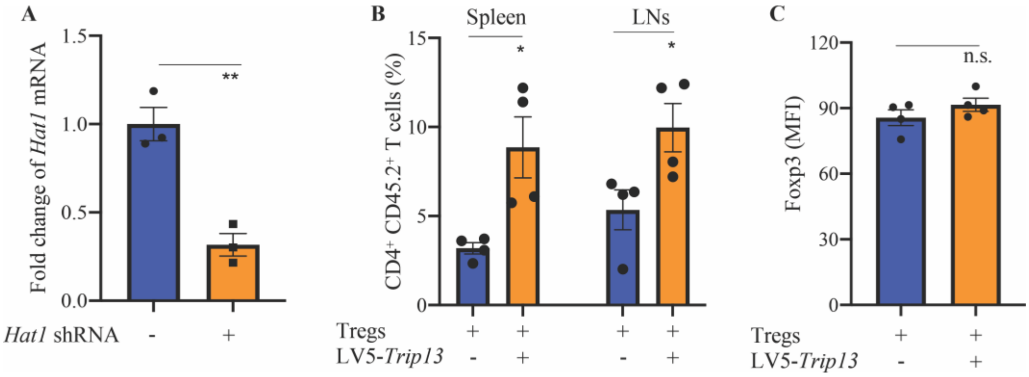

Fig. S10. Validation of the knockdown efficiency of *Hat1* shRNA and the effect of LV-*Trip13* on the proportion of CD4^{+}CD45.2^{+} T cells in lymphoid tissues and Foxp3 expression in CD45.1^{+} cells. (A-C) Naïve CD4^{+} T cells (Teffs, 4×10^{5} cells/mouse) from Ly5.1 B6 mice (CD45.1^{+}) were injected intraperitoneally into *Rag1*$^{-/-}$ mice, either alone or together with CD4^{+}Foxp3/YFP^{+} cells (Tregs, CD45.2^{+}, 2×10^{4} cells/mouse). The mice were treated with LV-*Trip13*, *Hat1* shRNA, or vehicle control (i.p.) once a week, starting on the 2nd day after cell injection, for 5 weeks. Colon, spleen, and lymph nodes were harvested 8 weeks after cell transfer. (A) Knockdown efficiency of *Hat1* shRNA is shown. (B) The proportion of CD45.2^{+} cells in the spleen and mesenteric lymph nodes of *Rag1* KO mice after transfer of Tregs and treatment with LV5-*Trip13*. (C) The expression of Foxp3 in CD4^{+}CD45.1^{+} T cells was determined in recipient *Rag1* KO mice following Treg transfer and LV5-*Trip13* treatment by FCM. Data represents means ± SEM (n=4 mice) and are representative of three separate experiments. * $P < 0.05$, ** $P < 0.01$, n.s.: no significant differences.

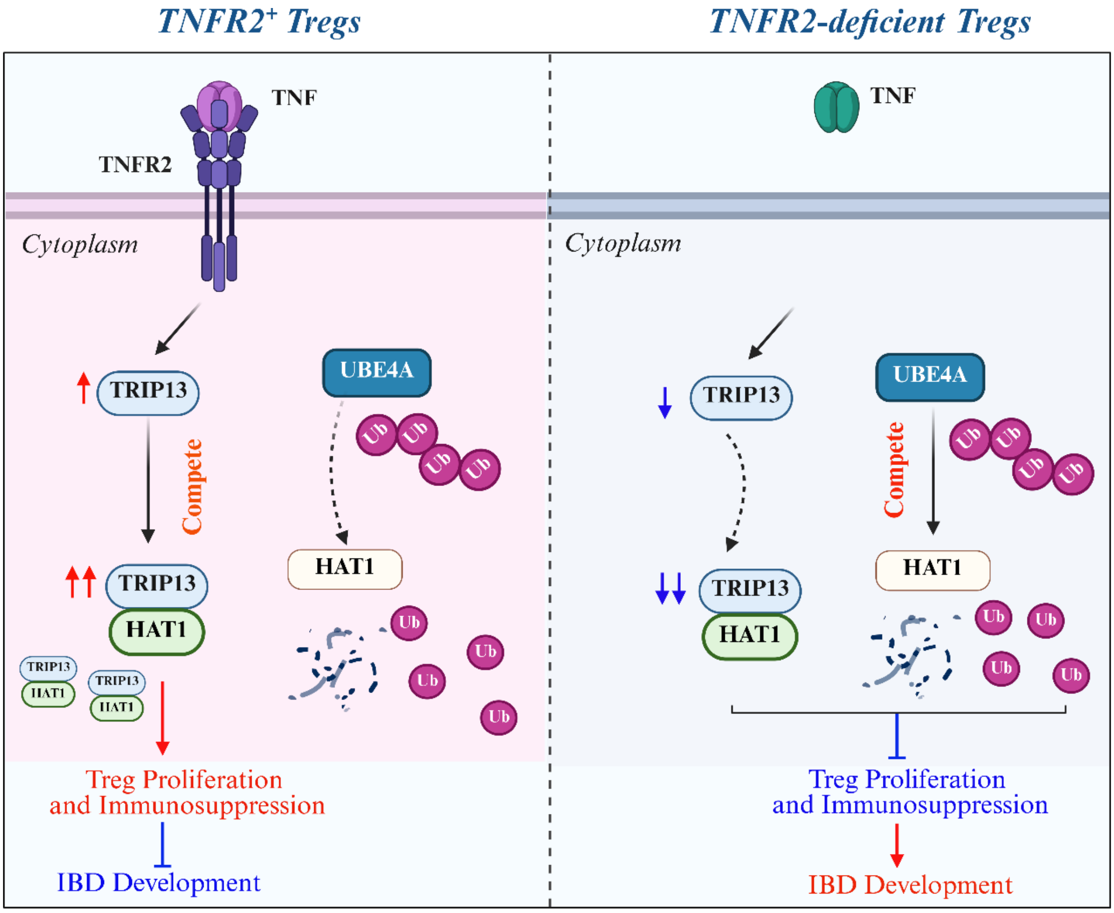

Fig. S11. Schematic model of the TNFR2-TRIP13-HAT1 axis in regulating Treg proliferation and colitis development. TRIP13, a downstream target of the TNF-TNFR2 pathway, inhibits UBE4A-mediated ubiquitination and degradation of HAT1 by directly binding to HAT1, competing with UBE4A, and promoting Treg expansion, which in turn suppresses colitis progression.
