# Supplementary material for: TRIP13 promotes the expansion and immunosuppression of CD4+Foxp3+ regulatory T cells by sustaining HAT1 stability
Source: Cell Death Dis. 2026 Jan 14;17(1):32. doi: 10.1038/s41419-025-08214-7 (PMC12804696; doi:10.1038/s41419-025-08214-7)
Supplement: Supplementary file 2 — Supplemental data set 1 [file 41419_2025_8214_MOESM2_ESM.docx]

| Supplementary data 1. TRIP13-interacting proteins identified by mass spectrometry analysis. | | | | | | | | | | | | | | |
| --- | --- | --- | --- | --- | --- | --- | --- | --- | --- | --- | --- | --- | --- | --- |
| **Accession** | **Protein ID** | **Gene** | **Mw (kDa)** | **Length** | **Spectra count** | | | **Intensity** | | | **iBAQ** | | | **log2 FC** |
|  |  |  |  |  | **Total** | **Trip13** | **IgG** | **Total** | **Trip13** | **IgG** | **Total** | **Trip13** | **IgG** |  |
| Q3TVC7 | CCDB1_MOUSE | Ccndbp1 | 39.356 | 356 | 8 | 8 | 0 | 293800000 | 293800000 | 0 | 18362000 | 18362000 | 0 | 9.951140304 |
| Q9D9J3 | ACTT1_MOUSE | Actrt1 | 42.216 | 376 | 1 | 1 | 0 | 426040000 | 426040000 | 0 | 20287000 | 20287000 | 0 | 9.534040264 |
| Q8BY71 | HAT1_MOUSE | Hat1 | 49.278 | 416 | 72 | 67 | 5 | 5389100000 | 5380600000 | 8571900 | 78103000 | 77979000 | 124230 | 9.293936324 |
| Q8R123 | FAD1_MOUSE | Flad1 | 54.766 | 492 | 1 | 1 | 0 | 231280000 | 231280000 | 0 | 6250800 | 6250800 | 0 | 8.559354846 |
| O55029 | COPB2_MOUSE | Copb2 | 102.449 | 905 | 61 | 50 | 11 | 4834500000 | 4817600000 | 1.7E+07 | 91217000 | 90898000 | 318400 | 8.157283301 |
| Q91ZZ3 | SYUB_MOUSE | Sncb | 14.052 | 133 | 1 | 1 | 0 | 106980000 | 106980000 | 0 | 13372000 | 13372000 | 0 | 8.035288917 |
| Q8CIE6 | COPA_MOUSE | Copa | 138.432 | 1224 | 62 | 55 | 7 | 2007400000 | 1998400000 | 9014800 | 26413000 | 26295000 | 118620 | 7.792334182 |
| P62309 | RUXG_MOUSE | Snrpg | 8.496 | 76 | 3 | 3 | 0 | 91513000 | 91513000 | 0 | 30504000 | 30504000 | 0 | 7.406580332 |
| Q9D7N9 | APMAP_MOUSE | Apmap | 46.434 | 415 | 10 | 9 | 1 | 69707000 | 69707000 | 0 | 2788300 | 2788300 | 0 | 7.147383704 |
| Q5XG73 | ACBD5_MOUSE | Acbd5 | 56.614 | 508 | 8 | 8 | 0 | 118280000 | 118280000 | 0 | 4078600 | 4078600 | 0 | 6.960694057 |
| Q3UA06 | TRIP13_MOUSE | Trip13 | 48.377 | 432 | 35 | 29 | 6 | 1700400000 | 1686800000 | 1.4E+07 | 32701000 | 32438000 | 263280 | 6.94502267 |
| P61924 | COPZ1_MOUSE | Copz1 | 20.198 | 177 | 4 | 3 | 1 | 53048000 | 52492000 | 555910 | 10610000 | 10498000 | 111180 | 6.561102422 |
| Q8R420 | ABCA3_MOUSE | Abca3 | 191.971 | 1704 | 1 | 1 | 0 | 60373000 | 60373000 | 0 | 794380 | 794380 | 0 | 6.488531299 |
| Q9QXK3 | COPG2_MOUSE | Copg2 | 97.681 | 871 | 42 | 34 | 8 | 878490000 | 867930000 | 1.1E+07 | 18302000 | 18082000 | 219990 | 6.360896952 |
| Q5XJY5 | COPD_MOUSE | Arcn1 | 57.229 | 511 | 41 | 33 | 8 | 2012500000 | 1988300000 | 2.4E+07 | 64919000 | 64137000 | 782260 | 6.357406893 |
| Q921M3 | SF3B3_MOUSE | Sf3b3 | 135.55 | 1217 | 13 | 11 | 2 | 90146000 | 88725000 | 1421300 | 1502400 | 1478800 | 23688 | 5.964057662 |
| Q62191 | RO52_MOUSE | Trim21 | 54.175 | 470 | 4 | 4 | 0 | 62712000 | 62712000 | 0 | 2508500 | 2508500 | 0 | 5.899065986 |
| P59708 | SF3B6_MOUSE | Sf3b6 | 14.585 | 125 | 7 | 5 | 2 | 103050000 | 101200000 | 1852500 | 12881000 | 12650000 | 231560 | 5.771591937 |
| O89079 | COPE_MOUSE | Cope | 34.567 | 308 | 25 | 18 | 7 | 2608300000 | 2561300000 | 4.7E+07 | 124200000 | 121970000 | 2237700 | 5.768347963 |
| Q9QZE5 | COPG1_MOUSE | Copg1 | 97.513 | 874 | 67 | 46 | 21 | 2841500000 | 2785600000 | 5.6E+07 | 59197000 | 58033000 | 1163900 | 5.639770478 |
| Q60931 | VDAC3_MOUSE | Vdac3 | 30.753 | 283 | 1 | 1 | 0 | 19350000 | 19350000 | 0 | 1138300 | 1138300 | 0 | 5.452652406 |
| Q8BTU1 | CFA20_MOUSE | Cfap20 | 22.748 | 193 | 3 | 3 | 0 | 26700000 | 26700000 | 0 | 2053800 | 2053800 | 0 | 5.34048977 |
| Q9EP69 | SAC1_MOUSE | Sacm1l | 66.944 | 587 | 31 | 23 | 8 | 501850000 | 489690000 | 1.2E+07 | 13564000 | 13235000 | 328720 | 5.331297717 |
| Q921N7 | TMM70_MOUSE | Tmem70 | 28.275 | 253 | 1 | 1 | 0 | 12774000 | 12774000 | 0 | 912430 | 912430 | 0 | 5.321629109 |
| O70200 | AIF1_MOUSE | Aif1 | 16.911 | 147 | 6 | 5 | 1 | 45163000 | 43967000 | 1195500 | 3763600 | 3663900 | 99629 | 5.200735061 |
| Q8CHP5 | PYM1_MOUSE | Pym1 | 22.69 | 203 | 4 | 2 | 2 | 169170000 | 164440000 | 4732200 | 16917000 | 16444000 | 473220 | 5.118906418 |
| Q5RL79 | KTAP2_MOUSE | Krtcap2 | 14.675 | 136 | 1 | 1 | 0 | 20091000 | 20091000 | 0 | 5022800 | 5022800 | 0 | 5.024280838 |
| Q9JIY5 | HTRA2_MOUSE | Htra2 | 49.348 | 458 | 3 | 3 | 0 | 20445000 | 20445000 | 0 | 851870 | 851870 | 0 | 4.966796303 |
| Q8BIG7 | CMTD1_MOUSE | Comtd1 | 28.961 | 262 | 3 | 2 | 1 | 8176300 | 7909700 | 266580 | 511020 | 494360 | 16662 | 4.890982525 |
| Q9QXN3 | TRIP4_MOUSE | Trip4 | 66.197 | 581 | 1 | 1 | 0 | 13425000 | 13425000 | 0 | 394850 | 394850 | 0 | 4.799734302 |
| Q8BFZ3 | ACTBL_MOUSE | Actbl2 | 42.004 | 376 | 7 | 5 | 2 | 483240000 | 466330000 | 1.7E+07 | 20135000 | 19431000 | 704590 | 4.785402679 |
| Q9JIF7 | COPB_MOUSE | Copb1 | 107.066 | 953 | 135 | 83 | 52 | 1.1578E+10 | 1.1154E+10 | 4.2E+08 | 203120000 | 195680000 | 7443500 | 4.716400692 |
| P35951 | LDLR_MOUSE | Ldlr | 94.947 | 862 | 6 | 5 | 1 | 32861000 | 31599000 | 1261500 | 714370 | 686940 | 27425 | 4.646666791 |
| Q9D2R6 | COA3_MOUSE | Coa3 | 11.987 | 108 | 2 | 2 | 0 | 21334000 | 21334000 | 0 | 3555700 | 3555700 | 0 | 4.625935544 |
| Q91YR1 | TWF1_MOUSE | Twf1 | 40.079 | 350 | 2 | 2 | 0 | 13886000 | 13886000 | 0 | 694290 | 694290 | 0 | 4.544153138 |
| Q9DC28 | KC1D_MOUSE | Csnk1d | 47.316 | 415 | 12 | 8 | 4 | 109320000 | 104730000 | 4585200 | 4752900 | 4553500 | 199360 | 4.513546289 |
| Q9DAV9 | TM38B_MOUSE | Tmem38b | 32.64 | 292 | 10 | 6 | 4 | 275430000 | 263700000 | 1.2E+07 | 16202000 | 15512000 | 690240 | 4.490130769 |
| Q9D023 | MPC2_MOUSE | Mpc2 | 14.286 | 127 | 2 | 2 | 0 | 11456000 | 11456000 | 0 | 1272900 | 1272900 | 0 | 4.453644712 |
| Q8C147 | DOCK8_MOUSE | Dock8 | 238.978 | 2100 | 3 | 2 | 1 | 9960200 | 9523600 | 436670 | 84409 | 80708 | 3700.6 | 4.446891703 |
| P62305 | RUXE_MOUSE | Snrpe | 10.804 | 92 | 5 | 4 | 1 | 131340000 | 125490000 | 5855100 | 32836000 | 31372000 | 1463800 | 4.421734783 |
| O08547 | SC22B_MOUSE | Sec22b | 24.741 | 215 | 7 | 5 | 2 | 57685000 | 55035000 | 2649600 | 4120400 | 3931100 | 189260 | 4.376502922 |
| Q6WKZ8 | UBR2_MOUSE | Ubr2 | 199.155 | 1755 | 3 | 3 | 0 | 13209000 | 13209000 | 0 | 140520 | 140520 | 0 | 4.343149929 |
| P11103 | PARP1_MOUSE | Parp1 | 113.1 | 1013 | 9 | 7 | 2 | 74415000 | 70836000 | 3579300 | 1219900 | 1161200 | 58677 | 4.306733374 |
| Q99PV0 | PRP8_MOUSE | Prpf8 | 273.616 | 2335 | 5 | 4 | 1 | 11375000 | 10814000 | 560070 | 76855 | 73071 | 3784.3 | 4.271149299 |
| Q6P4T2 | U520_MOUSE | Snrnp200 | 244.547 | 2136 | 8 | 8 | 0 | 25720000 | 25720000 | 0 | 199380 | 199380 | 0 | 4.243265316 |
| Q9JHJ3 | GLMP_MOUSE | Glmp | 43.804 | 404 | 1 | 1 | 0 | 10068000 | 10068000 | 0 | 774490 | 774490 | 0 | 4.203273788 |
| P28667 | MRP_MOUSE | Marcksl1 | 20.165 | 200 | 1 | 1 | 0 | 15797000 | 15797000 | 0 | 1974600 | 1974600 | 0 | 4.147894941 |
| P29391 | FRIL1_MOUSE | Ftl1 | 20.802 | 183 | 13 | 10 | 3 | 314290000 | 296840000 | 1.7E+07 | 22449000 | 21203000 | 1246000 | 4.088882712 |
| Q9DAJ4 | WDR83_MOUSE | Wdr83 | 34.444 | 315 | 1 | 1 | 0 | 12292000 | 12292000 | 0 | 723040 | 723040 | 0 | 4.051223743 |
| Q3UKJ7 | SMU1_MOUSE | Smu1 | 57.544 | 513 | 14 | 9 | 5 | 79266000 | 74659000 | 4607200 | 2477100 | 2333100 | 143970 | 4.018354053 |
| P04441 | HG2A_MOUSE | Cd74 | 31.557 | 279 | 1 | 1 | 0 | 9339800 | 9339800 | 0 | 583740 | 583740 | 0 | 4.010950172 |
| Q99NB9 | SF3B1_MOUSE | Sf3b1 | 145.816 | 1304 | 67 | 47 | 20 | 1406200000 | 1321300000 | 8.5E+07 | 19263000 | 18100000 | 1163100 | 3.959947749 |
| Q99JN2 | KLH22_MOUSE | Klhl22 | 71.687 | 634 | 1 | 1 | 0 | 7108200 | 7108200 | 0 | 187060 | 187060 | 0 | 3.939299706 |
| Q9CQ75 | NDUA2_MOUSE | Ndufa2 | 10.916 | 99 | 1 | 1 | 0 | 11187000 | 11187000 | 0 | 1243000 | 1243000 | 0 | 3.924071498 |
| Q8K4Z5 | SF3A1_MOUSE | Sf3a1 | 88.545 | 791 | 35 | 22 | 13 | 669650000 | 628050000 | 4.2E+07 | 14558000 | 13653000 | 904370 | 3.916189306 |
| Q9ERG0 | LIMA1_MOUSE | Lima1 | 84.06 | 753 | 15 | 10 | 5 | 179970000 | 168740000 | 1.1E+07 | 3528900 | 3308700 | 220190 | 3.909372174 |
| Q9QYJ0 | DNJA2_MOUSE | Dnaja2 | 45.746 | 412 | 17 | 11 | 6 | 288610000 | 270590000 | 1.8E+07 | 11545000 | 10823000 | 721150 | 3.90771724 |
| Q9Z126 | PLF4_MOUSE | Pf4 | 11.243 | 105 | 2 | 2 | 0 | 10035000 | 10035000 | 0 | 2007000 | 2007000 | 0 | 3.905905693 |
| Q9Z0R9 | FADS2_MOUSE | Fads2 | 52.387 | 444 | 7 | 5 | 2 | 47893000 | 44782000 | 3111000 | 1995500 | 1865900 | 129620 | 3.847468662 |
| Q8VDD5 | MYH9_MOUSE | Myh9 | 226.372 | 1960 | 165 | 104 | 61 | 4990400000 | 4666200000 | 3.2E+08 | 38685000 | 36172000 | 2513400 | 3.847158654 |
| A2AN08 | UBR4_MOUSE | Ubr4 | 572.29 | 5180 | 3 | 2 | 1 | 7552500 | 7061800 | 490770 | 28608 | 26749 | 1859 | 3.846916995 |
| Q6IRU2 | TPM4_MOUSE | Tpm4 | 28.468 | 248 | 2 | 1 | 1 | 9011500 | 8418300 | 593170 | 450570 | 420920 | 29658 | 3.827011384 |
| Q9WV32 | ARC1B_MOUSE | Arpc1b | 41.064 | 372 | 8 | 5 | 3 | 63578000 | 59387000 | 4190800 | 3346200 | 3125600 | 220570 | 3.824849577 |
| Q91VN6 | DDX41_MOUSE | Ddx41 | 69.82 | 622 | 54 | 35 | 19 | 888430000 | 829480000 | 5.9E+07 | 18903000 | 17648000 | 1254300 | 3.814619005 |
| O08573 | LEG9_MOUSE | Lgals9 | 40.036 | 353 | 12 | 8 | 4 | 147760000 | 137620000 | 1E+07 | 9235000 | 8601000 | 633940 | 3.762133822 |
| Q5SUA5 | MYO1G_MOUSE | Myo1g | 117.227 | 1024 | 7 | 5 | 2 | 28131000 | 26124000 | 2007000 | 432790 | 401910 | 30877 | 3.702263292 |
| Q9D7M1 | GID8_MOUSE | Gid8 | 26.779 | 228 | 4 | 3 | 1 | 23504000 | 21782000 | 1722600 | 1567000 | 1452100 | 114840 | 3.660476785 |
| P63037 | DNJA1_MOUSE | Dnaja1 | 44.868 | 397 | 14 | 9 | 5 | 100150000 | 92803000 | 7349600 | 4173000 | 3866800 | 306230 | 3.658433805 |
| Q8BHC4 | DCAKD_MOUSE | Dcakd | 26.476 | 231 | 3 | 2 | 1 | 7866600 | 7280100 | 586480 | 491660 | 455010 | 36655 | 3.633804452 |
| Q8BQ30 | PPR18_MOUSE | Ppp1r18 | 65.63 | 594 | 14 | 10 | 4 | 158500000 | 146340000 | 1.2E+07 | 4402600 | 4064900 | 337700 | 3.589465002 |
| P46062 | SIPA1_MOUSE | Sipa1 | 112.066 | 1037 | 32 | 20 | 12 | 342870000 | 316430000 | 2.6E+07 | 5911600 | 5455700 | 455880 | 3.581037739 |
| Q80TP3 | UBR5_MOUSE | Ubr5 | 308.352 | 2792 | 6 | 4 | 2 | 26494000 | 24447000 | 2046900 | 176630 | 162980 | 13646 | 3.578144909 |
| Q6P1F6 | 2ABA_MOUSE | Ppp2r2a | 51.692 | 447 | 3 | 2 | 1 | 4826300 | 4450600 | 375750 | 172370 | 158950 | 13420 | 3.566154834 |
| Q9DAW6 | PRP4_MOUSE | Prpf4 | 58.37 | 521 | 2 | 2 | 0 | 9459200 | 9459200 | 0 | 315310 | 315310 | 0 | 3.564341995 |
| Q9WTI7 | MYO1C_MOUSE | Myo1c | 121.944 | 1063 | 75 | 44 | 31 | 1526600000 | 1406200000 | 1.2E+08 | 23131000 | 21306000 | 1824100 | 3.546014333 |
| P11881 | ITPR1_MOUSE | Itpr1 | 313.167 | 2749 | 3 | 2 | 1 | 4026500 | 3706800 | 319730 | 25324 | 23313 | 2010.9 | 3.535248254 |
| B7ZMP1 | XPP3_MOUSE | Xpnpep3 | 56.677 | 506 | 4 | 3 | 1 | 16917000 | 15571000 | 1345300 | 545700 | 502300 | 43396 | 3.532861767 |
| Q9CVB6 | ARPC2_MOUSE | Arpc2 | 34.357 | 300 | 20 | 14 | 6 | 145900000 | 134230000 | 1.2E+07 | 6343400 | 5836300 | 507070 | 3.524696309 |
| O88554 | PARP2_MOUSE | Parp2 | 63.397 | 559 | 3 | 2 | 1 | 7765700 | 7144600 | 621090 | 215710 | 198460 | 17253 | 3.523978998 |
| O35226 | PSMD4_MOUSE | Psmd4 | 40.704 | 376 | 2 | 2 | 0 | 6322800 | 6322800 | 0 | 421520 | 421520 | 0 | 3.48193662 |
| P49710 | HCLS1_MOUSE | Hcls1 | 54.24 | 486 | 12 | 9 | 3 | 161380000 | 148050000 | 1.3E+07 | 8069100 | 7402600 | 666480 | 3.473335805 |
| Q9WV84 | NDKM_MOUSE | Nme4 | 20.549 | 186 | 1 | 1 | 0 | 7336200 | 7336200 | 0 | 564330 | 564330 | 0 | 3.462169059 |
| P51863 | VA0D1_MOUSE | Atp6v0d1 | 40.301 | 351 | 3 | 2 | 1 | 9257800 | 8479400 | 778470 | 544580 | 498790 | 45792 | 3.445248836 |
| Q6URW6 | MYH14_MOUSE | Myh14 | 228.586 | 2000 | 4 | 2 | 2 | 153510000 | 140600000 | 1.3E+07 | 1199300 | 1098400 | 100900 | 3.444477046 |
| Q8BHI7 | ELOV5_MOUSE | Elovl5 | 35.332 | 299 | 1 | 1 | 0 | 5948000 | 5948000 | 0 | 540730 | 540730 | 0 | 3.423143367 |
| Q3U4G3 | XXLT1_MOUSE | Xxylt1 | 43.839 | 392 | 1 | 1 | 0 | 10703000 | 10703000 | 0 | 445960 | 445960 | 0 | 3.378060801 |
| P58501 | PAXB1_MOUSE | Paxbp1 | 104.836 | 919 | 29 | 18 | 11 | 285400000 | 259790000 | 2.6E+07 | 5824500 | 5301900 | 522650 | 3.342566741 |
| Q8R2Y8 | PTH2_MOUSE | Ptrh2 | 19.527 | 181 | 3 | 2 | 1 | 20525000 | 18683000 | 1842700 | 1865900 | 1698400 | 167520 | 3.341833015 |
| Q923D4 | SF3B5_MOUSE | Sf3b5 | 10.119 | 86 | 6 | 4 | 2 | 249360000 | 226700000 | 2.3E+07 | 41559000 | 37784000 | 3775700 | 3.322946678 |
| O70310 | NMT1_MOUSE | Nmt1 | 56.888 | 496 | 2 | 1 | 1 | 3915000 | 3558600 | 356390 | 145000 | 131800 | 13200 | 3.319781016 |
| Q5SYD0 | MYO1D_MOUSE | Myo1d | 116.081 | 1006 | 61 | 36 | 25 | 808200000 | 734320000 | 7.4E+07 | 10922000 | 9923200 | 998370 | 3.313153122 |
| Q8K2C9 | HACD3_MOUSE | Hacd3 | 43.131 | 362 | 1 | 1 | 0 | 6729300 | 6729300 | 0 | 373850 | 373850 | 0 | 3.306740183 |
| Q9JHS4 | CLPX_MOUSE | Clpx | 69.229 | 634 | 3 | 2 | 1 | 9502500 | 8630100 | 872400 | 211170 | 191780 | 19387 | 3.306315601 |
| P14148 | RL7_MOUSE | Rpl7 | 31.42 | 270 | 14 | 9 | 5 | 230960000 | 209480000 | 2.1E+07 | 12831000 | 11638000 | 1193400 | 3.285679448 |
| O08810 | U5S1_MOUSE | Eftud2 | 109.361 | 971 | 12 | 8 | 4 | 49859000 | 45184000 | 4674900 | 923310 | 836740 | 86571 | 3.272804584 |
| Q8BK63 | KC1A_MOUSE | Csnk1a1 | 38.915 | 337 | 17 | 11 | 6 | 206180000 | 186720000 | 1.9E+07 | 9818100 | 8891400 | 926720 | 3.262218716 |
| P20060 | HEXB_MOUSE | Hexb | 61.116 | 536 | 4 | 3 | 1 | 13462000 | 12141000 | 1320700 | 448740 | 404720 | 44023 | 3.200512557 |
| P10922 | H10_MOUSE | H1-0 | 20.861 | 194 | 3 | 2 | 1 | 6247000 | 6247000 | 0 | 892430 | 892430 | 0 | 3.200470431 |
| Q3URK1 | CC190_MOUSE | Ccdc190 | 32.592 | 288 | 2 | 1 | 1 | 15723000 | 14163000 | 1560100 | 748710 | 674420 | 74293 | 3.182416477 |
| Q9ERA6 | TFP11_MOUSE | Tfip11 | 96.305 | 838 | 27 | 15 | 12 | 420960000 | 379070000 | 4.2E+07 | 7015900 | 6317900 | 698070 | 3.177993151 |
| Q9EQK5 | MVP_MOUSE | Mvp | 95.924 | 861 | 12 | 7 | 5 | 62456000 | 56192000 | 6264900 | 1224600 | 1101800 | 122840 | 3.165001363 |
| B2RQC6 | PYR1_MOUSE | Cad | 243.238 | 2225 | 8 | 5 | 3 | 25021000 | 22509000 | 2512400 | 208510 | 187570 | 20937 | 3.163363885 |
| Q9CR26 | VTA1_MOUSE | Vta1 | 33.913 | 309 | 2 | 1 | 1 | 12157000 | 10929000 | 1227800 | 935120 | 840670 | 94449 | 3.154013921 |
| Q02614 | S30BP_MOUSE | Sap30bp | 33.832 | 308 | 9 | 6 | 3 | 70677000 | 63483000 | 7194400 | 4417300 | 3967700 | 449650 | 3.141424028 |
| Q9CQ69 | QCR8_MOUSE | Uqcrq | 9.768 | 82 | 5 | 3 | 2 | 19349000 | 17373000 | 1976500 | 3224900 | 2895400 | 329420 | 3.135827042 |
| Q6ZQ58 | LARP1_MOUSE | Larp1 | 121.125 | 1072 | 5 | 3 | 2 | 9958300 | 8935500 | 1022800 | 153200 | 137470 | 15735 | 3.127024394 |
| P84089 | ERH_MOUSE | Erh | 12.259 | 104 | 7 | 4 | 3 | 165430000 | 148200000 | 1.7E+07 | 27572000 | 24701000 | 2871400 | 3.104634575 |
| P19973 | LSP1_MOUSE | Lsp1 | 36.714 | 330 | 17 | 10 | 7 | 249590000 | 223570000 | 2.6E+07 | 11345000 | 10162000 | 1182800 | 3.102922857 |
| Q8QZY9 | SF3B4_MOUSE | Sf3b4 | 44.356 | 424 | 7 | 4 | 3 | 188090000 | 168410000 | 2E+07 | 9899400 | 8863900 | 1035500 | 3.097542266 |
| P13020 | GELS_MOUSE | Gsn | 85.942 | 780 | 4 | 3 | 1 | 186140000 | 166440000 | 2E+07 | 5030800 | 4498300 | 532520 | 3.078514976 |
| Q8BFZ9 | ERLN2_MOUSE | Erlin2 | 37.873 | 340 | 1 | 1 | 0 | 6998300 | 6998300 | 0 | 291590 | 291590 | 0 | 3.068769792 |
| Q8BTM8 | FLNA_MOUSE | Flna | 281.222 | 2647 | 84 | 52 | 32 | 929710000 | 830260000 | 9.9E+07 | 6198100 | 5535100 | 662970 | 3.061577946 |
| Q5SW75 | SSH2_MOUSE | Ssh2 | 158.23 | 1423 | 2 | 1 | 1 | 5947400 | 5300400 | 647060 | 75284 | 67093 | 8190.6 | 3.034129838 |
| P62307 | RUXF_MOUSE | Snrpf | 9.725 | 86 | 5 | 3 | 2 | 110150000 | 97917000 | 1.2E+07 | 22030000 | 19583000 | 2446900 | 3.000663176 |
| Q99104 | MYO5A_MOUSE | Myo5a | 215.538 | 1853 | 78 | 46 | 32 | 780800000 | 693470000 | 8.7E+07 | 6789500 | 6030200 | 759360 | 2.989350308 |
| Q9CR46 | SKA2_MOUSE | Ska2 | 13.725 | 120 | 1 | 1 | 0 | 5395700 | 5395700 | 0 | 415060 | 415060 | 0 | 2.98189237 |
| Q9CPP6 | NDUA5_MOUSE | Ndufa5 | 13.36 | 116 | 5 | 3 | 2 | 41078000 | 36444000 | 4634900 | 4564300 | 4049300 | 514990 | 2.975071197 |
| Q9D554 | SF3A3_MOUSE | Sf3a3 | 58.842 | 501 | 22 | 12 | 10 | 407790000 | 361750000 | 4.6E+07 | 14564000 | 12919000 | 1644400 | 2.973970608 |
| P63166 | SUMO1_MOUSE | Sumo1 | 11.557 | 101 | 2 | 1 | 1 | 5227700 | 5227700 | 0 | 522770 | 522770 | 0 | 2.9200443 |
| P21107 | TPM3_MOUSE | Tpm3 | 32.994 | 285 | 3 | 2 | 1 | 20969000 | 18511000 | 2458000 | 873700 | 771290 | 102410 | 2.912826014 |
| Q6R891 | NEB2_MOUSE | Ppp1r9b | 89.52 | 817 | 17 | 10 | 7 | 127580000 | 112590000 | 1.5E+07 | 3357300 | 2962800 | 394440 | 2.909102655 |
| Q9CPQ3 | TOM22_MOUSE | Tomm22 | 15.537 | 142 | 5 | 3 | 2 | 20139000 | 17770000 | 2369900 | 2877100 | 2538500 | 338550 | 2.906545592 |
| Q9D6U8 | F162A_MOUSE | Fam162a | 17.725 | 155 | 5 | 3 | 2 | 54880000 | 48411000 | 6469200 | 4221500 | 3723900 | 497630 | 2.903675675 |
| O54962 | BAF_MOUSE | Banf1 | 10.103 | 89 | 3 | 2 | 1 | 35490000 | 31270000 | 4219300 | 4436200 | 3908800 | 527420 | 2.889703645 |
| Q920L1 | FADS1_MOUSE | Fads1 | 52.323 | 447 | 1 | 1 | 0 | 3694300 | 3694300 | 0 | 184710 | 184710 | 0 | 2.884222131 |
| P62342 | SELT_MOUSE | Selenot | 22.292 | 195 | 4 | 3 | 1 | 36653000 | 32268000 | 4385000 | 3332100 | 2933500 | 398640 | 2.879455413 |
| O89112 | LANC1_MOUSE | Lancl1 | 45.341 | 399 | 1 | 1 | 0 | 4124900 | 4124900 | 0 | 196420 | 196420 | 0 | 2.879265104 |
| P62320 | SMD3_MOUSE | Snrpd3 | 13.916 | 126 | 13 | 7 | 6 | 807690000 | 710960000 | 9.7E+07 | 89743000 | 78996000 | 1.1E+07 | 2.877837496 |
| O88384 | VTI1B_MOUSE | Vti1b | 26.713 | 232 | 3 | 2 | 1 | 5572600 | 4897300 | 675300 | 398040 | 349810 | 48236 | 2.858386113 |
| Q91WJ8 | FUBP1_MOUSE | Fubp1 | 68.54 | 651 | 2 | 1 | 1 | 25284000 | 22205000 | 3078700 | 665370 | 584350 | 81020 | 2.850491374 |
| P57784 | RU2A_MOUSE | Snrpa1 | 28.357 | 255 | 16 | 8 | 8 | 376490000 | 329990000 | 4.6E+07 | 26892000 | 23571000 | 3321300 | 2.827181737 |
| P58058 | NADK_MOUSE | Nadk | 48.597 | 439 | 19 | 12 | 7 | 401500000 | 351730000 | 5E+07 | 28679000 | 25124000 | 3555300 | 2.820975171 |
| Q9WUU7 | CATZ_MOUSE | Ctsz | 33.996 | 306 | 5 | 3 | 2 | 43881000 | 38349000 | 5532500 | 3134400 | 2739200 | 395180 | 2.793185507 |
| Q9DC63 | FBX3_MOUSE | Fbxo3 | 55.227 | 480 | 3 | 2 | 1 | 11104000 | 9703600 | 1400600 | 555210 | 485180 | 70028 | 2.792475089 |
| P18760 | COF1_MOUSE | Cfl1 | 18.56 | 166 | 7 | 4 | 3 | 110110000 | 96118000 | 1.4E+07 | 9175700 | 8009900 | 1165900 | 2.780410667 |
| Q8R2R6 | MTG1_MOUSE | Mtg1 | 36.679 | 326 | 9 | 6 | 3 | 36463000 | 31824000 | 4638800 | 1657400 | 1446500 | 210850 | 2.77829163 |
| Q78JE5 | FBX22_MOUSE | Fbxo22 | 44.203 | 402 | 14 | 8 | 6 | 211490000 | 184510000 | 2.7E+07 | 8459600 | 7380300 | 1079300 | 2.773629814 |
| Q91WD5 | NDUS2_MOUSE | Ndufs2 | 52.626 | 463 | 7 | 4 | 3 | 98912000 | 86193000 | 1.3E+07 | 3410700 | 2972200 | 438570 | 2.760585462 |
| P25206 | MCM3_MOUSE | Mcm3 | 91.546 | 812 | 10 | 6 | 4 | 48811000 | 42533000 | 6277900 | 920950 | 802500 | 118450 | 2.760228663 |
| Q6NZB0 | DNJC8_MOUSE | Dnajc8 | 29.813 | 253 | 1 | 1 | 0 | 7336200 | 7336200 | 0 | 524020 | 524020 | 0 | 2.759487192 |
| P62315 | SMD1_MOUSE | Snrpd1 | 13.282 | 119 | 16 | 9 | 7 | 224740000 | 195600000 | 2.9E+07 | 44948000 | 39121000 | 5827600 | 2.746932609 |
| P58771 | TPM1_MOUSE | Tpm1 | 32.681 | 284 | 7 | 4 | 3 | 99466000 | 86533000 | 1.3E+07 | 4324600 | 3762300 | 562320 | 2.742193452 |
| Q9D8Y0 | EFHD2_MOUSE | Efhd2 | 26.791 | 240 | 24 | 15 | 9 | 1124200000 | 976880000 | 1.5E+08 | 70261000 | 61055000 | 9206300 | 2.729423922 |
| Q6NV83 | SR140_MOUSE | U2surp | 118.261 | 1029 | 3 | 2 | 1 | 6794400 | 5892300 | 902140 | 141550 | 122760 | 18795 | 2.707407643 |
| Q91VI7 | RINI_MOUSE | Rnh1 | 49.816 | 456 | 20 | 12 | 8 | 126090000 | 109260000 | 1.7E+07 | 4670200 | 4046800 | 623320 | 2.698658246 |
| Q9JJZ4 | UB2J1_MOUSE | Ube2j1 | 34.99 | 318 | 4 | 2 | 2 | 2552200 | 2210900 | 341270 | 150130 | 130050 | 20074 | 2.695648271 |
| E9Q634 | MYO1E_MOUSE | Myo1e | 126.818 | 1107 | 53 | 30 | 23 | 840120000 | 727390000 | 1.1E+08 | 12539000 | 10857000 | 1682600 | 2.689857592 |
| Q9JIX0 | ENY2_MOUSE | Eny2 | 11.529 | 101 | 7 | 4 | 3 | 37909000 | 32819000 | 5089500 | 6318100 | 5469900 | 848250 | 2.688935444 |
| Q9D1L9 | LTOR5_MOUSE | Lamtor5 | 9.642 | 91 | 2 | 1 | 1 | 14128000 | 12226000 | 1901800 | 2825500 | 2445100 | 380360 | 2.684515032 |
| Q60605 | MYL6_MOUSE | Myl6 | 16.93 | 151 | 22 | 13 | 9 | 3615400000 | 3128500000 | 4.9E+08 | 328670000 | 284410000 | 4.4E+07 | 2.683803329 |
| Q9WUM4 | COR1C_MOUSE | Coro1c | 53.121 | 474 | 7 | 4 | 3 | 64780000 | 56025000 | 8755400 | 2699200 | 2334400 | 364810 | 2.677825747 |
| Q91WS0 | CISD1_MOUSE | Cisd1 | 12.097 | 108 | 3 | 2 | 1 | 20285000 | 17541000 | 2744200 | 3380800 | 2923400 | 457360 | 2.676273462 |
| P83870 | PHF5A_MOUSE | Phf5a | 12.405 | 110 | 6 | 3 | 3 | 92163000 | 79655000 | 1.3E+07 | 10240000 | 8850600 | 1389700 | 2.670913799 |
| P19096 | FAS_MOUSE | Fasn | 272.428 | 2504 | 3 | 2 | 1 | 4405400 | 3799000 | 606470 | 32393 | 27934 | 4459.4 | 2.647111525 |
| Q60737 | CSK21_MOUSE | Csnk2a1 | 45.134 | 391 | 8 | 5 | 3 | 58618000 | 50541000 | 8076000 | 2930900 | 2527100 | 403800 | 2.645741394 |
| Q9D2P4 | URM1_MOUSE | Urm1 | 11.323 | 101 | 3 | 2 | 1 | 43765000 | 37682000 | 6083400 | 8753000 | 7536300 | 1216700 | 2.630925766 |
| Q9CQI7 | RU2B_MOUSE | Snrpb2 | 25.323 | 225 | 6 | 4 | 2 | 98830000 | 85077000 | 1.4E+07 | 8984600 | 7734300 | 1250300 | 2.629022808 |
| P47962 | RL5_MOUSE | Rpl5 | 34.401 | 297 | 20 | 12 | 8 | 264290000 | 227500000 | 3.7E+07 | 15546000 | 13382000 | 2164100 | 2.628520179 |
| Q9JMH9 | MY18A_MOUSE | Myo18a | 232.755 | 2050 | 1 | 1 | 0 | 3138600 | 3138600 | 0 | 22580 | 22580 | 0 | 2.610472198 |
| P17710 | HXK1_MOUSE | Hk1 | 108.303 | 974 | 7 | 5 | 2 | 31236000 | 26830000 | 4405600 | 548000 | 470710 | 77292 | 2.606436636 |
| Q8JZQ9 | EIF3B_MOUSE | Eif3b | 91.37 | 803 | 7 | 4 | 3 | 33437000 | 28658000 | 4779400 | 835930 | 716440 | 119490 | 2.584036509 |
| Q6ZWX6 | IF2A_MOUSE | Eif2s1 | 36.108 | 315 | 3 | 1 | 2 | 5287100 | 4531000 | 756090 | 240320 | 205950 | 34368 | 2.583199612 |
| Q91VR5 | DDX1_MOUSE | Ddx1 | 82.5 | 740 | 11 | 6 | 5 | 97321000 | 83362000 | 1.4E+07 | 2115700 | 1812200 | 303460 | 2.578194298 |
| O35701 | MATN3_MOUSE | Matn3 | 51.845 | 481 | 2 | 1 | 1 | 9736200 | 8338900 | 1397300 | 347720 | 297820 | 49904 | 2.577215287 |
| P48771 | CX7A2_MOUSE | Cox7a2 | 9.291 | 83 | 3 | 2 | 1 | 18900000 | 16183000 | 2717100 | 3780000 | 3236600 | 543420 | 2.57433951 |
| Q62203 | SF3A2_MOUSE | Sf3a2 | 49.911 | 475 | 10 | 6 | 4 | 130150000 | 111410000 | 1.9E+07 | 8134600 | 6963300 | 1171300 | 2.571608892 |
| P19253 | RL13A_MOUSE | Rpl13a | 23.464 | 203 | 7 | 5 | 2 | 41220000 | 35217000 | 6003400 | 2424700 | 2071600 | 353140 | 2.552420315 |
| P35279 | RAB6A_MOUSE | Rab6a | 23.59 | 208 | 3 | 2 | 1 | 7035500 | 6005700 | 1029800 | 413860 | 353280 | 60579 | 2.543968235 |
| Q9D8B3 | CHM4B_MOUSE | Chmp4b | 24.936 | 224 | 3 | 2 | 1 | 33587000 | 28657000 | 4929300 | 2099200 | 1791100 | 308080 | 2.539432895 |
| Q9CZM2 | RL15_MOUSE | Rpl15 | 24.146 | 204 | 10 | 5 | 5 | 167260000 | 142530000 | 2.5E+07 | 11947000 | 10181000 | 1766300 | 2.527048148 |
| Q6ZPF4 | FMNL3_MOUSE | Fmnl3 | 117.169 | 1028 | 1 | 1 | 0 | 3036100 | 3036100 | 0 | 46710 | 46710 | 0 | 2.509057711 |
| Q9CWU6 | UQCC1_MOUSE | Uqcc1 | 34.3 | 295 | 2 | 1 | 1 | 15400000 | 13081000 | 2319000 | 855550 | 726720 | 128840 | 2.495898109 |
| Q9R0P6 | SC11A_MOUSE | Sec11a | 20.626 | 179 | 3 | 2 | 1 | 10506000 | 8923500 | 1582000 | 875460 | 743630 | 131830 | 2.495860079 |
| Q9CQS8 | SC61B_MOUSE | Sec61b | 9.958 | 96 | 4 | 3 | 1 | 379130000 | 322010000 | 5.7E+07 | 63188000 | 53669000 | 9519500 | 2.495113381 |
| Q8VBZ3 | CLPT1_MOUSE | Clptm1 | 75.291 | 664 | 3 | 2 | 1 | 11669000 | 9892100 | 1776600 | 432170 | 366370 | 65799 | 2.477157928 |
| Q9DAS9 | GBG12_MOUSE | Gng12 | 7.997 | 72 | 2 | 2 | 0 | 7085600 | 7085600 | 0 | 1417100 | 1417100 | 0 | 2.463255923 |
| Q8BMG7 | RBGPR_MOUSE | Rab3gap2 | 152.535 | 1366 | 2 | 1 | 1 | 6774700 | 5731600 | 1043100 | 98184 | 83067 | 15117 | 2.458060457 |
| O70579 | PM34_MOUSE | Slc25a17 | 34.413 | 307 | 2 | 1 | 1 | 7111200 | 5984100 | 1127100 | 474080 | 398940 | 75141 | 2.408518762 |
| Q9Z0P5 | TWF2_MOUSE | Twf2 | 39.471 | 349 | 6 | 3 | 3 | 24128000 | 20273000 | 3854700 | 1096700 | 921520 | 175210 | 2.394869106 |
| Q9D0Q7 | RM45_MOUSE | Mrpl45 | 35.411 | 306 | 2 | 1 | 1 | 4047000 | 3389000 | 658020 | 175960 | 147350 | 28609 | 2.364656298 |
| P39098 | MA1A2_MOUSE | Man1a2 | 72.871 | 641 | 7 | 4 | 3 | 27411000 | 22889000 | 4522500 | 783170 | 653960 | 129210 | 2.339462026 |
| Q99K48 | NONO_MOUSE | Nono | 54.541 | 473 | 3 | 2 | 1 | 16442000 | 13723000 | 2718700 | 632380 | 527820 | 104560 | 2.335607037 |
| Q9QZD8 | DIC_MOUSE | Slc25a10 | 31.715 | 287 | 3 | 2 | 1 | 16031000 | 13369000 | 2662900 | 943030 | 786390 | 156640 | 2.327821399 |
| Q9DC23 | DJC10_MOUSE | Dnajc10 | 90.583 | 793 | 30 | 17 | 13 | 493980000 | 411190000 | 8.3E+07 | 12666000 | 10543000 | 2122800 | 2.312294181 |
| P61804 | DAD1_MOUSE | Dad1 | 12.497 | 113 | 2 | 1 | 1 | 22634000 | 18801000 | 3833300 | 4526800 | 3760200 | 766670 | 2.294150584 |
| Q8C4Q6 | AIDA_MOUSE | Aida | 34.888 | 305 | 1 | 1 | 0 | 6692000 | 6692000 | 0 | 352210 | 352210 | 0 | 2.289462641 |
| Q9R0P5 | DEST_MOUSE | Dstn | 18.522 | 165 | 6 | 4 | 2 | 20995000 | 17424000 | 3570500 | 1615000 | 1340300 | 274650 | 2.286877836 |
| O09106 | HDAC1_MOUSE | Hdac1 | 55.075 | 482 | 4 | 2 | 2 | 15627000 | 12948000 | 2679400 | 625090 | 517920 | 107180 | 2.272747392 |
| Q3UEB3 | PUF60_MOUSE | Puf60 | 60.249 | 564 | 29 | 15 | 14 | 484460000 | 400610000 | 8.4E+07 | 16149000 | 13354000 | 2794900 | 2.256384569 |
| Q9R0E1 | PLOD3_MOUSE | Plod3 | 84.922 | 741 | 2 | 1 | 1 | 4205500 | 3476100 | 729460 | 100130 | 82764 | 17368 | 2.252568811 |
| Q9CPQ1 | COX6C_MOUSE | Cox6c | 8.469 | 76 | 2 | 1 | 1 | 40465000 | 33436000 | 7029600 | 5780700 | 4776500 | 1004200 | 2.249887762 |
| P97379 | G3BP2_MOUSE | G3bp2 | 54.088 | 482 | 14 | 7 | 7 | 167160000 | 138000000 | 2.9E+07 | 5970100 | 4928400 | 1041600 | 2.242308822 |
| Q91ZX7 | LRP1_MOUSE | Lrp1 | 504.742 | 4545 | 57 | 40 | 17 | 1618300000 | 1329000000 | 2.9E+08 | 6421700 | 5273600 | 1148000 | 2.199702876 |
| Q99KV1 | DJB11_MOUSE | Dnajb11 | 40.555 | 358 | 9 | 6 | 3 | 166670000 | 136840000 | 3E+07 | 8771900 | 7202100 | 1569900 | 2.197750857 |
| Q9D173 | TOM7_MOUSE | Tomm7 | 6.177 | 55 | 2 | 1 | 1 | 17533000 | 14372000 | 3160300 | 4383200 | 3593100 | 790070 | 2.185127419 |
| Q99JH8 | ERD21_MOUSE | Kdelr1 | 24.56 | 212 | 2 | 1 | 1 | 13582000 | 11117000 | 2465300 | 1697700 | 1389600 | 308160 | 2.172932397 |
| Q9CQF3 | CPSF5_MOUSE | Nudt21 | 26.24 | 227 | 18 | 10 | 8 | 414540000 | 339130000 | 7.5E+07 | 23030000 | 18841000 | 4189600 | 2.168972396 |
| Q6PA06 | ATLA2_MOUSE | Atl2 | 66.224 | 583 | 2 | 1 | 1 | 5193000 | 4247700 | 945370 | 179070 | 146470 | 32599 | 2.167730889 |
| Q9CPQ8 | ATP5L_MOUSE | Atp5mg | 11.425 | 103 | 6 | 3 | 3 | 54249000 | 44362000 | 9886600 | 9041500 | 7393700 | 1647800 | 2.16577804 |
| Q8BK64 | AHSA1_MOUSE | Ahsa1 | 38.117 | 338 | 4 | 3 | 1 | 17873000 | 14613000 | 3259200 | 777070 | 635370 | 141710 | 2.164662598 |
| Q61102 | ABCB7_MOUSE | Abcb7 | 82.581 | 752 | 4 | 2 | 2 | 20108000 | 16441000 | 3667400 | 490450 | 401000 | 89448 | 2.164468518 |
| Q9CQH3 | NDUB5_MOUSE | Ndufb5 | 21.71 | 189 | 5 | 3 | 2 | 44127000 | 36068000 | 8059600 | 4412700 | 3606800 | 805960 | 2.161939282 |
| Q8K3J1 | NDUS8_MOUSE | Ndufs8 | 24.038 | 212 | 2 | 1 | 1 | 21631000 | 17675000 | 3955800 | 1442100 | 1178400 | 263720 | 2.159668823 |
| Q922Q8 | LRC59_MOUSE | Lrrc59 | 34.877 | 307 | 13 | 7 | 6 | 120770000 | 98643000 | 2.2E+07 | 7104100 | 5802500 | 1301600 | 2.156474016 |
| P59999 | ARPC4_MOUSE | Arpc4 | 19.667 | 168 | 13 | 8 | 5 | 312260000 | 255010000 | 5.7E+07 | 22304000 | 18215000 | 4088900 | 2.155357431 |
| P62317 | SMD2_MOUSE | Snrpd2 | 13.527 | 118 | 14 | 8 | 6 | 216600000 | 176870000 | 4E+07 | 27074000 | 22109000 | 4965800 | 2.154497607 |
| Q9ER72 | SYCC_MOUSE | Cars1 | 94.86 | 831 | 2 | 1 | 1 | 6257200 | 5106100 | 1151000 | 122690 | 100120 | 22569 | 2.149333959 |
| Q9WVQ5 | MTNB_MOUSE | Apip | 26.949 | 241 | 14 | 7 | 7 | 220720000 | 180060000 | 4.1E+07 | 13795000 | 11254000 | 2541100 | 2.146902054 |
| Q80VA0 | GALT7_MOUSE | Galnt7 | 75.419 | 657 | 4 | 2 | 2 | 13690000 | 11166000 | 2523900 | 291280 | 237580 | 53700 | 2.145385806 |
| P56135 | ATPK_MOUSE | Atp5mf | 10.344 | 88 | 4 | 3 | 1 | 75929000 | 61820000 | 1.4E+07 | 18982000 | 15455000 | 3527300 | 2.131457916 |
| O88441 | MTX2_MOUSE | Mtx2 | 29.758 | 263 | 5 | 3 | 2 | 21249000 | 17292000 | 3957000 | 1770700 | 1441000 | 329750 | 2.127625771 |
| Q9EQC5 | SCYL1_MOUSE | Scyl1 | 89.16 | 806 | 4 | 2 | 2 | 10219000 | 8313400 | 1905500 | 243310 | 197940 | 45369 | 2.12526902 |
| Q6ZWR6 | SYNE1_MOUSE | Syne1 | 1009.926 | 8799 | 2 | 1 | 1 | 49304000 | 40101000 | 9202800 | 85301 | 69379 | 15922 | 2.123493433 |
| Q9DCF9 | SSRG_MOUSE | Ssr3 | 21.065 | 185 | 2 | 1 | 1 | 61149000 | 49733000 | 1.1E+07 | 10192000 | 8288900 | 1902600 | 2.12314622 |
| Q91XB0 | TREX1_MOUSE | Trex1 | 33.675 | 314 | 6 | 3 | 3 | 40164000 | 32664000 | 7499900 | 2231300 | 1814700 | 416660 | 2.122758208 |
| P70227 | ITPR3_MOUSE | Itpr3 | 304.275 | 2670 | 26 | 14 | 12 | 114090000 | 92729000 | 2.1E+07 | 755570 | 614100 | 141470 | 2.117973872 |
| P57746 | VATD_MOUSE | Atp6v1d | 28.369 | 247 | 1 | 1 | 0 | 2918400 | 2918400 | 0 | 208460 | 208460 | 0 | 2.111115124 |
| P43276 | H15_MOUSE | H1-5 | 22.576 | 223 | 11 | 6 | 5 | 306260000 | 248650000 | 5.8E+07 | 30626000 | 24865000 | 5761100 | 2.109700226 |
| O35130 | NEP1_MOUSE | Emg1 | 26.974 | 244 | 5 | 3 | 2 | 29162000 | 23654000 | 5508500 | 1715400 | 1391400 | 324030 | 2.102352748 |
| Q0GNC1 | INF2_MOUSE | Inf2 | 138.56 | 1273 | 7 | 4 | 3 | 16520000 | 13389000 | 3130900 | 330410 | 267790 | 62618 | 2.096398877 |
| Q00612 | G6PD1_MOUSE | G6pdx | 59.263 | 515 | 7 | 4 | 3 | 19683000 | 15942000 | 3741500 | 562370 | 455470 | 106900 | 2.091143953 |
| Q3THE2 | ML12B_MOUSE | Myl12b | 19.779 | 172 | 25 | 13 | 12 | 2766600000 | 2238300000 | 5.3E+08 | 251510000 | 203480000 | 4.8E+07 | 2.083056026 |
| Q8VEK3 | HNRPU_MOUSE | Hnrnpu | 87.918 | 800 | 9 | 5 | 4 | 70994000 | 57386000 | 1.4E+07 | 1510500 | 1221000 | 289530 | 2.076243771 |
| Q91YQ5 | RPN1_MOUSE | Rpn1 | 68.528 | 608 | 26 | 14 | 12 | 292740000 | 236560000 | 5.6E+07 | 7140000 | 5769800 | 1370100 | 2.074206024 |
| Q61191 | HCFC1_MOUSE | Hcfc1 | 210.437 | 2045 | 3 | 2 | 1 | 18107000 | 14630000 | 3476600 | 226340 | 182880 | 43458 | 2.073180777 |
| Q91V41 | RAB14_MOUSE | Rab14 | 23.897 | 215 | 3 | 2 | 1 | 14215000 | 11478000 | 2736700 | 888410 | 717370 | 171040 | 2.068362081 |
| P97386 | DNLI3_MOUSE | Lig3 | 113.072 | 1015 | 10 | 5 | 5 | 54505000 | 43988000 | 1.1E+07 | 838540 | 676750 | 161790 | 2.064523961 |
| Q9JJQ0 | PIGB_MOUSE | Pigb | 63.12 | 542 | 4 | 2 | 2 | 7332800 | 5913000 | 1419900 | 244430 | 197100 | 47328 | 2.058100949 |
| P50543 | S10AB_MOUSE | S100a11 | 11.083 | 98 | 6 | 4 | 2 | 47719000 | 38460000 | 9259300 | 9543800 | 7691900 | 1851900 | 2.054383727 |
| Q9CQV1 | TIM16_MOUSE | Pam16 | 13.785 | 125 | 5 | 3 | 2 | 77045000 | 62062000 | 1.5E+07 | 9630600 | 7757800 | 1872800 | 2.050479963 |
| P26041 | MOES_MOUSE | Msn | 67.767 | 577 | 10 | 6 | 4 | 65803000 | 53002000 | 1.3E+07 | 1687300 | 1359000 | 328220 | 2.04990299 |
| P17665 | COX7C_MOUSE | Cox7c | 7.333 | 63 | 2 | 1 | 1 | 27591000 | 22199000 | 5392000 | 5518100 | 4439700 | 1078400 | 2.041602287 |
| A2AH22 | AMRA1_MOUSE | Ambra1 | 142.879 | 1300 | 2 | 1 | 1 | 3412300 | 2743900 | 668440 | 52498 | 42214 | 10284 | 2.037357931 |
| Q9Z1G3 | VATC1_MOUSE | Atp6v1c1 | 43.888 | 382 | 3 | 2 | 1 | 12617000 | 10139000 | 2478100 | 450610 | 362110 | 88504 | 2.032609056 |
| Q9DBG7 | SRPRA_MOUSE | Srpra | 69.623 | 636 | 1 | 1 | 0 | 2203800 | 2203800 | 0 | 51251 | 51251 | 0 | 2.028080678 |
| P17433 | SPI1_MOUSE | Spi1 | 31.349 | 272 | 2 | 1 | 1 | 1863800 | 1496800 | 367040 | 169440 | 136070 | 33367 | 2.027872262 |
| O09110 | MP2K3_MOUSE | Map2k3 | 39.296 | 347 | 3 | 2 | 1 | 9733200 | 7815500 | 1917700 | 389330 | 312620 | 76707 | 2.026961127 |
| Q8K2B3 | SDHA_MOUSE | Sdha | 72.585 | 664 | 7 | 4 | 3 | 18616000 | 14933000 | 3682800 | 503130 | 403590 | 99536 | 2.019629071 |
| Q8JZX4 | SPF45_MOUSE | Rbm17 | 45.304 | 405 | 4 | 2 | 2 | 16125000 | 12933000 | 3192500 | 620210 | 497420 | 122790 | 2.018298443 |
| P52927 | HMGA2_MOUSE | Hmga2 | 11.819 | 108 | 6 | 3 | 3 | 85262000 | 68367000 | 1.7E+07 | 17052000 | 13673000 | 3379000 | 2.01670377 |
| Q99MS7 | EH1L1_MOUSE | Ehbp1l1 | 184.834 | 1716 | 2 | 1 | 1 | 2149500 | 1723000 | 426540 | 21074 | 16892 | 4181.8 | 2.014169756 |
| P35564 | CALX_MOUSE | Canx | 67.278 | 591 | 50 | 25 | 25 | 1540300000 | 1233100000 | 3.1E+08 | 42786000 | 34253000 | 8533400 | 2.005039681 |
| P37040 | NCPR_MOUSE | Por | 77.044 | 678 | 6 | 4 | 2 | 32646000 | 26124000 | 6521400 | 882320 | 706060 | 176260 | 2.002122196 |
| Q8VEE4 | RFA1_MOUSE | Rpa1 | 69.037 | 623 | 34 | 18 | 16 | 405550000 | 324140000 | 8.1E+07 | 11265000 | 9003800 | 2261600 | 1.993179658 |
| P14211 | CALR_MOUSE | Calr | 47.995 | 416 | 11 | 6 | 5 | 102970000 | 82262000 | 2.1E+07 | 3432200 | 2742100 | 690160 | 1.990246945 |
| Q9R0Q9 | MPU1_MOUSE | Mpdu1 | 26.498 | 247 | 4 | 2 | 2 | 17413000 | 13903000 | 3510200 | 2487600 | 1986100 | 501450 | 1.985771084 |
| Q9CQQ7 | AT5F1_MOUSE | Atp5pb | 28.949 | 256 | 8 | 5 | 3 | 134800000 | 107610000 | 2.7E+07 | 7489100 | 5978200 | 1510900 | 1.984345771 |
| P49718 | MCM5_MOUSE | Mcm5 | 82.407 | 734 | 3 | 2 | 1 | 12372000 | 9868200 | 2503400 | 252480 | 201390 | 51090 | 1.978898128 |
| P47911 | RL6_MOUSE | Rpl6 | 33.51 | 296 | 14 | 7 | 7 | 344880000 | 274720000 | 7E+07 | 19160000 | 15262000 | 3897700 | 1.969261855 |
| Q64310 | SURF4_MOUSE | Surf4 | 30.381 | 269 | 7 | 4 | 3 | 35970000 | 28652000 | 7318200 | 3270000 | 2604700 | 665290 | 1.969075098 |
| Q64337 | SQSTM_MOUSE | Sqstm1 | 48.163 | 442 | 4 | 2 | 2 | 13128000 | 10453000 | 2675300 | 690950 | 550140 | 140810 | 1.966144469 |
| Q9D0K2 | SCOT1_MOUSE | Oxct1 | 55.989 | 520 | 2 | 1 | 1 | 3344900 | 2661700 | 683190 | 133800 | 106470 | 27328 | 1.961989211 |
| Q91VC9 | GHITM_MOUSE | Ghitm | 37.275 | 346 | 3 | 2 | 1 | 11264000 | 8952400 | 2311600 | 804570 | 639460 | 165110 | 1.953382723 |
| Q8BGR2 | LRC8D_MOUSE | Lrrc8d | 98.113 | 859 | 3 | 2 | 1 | 9183700 | 7288200 | 1895500 | 199650 | 158440 | 41207 | 1.942984093 |
| Q91YR7 | PRP6_MOUSE | Prpf6 | 106.722 | 941 | 11 | 7 | 4 | 51572000 | 40907000 | 1.1E+07 | 831800 | 659790 | 172010 | 1.939599053 |
| E9Q9F6 | CTSRD_MOUSE | Catsperd | 91.096 | 805 | 3 | 2 | 1 | 24330000 | 19279000 | 5050200 | 675820 | 535540 | 140280 | 1.932617793 |
| Q9CQM2 | ERD22_MOUSE | Kdelr2 | 24.454 | 212 | 5 | 3 | 2 | 77440000 | 61294000 | 1.6E+07 | 9680000 | 7661700 | 2018300 | 1.92456906 |
| Q9DBC3 | CMTR1_MOUSE | Cmtr1 | 95.676 | 837 | 2 | 1 | 1 | 2708600 | 2142500 | 566060 | 45909 | 36314 | 9594.2 | 1.920268318 |
| P62900 | RL31_MOUSE | Rpl31 | 14.463 | 125 | 5 | 3 | 2 | 697630000 | 551140000 | 1.5E+08 | 99662000 | 78735000 | 2.1E+07 | 1.911616653 |
| P23475 | XRCC6_MOUSE | Xrcc6 | 69.484 | 608 | 11 | 7 | 4 | 51824000 | 40902000 | 1.1E+07 | 1480700 | 1168600 | 312070 | 1.904934327 |
| Q9EQS3 | MYCBP_MOUSE | Mycbp | 11.97 | 103 | 6 | 3 | 3 | 52032000 | 40998000 | 1.1E+07 | 7433100 | 5856900 | 1576300 | 1.893597647 |
| Q8BX70 | VP13C_MOUSE | Vps13c | 420.089 | 3748 | 2 | 1 | 1 | 1767300 | 1391200 | 376060 | 8106.7 | 6381.6 | 1725.1 | 1.887295072 |
| P42208 | SEPT2_MOUSE | Septin2 | 41.526 | 361 | 7 | 4 | 3 | 21086000 | 16594000 | 4492700 | 958470 | 754260 | 204210 | 1.885007057 |
| Q9WU28 | PFD5_MOUSE | Pfdn5 | 17.356 | 154 | 2 | 1 | 1 | 4286500 | 3373100 | 913450 | 428650 | 337310 | 91345 | 1.884677423 |
| P11680 | PROP_MOUSE | Cfp | 50.327 | 464 | 10 | 6 | 4 | 120290000 | 94608000 | 2.6E+07 | 5728100 | 4505100 | 1223000 | 1.881204625 |
| Q3UBG2 | PCLI1_MOUSE | Pid1 | 24.784 | 217 | 3 | 2 | 1 | 16065000 | 12598000 | 3467700 | 1004100 | 787350 | 216730 | 1.861143719 |
| Q8VD65 | PI3R4_MOUSE | Pik3r4 | 152.599 | 1358 | 4 | 2 | 2 | 11456000 | 8977500 | 2478200 | 156930 | 122980 | 33947 | 1.857021125 |
| Q9JJI8 | RL38_MOUSE | Rpl38 | 8.204 | 70 | 14 | 7 | 7 | 325000000 | 254620000 | 7E+07 | 108330000 | 84874000 | 2.3E+07 | 1.855128825 |
| Q9JKF1 | IQGA1_MOUSE | Iqgap1 | 188.742 | 1657 | 31 | 18 | 13 | 166830000 | 130690000 | 3.6E+07 | 1756100 | 1375700 | 380400 | 1.854560185 |
| Q9CQW2 | ARL8B_MOUSE | Arl8b | 21.539 | 186 | 4 | 2 | 2 | 21156000 | 16530000 | 4625500 | 1627400 | 1271600 | 355810 | 1.837405495 |
| Q06185 | ATP5I_MOUSE | Atp5me | 8.236 | 71 | 4 | 2 | 2 | 67105000 | 52406000 | 1.5E+07 | 16776000 | 13102000 | 3674700 | 1.834013987 |
| Q9CR67 | TMM33_MOUSE | Tmem33 | 28.031 | 247 | 6 | 3 | 3 | 68641000 | 53586000 | 1.5E+07 | 5720100 | 4465500 | 1254600 | 1.83161342 |
| Q8R1B4 | EIF3C_MOUSE | Eif3c | 105.531 | 911 | 17 | 9 | 8 | 92882000 | 72492000 | 2E+07 | 2064000 | 1610900 | 453120 | 1.829889264 |
| Q8BHN3 | GANAB_MOUSE | Ganab | 106.911 | 944 | 2 | 1 | 1 | 2992600 | 2335100 | 657490 | 52502 | 40967 | 11535 | 1.828443477 |
| Q3THS6 | METK2_MOUSE | Mat2a | 43.689 | 395 | 14 | 8 | 6 | 93994000 | 73325000 | 2.1E+07 | 4699700 | 3666300 | 1033500 | 1.826836575 |
| O35286 | DHX15_MOUSE | Dhx15 | 91.007 | 795 | 21 | 12 | 9 | 124210000 | 96845000 | 2.7E+07 | 2700300 | 2105300 | 594980 | 1.823134843 |
| P0DP28 | CALM3_MOUSE | Calm3 | 16.838 | 149 | 31 | 16 | 15 | 2340900000 | 1821500000 | 5.2E+08 | 260100000 | 202390000 | 5.8E+07 | 1.810320185 |
| Q61081 | CDC37_MOUSE | Cdc37 | 44.593 | 379 | 2 | 1 | 1 | 5066400 | 3937600 | 1128800 | 253320 | 196880 | 56440 | 1.80252667 |
| Q8VDL4 | ADPGK_MOUSE | Adpgk | 53.902 | 496 | 3 | 2 | 1 | 14259000 | 11075000 | 3183600 | 648130 | 503430 | 144710 | 1.798575712 |
| Q920Q4 | VPS16_MOUSE | Vps16 | 94.928 | 839 | 3 | 2 | 1 | 13660000 | 10610000 | 3050300 | 284590 | 221040 | 63548 | 1.798401611 |
| Q9CR57 | RL14_MOUSE | Rpl14 | 23.564 | 217 | 2 | 1 | 1 | 24052000 | 18650000 | 5401900 | 2004400 | 1554200 | 450160 | 1.787636792 |
| P16110 | LEG3_MOUSE | Lgals3 | 27.515 | 264 | 6 | 3 | 3 | 42707000 | 33110000 | 9597400 | 4270700 | 3311000 | 959740 | 1.786551483 |
| Q921L3 | TMCO1_MOUSE | Tmco1 | 21.175 | 188 | 6 | 3 | 3 | 30667000 | 23768000 | 6899500 | 4381000 | 3395400 | 985650 | 1.78445679 |
| Q8BGH2 | SAM50_MOUSE | Samm50 | 51.864 | 469 | 4 | 3 | 1 | 26908000 | 20847000 | 6060900 | 791420 | 613150 | 178260 | 1.782235842 |
| P29341 | PABP1_MOUSE | Pabpc1 | 70.671 | 636 | 39 | 20 | 19 | 980010000 | 758420000 | 2.2E+08 | 22791000 | 17638000 | 5153100 | 1.775104233 |
| Q8VEA8 | RAB7B_MOUSE | Rab7b | 22.502 | 199 | 2 | 1 | 1 | 6096800 | 4717800 | 1379000 | 406450 | 314520 | 91936 | 1.774491803 |
| O08528 | HXK2_MOUSE | Hk2 | 102.535 | 917 | 5 | 3 | 2 | 16843000 | 13027000 | 3816000 | 306240 | 236850 | 69382 | 1.771371806 |
| O35658 | C1QBP_MOUSE | C1qbp | 31.013 | 278 | 8 | 4 | 4 | 104580000 | 80876000 | 2.4E+07 | 6972100 | 5391700 | 1580400 | 1.770459393 |
| Q3TKY6 | CWC27_MOUSE | Cwc27 | 53.543 | 469 | 7 | 4 | 3 | 81387000 | 62883000 | 1.9E+07 | 3391100 | 2620100 | 771010 | 1.764832876 |
| Q3TIX9 | SNUT2_MOUSE | Usp39 | 65.146 | 564 | 15 | 8 | 7 | 62635000 | 48384000 | 1.4E+07 | 1898000 | 1466200 | 431830 | 1.763466887 |
| Q91V81 | RBM42_MOUSE | Rbm42 | 50.236 | 478 | 2 | 1 | 1 | 7838400 | 6053600 | 1784800 | 391920 | 302680 | 89239 | 1.762030932 |
| Q3UWW6 | GA2L3_MOUSE | Gas2l3 | 74.235 | 683 | 7 | 4 | 3 | 20855000 | 16105000 | 4749800 | 463440 | 357890 | 105550 | 1.761569988 |
| P32067 | LA_MOUSE | Ssb | 47.756 | 415 | 13 | 7 | 6 | 93464000 | 72171000 | 2.1E+07 | 3115500 | 2405700 | 709760 | 1.761040017 |
| P12970 | RL7A_MOUSE | Rpl7a | 29.977 | 266 | 14 | 7 | 7 | 472060000 | 364400000 | 1.1E+08 | 33718000 | 26029000 | 7689500 | 1.75917464 |
| P12382 | PFKAL_MOUSE | Pfkl | 85.36 | 780 | 15 | 9 | 6 | 101060000 | 78001000 | 2.3E+07 | 2526500 | 1950000 | 576530 | 1.758037546 |
| Q9D898 | ARP5L_MOUSE | Arpc5l | 16.98 | 153 | 6 | 3 | 3 | 37597000 | 28999000 | 8597300 | 3133100 | 2416600 | 716440 | 1.754047597 |
| Q8K2C7 | OS9_MOUSE | Os9 | 76.108 | 672 | 7 | 4 | 3 | 29521000 | 22770000 | 6751600 | 720040 | 555360 | 164670 | 1.753832953 |
| F8VPN2 | TEX15_MOUSE | Tex15 | 311.28 | 2785 | 2 | 1 | 1 | 94467000 | 72857000 | 2.2E+07 | 669980 | 516710 | 153260 | 1.753368519 |
| P97493 | THIOM_MOUSE | Txn2 | 18.255 | 166 | 4 | 2 | 2 | 32172000 | 24788000 | 7383400 | 3574600 | 2754200 | 820380 | 1.747284649 |
| Q60766 | IRGM1_MOUSE | Irgm1 | 46.552 | 409 | 4 | 3 | 1 | 8820100 | 6794700 | 2025400 | 420000 | 323560 | 96448 | 1.746202998 |
| P62918 | RL8_MOUSE | Rpl8 | 28.025 | 257 | 4 | 2 | 2 | 31152000 | 23992000 | 7159100 | 2396300 | 1845600 | 550700 | 1.74470329 |
| P25911 | LYN_MOUSE | Lyn | 58.812 | 512 | 4 | 2 | 2 | 11028000 | 8484300 | 2543300 | 334170 | 257100 | 77069 | 1.738093987 |
| P55096 | ABCD3_MOUSE | Abcd3 | 75.475 | 659 | 2 | 1 | 1 | 13809000 | 10598000 | 3211100 | 306860 | 235500 | 71358 | 1.722652533 |
| O55142 | RL35A_MOUSE | Rpl35a | 12.554 | 110 | 8 | 4 | 4 | 160920000 | 123420000 | 3.7E+07 | 17879000 | 13714000 | 4165800 | 1.718921507 |
| Q6A068 | CDC5L_MOUSE | Cdc5l | 92.19 | 802 | 4 | 2 | 2 | 7360400 | 5643100 | 1717300 | 153340 | 117560 | 35777 | 1.716345826 |
| Q9JIK5 | DDX21_MOUSE | Ddx21 | 93.551 | 851 | 9 | 5 | 4 | 23411000 | 17936000 | 5474900 | 450200 | 344920 | 105290 | 1.711953664 |
| Q9D0R8 | LSM12_MOUSE | Lsm12 | 21.701 | 195 | 4 | 2 | 2 | 28473000 | 21813000 | 6660700 | 2372800 | 1817700 | 555060 | 1.711442492 |
| Q8R404 | MIC13_MOUSE | Micos13 | 13.373 | 119 | 4 | 2 | 2 | 33327000 | 25519000 | 7807500 | 6665300 | 5103800 | 1561500 | 1.708639227 |
| Q80ZW2 | THEM6_MOUSE | Them6 | 23.803 | 207 | 12 | 6 | 6 | 79071000 | 60526000 | 1.9E+07 | 4651200 | 3560400 | 1090900 | 1.706602539 |
| P16675 | PPGB_MOUSE | Ctsa | 53.844 | 474 | 8 | 4 | 4 | 68399000 | 52331000 | 1.6E+07 | 3109000 | 2378700 | 730340 | 1.703475461 |
| Q9R1K9 | CETN2_MOUSE | Cetn2 | 19.797 | 172 | 2 | 1 | 1 | 19261000 | 14735000 | 4526500 | 1926100 | 1473500 | 452650 | 1.702779201 |
| Q3U9G9 | LBR_MOUSE | Lbr | 71.44 | 626 | 11 | 5 | 6 | 69876000 | 53437000 | 1.6E+07 | 2329200 | 1781200 | 547960 | 1.700716475 |
| Q8BG60 | TXNIP_MOUSE | Txnip | 44.363 | 397 | 2 | 1 | 1 | 2658900 | 2032400 | 626540 | 120860 | 92382 | 28479 | 1.697705846 |
| Q99JI4 | PSMD6_MOUSE | Psmd6 | 45.536 | 389 | 17 | 10 | 7 | 119280000 | 91125000 | 2.8E+07 | 4113100 | 3142200 | 970900 | 1.694404518 |
| Q8C7X2 | EMC1_MOUSE | Emc1 | 111.605 | 997 | 8 | 4 | 4 | 22699000 | 17336000 | 5362800 | 436520 | 333390 | 103130 | 1.692712703 |
| Q9JLJ5 | ELOV1_MOUSE | Elovl1 | 32.678 | 279 | 9 | 5 | 4 | 29328000 | 22370000 | 6957800 | 4189700 | 3195700 | 993970 | 1.684862142 |
| Q9WTX6 | CUL1_MOUSE | Cul1 | 89.692 | 776 | 33 | 19 | 14 | 250400000 | 190790000 | 6E+07 | 4815300 | 3669100 | 1146200 | 1.678552909 |
| Q9D0C1 | RN115_MOUSE | Rnf115 | 33.859 | 305 | 2 | 1 | 1 | 7654900 | 5828500 | 1826400 | 546780 | 416320 | 130460 | 1.67412188 |
| P24369 | PPIB_MOUSE | Ppib | 23.713 | 216 | 10 | 6 | 4 | 167600000 | 127580000 | 4E+07 | 11173000 | 8505400 | 2667800 | 1.672717263 |
| Q9QXS1 | PLEC_MOUSE | Plec | 534.188 | 4691 | 208 | 114 | 94 | 1824700000 | 1388800000 | 4.4E+08 | 5288900 | 4025600 | 1263300 | 1.672001441 |
| Q6ZWM4 | LSM8_MOUSE | Lsm8 | 10.403 | 96 | 2 | 1 | 1 | 6654200 | 5063100 | 1591100 | 950600 | 723300 | 227310 | 1.669996468 |
| Q99LJ7 | RCBT2_MOUSE | Rcbtb2 | 60.164 | 551 | 13 | 7 | 6 | 129670000 | 98571000 | 3.1E+07 | 6483700 | 4928600 | 1555100 | 1.664109522 |
| Q8BK67 | RCC2_MOUSE | Rcc2 | 55.983 | 520 | 7 | 4 | 3 | 21218000 | 16120000 | 5098000 | 642960 | 488470 | 154490 | 1.660848465 |
| Q8BG73 | SH3L2_MOUSE | Sh3bgrl2 | 12.255 | 107 | 8 | 4 | 4 | 71119000 | 54028000 | 1.7E+07 | 7902100 | 6003100 | 1898900 | 1.66055488 |
| Q8R1I1 | QCR9_MOUSE | Uqcr10 | 7.446 | 64 | 4 | 2 | 2 | 46172000 | 35046000 | 1.1E+07 | 11543000 | 8761500 | 2781600 | 1.65531478 |
| Q99J56 | DERL1_MOUSE | Derl1 | 28.835 | 251 | 3 | 2 | 1 | 34651000 | 26268000 | 8382800 | 3150100 | 2388000 | 762070 | 1.647802246 |
| Q64213 | SF01_MOUSE | Sf1 | 70.403 | 653 | 5 | 3 | 2 | 29207000 | 22136000 | 7070800 | 1327600 | 1006200 | 321400 | 1.646449191 |
| P97351 | RS3A_MOUSE | Rps3a | 29.885 | 264 | 20 | 11 | 9 | 308430000 | 233650000 | 7.5E+07 | 16233000 | 12297000 | 3935700 | 1.643663246 |
| P56183 | RRP1_MOUSE | Rrp1 | 54.777 | 494 | 2 | 1 | 1 | 9675700 | 7329000 | 2346700 | 387030 | 293160 | 93868 | 1.642982944 |
| Q9JM76 | ARPC3_MOUSE | Arpc3 | 20.525 | 178 | 15 | 8 | 7 | 401670000 | 304240000 | 9.7E+07 | 33473000 | 25353000 | 8119400 | 1.642727451 |
| Q8R081 | HNRPL_MOUSE | Hnrnpl | 63.964 | 586 | 5 | 3 | 2 | 37111000 | 28094000 | 9017000 | 1686800 | 1277000 | 409860 | 1.639542622 |
| Q9JHJ0 | TMOD3_MOUSE | Tmod3 | 39.503 | 352 | 36 | 20 | 16 | 953250000 | 720520000 | 2.3E+08 | 39719000 | 30022000 | 9697000 | 1.630381284 |
| Q62425 | NDUA4_MOUSE | Ndufa4 | 9.327 | 82 | 6 | 3 | 3 | 590240000 | 446000000 | 1.4E+08 | 98374000 | 74333000 | 2.4E+07 | 1.628472391 |
| Q8BG05 | ROA3_MOUSE | Hnrnpa3 | 39.652 | 379 | 11 | 7 | 4 | 274880000 | 207210000 | 6.8E+07 | 13744000 | 10361000 | 3383500 | 1.614484016 |
| Q61335 | BAP31_MOUSE | Bcap31 | 27.957 | 245 | 2 | 1 | 1 | 16505000 | 12435000 | 4069200 | 1100300 | 829030 | 271280 | 1.611589412 |
| P14069 | S10A6_MOUSE | S100a6 | 10.051 | 89 | 3 | 2 | 1 | 79669000 | 60024000 | 2E+07 | 15934000 | 12005000 | 3928900 | 1.611450736 |
| Q8BYP3 | RHOF_MOUSE | Rhof | 23.577 | 211 | 2 | 1 | 1 | 4221700 | 3180600 | 1041000 | 351810 | 265050 | 86754 | 1.611328878 |
| O88668 | CREG1_MOUSE | Creg1 | 24.452 | 220 | 2 | 1 | 1 | 6385000 | 4807900 | 1577100 | 580460 | 437090 | 143370 | 1.608132749 |
| Q6NXM2 | RCBT1_MOUSE | Rcbtb1 | 58.379 | 531 | 13 | 7 | 6 | 240640000 | 181070000 | 6E+07 | 14155000 | 10651000 | 3504100 | 1.603889674 |
| Q60597 | ODO1_MOUSE | Ogdh | 116.449 | 1023 | 9 | 5 | 4 | 34175000 | 25710000 | 8465500 | 670100 | 504110 | 165990 | 1.602662424 |
| Q9DCX2 | ATP5H_MOUSE | Atp5pd | 18.749 | 161 | 18 | 10 | 8 | 351320000 | 264260000 | 8.7E+07 | 29277000 | 22022000 | 7254900 | 1.601892713 |
| Q99M87 | DNJA3_MOUSE | Dnaja3 | 52.443 | 480 | 12 | 7 | 5 | 113240000 | 85049000 | 2.8E+07 | 4355200 | 3271100 | 1084100 | 1.59331552 |
| Q9DBH5 | LMAN2_MOUSE | Lman2 | 40.43 | 358 | 2 | 1 | 1 | 4317100 | 3238000 | 1079100 | 187700 | 140780 | 46916 | 1.58527442 |
| Q9JLV6 | PNKP_MOUSE | Pnkp | 57.223 | 522 | 13 | 7 | 6 | 123910000 | 92896000 | 3.1E+07 | 4130300 | 3096500 | 1033800 | 1.582696869 |
| Q60596 | XRCC1_MOUSE | Xrcc1 | 68.971 | 631 | 9 | 6 | 3 | 68131000 | 51057000 | 1.7E+07 | 2064600 | 1547200 | 517370 | 1.580392185 |
| Q8BRH4 | KMT2C_MOUSE | Kmt2c | 540.187 | 4903 | 2 | 1 | 1 | 106000000 | 79127000 | 2.7E+07 | 415690 | 310300 | 105380 | 1.558012673 |
| P68134 | ACTS_MOUSE | Acta1 | 42.051 | 377 | 17 | 6 | 11 | 1.3568E+10 | 1.012E+10 | 3.4E+09 | 589900000 | 439990000 | 1.5E+08 | 1.55337761 |
| Q61510 | TRI25_MOUSE | Trim25 | 71.726 | 634 | 9 | 5 | 4 | 55462000 | 41367000 | 1.4E+07 | 1289800 | 962020 | 327800 | 1.553296857 |
| Q9CYH2 | PXL2A_MOUSE | Prxl2a | 24.395 | 218 | 2 | 1 | 1 | 11748000 | 8761600 | 2986500 | 783210 | 584110 | 199100 | 1.55273863 |
| P27048 | RSMB_MOUSE | Snrpb | 23.656 | 231 | 8 | 4 | 4 | 336260000 | 250770000 | 8.5E+07 | 21017000 | 15673000 | 5343500 | 1.552419063 |
| P10404 | ENV1_MOUSE |  | 69.613 | 641 | 14 | 8 | 6 | 288000000 | 214340000 | 7.4E+07 | 8999800 | 6698200 | 2301600 | 1.541084915 |
| P61982 | 1433G_MOUSE | Ywhag | 28.303 | 247 | 5 | 3 | 2 | 96353000 | 71684000 | 2.5E+07 | 5352900 | 3982500 | 1370500 | 1.539010391 |
| Q8CGK3 | LONM_MOUSE | Lonp1 | 105.843 | 949 | 2 | 1 | 1 | 7292200 | 5423400 | 1868800 | 135040 | 100430 | 34608 | 1.5370854 |
| O35218 | CPSF2_MOUSE | Cpsf2 | 88.383 | 782 | 2 | 1 | 1 | 6261800 | 4654000 | 1607800 | 136130 | 101170 | 34952 | 1.533383255 |
| Q9R233 | TPSN_MOUSE | Tapbp | 49.736 | 465 | 4 | 2 | 2 | 19469000 | 14470000 | 4999500 | 884970 | 657720 | 227250 | 1.533209199 |
| Q62095 | DDX3Y_MOUSE | Ddx3y | 73.428 | 658 | 10 | 6 | 4 | 76064000 | 56510000 | 2E+07 | 1690300 | 1255800 | 434540 | 1.531042431 |
| Q9QZ88 | VPS29_MOUSE | Vps29 | 20.496 | 182 | 4 | 2 | 2 | 17193000 | 12770000 | 4423800 | 1563000 | 1160900 | 402160 | 1.529400457 |
| Q80UM7 | MOGS_MOUSE | Mogs | 91.831 | 834 | 23 | 12 | 11 | 159930000 | 118760000 | 4.1E+07 | 3900800 | 2896600 | 1004200 | 1.528313562 |
| Q9DBR7 | MYPT1_MOUSE | Ppp1r12a | 114.996 | 1029 | 2 | 1 | 1 | 5124300 | 3804700 | 1319600 | 93168 | 69176 | 23992 | 1.527682019 |
| Q8VDH1 | FBX21_MOUSE | Fbxo21 | 72.173 | 627 | 8 | 4 | 4 | 51884000 | 38477000 | 1.3E+07 | 1853000 | 1374200 | 478820 | 1.521009868 |
| P62245 | RS15A_MOUSE | Rps15a | 14.84 | 130 | 10 | 5 | 5 | 129900000 | 96228000 | 3.4E+07 | 12990000 | 9622800 | 3366900 | 1.515035872 |
| Q8BTZ5 | ANR46_MOUSE | Ankrd46 | 25.223 | 228 | 2 | 1 | 1 | 9254300 | 6846300 | 2407900 | 1322000 | 978050 | 343990 | 1.507549033 |
| O54734 | OST48_MOUSE | Ddost | 49.028 | 441 | 24 | 13 | 11 | 1340900000 | 991820000 | 3.5E+08 | 53636000 | 39673000 | 1.4E+07 | 1.506561944 |
| Q8CCF0 | PRP31_MOUSE | Prpf31 | 55.43 | 499 | 14 | 7 | 7 | 73759000 | 54537000 | 1.9E+07 | 3073300 | 2272400 | 800930 | 1.504476889 |
| Q6PCN7 | HLTF_MOUSE | Hltf | 113.317 | 1003 | 8 | 4 | 4 | 37700000 | 27859000 | 9841500 | 628340 | 464310 | 164030 | 1.501193346 |
| Q3UJU9 | RMD3_MOUSE | Rmdn3 | 52.029 | 470 | 2 | 1 | 1 | 9178600 | 6778400 | 2400200 | 339950 | 251050 | 88896 | 1.497790149 |
| P35980 | RL18_MOUSE | Rpl18 | 21.645 | 188 | 6 | 3 | 3 | 155940000 | 115000000 | 4.1E+07 | 17326000 | 12778000 | 4548400 | 1.490227061 |
| Q91VH2 | SNX9_MOUSE | Snx9 | 66.546 | 595 | 15 | 7 | 8 | 87794000 | 64683000 | 2.3E+07 | 3027400 | 2230500 | 796910 | 1.484869333 |
| Q9R0N0 | GALK1_MOUSE | Galk1 | 42.295 | 392 | 6 | 4 | 2 | 43207000 | 31825000 | 1.1E+07 | 2541600 | 1872000 | 669530 | 1.48340643 |
| Q9CXW4 | RL11_MOUSE | Rpl11 | 20.252 | 178 | 8 | 4 | 4 | 153570000 | 113110000 | 4E+07 | 15357000 | 11311000 | 4045500 | 1.483336556 |
| P62627 | DLRB1_MOUSE | Dynlrb1 | 10.99 | 96 | 5 | 3 | 2 | 48775000 | 35876000 | 1.3E+07 | 8129100 | 5979300 | 2149800 | 1.475759821 |
| Q9CYN9 | RENR_MOUSE | Atp6ap2 | 39.092 | 350 | 10 | 6 | 4 | 49156000 | 36142000 | 1.3E+07 | 2891500 | 2126000 | 765550 | 1.473611884 |
| P47757 | CAPZB_MOUSE | Capzb | 31.345 | 277 | 30 | 17 | 13 | 853770000 | 626830000 | 2.3E+08 | 42688000 | 31341000 | 1.1E+07 | 1.465763311 |
| Q9D024 | CCD47_MOUSE | Ccdc47 | 55.844 | 483 | 4 | 2 | 2 | 13624000 | 9975600 | 3648500 | 619280 | 453430 | 165840 | 1.451100164 |
| Q9CX56 | PSMD8_MOUSE | Psmd8 | 39.93 | 353 | 12 | 6 | 6 | 148820000 | 108940000 | 4E+07 | 6764500 | 4952000 | 1812500 | 1.449977348 |
| P61022 | CHP1_MOUSE | Chp1 | 22.432 | 195 | 8 | 4 | 4 | 47506000 | 34776000 | 1.3E+07 | 2969100 | 2173500 | 795640 | 1.449859581 |
| Q8K297 | GT251_MOUSE | Colgalt1 | 71.061 | 617 | 2 | 1 | 1 | 4662800 | 3411300 | 1251500 | 116570 | 85282 | 31288 | 1.446663344 |
| P57780 | ACTN4_MOUSE | Actn4 | 104.977 | 912 | 128 | 68 | 60 | 7387500000 | 5400700000 | 2E+09 | 123120000 | 90012000 | 3.3E+07 | 1.442699759 |
| Q3U308 | CTU2_MOUSE | Ctu2 | 56.105 | 514 | 13 | 6 | 7 | 67656000 | 49440000 | 1.8E+07 | 2416300 | 1765700 | 650540 | 1.440551748 |
| Q6ZQI3 | MLEC_MOUSE | Mlec | 32.342 | 291 | 7 | 4 | 3 | 31573000 | 23060000 | 8513300 | 1754100 | 1281100 | 472960 | 1.437602137 |
| Q78XF5 | OSTC_MOUSE | Ostc | 16.815 | 149 | 2 | 1 | 1 | 31738000 | 23177000 | 8561400 | 4534000 | 3311000 | 1223100 | 1.436775201 |
| Q6P8I4 | PCNP_MOUSE | Pcnp | 18.963 | 178 | 4 | 2 | 2 | 34517000 | 25189000 | 9327800 | 3451700 | 2518900 | 932780 | 1.433185088 |
| P48036 | ANXA5_MOUSE | Anxa5 | 35.752 | 319 | 2 | 1 | 1 | 7162000 | 5226100 | 1935900 | 286480 | 209040 | 77435 | 1.432730299 |
| P00416 | COX3_MOUSE | mt-Co3 | 29.923 | 261 | 4 | 2 | 2 | 73123000 | 53351000 | 2E+07 | 36561000 | 26676000 | 9885800 | 1.432056501 |
| Q9Z0X1 | AIFM1_MOUSE | Aifm1 | 66.765 | 612 | 13 | 7 | 6 | 93136000 | 67940000 | 2.5E+07 | 2739300 | 1998200 | 741060 | 1.431066502 |
| P62880 | GBB2_MOUSE | Gnb2 | 37.331 | 340 | 9 | 5 | 4 | 114220000 | 83230000 | 3.1E+07 | 8785800 | 6402300 | 2383500 | 1.425487109 |
| O70551 | SRPK1_MOUSE | Srpk1 | 73.088 | 648 | 15 | 8 | 7 | 95027000 | 69231000 | 2.6E+07 | 2879600 | 2097900 | 781700 | 1.424270813 |
| Q61233 | PLSL_MOUSE | Lcp1 | 70.149 | 627 | 34 | 18 | 16 | 275230000 | 200300000 | 7.5E+07 | 6713000 | 4885400 | 1827600 | 1.418489304 |
| Q9JMK0 | B4GT5_MOUSE | B4galt5 | 44.814 | 388 | 2 | 1 | 1 | 4473800 | 3255600 | 1218200 | 235460 | 171350 | 64116 | 1.418172444 |
| Q80ZK0 | RT10_MOUSE | Mrps10 | 18.698 | 160 | 5 | 3 | 2 | 32287000 | 23469000 | 8818200 | 2690600 | 1955700 | 734850 | 1.412200268 |
| Q8BFQ4 | WDR82_MOUSE | Wdr82 | 35.079 | 313 | 11 | 6 | 5 | 46301000 | 33565000 | 1.3E+07 | 2315100 | 1678200 | 636820 | 1.398045401 |
| P68510 | 1433F_MOUSE | Ywhah | 28.212 | 246 | 8 | 4 | 4 | 79523000 | 57594000 | 2.2E+07 | 4417900 | 3199700 | 1218200 | 1.39314429 |
| Q9EQ06 | DHB11_MOUSE | Hsd17b11 | 32.881 | 298 | 3 | 2 | 1 | 11914000 | 8617100 | 3297100 | 700840 | 506890 | 193950 | 1.386004782 |
| P08752 | GNAI2_MOUSE | Gnai2 | 40.489 | 355 | 7 | 4 | 3 | 35288000 | 25509000 | 9779100 | 1764400 | 1275500 | 488950 | 1.383232743 |
| P20491 | FCERG_MOUSE | Fcer1g | 9.652 | 86 | 4 | 2 | 2 | 86024000 | 62120000 | 2.4E+07 | 28675000 | 20707000 | 7967800 | 1.377866131 |
| Q8K358 | PIGU_MOUSE | Pigu | 49.804 | 434 | 4 | 2 | 2 | 6497100 | 4688600 | 1808500 | 541430 | 390720 | 150710 | 1.374363605 |
| Q8BSL7 | ARF2_MOUSE | Arf2 | 20.746 | 181 | 2 | 1 | 1 | 5465200 | 3943900 | 1521400 | 496840 | 358530 | 138310 | 1.374223462 |
| Q8BWM0 | PGES2_MOUSE | Ptges2 | 43.324 | 384 | 3 | 1 | 2 | 10247000 | 7391000 | 2855700 | 379510 | 273740 | 105770 | 1.371925146 |
| P09528 | FRIH_MOUSE | Fth1 | 21.067 | 182 | 9 | 5 | 4 | 119620000 | 86158000 | 3.3E+07 | 9968700 | 7179800 | 2788800 | 1.364288637 |
| P03930 | ATP8_MOUSE | Mtatp8 | 7.766 | 67 | 2 | 1 | 1 | 24961000 | 17950000 | 7011200 | 4992100 | 3589900 | 1402200 | 1.356250549 |
| Q9CQ71 | RFA3_MOUSE | Rpa3 | 13.584 | 121 | 7 | 4 | 3 | 85503000 | 61485000 | 2.4E+07 | 9500300 | 6831600 | 2668700 | 1.356058403 |
| Q8BXA5 | CLP1L_MOUSE | Clptm1l | 62.183 | 539 | 2 | 1 | 1 | 3476400 | 2499200 | 977150 | 124160 | 89257 | 34898 | 1.35481441 |
| Q99LL5 | PWP1_MOUSE | Pwp1 | 55.587 | 501 | 2 | 1 | 1 | 4170300 | 2995700 | 1174600 | 181320 | 130250 | 51069 | 1.350723611 |
| Q91YE6 | IPO9_MOUSE | Ipo9 | 116.052 | 1041 | 10 | 5 | 5 | 49941000 | 35873000 | 1.4E+07 | 1189100 | 854130 | 334950 | 1.35048116 |
| Q9D1K2 | VATF_MOUSE | Atp6v1f | 13.37 | 119 | 3 | 2 | 1 | 8912500 | 6400900 | 2511600 | 1114100 | 800110 | 313950 | 1.349668052 |
| Q80SW1 | SAHH2_MOUSE | Ahcyl1 | 58.951 | 530 | 2 | 1 | 1 | 6764900 | 4855000 | 1910000 | 205000 | 147120 | 57878 | 1.345898657 |
| Q99ME9 | GTPB4_MOUSE | Gtpbp4 | 74.113 | 634 | 3 | 2 | 1 | 12452000 | 8935600 | 3516200 | 289580 | 207810 | 81771 | 1.345547473 |
| Q7TMK9 | HNRPQ_MOUSE | Syncrip | 69.633 | 623 | 15 | 8 | 7 | 141490000 | 101300000 | 4E+07 | 3627900 | 2597400 | 1030500 | 1.333761589 |
| P62960 | YBOX1_MOUSE | Ybx1 | 35.73 | 322 | 16 | 8 | 8 | 666040000 | 476500000 | 1.9E+08 | 47574000 | 34036000 | 1.4E+07 | 1.329973871 |
| Q8K019 | BCLF1_MOUSE | Bclaf1 | 106.002 | 919 | 4 | 2 | 2 | 11978000 | 8567000 | 3410700 | 244440 | 174840 | 69607 | 1.328722225 |
| P60710 | ACTB_MOUSE | Actb | 41.737 | 375 | 185 | 81 | 104 | 8.3358E+10 | 5.954E+10 | 2.4E+10 | 3.624E+09 | 2.589E+09 | 1E+09 | 1.321806947 |
| P62843 | RS15_MOUSE | Rps15 | 17.04 | 145 | 21 | 12 | 9 | 603010000 | 430280000 | 1.7E+08 | 75376000 | 53786000 | 2.2E+07 | 1.316840635 |
| Q8BVY0 | RL1D1_MOUSE | Rsl1d1 | 50.421 | 452 | 4 | 2 | 2 | 12303000 | 8766000 | 3537200 | 585870 | 417430 | 168440 | 1.309310885 |
| O54833 | CSK22_MOUSE | Csnk2a2 | 41.215 | 350 | 5 | 3 | 2 | 19591000 | 13934000 | 5657500 | 890520 | 633360 | 257160 | 1.300372884 |
| Q8BV66 | IFI44_MOUSE | Ifi44 | 47.852 | 422 | 4 | 2 | 2 | 9624600 | 6844500 | 2780100 | 370180 | 263250 | 106930 | 1.299808377 |
| P47753 | CAZA1_MOUSE | Capza1 | 32.94 | 286 | 18 | 9 | 9 | 719400000 | 510130000 | 2.1E+08 | 42317000 | 30007000 | 1.2E+07 | 1.285499438 |
| P70248 | MYO1F_MOUSE | Myo1f | 125.947 | 1099 | 1 | 1 | 0 | 2473600 | 2473600 | 0 | 39897 | 39897 | 0 | 1.284215718 |
| Q9CQU3 | RER1_MOUSE | Rer1 | 22.988 | 196 | 3 | 2 | 1 | 5628600 | 3990000 | 1638600 | 562860 | 399000 | 163860 | 1.283925027 |
| P42225 | STAT1_MOUSE | Stat1 | 87.197 | 749 | 4 | 2 | 2 | 15393000 | 10911000 | 4482500 | 366510 | 259780 | 106730 | 1.283407843 |
| Q52KI8 | SRRM1_MOUSE | Srrm1 | 106.862 | 946 | 4 | 2 | 2 | 33804000 | 23958000 | 9845500 | 626000 | 443670 | 182320 | 1.282971098 |
| P61965 | WDR5_MOUSE | Wdr5 | 36.588 | 334 | 10 | 5 | 5 | 70889000 | 50240000 | 2.1E+07 | 3082100 | 2184300 | 897790 | 1.282764548 |
| P63087 | PP1G_MOUSE | Ppp1cc | 36.984 | 323 | 23 | 11 | 12 | 360180000 | 255250000 | 1E+08 | 20010000 | 14180000 | 5829600 | 1.282483751 |
| Q6ZWY3 | RS27L_MOUSE | Rps27l | 9.477 | 84 | 2 | 1 | 1 | 50603000 | 35836000 | 1.5E+07 | 10121000 | 7167300 | 2953300 | 1.27913055 |
| Q9D136 | OGFD3_MOUSE | Ogfod3 | 35.385 | 315 | 2 | 1 | 1 | 18004000 | 12736000 | 5267400 | 1000200 | 707560 | 292630 | 1.273749316 |
| Q9D8E6 | RL4_MOUSE | Rpl4 | 47.154 | 419 | 13 | 8 | 5 | 189650000 | 134140000 | 5.6E+07 | 7585900 | 5365600 | 2220300 | 1.272997881 |
| Q62189 | SNRPA_MOUSE | Snrpa | 31.835 | 287 | 14 | 7 | 7 | 465680000 | 329290000 | 1.4E+08 | 35822000 | 25330000 | 1E+07 | 1.271620829 |
| P20152 | VIME_MOUSE | Vim | 53.688 | 466 | 69 | 36 | 33 | 4362400000 | 3082500000 | 1.3E+09 | 124640000 | 88072000 | 3.7E+07 | 1.268069799 |
| O35648 | CETN3_MOUSE | Cetn3 | 19.519 | 167 | 4 | 2 | 2 | 32813000 | 23180000 | 9632200 | 2734400 | 1931700 | 802680 | 1.266943313 |
| P27641 | XRCC5_MOUSE | Xrcc5 | 83.057 | 732 | 4 | 2 | 2 | 15584000 | 10989000 | 4594900 | 324670 | 228940 | 95726 | 1.257954738 |
| P62141 | PP1B_MOUSE | Ppp1cb | 37.187 | 327 | 5 | 3 | 2 | 35772000 | 25224000 | 1.1E+07 | 1882700 | 1327600 | 555180 | 1.257827599 |
| P15105 | GLNA_MOUSE | Glul | 42.12 | 373 | 1 | 1 | 0 | 5845100 | 5845100 | 0 | 278340 | 278340 | 0 | 1.25408118 |
| Q9DB20 | ATPO_MOUSE | Atp5po | 23.364 | 213 | 9 | 5 | 4 | 298210000 | 210090000 | 8.8E+07 | 21300000 | 15006000 | 6294100 | 1.25351521 |
| Q8VDP6 | CDIPT_MOUSE | Cdipt | 23.599 | 213 | 2 | 1 | 1 | 12118000 | 8527700 | 3590100 | 1101600 | 775240 | 326380 | 1.248132656 |
| P29037 | TBP_MOUSE | Tbp | 34.709 | 316 | 4 | 2 | 2 | 9983100 | 7021400 | 2961700 | 907560 | 638310 | 269250 | 1.245333206 |
| Q9CWX9 | DDX47_MOUSE | Ddx47 | 50.639 | 455 | 2 | 1 | 1 | 6351600 | 4465300 | 1886200 | 244290 | 171740 | 72547 | 1.243274448 |
| Q66GT5 | PTPM1_MOUSE | Ptpmt1 | 21.943 | 193 | 7 | 4 | 3 | 36890000 | 25903000 | 1.1E+07 | 2837700 | 1992500 | 845140 | 1.237321684 |
| P97450 | ATP5J_MOUSE | Atp5pf | 12.496 | 108 | 4 | 2 | 2 | 71726000 | 50353000 | 2.1E+07 | 10247000 | 7193200 | 3053300 | 1.236288311 |
| P67778 | PHB1_MOUSE | Phb1 | 29.82 | 272 | 27 | 13 | 14 | 380780000 | 267220000 | 1.1E+08 | 20041000 | 14064000 | 5977200 | 1.234446199 |
| Q9WUA3 | PFKAP_MOUSE | Pfkp | 85.455 | 784 | 29 | 15 | 14 | 213910000 | 150030000 | 6.4E+07 | 5781200 | 4054900 | 1726300 | 1.231950306 |
| Q60817 | NACA_MOUSE | Naca | 23.384 | 215 | 11 | 6 | 5 | 427420000 | 299100000 | 1.3E+08 | 71237000 | 49849000 | 2.1E+07 | 1.220881864 |
| Q9CR62 | M2OM_MOUSE | Slc25a11 | 34.155 | 314 | 18 | 10 | 8 | 173390000 | 121180000 | 5.2E+07 | 9125800 | 6377700 | 2748100 | 1.214615391 |
| Q9WTX5 | SKP1_MOUSE | Skp1 | 18.672 | 163 | 23 | 12 | 11 | 2370000000 | 1653000000 | 7.2E+08 | 197500000 | 137750000 | 6E+07 | 1.205182556 |
| Q99JX4 | EIF3M_MOUSE | Eif3m | 42.517 | 374 | 7 | 4 | 3 | 50076000 | 34919000 | 1.5E+07 | 2276200 | 1587200 | 688950 | 1.204028013 |
| Q69Z38 | PEAK1_MOUSE | Peak1 | 191.097 | 1735 | 4 | 2 | 2 | 20316000 | 14166000 | 6149900 | 201150 | 140260 | 60890 | 1.20379759 |
| Q8VEM8 | MPCP_MOUSE | Slc25a3 | 39.632 | 357 | 24 | 13 | 11 | 767430000 | 534080000 | 2.3E+08 | 38372000 | 26704000 | 1.2E+07 | 1.194498568 |
| P58252 | EF2_MOUSE | Eef2 | 95.314 | 858 | 27 | 14 | 13 | 226350000 | 157460000 | 6.9E+07 | 4270700 | 2971000 | 1299700 | 1.192744558 |
| P61979 | HNRPK_MOUSE | Hnrnpk | 50.976 | 463 | 18 | 10 | 8 | 187810000 | 130630000 | 5.7E+07 | 7223500 | 5024300 | 2199300 | 1.191878503 |
| Q9DCD2 | SYF1_MOUSE | Xab2 | 99.988 | 855 | 2 | 1 | 1 | 5011200 | 3482800 | 1528400 | 91113 | 63323 | 27790 | 1.188225466 |
| Q9R1Q6 | T176B_MOUSE | Tmem176b | 28.367 | 263 | 2 | 1 | 1 | 10915000 | 7583200 | 3332200 | 1212800 | 842580 | 370240 | 1.186331779 |
| Q91VC3 | IF4A3_MOUSE | Eif4a3 | 46.84 | 411 | 13 | 6 | 7 | 87780000 | 60949000 | 2.7E+07 | 3376200 | 2344200 | 1032000 | 1.183701725 |
| Q9D6L8 | PPIL3_MOUSE | Ppil3 | 18.128 | 161 | 4 | 2 | 2 | 22968000 | 15947000 | 7020900 | 1766800 | 1226700 | 540070 | 1.18355716 |
| Q91YT0 | NDUV1_MOUSE | Ndufv1 | 50.834 | 464 | 4 | 2 | 2 | 21942000 | 15234000 | 6708000 | 783640 | 544070 | 239570 | 1.183340207 |
| O55022 | PGRC1_MOUSE | Pgrmc1 | 21.694 | 195 | 5 | 3 | 2 | 27045000 | 18771000 | 8274600 | 2704500 | 1877100 | 827460 | 1.181744032 |
| P84078 | ARF1_MOUSE | Arf1 | 20.697 | 181 | 19 | 11 | 8 | 507760000 | 352340000 | 1.6E+08 | 46160000 | 32031000 | 1.4E+07 | 1.180796102 |
| Q9EQQ2 | YIPF5_MOUSE | Yipf5 | 27.873 | 257 | 2 | 1 | 1 | 6760400 | 4690800 | 2069600 | 3380200 | 2345400 | 1034800 | 1.180482031 |
| P62855 | RS26_MOUSE | Rps26 | 13.015 | 115 | 2 | 1 | 1 | 39207000 | 27180000 | 1.2E+07 | 6534500 | 4530000 | 2004600 | 1.176268633 |
| Q3TLH4 | PRC2C_MOUSE | Prrc2c | 310.892 | 2846 | 6 | 3 | 3 | 34738000 | 24066000 | 1.1E+07 | 267220 | 185120 | 82094 | 1.1731658 |
| P62082 | RS7_MOUSE | Rps7 | 22.127 | 194 | 25 | 13 | 12 | 1356700000 | 938970000 | 4.2E+08 | 135670000 | 93897000 | 4.2E+07 | 1.168542845 |
| Q9D0I9 | SYRC_MOUSE | Rars1 | 75.674 | 660 | 13 | 7 | 6 | 55878000 | 38646000 | 1.7E+07 | 1330400 | 920140 | 410280 | 1.165228943 |
| Q60932 | VDAC1_MOUSE | Vdac1 | 32.351 | 296 | 16 | 8 | 8 | 119150000 | 82179000 | 3.7E+07 | 6619700 | 4565500 | 2054200 | 1.152219628 |
| Q8BMB3 | IF4E2_MOUSE | Eif4e2 | 28.263 | 245 | 8 | 4 | 4 | 114230000 | 78665000 | 3.6E+07 | 8159600 | 5618900 | 2540700 | 1.145060913 |
| Q3U186 | SYRM_MOUSE | Rars2 | 65.336 | 578 | 2 | 1 | 1 | 4437200 | 3055500 | 1381700 | 113770 | 78347 | 35427 | 1.144964075 |
| Q9R061 | NUBP2_MOUSE | Nubp2 | 29.518 | 275 | 4 | 2 | 2 | 9235100 | 6350900 | 2884300 | 769600 | 529240 | 240350 | 1.138739825 |
| Q9JLV5 | CUL3_MOUSE | Cul3 | 88.948 | 768 | 4 | 2 | 2 | 7463200 | 5128800 | 2334500 | 146340 | 100560 | 45774 | 1.135507725 |
| Q8BSY0 | ASPH_MOUSE | Asph | 83.042 | 741 | 15 | 8 | 7 | 153210000 | 105230000 | 4.8E+07 | 4506100 | 3094900 | 1411200 | 1.132980862 |
| Q61656 | DDX5_MOUSE | Ddx5 | 69.29 | 614 | 37 | 19 | 18 | 369450000 | 253500000 | 1.2E+08 | 10263000 | 7041600 | 3221000 | 1.128358509 |
| P62137 | PP1A_MOUSE | Ppp1ca | 37.54 | 330 | 2 | 1 | 1 | 10709000 | 7347000 | 3361500 | 535430 | 367350 | 168080 | 1.128050125 |
| Q8BSF4 | PISD_MOUSE | Pisd | 45.927 | 406 | 2 | 1 | 1 | 7496000 | 5141500 | 2354500 | 267710 | 183620 | 84089 | 1.126768595 |
| Q9CQY6 | UQCC2_MOUSE | Uqcc2 | 16.321 | 136 | 6 | 3 | 3 | 34874000 | 23907000 | 1.1E+07 | 4982000 | 3415300 | 1566800 | 1.12426417 |
| P09405 | NUCL_MOUSE | Ncl | 76.723 | 707 | 44 | 23 | 21 | 2391100000 | 1638900000 | 7.5E+08 | 50874000 | 34870000 | 1.6E+07 | 1.123616338 |
| Q9D662 | SC23B_MOUSE | Sec23b | 86.437 | 767 | 7 | 4 | 3 | 21015000 | 14400000 | 6614800 | 525370 | 360000 | 165370 | 1.12229937 |
| Q9CQE8 | RTRAF_MOUSE | RTRAF | 28.152 | 244 | 23 | 12 | 11 | 551160000 | 377630000 | 1.7E+08 | 30620000 | 20980000 | 9640400 | 1.121788282 |
| O08788 | DCTN1_MOUSE | Dctn1 | 141.676 | 1281 | 2 | 1 | 1 | 2007100 | 1374300 | 632820 | 26761 | 18324 | 8437.5 | 1.118829867 |
| Q91VU0 | FAM3C_MOUSE | Fam3c | 24.753 | 227 | 2 | 1 | 1 | 7661300 | 5243900 | 2417300 | 510750 | 349590 | 161160 | 1.117243643 |
| P61358 | RL27_MOUSE | Rpl27 | 15.798 | 136 | 9 | 4 | 5 | 235570000 | 161210000 | 7.4E+07 | 39262000 | 26868000 | 1.2E+07 | 1.11630376 |
| Q9CY27 | TECR_MOUSE | Tecr | 36.09 | 308 | 9 | 5 | 4 | 117920000 | 80672000 | 3.7E+07 | 7370200 | 5042000 | 2328100 | 1.114827598 |
| P62806 | H4_MOUSE | H4c16 | 11.367 | 103 | 9 | 4 | 5 | 100880000 | 68990000 | 3.2E+07 | 16813000 | 11498000 | 5314300 | 1.113464133 |
| Q9CPS7 | PNO1_MOUSE | Pno1 | 27.454 | 248 | 6 | 3 | 3 | 30360000 | 20751000 | 9608000 | 2024000 | 1383400 | 640530 | 1.110872806 |
| Q08288 | LYAR_MOUSE | Lyar | 43.736 | 388 | 4 | 2 | 2 | 27310000 | 18665000 | 8644800 | 1300500 | 888830 | 411660 | 1.110431016 |
| Q8BMZ5 | SEN34_MOUSE | Tsen34 | 34.196 | 316 | 2 | 1 | 1 | 7237300 | 4937600 | 2299700 | 380910 | 259880 | 121040 | 1.102364296 |
| Q62261 | SPTB2_MOUSE | Sptbn1 | 274.223 | 2363 | 4 | 2 | 2 | 8086700 | 5516400 | 2570400 | 49612 | 33843 | 15769 | 1.101734186 |
| Q9D074 | MGRN1_MOUSE | Mgrn1 | 58.477 | 532 | 7 | 4 | 3 | 41944000 | 28602000 | 1.3E+07 | 1613200 | 1100100 | 513160 | 1.100141085 |
| P97783 | AF1Q_MOUSE | Mllt11 | 10.029 | 90 | 2 | 1 | 1 | 6194300 | 4223800 | 1970400 | 1032400 | 703970 | 328400 | 1.100052991 |
| Q62318 | TIF1B_MOUSE | Trim28 | 88.847 | 834 | 26 | 12 | 14 | 435140000 | 296570000 | 1.4E+08 | 11761000 | 8015400 | 3745100 | 1.097757715 |
| P56959 | FUS_MOUSE | Fus | 52.673 | 518 | 17 | 9 | 8 | 502450000 | 342220000 | 1.6E+08 | 29556000 | 20131000 | 9424900 | 1.094869828 |
| Q9D6N5 | NC2A_MOUSE | Drap1 | 22.278 | 205 | 3 | 2 | 1 | 20729000 | 14110000 | 6618700 | 2303200 | 1567800 | 735410 | 1.092098202 |
| Q8BXZ1 | TMX3_MOUSE | Tmx3 | 51.848 | 456 | 11 | 6 | 5 | 67494000 | 45897000 | 2.2E+07 | 2595900 | 1765300 | 830670 | 1.087568933 |
| Q8R570 | SNP47_MOUSE | Snap47 | 46.524 | 413 | 6 | 3 | 3 | 11690000 | 7914700 | 3775300 | 377100 | 255310 | 121780 | 1.067943376 |
| P97479 | MYO7A_MOUSE | Myo7a | 254.939 | 2215 | 2 | 1 | 1 | 2137200 | 1446000 | 691180 | 16697 | 11297 | 5399.8 | 1.064934175 |
| O35207 | CDKA1_MOUSE | Cdk2ap1 | 12.354 | 114 | 2 | 1 | 1 | 3654000 | 2472200 | 1181800 | 730800 | 494450 | 236350 | 1.064809557 |
| Q9CX86 | ROA0_MOUSE | Hnrnpa0 | 30.53 | 305 | 19 | 9 | 10 | 387730000 | 262080000 | 1.3E+08 | 25849000 | 17472000 | 8376400 | 1.060596591 |
| P61255 | RL26_MOUSE | Rpl26 | 17.258 | 145 | 11 | 5 | 6 | 214810000 | 145100000 | 7E+07 | 23868000 | 16123000 | 7745400 | 1.057630682 |
| Q9D1D4 | TMEDA_MOUSE | Tmed10 | 24.911 | 219 | 13 | 7 | 6 | 109510000 | 73971000 | 3.6E+07 | 9955600 | 6724700 | 3230900 | 1.057516098 |
| P07091 | S10A4_MOUSE | S100a4 | 11.721 | 101 | 7 | 3 | 4 | 170220000 | 114930000 | 5.5E+07 | 34043000 | 22987000 | 1.1E+07 | 1.055847619 |
| Q01853 | TERA_MOUSE | Vcp | 89.322 | 806 | 18 | 9 | 9 | 140830000 | 94995000 | 4.6E+07 | 2934000 | 1979100 | 954950 | 1.051338956 |
| P10810 | CD14_MOUSE | Cd14 | 39.204 | 366 | 9 | 4 | 5 | 40144000 | 27071000 | 1.3E+07 | 2361400 | 1592400 | 768970 | 1.050268293 |
| Q9DAM7 | TM263_MOUSE | Tmem263 | 11.549 | 115 | 2 | 1 | 1 | 8999600 | 6066400 | 2933200 | 999960 | 674050 | 325910 | 1.048365184 |
| Q91Z69 | SRGP1_MOUSE | Srgap1 | 121.43 | 1062 | 2 | 1 | 1 | 6188600 | 4167800 | 2020800 | 98232 | 66155 | 32077 | 1.044359505 |
| Q9Z210 | PX11B_MOUSE | Pex11b | 28.71 | 259 | 5 | 3 | 2 | 25071000 | 16854000 | 8216500 | 1474800 | 991430 | 483330 | 1.036495148 |
| P01897 | HA1L_MOUSE | H2-L | 40.711 | 362 | 6 | 3 | 3 | 20156000 | 13550000 | 6606100 | 876340 | 589120 | 287220 | 1.036422138 |
| Q9JHU4 | DYHC1_MOUSE | Dync1h1 | 532.045 | 4644 | 14 | 7 | 7 | 41547000 | 27917000 | 1.4E+07 | 144760 | 97270 | 47494 | 1.034252511 |
| P62751 | RL23A_MOUSE | Rpl23a | 17.695 | 156 | 16 | 9 | 7 | 766490000 | 514990000 | 2.5E+08 | 85166000 | 57222000 | 2.8E+07 | 1.033986018 |
| P62259 | 1433E_MOUSE | Ywhae | 29.174 | 255 | 22 | 12 | 10 | 644980000 | 433330000 | 2.1E+08 | 35832000 | 24074000 | 1.2E+07 | 1.033785631 |
| Q99KP6 | PRP19_MOUSE | Prpf19 | 55.239 | 504 | 9 | 5 | 4 | 70861000 | 47601000 | 2.3E+07 | 3221000 | 2163700 | 1057300 | 1.033078762 |
| Q9CXL3 | CG050_MOUSE |  | 22.168 | 195 | 4 | 2 | 2 | 51494000 | 34590000 | 1.7E+07 | 4291100 | 2882500 | 1408600 | 1.03307569 |
| Q99JY9 | ARP3_MOUSE | Actr3 | 47.357 | 418 | 29 | 15 | 14 | 611060000 | 410450000 | 2E+08 | 27776000 | 18657000 | 9118900 | 1.03274105 |
| Q8BFR5 | EFTU_MOUSE | Tufm | 49.508 | 452 | 31 | 16 | 15 | 463000000 | 310920000 | 1.5E+08 | 14469000 | 9716300 | 4752300 | 1.031712984 |
| Q8BW10 | NOB1_MOUSE | Nob1 | 45.464 | 403 | 5 | 3 | 2 | 15909000 | 10681000 | 5228200 | 723160 | 485510 | 237650 | 1.030660488 |
| P62889 | RL30_MOUSE | Rpl30 | 12.784 | 115 | 11 | 6 | 5 | 235080000 | 157800000 | 7.7E+07 | 29384000 | 19725000 | 9659200 | 1.030060891 |
| Q925I1 | ATAD3_MOUSE | Atad3 | 66.742 | 591 | 20 | 9 | 11 | 192170000 | 128970000 | 6.3E+07 | 4804300 | 3224200 | 1580000 | 1.029016225 |
| Q9CXY9 | GPI8_MOUSE | Pigk | 44.895 | 395 | 2 | 1 | 1 | 7939600 | 5327300 | 2612200 | 345200 | 231620 | 113580 | 1.028139167 |
| Q9D6V8 | PAIP2_MOUSE | Paip2 | 14.7 | 124 | 2 | 1 | 1 | 12778000 | 8569200 | 4208400 | 2555500 | 1713800 | 841680 | 1.025888688 |
| P18155 | MTDC_MOUSE | Mthfd2 | 37.863 | 350 | 3 | 2 | 1 | 16688000 | 11190000 | 5498100 | 695350 | 466270 | 229090 | 1.025204984 |
| Q99JY0 | ECHB_MOUSE | Hadhb | 51.386 | 475 | 27 | 14 | 13 | 620930000 | 415930000 | 2.1E+08 | 22176000 | 14855000 | 7321500 | 1.020716837 |
| P62242 | RS8_MOUSE | Rps8 | 24.205 | 208 | 8 | 4 | 4 | 106450000 | 71284000 | 3.5E+07 | 9677300 | 6480400 | 3196900 | 1.019438077 |
| P11031 | TCP4_MOUSE | Sub1 | 14.427 | 127 | 13 | 7 | 6 | 169760000 | 113560000 | 5.6E+07 | 16976000 | 11356000 | 5620000 | 1.014812719 |
| Q8BJ03 | COX15_MOUSE | Cox15 | 45.853 | 413 | 8 | 4 | 4 | 36792000 | 24596000 | 1.2E+07 | 1752000 | 1171200 | 580760 | 1.012015656 |
| Q3UMR5 | MCU_MOUSE | Mcu | 39.682 | 350 | 2 | 1 | 1 | 7355400 | 4916500 | 2439000 | 408640 | 273140 | 135500 | 1.011341885 |
| Q5F2E7 | NUFP2_MOUSE | Nufip2 | 75.657 | 692 | 4 | 2 | 2 | 28512000 | 19020000 | 9491900 | 950410 | 634010 | 316400 | 1.00274844 |
| Q8VH51 | RBM39_MOUSE | Rbm39 | 59.407 | 530 | 4 | 2 | 2 | 9126400 | 6084800 | 3041600 | 351010 | 234030 | 116980 | 1.000379407 |
| P51410 | RL9_MOUSE | Rpl9 | 21.881 | 192 | 19 | 10 | 9 | 719480000 | 479080000 | 2.4E+08 | 65407000 | 43553000 | 2.2E+07 | 0.994829691 |
| P54823 | DDX6_MOUSE | Ddx6 | 54.192 | 483 | 10 | 5 | 5 | 49517000 | 32950000 | 1.7E+07 | 1500500 | 998490 | 502020 | 0.991966086 |
| O88569 | ROA2_MOUSE | Hnrnpa2b1 | 37.403 | 353 | 17 | 9 | 8 | 303460000 | 201690000 | 1E+08 | 15173000 | 10084000 | 5088500 | 0.986827212 |
| Q9D1G1 | RAB1B_MOUSE | Rab1b | 22.187 | 201 | 4 | 2 | 2 | 27525000 | 18293000 | 9232000 | 1529200 | 1016300 | 512890 | 0.986576563 |
| Q8K2P7 | S38A1_MOUSE | Slc38a1 | 53.795 | 485 | 2 | 1 | 1 | 7678000 | 5099900 | 2578100 | 404110 | 268410 | 135690 | 0.984160734 |
| Q9ESP1 | SDF2L_MOUSE | Sdf2l1 | 23.648 | 221 | 15 | 7 | 8 | 364880000 | 242190000 | 1.2E+08 | 24325000 | 16146000 | 8179300 | 0.981121632 |
| P69566 | RANB9_MOUSE | Ranbp9 | 71.012 | 653 | 5 | 3 | 2 | 17451000 | 11583000 | 5868800 | 623260 | 413660 | 209600 | 0.980871511 |
| Q62426 | CYTB_MOUSE | Cstb | 11.046 | 98 | 2 | 1 | 1 | 16109000 | 10687000 | 5421600 | 2301300 | 1526800 | 774510 | 0.979066342 |
| Q9ERS2 | NDUAD_MOUSE | Ndufa13 | 16.86 | 144 | 11 | 6 | 5 | 152520000 | 101160000 | 5.1E+07 | 15252000 | 10116000 | 5135400 | 0.978090384 |
| P68040 | RACK1_MOUSE | Rack1 | 35.077 | 317 | 28 | 14 | 14 | 480660000 | 318790000 | 1.6E+08 | 21848000 | 14491000 | 7357600 | 0.977770745 |
| Q8BHE8 | MAIP1_MOUSE | Maip1 | 32.985 | 291 | 2 | 1 | 1 | 8606800 | 5706500 | 2900300 | 374210 | 248110 | 126100 | 0.976404024 |
| Q8BXQ2 | PIGT_MOUSE | Pigt | 65.705 | 582 | 4 | 2 | 2 | 9057000 | 5993700 | 3063400 | 266380 | 176280 | 90099 | 0.968313118 |
| P61750 | ARF4_MOUSE | Arf4 | 20.397 | 180 | 4 | 2 | 2 | 65467000 | 43322000 | 2.2E+07 | 5951600 | 3938300 | 2013200 | 0.968118851 |
| Q6P5B0 | RRP12_MOUSE | Rrp12 | 143.131 | 1295 | 4 | 2 | 2 | 6107100 | 4037600 | 2069500 | 80357 | 53127 | 27230 | 0.964215743 |
| P50518 | VATE1_MOUSE | Atp6v1e1 | 26.157 | 226 | 4 | 2 | 2 | 24135000 | 15956000 | 8179700 | 1508500 | 997230 | 511230 | 0.963979192 |
| Q9ERR7 | SEP15_MOUSE | Selenof | 17.806 | 162 | 4 | 2 | 2 | 60846000 | 40150000 | 2.1E+07 | 6084600 | 4015000 | 2069600 | 0.956048029 |
| P63168 | DYL1_MOUSE | Dynll1 | 10.366 | 89 | 3 | 2 | 1 | 84492000 | 55703000 | 2.9E+07 | 16898000 | 11141000 | 5757800 | 0.952237352 |
| P62858 | RS28_MOUSE | Rps28 | 7.841 | 69 | 4 | 2 | 2 | 351660000 | 231650000 | 1.2E+08 | 117220000 | 77217000 | 4E+07 | 0.948792057 |
| Q91W90 | TXND5_MOUSE | Txndc5 | 46.415 | 417 | 14 | 7 | 7 | 121680000 | 80151000 | 4.2E+07 | 5530900 | 3643200 | 1887700 | 0.948601387 |
| Q9JHR7 | IDE_MOUSE | Ide | 117.772 | 1019 | 2 | 1 | 1 | 5765700 | 3794400 | 1971300 | 96096 | 63240 | 32855 | 0.944724426 |
| P45878 | FKBP2_MOUSE | Fkbp2 | 15.344 | 140 | 4 | 2 | 2 | 79280000 | 52053000 | 2.7E+07 | 9910000 | 6506600 | 3403300 | 0.934996274 |
| Q9Z0V8 | TI17A_MOUSE | Timm17a | 18.112 | 171 | 2 | 1 | 1 | 11309000 | 7425100 | 3884300 | 1413700 | 928140 | 485540 | 0.934755826 |
| Q8BK72 | RT27_MOUSE | Mrps27 | 47.779 | 415 | 9 | 5 | 4 | 60669000 | 39831000 | 2.1E+07 | 2247000 | 1475200 | 771790 | 0.934674884 |
| Q9CQY5 | MAGT1_MOUSE | Magt1 | 37.97 | 335 | 10 | 5 | 5 | 98794000 | 64804000 | 3.4E+07 | 5811400 | 3812000 | 1999400 | 0.930972504 |
| O09005 | DEGS1_MOUSE | Degs1 | 38.241 | 323 | 4 | 2 | 2 | 27806000 | 18237000 | 9569100 | 2139000 | 1402900 | 736080 | 0.930413278 |
| Q9D4H1 | EXOC2_MOUSE | Exoc2 | 103.959 | 924 | 2 | 1 | 1 | 3678400 | 2412400 | 1266000 | 69403 | 45517 | 23886 | 0.930191736 |
| Q8CIG8 | ANM5_MOUSE | Prmt5 | 72.68 | 637 | 12 | 6 | 6 | 74667000 | 48932000 | 2.6E+07 | 1821100 | 1193500 | 627670 | 0.927046469 |
| Q9CYZ6 | REX1B_MOUSE | Rex1bd | 18.706 | 169 | 1 | 1 | 0 | 3032000 | 3032000 | 0 | 252660 | 252660 | 0 | 0.925217912 |
| O88986 | KBL_MOUSE | Gcat | 44.931 | 416 | 38 | 20 | 18 | 913950000 | 598660000 | 3.2E+08 | 45697000 | 29933000 | 1.6E+07 | 0.925057465 |
| Q9QXX4 | S2513_MOUSE | Slc25a13 | 74.467 | 676 | 19 | 9 | 10 | 112600000 | 73719000 | 3.9E+07 | 2746300 | 1798000 | 948270 | 0.923045389 |
| P84099 | RL19_MOUSE | Rpl19 | 23.466 | 196 | 3 | 2 | 1 | 20148000 | 13187000 | 6960700 | 2238700 | 1465300 | 773410 | 0.921812091 |
| Q9JKB3 | YBOX3_MOUSE | Ybx3 | 38.814 | 361 | 6 | 3 | 3 | 254640000 | 166670000 | 8.8E+07 | 18189000 | 11905000 | 6284100 | 0.921796136 |
| Q924C1 | XPO5_MOUSE | Xpo5 | 136.973 | 1204 | 2 | 1 | 1 | 8159700 | 5339800 | 2819900 | 121790 | 79698 | 42089 | 0.921141705 |
| P60335 | PCBP1_MOUSE | Pcbp1 | 37.498 | 356 | 10 | 5 | 5 | 252380000 | 164900000 | 8.7E+07 | 12018000 | 7852200 | 4166100 | 0.914417855 |
| O09159 | MA2B1_MOUSE | Man2b1 | 114.648 | 1013 | 4 | 2 | 2 | 19358000 | 12622000 | 6735800 | 395060 | 257590 | 137460 | 0.906019321 |
| Q9DBZ5 | EIF3K_MOUSE | Eif3k | 25.087 | 218 | 5 | 2 | 3 | 71409000 | 46559000 | 2.5E+07 | 6491700 | 4232600 | 2259100 | 0.90581422 |
| P47856 | GFPT1_MOUSE | Gfpt1 | 78.539 | 697 | 4 | 2 | 2 | 21957000 | 14308000 | 7649400 | 467170 | 304420 | 162750 | 0.903403528 |
| P19536 | COX5B_MOUSE | Cox5b | 13.813 | 128 | 7 | 3 | 4 | 52484000 | 34114000 | 1.8E+07 | 5248400 | 3411400 | 1837000 | 0.8930123 |
| Q9DCJ5 | NDUA8_MOUSE | Ndufa8 | 19.992 | 172 | 8 | 4 | 4 | 91844000 | 59697000 | 3.2E+07 | 8349500 | 5427000 | 2922400 | 0.892974323 |
| O08553 | DPYL2_MOUSE | Dpysl2 | 62.278 | 572 | 3 | 2 | 1 | 7824600 | 5082400 | 2742200 | 230140 | 149480 | 80654 | 0.890176128 |
| O55135 | IF6_MOUSE | Eif6 | 26.511 | 245 | 5 | 3 | 2 | 20024000 | 13002000 | 7022300 | 2002400 | 1300200 | 702230 | 0.888718023 |
| P49312 | ROA1_MOUSE | Hnrnpa1 | 34.196 | 320 | 22 | 11 | 11 | 257380000 | 167010000 | 9E+07 | 13546000 | 8790200 | 4756000 | 0.88611445 |
| Q8VEJ9 | VPS4A_MOUSE | Vps4a | 48.907 | 437 | 2 | 1 | 1 | 6635500 | 4300300 | 2335200 | 228810 | 148290 | 80524 | 0.880891193 |
| Q99MN1 | SYK_MOUSE | Kars1 | 67.84 | 595 | 2 | 1 | 1 | 7294600 | 4726300 | 2568300 | 227960 | 147700 | 80260 | 0.879897475 |
| Q9Z1B5 | MD2L1_MOUSE | Mad2l1 | 23.598 | 205 | 4 | 2 | 2 | 57986000 | 37562000 | 2E+07 | 4141800 | 2683000 | 1458800 | 0.879008439 |
| P70372 | ELAV1_MOUSE | Elavl1 | 36.169 | 326 | 6 | 3 | 3 | 22579000 | 14619000 | 7959600 | 1254400 | 812170 | 442200 | 0.877076792 |
| Q9ESJ0 | XPO4_MOUSE | Xpo4 | 129.979 | 1151 | 2 | 1 | 1 | 2818300 | 1822700 | 995550 | 46971 | 30379 | 16592 | 0.872511446 |
| Q9CQA1 | TPPC5_MOUSE | Trappc5 | 20.795 | 188 | 4 | 2 | 2 | 14149000 | 9122500 | 5026100 | 1088300 | 701730 | 386620 | 0.85998987 |
| P51807 | DYLT1_MOUSE | Dynlt1 | 12.483 | 113 | 2 | 1 | 1 | 21700000 | 13990000 | 7709600 | 3616700 | 2331700 | 1284900 | 0.859668047 |
| P09925 | SURF1_MOUSE | Surf1 | 34.798 | 306 | 2 | 1 | 1 | 5663300 | 3648100 | 2015200 | 283160 | 182400 | 100760 | 0.856222249 |
| P62849 | RS24_MOUSE | Rps24 | 15.423 | 133 | 16 | 8 | 8 | 1235400000 | 795600000 | 4.4E+08 | 176480000 | 113660000 | 6.3E+07 | 0.855294086 |
| Q61263 | SOAT1_MOUSE | Soat1 | 63.799 | 540 | 14 | 8 | 6 | 162630000 | 104710000 | 5.8E+07 | 6505400 | 4188300 | 2317000 | 0.854116279 |
| Q9DBS1 | TMM43_MOUSE | Tmem43 | 44.783 | 400 | 5 | 3 | 2 | 19994000 | 12870000 | 7123400 | 869290 | 559580 | 309710 | 0.853374144 |
| Q9D902 | T2EB_MOUSE | Gtf2e2 | 33.047 | 292 | 2 | 1 | 1 | 6318000 | 4066100 | 2251800 | 287180 | 184820 | 102360 | 0.852567001 |
| Q9CZU4 | ERAL1_MOUSE | Eral1 | 48.187 | 437 | 4 | 2 | 2 | 4451400 | 2863700 | 1587700 | 178060 | 114550 | 63509 | 0.850942027 |
| Q8VBT0 | TMX1_MOUSE | Tmx1 | 31.396 | 278 | 2 | 1 | 1 | 6434600 | 4134300 | 2300200 | 584960 | 375850 | 209110 | 0.845883772 |
| Q9DB15 | RM12_MOUSE | Mrpl12 | 21.708 | 201 | 2 | 1 | 1 | 41242000 | 26498000 | 1.5E+07 | 4582400 | 2944300 | 1638200 | 0.84585335 |
| Q07797 | LG3BP_MOUSE | Lgals3bp | 64.491 | 577 | 4 | 2 | 2 | 38622000 | 24785000 | 1.4E+07 | 1485400 | 953260 | 532190 | 0.840936073 |
| P27773 | PDIA3_MOUSE | Pdia3 | 56.678 | 505 | 33 | 16 | 17 | 440940000 | 282830000 | 1.6E+08 | 12248000 | 7856400 | 4391900 | 0.839006539 |
| Q9DC69 | NDUA9_MOUSE | Ndufa9 | 42.525 | 377 | 18 | 10 | 8 | 166210000 | 106530000 | 6E+07 | 5731200 | 3673600 | 2057700 | 0.836133729 |
| P61027 | RAB10_MOUSE | Rab10 | 22.541 | 200 | 2 | 1 | 1 | 10301000 | 6593000 | 3707800 | 605930 | 387820 | 218110 | 0.830371655 |
| P17439 | GBA1_MOUSE | Gba1 | 57.622 | 515 | 13 | 7 | 6 | 73892000 | 47266000 | 2.7E+07 | 3078800 | 1969400 | 1109400 | 0.828021253 |
| P54987 | IRG1_MOUSE | Acod1 | 53.758 | 488 | 19 | 9 | 10 | 271210000 | 173420000 | 9.8E+07 | 10431000 | 6669800 | 3761400 | 0.826393423 |
| P62911 | RL32_MOUSE | Rpl32 | 15.86 | 135 | 3 | 2 | 1 | 13219000 | 8452000 | 4766800 | 1888400 | 1207400 | 680970 | 0.826271672 |
| P26369 | U2AF2_MOUSE | U2af2 | 53.517 | 475 | 11 | 5 | 6 | 100300000 | 64131000 | 3.6E+07 | 5279000 | 3375300 | 1903700 | 0.826228305 |
| P61028 | RAB8B_MOUSE | Rab8b | 23.603 | 207 | 2 | 1 | 1 | 9343800 | 5972000 | 3371800 | 583980 | 373250 | 210740 | 0.824695201 |
| Q8C2Q3 | RBM14_MOUSE | Rbm14 | 69.449 | 669 | 4 | 2 | 2 | 21205000 | 13550000 | 7655000 | 815590 | 521170 | 294420 | 0.823818569 |
| Q8BG32 | PSD11_MOUSE | Psmd11 | 47.437 | 422 | 20 | 10 | 10 | 192020000 | 122670000 | 6.9E+07 | 6000600 | 3833400 | 2167100 | 0.822856288 |
| Q9DCT2 | NDUS3_MOUSE | Ndufs3 | 30.149 | 263 | 12 | 6 | 6 | 99699000 | 63612000 | 3.6E+07 | 5247300 | 3348000 | 1899300 | 0.817819733 |
| P62301 | RS13_MOUSE | Rps13 | 17.222 | 151 | 12 | 6 | 6 | 389280000 | 248320000 | 1.4E+08 | 35389000 | 22575000 | 1.3E+07 | 0.816914633 |
| Q62086 | PON2_MOUSE | Pon2 | 39.617 | 354 | 2 | 1 | 1 | 8607600 | 5490500 | 3117100 | 506330 | 322970 | 183360 | 0.816733097 |
| Q05186 | RCN1_MOUSE | Rcn1 | 38.113 | 325 | 2 | 1 | 1 | 6133900 | 3912000 | 2221800 | 383370 | 244500 | 138870 | 0.816177415 |
| P97363 | SPTC2_MOUSE | Sptlc2 | 62.982 | 560 | 7 | 3 | 4 | 32522000 | 20740000 | 1.2E+07 | 903400 | 576120 | 327280 | 0.815831436 |
| P57716 | NICA_MOUSE | Ncstn | 78.492 | 708 | 6 | 3 | 3 | 20828000 | 13281000 | 7547200 | 562930 | 358950 | 203980 | 0.815350368 |
| Q60930 | VDAC2_MOUSE | Vdac2 | 31.733 | 295 | 13 | 6 | 7 | 154760000 | 98659000 | 5.6E+07 | 9103600 | 5803500 | 3300100 | 0.814424177 |
| P63242 | IF5A1_MOUSE | Eif5a | 16.832 | 154 | 16 | 8 | 8 | 557060000 | 355120000 | 2E+08 | 69633000 | 44391000 | 2.5E+07 | 0.814379908 |
| P51881 | ADT2_MOUSE | Slc25a5 | 32.931 | 298 | 42 | 22 | 20 | 4739500000 | 3018500000 | 1.7E+09 | 225690000 | 143740000 | 8.2E+07 | 0.810584704 |
| Q8K5B2 | MCFD2_MOUSE | Mcfd2 | 16.168 | 145 | 5 | 3 | 2 | 41932000 | 26705000 | 1.5E+07 | 6988700 | 4450900 | 2537800 | 0.810478152 |
| P63276 | RS17_MOUSE | Rps17 | 15.524 | 135 | 20 | 10 | 10 | 672900000 | 428380000 | 2.4E+08 | 112150000 | 71396000 | 4.1E+07 | 0.808938654 |
| P10605 | CATB_MOUSE | Ctsb | 37.28 | 339 | 25 | 13 | 12 | 411320000 | 261730000 | 1.5E+08 | 21649000 | 13775000 | 7873500 | 0.806969123 |
| P99029 | PRDX5_MOUSE | Prdx5 | 21.897 | 210 | 7 | 3 | 4 | 36719000 | 23363000 | 1.3E+07 | 2448000 | 1557500 | 890420 | 0.806737541 |
| Q9CRT8 | XPOT_MOUSE | Xpot | 109.734 | 963 | 4 | 2 | 2 | 11622000 | 7394400 | 4227300 | 264130 | 168050 | 96074 | 0.806696588 |
| Q8BXV2 | BRI3B_MOUSE | Bri3bp | 28.263 | 253 | 2 | 1 | 1 | 75160000 | 47817000 | 2.7E+07 | 7516000 | 4781700 | 2734400 | 0.806299317 |
| O88597 | BECN1_MOUSE | Becn1 | 51.589 | 448 | 2 | 1 | 1 | 7012700 | 4461400 | 2551400 | 241820 | 153840 | 87978 | 0.806207405 |
| P11928 | OAS1A_MOUSE | Oas1a | 42.429 | 367 | 14 | 7 | 7 | 156310000 | 99380000 | 5.7E+07 | 8684000 | 5521100 | 3162900 | 0.80371576 |
| P61089 | UBE2N_MOUSE | Ube2n | 17.138 | 152 | 5 | 3 | 2 | 29869000 | 18989000 | 1.1E+07 | 2715400 | 1726300 | 989060 | 0.803485376 |
| Q78IK2 | ATPMK_MOUSE | Atp5mk | 6.382 | 58 | 3 | 1 | 2 | 152870000 | 97124000 | 5.6E+07 | 50958000 | 32375000 | 1.9E+07 | 0.800856034 |
| Q61990 | PCBP2_MOUSE | Pcbp2 | 38.222 | 362 | 6 | 3 | 3 | 113320000 | 71969000 | 4.1E+07 | 5666000 | 3598500 | 2067600 | 0.799453394 |
| Q91ZE0 | TMLH_MOUSE | Tmlhe | 49.61 | 421 | 2 | 1 | 1 | 6952400 | 4415300 | 2537100 | 217260 | 137980 | 79285 | 0.799331081 |
| Q60668 | HNRPD_MOUSE | Hnrnpd | 38.354 | 355 | 18 | 9 | 9 | 380490000 | 241610000 | 1.4E+08 | 25366000 | 16107000 | 9258300 | 0.798841315 |
| Q3U1J4 | DDB1_MOUSE | Ddb1 | 126.853 | 1140 | 66 | 33 | 33 | 739870000 | 469540000 | 2.7E+08 | 12756000 | 8095500 | 4660900 | 0.796526441 |
| P62702 | RS4X_MOUSE | Rps4x | 29.598 | 263 | 17 | 9 | 8 | 420700000 | 266900000 | 1.5E+08 | 20033000 | 12710000 | 7323800 | 0.795243802 |
| Q61937 | NPM_MOUSE | Npm1 | 32.56 | 292 | 21 | 11 | 10 | 454460000 | 288210000 | 1.7E+08 | 28404000 | 18013000 | 1E+07 | 0.793766053 |
| O70133 | DHX9_MOUSE | Dhx9 | 149.475 | 1380 | 4 | 2 | 2 | 6552000 | 4152500 | 2399600 | 86211 | 54638 | 31573 | 0.791186232 |
| Q6PDI5 | ECM29_MOUSE | Ecpas | 203.703 | 1840 | 2 | 1 | 1 | 4838800 | 3064800 | 1774100 | 43593 | 27610 | 15983 | 0.788705599 |
| P97855 | G3BP1_MOUSE | G3bp1 | 51.829 | 465 | 46 | 24 | 22 | 1335700000 | 845320000 | 4.9E+08 | 49472000 | 31308000 | 1.8E+07 | 0.785450351 |
| O89017 | LGMN_MOUSE | Lgmn | 49.373 | 435 | 2 | 1 | 1 | 9814000 | 6210200 | 3603800 | 446090 | 282280 | 163810 | 0.785120783 |
| Q99N84 | RT18B_MOUSE | Mrps18b | 28.703 | 254 | 6 | 3 | 3 | 46639000 | 29510000 | 1.7E+07 | 3886600 | 2459200 | 1427300 | 0.78484722 |
| Q62241 | RU1C_MOUSE | Snrpc | 17.364 | 159 | 4 | 3 | 1 | 104310000 | 65998000 | 3.8E+07 | 14902000 | 9428400 | 5473400 | 0.784550654 |
| Q3UPL0 | SC31A_MOUSE | Sec31a | 133.569 | 1230 | 2 | 1 | 1 | 9318500 | 5895600 | 3422900 | 169430 | 107190 | 62234 | 0.7844195 |
| P61161 | ARP2_MOUSE | Actr2 | 44.761 | 394 | 28 | 14 | 14 | 678860000 | 429140000 | 2.5E+08 | 32326000 | 20435000 | 1.2E+07 | 0.78113701 |
| O70370 | CATS_MOUSE | Ctss | 38.475 | 340 | 8 | 4 | 4 | 111170000 | 70255000 | 4.1E+07 | 6176000 | 3903100 | 2272900 | 0.780076839 |
| Q9Z1D1 | EIF3G_MOUSE | Eif3g | 35.638 | 320 | 8 | 4 | 4 | 27316000 | 17260000 | 1E+07 | 1241700 | 784540 | 457110 | 0.77937591 |
| Q9WVA4 | TAGL2_MOUSE | Tagln2 | 22.395 | 199 | 7 | 4 | 3 | 21443000 | 13546000 | 7896600 | 1531600 | 967590 | 564040 | 0.778563383 |
| Q6P5E4 | UGGG1_MOUSE | Uggt1 | 176.434 | 1551 | 49 | 25 | 24 | 528080000 | 333470000 | 1.9E+08 | 6140500 | 3877600 | 2263000 | 0.776971133 |
| Q9WUM3 | COR1B_MOUSE | Coro1b | 53.912 | 484 | 2 | 1 | 1 | 17181000 | 10842000 | 6339000 | 747000 | 471390 | 275610 | 0.774303739 |
| Q9R1Q9 | VAS1_MOUSE | Atp6ap1 | 51.008 | 463 | 2 | 1 | 1 | 7474600 | 4716600 | 2758000 | 498310 | 314440 | 183870 | 0.774124799 |
| Q8BMS1 | ECHA_MOUSE | Hadha | 82.67 | 763 | 49 | 24 | 25 | 1193300000 | 752530000 | 4.4E+08 | 28413000 | 17917000 | 1E+07 | 0.771559416 |
| Q9D0R2 | SYTC_MOUSE | Tars1 | 83.356 | 722 | 2 | 1 | 1 | 2245900 | 1415900 | 829990 | 46790 | 29499 | 17292 | 0.770553517 |
| P63323 | RS12_MOUSE | Rps12 | 14.515 | 132 | 18 | 9 | 9 | 661400000 | 416950000 | 2.4E+08 | 94486000 | 59565000 | 3.5E+07 | 0.770334983 |
| Q9D0G0 | RT30_MOUSE | Mrps30 | 49.939 | 442 | 3 | 2 | 1 | 18151000 | 11417000 | 6734200 | 672270 | 422860 | 249420 | 0.761605135 |
| Q8CH72 | TRI32_MOUSE | Trim32 | 72.057 | 655 | 10 | 5 | 5 | 66237000 | 41656000 | 2.5E+07 | 1655900 | 1041400 | 614530 | 0.760980703 |
| Q9WTQ8 | TIM23_MOUSE | Timm23 | 21.978 | 209 | 2 | 1 | 1 | 27295000 | 17142000 | 1E+07 | 2729500 | 1714200 | 1015400 | 0.755487277 |
| Q3UBX0 | TM109_MOUSE | Tmem109 | 26.306 | 243 | 2 | 1 | 1 | 19201000 | 12056000 | 7145400 | 1920100 | 1205600 | 714540 | 0.754664641 |
| Q9Z2I9 | SUCB1_MOUSE | Sucla2 | 50.114 | 463 | 6 | 3 | 3 | 32163000 | 20146000 | 1.2E+07 | 1037500 | 649880 | 387630 | 0.745536701 |
| Q99JR1 | SFXN1_MOUSE | Sfxn1 | 35.649 | 322 | 3 | 2 | 1 | 18030000 | 11292000 | 6737700 | 1001700 | 627330 | 374320 | 0.744972936 |
| Q8VCL2 | SCO2_MOUSE | Sco2 | 28.944 | 255 | 13 | 6 | 7 | 246320000 | 153880000 | 9.2E+07 | 15395000 | 9617200 | 5777800 | 0.735138538 |
| P61620 | S61A1_MOUSE | Sec61a1 | 52.265 | 476 | 4 | 2 | 2 | 50650000 | 31573000 | 1.9E+07 | 2813900 | 1754100 | 1059800 | 0.726857034 |
| Q9WVM1 | RGAP1_MOUSE | Racgap1 | 70.158 | 628 | 2 | 1 | 1 | 6221200 | 3877300 | 2343900 | 159520 | 99417 | 60100 | 0.726141346 |
| P17918 | PCNA_MOUSE | Pcna | 28.785 | 261 | 19 | 10 | 9 | 297790000 | 185400000 | 1.1E+08 | 16544000 | 10300000 | 6243800 | 0.722127568 |
| Q99JI6 | RAP1B_MOUSE | Rap1b | 20.825 | 184 | 2 | 1 | 1 | 15593000 | 9702700 | 5890100 | 1299400 | 808560 | 490840 | 0.720094139 |
| Q9D1R9 | RL34_MOUSE | Rpl34 | 13.293 | 117 | 2 | 1 | 1 | 74459000 | 46319000 | 2.8E+07 | 12410000 | 7719800 | 4690100 | 0.718930511 |
| Q9D1H6 | NDUF4_MOUSE | Ndufaf4 | 20.082 | 173 | 4 | 2 | 2 | 18975000 | 11785000 | 7190000 | 1265000 | 785670 | 479330 | 0.712888083 |
| Q91WK0 | LRRF2_MOUSE | Lrrfip2 | 47.148 | 415 | 3 | 2 | 1 | 6893300 | 4276400 | 2617000 | 299710 | 185930 | 113780 | 0.70848288 |
| Q3TL44 | NLRX1_MOUSE | Nlrx1 | 107.831 | 975 | 2 | 1 | 1 | 4689900 | 2908500 | 1781400 | 91959 | 57029 | 34930 | 0.707263805 |
| O88967 | YMEL1_MOUSE | Yme1l1 | 80.028 | 715 | 30 | 14 | 16 | 271670000 | 168440000 | 1E+08 | 5544300 | 3437600 | 2106700 | 0.706372483 |
| P47963 | RL13_MOUSE | Rpl13 | 24.305 | 211 | 14 | 8 | 6 | 1214900000 | 752720000 | 4.6E+08 | 93454000 | 57902000 | 3.6E+07 | 0.703689689 |
| Q921E2 | RAB31_MOUSE | Rab31 | 21.331 | 194 | 4 | 2 | 2 | 18433000 | 11416000 | 7017100 | 1316600 | 815400 | 501220 | 0.702110413 |
| P32020 | SCP2_MOUSE | Scp2 | 59.126 | 547 | 4 | 2 | 2 | 18805000 | 11642000 | 7163300 | 626840 | 388060 | 238780 | 0.700642654 |
| Q11011 | PSA_MOUSE | Npepps | 103.325 | 920 | 4 | 2 | 2 | 8810600 | 5440500 | 3370100 | 154570 | 95447 | 59125 | 0.690947845 |
| P27659 | RL3_MOUSE | Rpl3 | 46.11 | 403 | 2 | 1 | 1 | 17068000 | 10522000 | 6546200 | 812770 | 501050 | 311720 | 0.68467937 |
| Q8BJW6 | EIF2A_MOUSE | Eif2a | 64.403 | 581 | 25 | 13 | 12 | 266590000 | 164320000 | 1E+08 | 7205100 | 4441100 | 2764000 | 0.684125081 |
| Q9DBG6 | RPN2_MOUSE | Rpn2 | 69.063 | 631 | 48 | 25 | 23 | 2755700000 | 1697700000 | 1.1E+09 | 102060000 | 62879000 | 3.9E+07 | 0.682241916 |
| Q9D0N7 | CAF1B_MOUSE | Chaf1b | 63.132 | 572 | 2 | 1 | 1 | 3615500 | 2226800 | 1388700 | 116630 | 71834 | 44797 | 0.68123702 |
| P53986 | MOT1_MOUSE | Slc16a1 | 53.267 | 493 | 17 | 9 | 8 | 242500000 | 149260000 | 9.3E+07 | 15156000 | 9328700 | 5827800 | 0.678744791 |
| Q9WVA3 | BUB3_MOUSE | Bub3 | 36.955 | 326 | 14 | 7 | 7 | 75681000 | 46486000 | 2.9E+07 | 4730000 | 2905400 | 1824700 | 0.671124397 |
| O70503 | DHB12_MOUSE | Hsd17b12 | 34.742 | 312 | 13 | 6 | 7 | 172980000 | 106240000 | 6.7E+07 | 9609700 | 5902400 | 3707300 | 0.670876404 |
| Q4VBE8 | WDR18_MOUSE | Wdr18 | 47.211 | 431 | 5 | 3 | 2 | 16319000 | 10020000 | 6299400 | 815950 | 500980 | 314970 | 0.669596181 |
| Q8BGX0 | TRI23_MOUSE | Trim23 | 63.931 | 574 | 2 | 1 | 1 | 9577100 | 5869500 | 3707600 | 308940 | 189340 | 119600 | 0.662752006 |
| Q8VCW4 | UN93B_MOUSE | Unc93b1 | 66.981 | 598 | 6 | 3 | 3 | 28338000 | 17351000 | 1.1E+07 | 1416900 | 867560 | 549350 | 0.659221301 |
| P68368 | TBA4A_MOUSE | Tuba4a | 49.924 | 448 | 4 | 2 | 2 | 87915000 | 53771000 | 3.4E+07 | 3822400 | 2337900 | 1484500 | 0.655196221 |
| Q3U0V1 | FUBP2_MOUSE | Khsrp | 76.775 | 748 | 2 | 1 | 1 | 12147000 | 7428500 | 4718200 | 311460 | 190470 | 120980 | 0.65483435 |
| Q3V009 | TMED1_MOUSE | Tmed1 | 25.263 | 227 | 5 | 3 | 2 | 51667000 | 31575000 | 2E+07 | 8611200 | 5262500 | 3348700 | 0.652161554 |
| Q91WV0 | NC2B_MOUSE | Dr1 | 19.431 | 176 | 2 | 1 | 1 | 12849000 | 7852100 | 4996500 | 1606100 | 981510 | 624560 | 0.652160692 |
| P62700 | YPEL5_MOUSE | Ypel5 | 13.842 | 121 | 5 | 3 | 2 | 25029000 | 15284000 | 9744500 | 2781000 | 1698200 | 1082700 | 0.649362096 |
| Q99P88 | NU155_MOUSE | Nup155 | 155.118 | 1391 | 3 | 1 | 2 | 6478900 | 3956100 | 2522700 | 96699 | 59047 | 37653 | 0.649110243 |
| P10639 | THIO_MOUSE | Txn | 11.675 | 105 | 14 | 7 | 7 | 1508300000 | 920290000 | 5.9E+08 | 215470000 | 131470000 | 8.4E+07 | 0.646321469 |
| Q99LC3 | NDUAA_MOUSE | Ndufa10 | 40.603 | 355 | 12 | 6 | 6 | 67518000 | 41144000 | 2.6E+07 | 2700700 | 1645700 | 1055000 | 0.641565665 |
| Q9JMD0 | ZN207_MOUSE | Znf207 | 52.793 | 495 | 2 | 1 | 1 | 8102600 | 4937400 | 3165200 | 450140 | 274300 | 175840 | 0.641454866 |
| Q8BGA9 | OXA1L_MOUSE | Oxa1l | 48.22 | 433 | 4 | 2 | 2 | 9945500 | 6056500 | 3889000 | 663030 | 403770 | 259270 | 0.639085079 |
| P99024 | TBB5_MOUSE | Tubb5 | 49.671 | 444 | 16 | 9 | 7 | 1939700000 | 1180600000 | 7.6E+08 | 88170000 | 53666000 | 3.5E+07 | 0.63715839 |
| Q9D2V8 | MFS10_MOUSE | Mfsd10 | 49.369 | 456 | 5 | 3 | 2 | 31413000 | 19119000 | 1.2E+07 | 1847800 | 1124600 | 723190 | 0.637052676 |
| Q3TYS2 | CYBC1_MOUSE | Cybc1 | 20.921 | 187 | 4 | 2 | 2 | 14941000 | 9091100 | 5850100 | 1149300 | 699310 | 450010 | 0.635993581 |
| P01887 | B2MG_MOUSE | B2m | 13.779 | 119 | 2 | 1 | 1 | 21937000 | 13347000 | 8590200 | 2742100 | 1668300 | 1073800 | 0.635751878 |
| Q9D5T0 | ATAD1_MOUSE | Atad1 | 40.744 | 361 | 6 | 3 | 3 | 48891000 | 29738000 | 1.9E+07 | 2222300 | 1351700 | 870590 | 0.634737239 |
| Q8VCK3 | TBG2_MOUSE | Tubg2 | 51.122 | 451 | 4 | 2 | 2 | 26835000 | 16322000 | 1.1E+07 | 1166700 | 709660 | 457070 | 0.63464343 |
| Q99KF1 | TMED9_MOUSE | Tmed9 | 27.127 | 235 | 5 | 3 | 2 | 29782000 | 18108000 | 1.2E+07 | 1861400 | 1131800 | 729630 | 0.633328238 |
| Q9D3B1 | HACD2_MOUSE | Hacd2 | 28.402 | 254 | 4 | 2 | 2 | 52973000 | 32181000 | 2.1E+07 | 4815700 | 2925600 | 1890200 | 0.630180618 |
| Q99LI2 | CLCC1_MOUSE | Clcc1 | 60.621 | 539 | 3 | 2 | 1 | 6314200 | 3834800 | 2479400 | 185710 | 112790 | 72922 | 0.629160298 |
| P19783 | COX41_MOUSE | Cox4i1 | 19.53 | 169 | 17 | 10 | 7 | 156980000 | 95294000 | 6.2E+07 | 13082000 | 7941200 | 5140700 | 0.627372121 |
| P99026 | PSB4_MOUSE | Psmb4 | 29.116 | 264 | 12 | 6 | 6 | 144660000 | 87768000 | 5.7E+07 | 13151000 | 7978900 | 5171900 | 0.625494592 |
| Q61112 | CAB45_MOUSE | Sdf4 | 42.064 | 361 | 10 | 5 | 5 | 90557000 | 54938000 | 3.6E+07 | 4116200 | 2497200 | 1619100 | 0.625116874 |
| Q8BH59 | S2512_MOUSE | Slc25a12 | 74.57 | 677 | 31 | 16 | 15 | 228240000 | 138200000 | 9E+07 | 5706000 | 3455100 | 2250900 | 0.618183747 |
| Q9DCS9 | NDUBA_MOUSE | Ndufb10 | 21.024 | 176 | 14 | 7 | 7 | 136040000 | 82293000 | 5.4E+07 | 12367000 | 7481200 | 4885700 | 0.614692861 |
| P20029 | BIP_MOUSE | Hspa5 | 72.422 | 655 | 63 | 32 | 31 | 3776800000 | 2284400000 | 1.5E+09 | 99391000 | 60116000 | 3.9E+07 | 0.614084358 |
| P42232 | STA5B_MOUSE | Stat5b | 90.002 | 786 | 2 | 1 | 1 | 15325000 | 9260900 | 6063900 | 333150 | 201320 | 131820 | 0.610906443 |
| P31650 | S6A11_MOUSE | Slc6a11 | 69.961 | 627 | 2 | 1 | 1 | 11499000 | 6939000 | 4559700 | 547560 | 330430 | 217130 | 0.60578886 |
| Q3UA06 | PCH2_MOUSE | Trip13 | 48.377 | 432 | 6 | 3 | 3 | 36558000 | 22012000 | 1.5E+07 | 1523200 | 917160 | 606080 | 0.597667752 |
| Q9D7G0 | PRPS1_MOUSE | Prps1 | 34.834 | 318 | 15 | 8 | 7 | 186810000 | 112450000 | 7.4E+07 | 9832300 | 5918200 | 3914100 | 0.596529782 |
| Q61216 | MRE11_MOUSE | Mre11 | 80.223 | 706 | 2 | 1 | 1 | 4934500 | 2969000 | 1965400 | 129850 | 78132 | 51722 | 0.595154132 |
| Q99LC8 | EI2BA_MOUSE | Eif2b1 | 33.816 | 305 | 2 | 1 | 1 | 11650000 | 7008600 | 4641400 | 685290 | 412270 | 273020 | 0.594566252 |
| Q9ERF3 | SKI8_MOUSE | Skic8 | 33.773 | 305 | 8 | 4 | 4 | 83482000 | 50101000 | 3.3E+07 | 5565400 | 3340100 | 2225400 | 0.585812225 |
| O35857 | TIM44_MOUSE | Timm44 | 51.091 | 452 | 2 | 1 | 1 | 5764300 | 3459300 | 2305000 | 185940 | 111590 | 74354 | 0.585713383 |
| Q9D1M4 | MCA3_MOUSE | Eef1e1 | 19.859 | 174 | 6 | 3 | 3 | 49179000 | 29476000 | 2E+07 | 3512800 | 2105400 | 1407400 | 0.581125446 |
| Q9D6Z1 | NOP56_MOUSE | Nop56 | 64.464 | 580 | 3 | 2 | 1 | 8045000 | 4821500 | 3223500 | 223470 | 133930 | 89541 | 0.580854065 |
| Q9Z0W3 | NU160_MOUSE | Nup160 | 158.232 | 1402 | 4 | 2 | 2 | 7123700 | 4268400 | 2855300 | 111310 | 66694 | 44614 | 0.580053046 |
| Q9D6T0 | NOSIP_MOUSE | Nosip | 33.209 | 301 | 2 | 1 | 1 | 8330900 | 4980500 | 3350500 | 416550 | 249020 | 167520 | 0.571914177 |
| Q9Z1Z0 | USO1_MOUSE | Uso1 | 106.983 | 959 | 1 | 0 | 1 | 1863000 | 0 | 1863000 | 36528 | 0 | 36528 | 0.571140215 |
| P62274 | RS29_MOUSE | Rps29 | 6.677 | 56 | 6 | 3 | 3 | 17081000 | 10205000 | 6876400 | 4270300 | 2551200 | 1719100 | 0.569550808 |
| P26450 | P85A_MOUSE | Pik3r1 | 83.517 | 724 | 2 | 1 | 1 | 3597100 | 2147800 | 1449300 | 78198 | 46692 | 31506 | 0.567503399 |
| Q8BP67 | RL24_MOUSE | Rpl24 | 17.779 | 157 | 10 | 5 | 5 | 134200000 | 80128000 | 5.4E+07 | 16775000 | 10016000 | 6758900 | 0.567451429 |
| P22366 | MYD88_MOUSE | Myd88 | 33.753 | 296 | 2 | 1 | 1 | 2791500 | 1665900 | 1125600 | 139580 | 83293 | 56282 | 0.565607568 |
| Q9WU56 | PUS1_MOUSE | Pus1 | 47.502 | 423 | 2 | 1 | 1 | 4226100 | 2519300 | 1706800 | 145730 | 86871 | 58856 | 0.561728914 |
| Q9Z110 | P5CS_MOUSE | Aldh18a1 | 87.266 | 795 | 6 | 3 | 3 | 18882000 | 11256000 | 7626800 | 377650 | 225110 | 152540 | 0.561544461 |
| Q9CY50 | SSRA_MOUSE | Ssr1 | 32.065 | 286 | 8 | 4 | 4 | 605760000 | 360900000 | 2.4E+08 | 55069000 | 32810000 | 2.2E+07 | 0.559700947 |
| O09167 | RL21_MOUSE | Rpl21 | 18.579 | 160 | 12 | 6 | 6 | 188570000 | 112340000 | 7.6E+07 | 23571000 | 14043000 | 9528200 | 0.559535558 |
| Q91VM5 | RMXL1_MOUSE | Rbmxl1 | 42.162 | 388 | 10 | 5 | 5 | 147030000 | 87595000 | 5.9E+07 | 4324500 | 2576300 | 1748200 | 0.55946295 |
| O55143 | AT2A2_MOUSE | Atp2a2 | 114.858 | 1044 | 57 | 31 | 26 | 775470000 | 461690000 | 3.1E+08 | 14632000 | 8711100 | 5920400 | 0.557171085 |
| Q64521 | GPDM_MOUSE | Gpd2 | 80.954 | 727 | 7 | 4 | 3 | 15837000 | 9415700 | 6421400 | 323210 | 192160 | 131050 | 0.552180485 |
| Q8K245 | UVRAG_MOUSE | Uvrag | 77.525 | 698 | 2 | 1 | 1 | 4105700 | 2440500 | 1665200 | 102640 | 61013 | 41631 | 0.551481288 |
| O54946 | DNJB6_MOUSE | Dnajb6 | 39.807 | 365 | 13 | 7 | 6 | 216340000 | 128600000 | 8.8E+07 | 12726000 | 7564400 | 5161700 | 0.551436053 |
| P24668 | MPRD_MOUSE | M6pr | 31.172 | 278 | 4 | 2 | 2 | 10554000 | 6269200 | 4284800 | 620820 | 368780 | 252050 | 0.54905349 |
| Q9CZD3 | GARS_MOUSE | Gars1 | 81.878 | 729 | 6 | 3 | 3 | 34806000 | 20662000 | 1.4E+07 | 682470 | 405130 | 277340 | 0.546789728 |
| Q9DBR3 | ARMC8_MOUSE | Armc8 | 75.364 | 673 | 8 | 4 | 4 | 19331000 | 11476000 | 7855900 | 439350 | 260810 | 178540 | 0.546771403 |
| O08579 | EMD_MOUSE | Emd | 29.436 | 259 | 16 | 8 | 8 | 175640000 | 104260000 | 7.1E+07 | 14637000 | 8688200 | 5948400 | 0.546573747 |
| Q9CZA6 | NDE1_MOUSE | Nde1 | 38.523 | 344 | 2 | 1 | 1 | 4706000 | 2792000 | 1914000 | 181000 | 107390 | 73616 | 0.544708112 |
| P60605 | UB2G2_MOUSE | Ube2g2 | 18.566 | 165 | 5 | 3 | 2 | 80710000 | 47838000 | 3.3E+07 | 13452000 | 7972900 | 5478700 | 0.541297839 |
| Q8BX10 | PGAM5_MOUSE | Pgam5 | 31.994 | 288 | 15 | 7 | 8 | 137830000 | 81504000 | 5.6E+07 | 6265200 | 3704700 | 2560400 | 0.532993005 |
| P06797 | CATL1_MOUSE | Ctsl | 37.547 | 334 | 11 | 5 | 6 | 114520000 | 67684000 | 4.7E+07 | 9543100 | 5640300 | 3902800 | 0.531289375 |
| P14685 | PSMD3_MOUSE | Psmd3 | 60.718 | 530 | 15 | 8 | 7 | 152580000 | 90068000 | 6.3E+07 | 3912400 | 2309400 | 1603000 | 0.526789154 |
| Q9QZL0 | RIPK3_MOUSE | Ripk3 | 53.322 | 486 | 5 | 3 | 2 | 54203000 | 31991000 | 2.2E+07 | 1693900 | 999730 | 694130 | 0.526326789 |
| Q9CRB9 | MIC19_MOUSE | Chchd3 | 26.335 | 227 | 4 | 2 | 2 | 35894000 | 21135000 | 1.5E+07 | 2111400 | 1243200 | 868200 | 0.518039138 |
| Q9D187 | CIA2B_MOUSE | Ciao2b | 17.667 | 163 | 4 | 2 | 2 | 22783000 | 13414000 | 9368200 | 3797100 | 2235700 | 1561400 | 0.517895726 |
| Q9JKL4 | NDUF3_MOUSE | Ndufaf3 | 20.734 | 185 | 2 | 1 | 1 | 4822600 | 2838900 | 1983700 | 535840 | 315430 | 220410 | 0.517138171 |
| Q9Z1R2 | BAG6_MOUSE | Bag6 | 121.037 | 1154 | 8 | 4 | 4 | 53785000 | 31661000 | 2.2E+07 | 1415400 | 833180 | 582220 | 0.517094576 |
| Q9QYA2 | TOM40_MOUSE | Tomm40 | 37.895 | 361 | 7 | 3 | 4 | 59584000 | 35059000 | 2.5E+07 | 4965400 | 2921600 | 2043800 | 0.51553171 |
| Q9CQV8 | 1433B_MOUSE | Ywhab | 28.086 | 246 | 7 | 4 | 3 | 74430000 | 43781000 | 3.1E+07 | 4378200 | 2575400 | 1802900 | 0.514464904 |
| Q78ZA7 | NP1L4_MOUSE | Nap1l4 | 42.679 | 375 | 3 | 2 | 1 | 7348900 | 4321300 | 3027500 | 408270 | 240070 | 168200 | 0.513338432 |
| Q9R1P3 | PSB2_MOUSE | Psmb2 | 22.906 | 201 | 8 | 4 | 4 | 59795000 | 35128000 | 2.5E+07 | 4982900 | 2927300 | 2055600 | 0.510039174 |
| Q60715 | P4HA1_MOUSE | P4ha1 | 60.91 | 534 | 5 | 2 | 3 | 19560000 | 11488000 | 8072700 | 698580 | 410270 | 288310 | 0.50900447 |
| P50429 | ARSB_MOUSE | Arsb | 59.647 | 534 | 2 | 1 | 1 | 6476100 | 3801800 | 2674300 | 231290 | 135780 | 95509 | 0.507521324 |
| P14901 | HMOX1_MOUSE | Hmox1 | 32.929 | 289 | 2 | 1 | 1 | 8153000 | 4785700 | 3367300 | 582360 | 341830 | 240520 | 0.507137702 |
| Q9CPW4 | ARPC5_MOUSE | Arpc5 | 16.288 | 151 | 15 | 8 | 7 | 243790000 | 143070000 | 1E+08 | 20316000 | 11922000 | 8393400 | 0.506371 |
| Q33DR2 | DPS1_MOUSE | Pdss1 | 45.895 | 409 | 2 | 1 | 1 | 7917800 | 4644000 | 3273900 | 282780 | 165860 | 116920 | 0.504357716 |
| P62270 | RS18_MOUSE | Rps18 | 17.719 | 152 | 30 | 16 | 14 | 3209400000 | 1881700000 | 1.3E+09 | 267450000 | 156810000 | 1.1E+08 | 0.503107437 |
| Q9CR98 | F136A_MOUSE | Fam136a | 15.674 | 138 | 2 | 1 | 1 | 10484000 | 6144800 | 4338800 | 1048400 | 614480 | 433880 | 0.502069969 |
| Q9CRD2 | EMC2_MOUSE | Emc2 | 34.935 | 297 | 4 | 2 | 2 | 18058000 | 10582000 | 7475800 | 1128600 | 661390 | 467240 | 0.501312445 |
| P62331 | ARF6_MOUSE | Arf6 | 20.082 | 175 | 2 | 1 | 1 | 7625200 | 4463700 | 3161500 | 847250 | 495960 | 351280 | 0.497630849 |
| P54775 | PRS6B_MOUSE | Psmc4 | 47.408 | 418 | 14 | 6 | 8 | 102550000 | 60033000 | 4.3E+07 | 3944300 | 2308900 | 1635400 | 0.497614168 |
| Q9D4H8 | CUL2_MOUSE | Cul2 | 86.877 | 745 | 4 | 2 | 2 | 13577000 | 7927800 | 5649300 | 277080 | 161790 | 115290 | 0.488848452 |
| Q8VCM8 | NCLN_MOUSE | Ncln | 62.908 | 563 | 10 | 5 | 5 | 50031000 | 29207000 | 2.1E+07 | 2382400 | 1390800 | 991650 | 0.487997684 |
| P23506 | PIMT_MOUSE | Pcmt1 | 24.634 | 227 | 10 | 5 | 5 | 102160000 | 59594000 | 4.3E+07 | 8513600 | 4966200 | 3547400 | 0.485363886 |
| Q64152 | BTF3_MOUSE | Btf3 | 22.031 | 204 | 17 | 8 | 9 | 613790000 | 357760000 | 2.6E+08 | 51149000 | 29814000 | 2.1E+07 | 0.482679227 |
| Q8K3W0 | BABA2_MOUSE | Babam2 | 43.545 | 383 | 2 | 1 | 1 | 9415100 | 5485900 | 3929200 | 495530 | 288730 | 206800 | 0.48149272 |
| P09103 | PDIA1_MOUSE | P4hb | 57.058 | 509 | 17 | 9 | 8 | 130140000 | 75758000 | 5.4E+07 | 3336900 | 1942500 | 1394400 | 0.478269034 |
| P17809 | GTR1_MOUSE | Slc2a1 | 53.985 | 492 | 8 | 4 | 4 | 72496000 | 42161000 | 3E+07 | 4833100 | 2810700 | 2022300 | 0.474925775 |
| Q8VDN2 | AT1A1_MOUSE | Atp1a1 | 112.982 | 1023 | 32 | 17 | 15 | 330730000 | 192230000 | 1.4E+08 | 6240200 | 3627000 | 2613200 | 0.472947529 |
| Q9D8W5 | PSD12_MOUSE | Psmd12 | 52.895 | 456 | 18 | 9 | 9 | 142540000 | 82818000 | 6E+07 | 4192400 | 2435800 | 1756600 | 0.471585259 |
| Q8CAS9 | PARP9_MOUSE | Parp9 | 96.659 | 866 | 31 | 16 | 15 | 229160000 | 133030000 | 9.6E+07 | 4583200 | 2660700 | 1922500 | 0.468783039 |
| Q3UYV9 | NCBP1_MOUSE | Ncbp1 | 91.927 | 790 | 15 | 6 | 9 | 65619000 | 38074000 | 2.8E+07 | 1726800 | 1002000 | 724860 | 0.467015682 |
| Q9D483 | RPC3_MOUSE | Polr3c | 60.706 | 533 | 2 | 1 | 1 | 5028700 | 2916000 | 2112700 | 139690 | 81000 | 58686 | 0.464902798 |
| P62717 | RL18A_MOUSE | Rpl18a | 20.732 | 176 | 3 | 2 | 1 | 26809000 | 15545000 | 1.1E+07 | 2062200 | 1195800 | 866460 | 0.464731378 |
| Q8BWT1 | THIM_MOUSE | Acaa2 | 41.83 | 397 | 3 | 2 | 1 | 10266000 | 5947700 | 4317900 | 410620 | 237910 | 172720 | 0.462002048 |
| Q9DCS3 | MECR_MOUSE | Mecr | 40.343 | 373 | 13 | 6 | 7 | 138480000 | 80218000 | 5.8E+07 | 6294500 | 3646300 | 2648300 | 0.461370771 |
| P48962 | ADT1_MOUSE | Slc25a4 | 32.904 | 298 | 12 | 6 | 6 | 378020000 | 218920000 | 1.6E+08 | 17183000 | 9950800 | 7231900 | 0.460469926 |
| P62754 | RS6_MOUSE | Rps6 | 28.681 | 249 | 13 | 6 | 7 | 533210000 | 308740000 | 2.2E+08 | 41017000 | 23750000 | 1.7E+07 | 0.459869765 |
| P68372 | TBB4B_MOUSE | Tubb4b | 49.831 | 445 | 58 | 30 | 28 | 5418800000 | 3135600000 | 2.3E+09 | 246310000 | 142530000 | 1E+08 | 0.457747479 |
| Q6ZQ08 | CNOT1_MOUSE | Cnot1 | 266.808 | 2375 | 6 | 3 | 3 | 19053000 | 11020000 | 8032600 | 140090 | 81029 | 59063 | 0.456185282 |
| Q6ZWU9 | RS27_MOUSE | Rps27 | 9.461 | 84 | 8 | 4 | 4 | 186140000 | 107630000 | 7.9E+07 | 37228000 | 21526000 | 1.6E+07 | 0.455058428 |
| P08113 | ENPL_MOUSE | Hsp90b1 | 92.476 | 802 | 28 | 14 | 14 | 200250000 | 115770000 | 8.4E+07 | 4260600 | 2463300 | 1797300 | 0.454682178 |
| Q9CRB2 | NHP2_MOUSE | Nhp2 | 17.247 | 153 | 2 | 1 | 1 | 13309000 | 7694600 | 5614700 | 2218200 | 1282400 | 935780 | 0.454637391 |
| O70152 | DPM1_MOUSE | Dpm1 | 29.175 | 260 | 17 | 8 | 9 | 194490000 | 112410000 | 8.2E+07 | 10805000 | 6244900 | 4560200 | 0.453615018 |
| Q7TQH0 | ATX2L_MOUSE | Atxn2l | 110.649 | 1049 | 17 | 9 | 8 | 119580000 | 69112000 | 5E+07 | 2781000 | 1607300 | 1173800 | 0.453452973 |
| Q91YN9 | BAG2_MOUSE | Bag2 | 23.474 | 210 | 12 | 6 | 6 | 190320000 | 109920000 | 8E+07 | 11895000 | 6870100 | 5025000 | 0.451168559 |
| Q9CY16 | RT28_MOUSE | Mrps28 | 20.52 | 186 | 4 | 2 | 2 | 20263000 | 11699000 | 8564000 | 1447300 | 835630 | 611710 | 0.450028516 |
| Q9CPR4 | RL17_MOUSE | Rpl17 | 21.423 | 184 | 12 | 6 | 6 | 274110000 | 158220000 | 1.2E+08 | 24919000 | 14384000 | 1.1E+07 | 0.449175894 |
| O88487 | DC1I2_MOUSE | Dync1i2 | 68.394 | 612 | 2 | 1 | 1 | 960150000 | 554150000 | 4.1E+08 | 41746000 | 24094000 | 1.8E+07 | 0.448832352 |
| Q6ZWV3 | RL10_MOUSE | Rpl10 | 24.604 | 214 | 6 | 3 | 3 | 190900000 | 110130000 | 8.1E+07 | 15908000 | 9177400 | 6730900 | 0.447298214 |
| Q80ZS3 | RT26_MOUSE | Mrps26 | 23.444 | 200 | 6 | 3 | 3 | 91166000 | 52578000 | 3.9E+07 | 5697900 | 3286100 | 2411700 | 0.446306993 |
| Q9CYA6 | ZCHC8_MOUSE | Zcchc8 | 78.026 | 709 | 4 | 2 | 2 | 24429000 | 14087000 | 1E+07 | 718500 | 414310 | 304190 | 0.445709703 |
| Q924Z4 | CERS2_MOUSE | Cers2 | 45.024 | 380 | 6 | 3 | 3 | 96136000 | 55409000 | 4.1E+07 | 6008500 | 3463100 | 2545400 | 0.444170206 |
| Q99LE6 | ABCF2_MOUSE | Abcf2 | 71.782 | 628 | 3 | 1 | 2 | 14622000 | 8423300 | 6198400 | 406160 | 233980 | 172180 | 0.44248969 |
| P05132 | KAPCA_MOUSE | Prkaca | 40.571 | 351 | 2 | 1 | 1 | 9462400 | 5450200 | 4012200 | 473120 | 272510 | 200610 | 0.441915649 |
| Q9DC16 | ERGI1_MOUSE | Ergic1 | 32.562 | 290 | 11 | 5 | 6 | 83871000 | 48266000 | 3.6E+07 | 6451600 | 3712800 | 2738800 | 0.438927417 |
| P17095 | HMGA1_MOUSE | Hmga1 | 11.614 | 107 | 4 | 2 | 2 | 64416000 | 37067000 | 2.7E+07 | 10736000 | 6177800 | 4558200 | 0.438647273 |
| Q9EQ32 | BCAP_MOUSE | Pik3ap1 | 90.928 | 811 | 5 | 2 | 3 | 27615000 | 15889000 | 1.2E+07 | 708080 | 407410 | 300680 | 0.438317367 |
| P12265 | BGLR_MOUSE | Gusb | 74.195 | 648 | 4 | 2 | 2 | 18478000 | 10628000 | 7849900 | 513270 | 295220 | 218050 | 0.437123952 |
| P00158 | CYB_MOUSE | Mt-Cyb | 43.21 | 381 | 2 | 1 | 1 | 6391000 | 3675000 | 2716100 | 1065200 | 612500 | 452680 | 0.436207653 |
| Q9Z2G6 | SE1L1_MOUSE | Sel1l | 88.34 | 790 | 8 | 4 | 4 | 72397000 | 41626000 | 3.1E+07 | 1765800 | 1015300 | 750510 | 0.435913602 |
| Q922F4 | TBB6_MOUSE | Tubb6 | 50.09 | 447 | 23 | 12 | 11 | 445780000 | 256270000 | 1.9E+08 | 19382000 | 11142000 | 8239500 | 0.435390623 |
| Q80X82 | SYMPK_MOUSE | Sympk | 142.62 | 1288 | 4 | 2 | 2 | 9459100 | 5426600 | 4032500 | 133230 | 76431 | 56796 | 0.428374037 |
| Q91WQ3 | SYYC_MOUSE | Yars1 | 59.105 | 528 | 10 | 6 | 4 | 42987000 | 24645000 | 1.8E+07 | 955270 | 547670 | 407600 | 0.426144023 |
| Q6ZWN5 | RS9_MOUSE | Rps9 | 22.591 | 194 | 16 | 8 | 8 | 801220000 | 458590000 | 3.4E+08 | 57230000 | 32756000 | 2.4E+07 | 0.420553419 |
| E9Q5C9 | NOLC1_MOUSE | Nolc1 | 73.698 | 702 | 8 | 4 | 4 | 32849000 | 18798000 | 1.4E+07 | 821220 | 469960 | 351250 | 0.420009045 |
| Q9CZ42 | NNRD_MOUSE | Naxd | 36.717 | 343 | 2 | 1 | 1 | 9887800 | 5657200 | 4230600 | 549320 | 314290 | 235030 | 0.419225889 |
| Q7TNP2 | 2AAB_MOUSE | Ppp2r1b | 65.934 | 601 | 5 | 2 | 3 | 18654000 | 10673000 | 7981600 | 504170 | 288450 | 215720 | 0.419215866 |
| Q9JII5 | DAZP1_MOUSE | Dazap1 | 43.214 | 406 | 6 | 3 | 3 | 30603000 | 17505000 | 1.3E+07 | 2354100 | 1346500 | 1007600 | 0.418420526 |
| P58064 | RT06_MOUSE | Mrps6 | 14.309 | 125 | 5 | 2 | 3 | 34470000 | 19701000 | 1.5E+07 | 3830000 | 2189000 | 1641000 | 0.415696716 |
| Q9D7H3 | RTCA_MOUSE | RtcA | 39.254 | 366 | 10 | 5 | 5 | 51433000 | 29386000 | 2.2E+07 | 2449200 | 1399300 | 1049900 | 0.414481201 |
| Q62419 | SH3G1_MOUSE | Sh3gl1 | 41.518 | 368 | 2 | 1 | 1 | 6032500 | 3446600 | 2586000 | 301630 | 172330 | 129300 | 0.414451599 |
| Q80VQ0 | AL3B1_MOUSE | Aldh3b1 | 52.292 | 468 | 2 | 1 | 1 | 9360300 | 5344800 | 4015500 | 374410 | 213790 | 160620 | 0.412556324 |
| Q7TPR4 | ACTN1_MOUSE | Actn1 | 103.068 | 892 | 31 | 16 | 15 | 281170000 | 160350000 | 1.2E+08 | 4847700 | 2764700 | 2083000 | 0.408484476 |
| Q91VD9 | NDUS1_MOUSE | Ndufs1 | 79.777 | 727 | 8 | 4 | 4 | 36630000 | 20877000 | 1.6E+07 | 813990 | 463930 | 350060 | 0.406287812 |
| P16858 | G3P_MOUSE | Gapdh | 35.81 | 333 | 59 | 30 | 29 | 5512500000 | 3140900000 | 2.4E+09 | 262500000 | 149570000 | 1.1E+08 | 0.405317309 |
| Q04447 | KCRB_MOUSE | Ckb | 42.713 | 381 | 26 | 13 | 13 | 346300000 | 197310000 | 1.5E+08 | 15056000 | 8578500 | 6477800 | 0.405248574 |
| Q80X50 | UBP2L_MOUSE | Ubap2l | 116.799 | 1107 | 8 | 4 | 4 | 59533000 | 33907000 | 2.6E+07 | 1751000 | 997270 | 753700 | 0.40397484 |
| Q5U458 | DJC11_MOUSE | Dnajc11 | 63.233 | 559 | 5 | 2 | 3 | 17675000 | 10065000 | 7609500 | 631250 | 359480 | 271770 | 0.403473606 |
| Q9D0M3 | CY1_MOUSE | Cyc1 | 35.328 | 325 | 12 | 6 | 6 | 134560000 | 76608000 | 5.8E+07 | 8410100 | 4788000 | 3622100 | 0.402586818 |
| P62334 | PRS10_MOUSE | Psmc6 | 44.173 | 389 | 11 | 6 | 5 | 79926000 | 45447000 | 3.4E+07 | 3074100 | 1748000 | 1326100 | 0.398467132 |
| P05213 | TBA1B_MOUSE | Tuba1b | 50.152 | 451 | 46 | 22 | 24 | 6248500000 | 3552900000 | 2.7E+09 | 271670000 | 154470000 | 1.2E+08 | 0.398390652 |
| Q8BP92 | RCN2_MOUSE | Rcn2 | 37.271 | 320 | 28 | 14 | 14 | 702790000 | 399090000 | 3E+08 | 46853000 | 26606000 | 2E+07 | 0.394019718 |
| O89086 | RBM3_MOUSE | Rbm3 | 16.605 | 153 | 3 | 2 | 1 | 29976000 | 17010000 | 1.3E+07 | 3330700 | 1890000 | 1440700 | 0.391649663 |
| Q8BMJ2 | SYLC_MOUSE | Lars1 | 134.192 | 1178 | 25 | 13 | 12 | 168040000 | 95273000 | 7.3E+07 | 2471200 | 1401100 | 1070200 | 0.388683957 |
| P28656 | NP1L1_MOUSE | Nap1l1 | 45.345 | 391 | 14 | 7 | 7 | 566710000 | 320980000 | 2.5E+08 | 40479000 | 22927000 | 1.8E+07 | 0.385409407 |
| P49717 | MCM4_MOUSE | Mcm4 | 96.736 | 862 | 2 | 1 | 1 | 9413500 | 5330200 | 4083300 | 196110 | 111050 | 85068 | 0.384454101 |
| O88685 | PRS6A_MOUSE | Psmc3 | 49.549 | 442 | 30 | 15 | 15 | 369130000 | 208960000 | 1.6E+08 | 13672000 | 7739400 | 5932100 | 0.383622847 |
| P43247 | MSH2_MOUSE | Msh2 | 104.151 | 935 | 2 | 1 | 1 | 6853600 | 3876200 | 2977400 | 124610 | 70476 | 54134 | 0.380589957 |
| P11499 | HS90B_MOUSE | Hsp90ab1 | 83.281 | 724 | 67 | 34 | 33 | 1906500000 | 1076700000 | 8.3E+08 | 50170000 | 28335000 | 2.2E+07 | 0.375885087 |
| P62984 | RL40_MOUSE | Uba52 | 14.728 | 128 | 13 | 7 | 6 | 2081300000 | 1174900000 | 9.1E+08 | 231260000 | 130550000 | 1E+08 | 0.374365954 |
| Q9CZH3 | PSMG3_MOUSE | Psmg3 | 13.331 | 122 | 2 | 1 | 1 | 3915600 | 2209500 | 1706200 | 559370 | 315640 | 243740 | 0.372933162 |
| Q99P91 | GPNMB_MOUSE | Gpnmb | 63.676 | 574 | 4 | 2 | 2 | 20262000 | 11431000 | 8830600 | 920980 | 519590 | 401390 | 0.372368247 |
| Q8R3P6 | INT14_MOUSE | Ints14 | 57.237 | 515 | 2 | 1 | 1 | 7618000 | 4294800 | 3323200 | 262690 | 148100 | 114590 | 0.370017828 |
| Q9JKX6 | NUDT5_MOUSE | Nudt5 | 23.984 | 218 | 12 | 6 | 6 | 177190000 | 99832000 | 7.7E+07 | 13630000 | 7679400 | 5950900 | 0.367877236 |
| Q9Z2N8 | ACL6A_MOUSE | Actl6a | 47.448 | 429 | 2 | 1 | 1 | 10586000 | 5964000 | 4622300 | 481200 | 271090 | 210110 | 0.36766936 |
| Q8CCS6 | PABP2_MOUSE | Pabpn1 | 32.297 | 302 | 6 | 3 | 3 | 134310000 | 75636000 | 5.9E+07 | 8953700 | 5042400 | 3911300 | 0.366450077 |
| Q9JL26 | FMNL1_MOUSE | Fmnl1 | 122.06 | 1094 | 15 | 7 | 8 | 53793000 | 30294000 | 2.3E+07 | 815050 | 459010 | 356040 | 0.366432719 |
| Q9WVJ2 | PSD13_MOUSE | Psmd13 | 42.809 | 376 | 18 | 9 | 9 | 124320000 | 69997000 | 5.4E+07 | 4781700 | 2692200 | 2089500 | 0.365597154 |
| Q8BJY1 | PSMD5_MOUSE | Psmd5 | 55.972 | 504 | 2 | 1 | 1 | 4657400 | 2620800 | 2036600 | 155250 | 87360 | 67888 | 0.363844608 |
| Q8VCB1 | NDC1_MOUSE | Ndc1 | 75.409 | 673 | 3 | 2 | 1 | 10736000 | 6040800 | 4694700 | 397610 | 223730 | 173880 | 0.363706652 |
| Q8C6A8 | BHE22_MOUSE | Bhlhe22 | 35.217 | 355 | 2 | 1 | 1 | 12427000 | 6986500 | 5440800 | 828490 | 465770 | 362720 | 0.360751098 |
| P97808 | FXYD5_MOUSE | Fxyd5 | 19.454 | 178 | 2 | 1 | 1 | 11972000 | 6726500 | 5245800 | 1088400 | 611500 | 476890 | 0.358693218 |
| O08756 | HCD2_MOUSE | Hsd17b10 | 27.419 | 261 | 7 | 4 | 3 | 22282000 | 12511000 | 9771300 | 1392600 | 781920 | 610700 | 0.356574688 |
| P00405 | COX2_MOUSE | Mtco2 | 25.976 | 227 | 12 | 7 | 5 | 251470000 | 141190000 | 1.1E+08 | 31434000 | 17649000 | 1.4E+07 | 0.356466739 |
| P14869 | RLA0_MOUSE | Rplp0 | 34.216 | 317 | 37 | 18 | 19 | 6417700000 | 3599200000 | 2.8E+09 | 356540000 | 199960000 | 1.6E+08 | 0.352799892 |
| P97287 | MCL1_MOUSE | Mcl1 | 35.217 | 331 | 4 | 2 | 2 | 9815200 | 5503300 | 4311900 | 490760 | 275170 | 215600 | 0.351973256 |
| P29416 | HEXA_MOUSE | Hexa | 60.613 | 528 | 6 | 3 | 3 | 43338000 | 24286000 | 1.9E+07 | 1666800 | 934060 | 732770 | 0.350182438 |
| Q9R1P4 | PSA1_MOUSE | Psma1 | 29.547 | 263 | 15 | 7 | 8 | 349540000 | 195690000 | 1.5E+08 | 19419000 | 10872000 | 8547200 | 0.347045591 |
| A2AAJ9 | OBSCN_MOUSE | Obscn | 966.591 | 8886 | 3 | 2 | 1 | 51230000 | 28678000 | 2.3E+07 | 99863 | 55902 | 43961 | 0.346689031 |
| Q9D7S7 | RL22L_MOUSE | Rpl22l1 | 14.467 | 122 | 2 | 1 | 1 | 79767000 | 44640000 | 3.5E+07 | 13294000 | 7440000 | 5854400 | 0.345756658 |
| Q99LT0 | DPY30_MOUSE | Dpy30 | 11.213 | 99 | 4 | 2 | 2 | 54009000 | 30204000 | 2.4E+07 | 9001600 | 5034000 | 3967600 | 0.34341439 |
| Q9Z2X1 | HNRPF_MOUSE | Hnrnpf | 45.73 | 415 | 12 | 6 | 6 | 72254000 | 40398000 | 3.2E+07 | 3141500 | 1756400 | 1385000 | 0.342718744 |
| O55125 | NIPS1_MOUSE | Nipsnap1 | 33.363 | 284 | 8 | 4 | 4 | 98251000 | 54912000 | 4.3E+07 | 5779500 | 3230100 | 2549300 | 0.341455593 |
| O08749 | DLDH_MOUSE | Dld | 54.272 | 509 | 11 | 5 | 6 | 58716000 | 32799000 | 2.6E+07 | 2258300 | 1261500 | 996820 | 0.3397531 |
| P47955 | RLA1_MOUSE | Rplp1 | 11.475 | 114 | 9 | 4 | 5 | 1539800000 | 859530000 | 6.8E+08 | 384960000 | 214880000 | 1.7E+08 | 0.337398112 |
| P13864 | DNMT1_MOUSE | Dnmt1 | 183.189 | 1620 | 2 | 1 | 1 | 6203100 | 3461600 | 2741500 | 63949 | 35686 | 28263 | 0.336473552 |
| P47740 | AL3A2_MOUSE | Aldh3a2 | 53.971 | 484 | 13 | 6 | 7 | 84880000 | 47228000 | 3.8E+07 | 3858200 | 2146700 | 1711400 | 0.326915942 |
| P46460 | NSF_MOUSE | Nsf | 82.613 | 744 | 11 | 6 | 5 | 38438000 | 21296000 | 1.7E+07 | 698870 | 387200 | 311670 | 0.313047034 |
| Q9ES28 | ARHG7_MOUSE | Arhgef7 | 97.056 | 862 | 4 | 2 | 2 | 10907000 | 6037300 | 4869600 | 201980 | 111800 | 90177 | 0.310100221 |
| Q6ZWQ7 | SPCS3_MOUSE | Spcs3 | 20.313 | 180 | 6 | 3 | 3 | 59846000 | 33113000 | 2.7E+07 | 8549500 | 4730400 | 3819100 | 0.30872201 |
| P33610 | PRI2_MOUSE | Prim2 | 58.408 | 505 | 6 | 3 | 3 | 24348000 | 13455000 | 1.1E+07 | 695650 | 384440 | 311210 | 0.304873503 |
| Q63850 | NUP62_MOUSE | Nup62 | 53.255 | 526 | 2 | 1 | 1 | 12008000 | 6633300 | 5374400 | 923670 | 510250 | 413420 | 0.303623075 |
| O08795 | GLU2B_MOUSE | Prkcsh | 58.793 | 521 | 3 | 2 | 1 | 28236000 | 15597000 | 1.3E+07 | 1411800 | 779870 | 631940 | 0.303386239 |
| P60229 | EIF3E_MOUSE | Eif3e | 52.221 | 445 | 25 | 13 | 12 | 196410000 | 108410000 | 8.8E+07 | 6137700 | 3387800 | 2749900 | 0.300971595 |
| Q9ERK4 | XPO2_MOUSE | Cse1l | 110.455 | 971 | 23 | 11 | 12 | 194090000 | 107050000 | 8.7E+07 | 3465900 | 1911600 | 1554400 | 0.298434887 |
| P61211 | ARL1_MOUSE | Arl1 | 20.412 | 181 | 9 | 5 | 4 | 75521000 | 41648000 | 3.4E+07 | 7552100 | 4164800 | 3387300 | 0.29811145 |
| Q99LF4 | RTCB_MOUSE | Rtcb | 55.249 | 505 | 42 | 23 | 19 | 1021100000 | 563080000 | 4.6E+08 | 35209000 | 19417000 | 1.6E+07 | 0.298086813 |
| Q9CQA3 | SDHB_MOUSE | Sdhb | 31.814 | 282 | 6 | 3 | 3 | 27711000 | 15275000 | 1.2E+07 | 1204800 | 664120 | 540710 | 0.296649858 |
| P63325 | RS10_MOUSE | Rps10 | 18.916 | 165 | 17 | 8 | 9 | 2143700000 | 1181300000 | 9.6E+08 | 178640000 | 98441000 | 8E+07 | 0.295726811 |
| Q8BVK9 | SP110_MOUSE | Sp110 | 50.14 | 445 | 4 | 2 | 2 | 15758000 | 8673300 | 7084600 | 562780 | 309760 | 253020 | 0.291894611 |
| P56395 | CYB5_MOUSE | Cyb5a | 15.241 | 134 | 2 | 1 | 1 | 16470000 | 9064900 | 7404800 | 2058700 | 1133100 | 925590 | 0.291830335 |
| P80317 | TCPZ_MOUSE | Cct6a | 58.004 | 531 | 29 | 14 | 15 | 372920000 | 205070000 | 1.7E+08 | 13319000 | 7324000 | 5994800 | 0.288943916 |
| Q9CX30 | YIF1B_MOUSE | Yif1b | 33.983 | 311 | 2 | 1 | 1 | 7305700 | 4014500 | 3291200 | 811740 | 446060 | 365690 | 0.286606614 |
| P25444 | RS2_MOUSE | Rps2 | 31.231 | 293 | 24 | 12 | 12 | 708840000 | 389330000 | 3.2E+08 | 32220000 | 17697000 | 1.5E+07 | 0.285177585 |
| Q02053 | UBA1_MOUSE | Uba1 | 117.809 | 1058 | 5 | 3 | 2 | 17185000 | 9430700 | 7754700 | 296300 | 162600 | 133700 | 0.28229389 |
| Q3TDQ1 | STT3B_MOUSE | Stt3b | 93.246 | 823 | 14 | 7 | 7 | 59284000 | 32507000 | 2.7E+07 | 1602300 | 878580 | 723700 | 0.279756084 |
| P17225 | PTBP1_MOUSE | Ptbp1 | 59.322 | 555 | 22 | 11 | 11 | 598200000 | 327880000 | 2.7E+08 | 27191000 | 14904000 | 1.2E+07 | 0.278553018 |
| P11835 | ITB2_MOUSE | Itgb2 | 85.026 | 771 | 5 | 2 | 3 | 19826000 | 10851000 | 8975000 | 381270 | 208670 | 172600 | 0.27384416 |
| P41105 | RL28_MOUSE | Rpl28 | 15.733 | 137 | 13 | 6 | 7 | 1064100000 | 582380000 | 4.8E+08 | 118230000 | 64708000 | 5.4E+07 | 0.273765989 |
| Q99LD9 | EI2BB_MOUSE | Eif2b2 | 38.898 | 351 | 6 | 3 | 3 | 24443000 | 13371000 | 1.1E+07 | 1286500 | 703740 | 582760 | 0.272191519 |
| P36371 | TAP2_MOUSE | Tap2 | 77.445 | 702 | 4 | 2 | 2 | 9752400 | 5330200 | 4422300 | 278640 | 152290 | 126350 | 0.269392769 |
| P46978 | STT3A_MOUSE | Stt3a | 80.598 | 705 | 27 | 13 | 14 | 354950000 | 193880000 | 1.6E+08 | 9859900 | 5385600 | 4474300 | 0.267476177 |
| Q62351 | TFR1_MOUSE | Tfrc | 85.731 | 763 | 36 | 19 | 17 | 428230000 | 233770000 | 1.9E+08 | 9309500 | 5081900 | 4227500 | 0.265542184 |
| P28658 | ATX10_MOUSE | Atxn10 | 53.707 | 475 | 6 | 3 | 3 | 32673000 | 17808000 | 1.5E+07 | 1054000 | 574470 | 479500 | 0.260703091 |
| O35316 | SC6A6_MOUSE | Slc6a6 | 69.856 | 621 | 4 | 2 | 2 | 21789000 | 11875000 | 9913800 | 1146800 | 625020 | 521780 | 0.260417454 |
| Q3TZ89 | SC31B_MOUSE | Sec31b | 125.633 | 1158 | 2 | 1 | 1 | 31675000 | 17262000 | 1.4E+07 | 546120 | 297630 | 248490 | 0.26032907 |
| Q9DAR7 | DCPS_MOUSE | Dcps | 38.988 | 338 | 2 | 1 | 1 | 8176600 | 4449800 | 3726700 | 389360 | 211900 | 177460 | 0.255841808 |
| P53702 | CCHL_MOUSE | Hccs | 30.978 | 272 | 4 | 2 | 2 | 9853700 | 5359300 | 4494300 | 615850 | 334960 | 280900 | 0.253948147 |
| Q61029 | LAP2B_MOUSE | Tmpo | 50.373 | 452 | 19 | 9 | 10 | 132340000 | 71815000 | 6.1E+07 | 5514300 | 2992300 | 2522000 | 0.246658695 |
| P57787 | MOT4_MOUSE | Slc16a3 | 50.373 | 470 | 10 | 5 | 5 | 87804000 | 47629000 | 4E+07 | 4621200 | 2506800 | 2114400 | 0.245578146 |
| Q9D1M7 | FKB11_MOUSE | Fkbp11 | 22.137 | 201 | 4 | 2 | 2 | 32132000 | 17421000 | 1.5E+07 | 3570300 | 1935700 | 1634600 | 0.243932121 |
| Q9D710 | TMX2_MOUSE | Tmx2 | 33.943 | 295 | 9 | 4 | 5 | 49169000 | 26638000 | 2.3E+07 | 2587900 | 1402000 | 1185900 | 0.241574421 |
| Q9D0F6 | RFC5_MOUSE | Rfc5 | 38.096 | 339 | 5 | 3 | 2 | 30923000 | 16745000 | 1.4E+07 | 1145300 | 620170 | 525120 | 0.240076341 |
| Q99J47 | DRS7B_MOUSE | Dhrs7b | 34.987 | 323 | 11 | 5 | 6 | 79909000 | 43254000 | 3.7E+07 | 3995400 | 2162700 | 1832800 | 0.238823549 |
| O35704 | SPTC1_MOUSE | Sptlc1 | 52.535 | 473 | 16 | 8 | 8 | 195020000 | 105530000 | 8.9E+07 | 8125600 | 4397000 | 3728600 | 0.237903167 |
| Q8BFV2 | PCID2_MOUSE | Pcid2 | 46.132 | 399 | 2 | 1 | 1 | 9392100 | 5075700 | 4316500 | 313070 | 169190 | 143880 | 0.233744813 |
| P62908 | RS3_MOUSE | Rps3 | 26.674 | 243 | 40 | 20 | 20 | 4698500000 | 2538300000 | 2.2E+09 | 247290000 | 133590000 | 1.1E+08 | 0.232697701 |
| Q3TJZ6 | FA98A_MOUSE | Fam98a | 55.055 | 515 | 4 | 2 | 2 | 31504000 | 17012000 | 1.4E+07 | 1312700 | 708820 | 603830 | 0.231296049 |
| Q9DCU6 | RM04_MOUSE | Mrpl4 | 33.073 | 294 | 17 | 8 | 9 | 148520000 | 80173000 | 6.8E+07 | 7426200 | 4008700 | 3417500 | 0.230154014 |
| P68181 | KAPCB_MOUSE | Prkacb | 40.708 | 351 | 7 | 3 | 4 | 40397000 | 21776000 | 1.9E+07 | 2019900 | 1088800 | 931050 | 0.22580842 |
| P70404 | IDHG1_MOUSE | Idh3g | 42.785 | 393 | 8 | 4 | 4 | 20043000 | 10801000 | 9241400 | 1054900 | 568490 | 486390 | 0.224981559 |
| P63073 | IF4E_MOUSE | Eif4e | 25.053 | 217 | 12 | 6 | 6 | 270770000 | 145880000 | 1.2E+08 | 19340000 | 10420000 | 8920300 | 0.224239662 |
| P63154 | CRNL1_MOUSE | Crnkl1 | 83.416 | 690 | 5 | 3 | 2 | 92913000 | 49994000 | 4.3E+07 | 2019800 | 1086800 | 933010 | 0.220138499 |
| Q8C1B7 | SEP11_MOUSE | Septin11 | 49.695 | 431 | 2 | 1 | 1 | 5534200 | 2975200 | 2558900 | 204970 | 110190 | 94776 | 0.217462884 |
| P47754 | CAZA2_MOUSE | Capza2 | 32.967 | 286 | 30 | 15 | 15 | 617430000 | 331930000 | 2.9E+08 | 38589000 | 20746000 | 1.8E+07 | 0.217438814 |
| Q8JZR0 | ACSL5_MOUSE | Acsl5 | 76.206 | 683 | 3 | 1 | 2 | 9339000 | 5020500 | 4318500 | 239460 | 128730 | 110730 | 0.217300762 |
| O35593 | PSDE_MOUSE | Psmd14 | 34.577 | 310 | 5 | 2 | 3 | 46269000 | 24870000 | 2.1E+07 | 2721700 | 1462900 | 1258800 | 0.216863128 |
| Q8BMD8 | SCMC1_MOUSE | Slc25a24 | 52.902 | 475 | 4 | 2 | 2 | 9988300 | 5368500 | 4619800 | 344430 | 185120 | 159300 | 0.216688649 |
| P18572 | BASI_MOUSE | Bsg | 42.445 | 389 | 16 | 8 | 8 | 242630000 | 130220000 | 1.1E+08 | 12131000 | 6511100 | 5620400 | 0.21218066 |
| Q99LI7 | CSTF3_MOUSE | Cstf3 | 82.877 | 717 | 4 | 2 | 2 | 12352000 | 6626900 | 5724800 | 308790 | 165670 | 143120 | 0.211108857 |
| P99027 | RLA2_MOUSE | Rplp2 | 11.651 | 115 | 28 | 14 | 14 | 3058100000 | 1639100000 | 1.4E+09 | 611610000 | 327820000 | 2.8E+08 | 0.208029285 |
| O55128 | SAP18_MOUSE | Sap18 | 17.595 | 153 | 4 | 2 | 2 | 29425000 | 15766000 | 1.4E+07 | 2263400 | 1212700 | 1050700 | 0.206964814 |
| Q9QZH6 | ECSIT_MOUSE | Ecsit | 49.799 | 435 | 16 | 8 | 8 | 89052000 | 47689000 | 4.1E+07 | 3180400 | 1703200 | 1477300 | 0.205315705 |
| Q9CPY7 | AMPL_MOUSE | Lap3 | 56.141 | 519 | 19 | 9 | 10 | 182850000 | 97806000 | 8.5E+07 | 6095200 | 3260200 | 2835000 | 0.201628698 |
| P17427 | AP2A2_MOUSE | Ap2a2 | 104.017 | 938 | 8 | 4 | 4 | 36623000 | 19575000 | 1.7E+07 | 620740 | 331780 | 288960 | 0.199325186 |
| Q9WU81 | G6PT3_MOUSE | Slc37a2 | 55.073 | 501 | 2 | 1 | 1 | 6229900 | 3329200 | 2900600 | 444990 | 237800 | 207190 | 0.198824184 |
| Q9CQR6 | PPP6_MOUSE | Ppp6c | 35.159 | 305 | 8 | 4 | 4 | 62902000 | 33606000 | 2.9E+07 | 3145100 | 1680300 | 1464800 | 0.198015138 |
| Q99J95 | CDK9_MOUSE | Cdk9 | 42.762 | 372 | 4 | 2 | 2 | 19950000 | 10654000 | 9296000 | 831230 | 443900 | 387330 | 0.196713212 |
| P62075 | TIM13_MOUSE | Timm13 | 10.458 | 95 | 5 | 3 | 2 | 86872000 | 46363000 | 4.1E+07 | 14479000 | 7727200 | 6751500 | 0.19473145 |
| Q9DBE8 | ALG2_MOUSE | Alg2 | 47.405 | 415 | 2 | 1 | 1 | 9399400 | 5013800 | 4385600 | 408670 | 217990 | 190680 | 0.193130215 |
| Q9Z0N1 | IF2G_MOUSE | Eif2s3x | 51.065 | 472 | 18 | 9 | 9 | 152760000 | 81374000 | 7.1E+07 | 6110300 | 3254900 | 2855400 | 0.188967164 |
| Q8BH15 | CNO10_MOUSE | Cnot10 | 81.818 | 744 | 2 | 1 | 1 | 5136300 | 2735700 | 2400600 | 116730 | 62175 | 54560 | 0.188514997 |
| Q76MZ3 | 2AAA_MOUSE | Ppp2r1a | 65.323 | 589 | 46 | 24 | 22 | 847350000 | 450940000 | 4E+08 | 21727000 | 11562000 | 1E+07 | 0.185905737 |
| Q9D1P0 | RM13_MOUSE | Mrpl13 | 20.677 | 178 | 6 | 3 | 3 | 80200000 | 42638000 | 3.8E+07 | 5012500 | 2664900 | 2347600 | 0.182865886 |
| P63101 | 1433Z_MOUSE | Ywhaz | 27.771 | 245 | 15 | 7 | 8 | 368400000 | 195840000 | 1.7E+08 | 19389000 | 10307000 | 9082000 | 0.182577382 |
| P62192 | PRS4_MOUSE | Psmc1 | 49.185 | 440 | 15 | 7 | 8 | 139400000 | 74055000 | 6.5E+07 | 5163100 | 2742800 | 2420400 | 0.18040991 |
| Q920E5 | FPPS_MOUSE | Fdps | 40.582 | 353 | 13 | 6 | 7 | 189320000 | 100440000 | 8.9E+07 | 9466100 | 5022100 | 4444000 | 0.17638698 |
| Q6NZJ6 | IF4G1_MOUSE | Eif4g1 | 176.077 | 1600 | 14 | 6 | 8 | 81185000 | 43062000 | 3.8E+07 | 1040800 | 552080 | 488760 | 0.175753673 |
| P70698 | PYRG1_MOUSE | Ctps1 | 66.682 | 591 | 12 | 6 | 6 | 116470000 | 61751000 | 5.5E+07 | 3327600 | 1764300 | 1563300 | 0.174526101 |
| Q9WUA2 | SYFB_MOUSE | Farsb | 65.697 | 589 | 14 | 7 | 7 | 59165000 | 31352000 | 2.8E+07 | 1443000 | 764690 | 678350 | 0.172849983 |
| Q923D2 | BLVRB_MOUSE | Blvrb | 22.197 | 206 | 8 | 4 | 4 | 80760000 | 42795000 | 3.8E+07 | 6212300 | 3291900 | 2920400 | 0.172772239 |
| Q9JKF7 | RM39_MOUSE | Mrpl39 | 38.549 | 336 | 4 | 2 | 2 | 10187000 | 5388200 | 4798600 | 442900 | 234270 | 208630 | 0.167189844 |
| P00397 | COX1_MOUSE | Mtco1 | 56.91 | 514 | 8 | 4 | 4 | 57208000 | 30258000 | 2.7E+07 | 11442000 | 6051600 | 5390000 | 0.167031358 |
| P59913 | PCMD1_MOUSE | Pcmtd1 | 40.693 | 357 | 2 | 1 | 1 | 7951500 | 4204100 | 3747400 | 361430 | 191100 | 170330 | 0.165907006 |
| Q9DCT5 | SDF2_MOUSE | Sdf2 | 23.159 | 211 | 6 | 3 | 3 | 58282000 | 30804000 | 2.7E+07 | 5298300 | 2800300 | 2498000 | 0.164840701 |
| Q03265 | ATPA_MOUSE | Atp5f1a | 59.753 | 553 | 51 | 25 | 26 | 3755200000 | 1984600000 | 1.8E+09 | 104310000 | 55129000 | 4.9E+07 | 0.164691415 |
| P07901 | HS90A_MOUSE | Hsp90aa1 | 84.788 | 733 | 23 | 12 | 11 | 195290000 | 103210000 | 9.2E+07 | 5139200 | 2715900 | 2423300 | 0.164560351 |
| P60843 | IF4A1_MOUSE | Eif4a1 | 46.154 | 406 | 20 | 10 | 10 | 192050000 | 101230000 | 9.1E+07 | 8002100 | 4217900 | 3784300 | 0.156507306 |
| Q61024 | ASNS_MOUSE | Asns | 64.283 | 561 | 12 | 6 | 6 | 65150000 | 34324000 | 3.1E+07 | 1916200 | 1009500 | 906650 | 0.155069993 |
| P68254 | 1433T_MOUSE | Ywhaq | 27.778 | 245 | 8 | 4 | 4 | 103490000 | 54463000 | 4.9E+07 | 5749600 | 3025700 | 2723900 | 0.151611691 |
| O08997 | ATOX1_MOUSE | Atox1 | 7.338 | 68 | 2 | 1 | 1 | 14354000 | 7545500 | 6808800 | 3588600 | 1886400 | 1702200 | 0.148215947 |
| Q8JZN5 | ACAD9_MOUSE | Acad9 | 68.722 | 625 | 45 | 22 | 23 | 1146100000 | 602180000 | 5.4E+08 | 30160000 | 15847000 | 1.4E+07 | 0.146853367 |
| O88455 | DHCR7_MOUSE | Dhcr7 | 53.919 | 471 | 2 | 1 | 1 | 5026400 | 2638800 | 2387500 | 335090 | 175920 | 159170 | 0.144381277 |
| Q9QUM9 | PSA6_MOUSE | Psma6 | 27.372 | 246 | 16 | 8 | 8 | 162100000 | 84970000 | 7.7E+07 | 9535600 | 4998200 | 4537300 | 0.139567933 |
| Q60973 | RBBP7_MOUSE | Rbbp7 | 47.79 | 425 | 5 | 3 | 2 | 40594000 | 21270000 | 1.9E+07 | 2255200 | 1181700 | 1073500 | 0.138426275 |
| Q9CRA4 | MSMO1_MOUSE | Msmo1 | 34.772 | 293 | 2 | 1 | 1 | 11735000 | 6147700 | 5586800 | 1466800 | 768460 | 698350 | 0.13802459 |
| P83940 | ELOC_MOUSE | Eloc | 12.473 | 112 | 10 | 5 | 5 | 244150000 | 127750000 | 1.2E+08 | 30519000 | 15969000 | 1.5E+07 | 0.134232233 |
| P13439 | UMPS_MOUSE | Umps | 52.292 | 481 | 10 | 5 | 5 | 83185000 | 43514000 | 4E+07 | 3327400 | 1740600 | 1586800 | 0.133431244 |
| Q8BFP9 | PDK1_MOUSE | Pdk1 | 48.995 | 434 | 2 | 1 | 1 | 3477900 | 1818000 | 1659800 | 112190 | 58646 | 53543 | 0.131342787 |
| Q3V3R1 | C1TM_MOUSE | Mthfd1l | 105.729 | 977 | 17 | 8 | 9 | 109970000 | 57482000 | 5.2E+07 | 1896000 | 991070 | 904950 | 0.131150119 |
| Q8CAQ8 | MIC60_MOUSE | Immt | 83.9 | 757 | 27 | 14 | 13 | 214760000 | 112080000 | 1E+08 | 3977000 | 2075500 | 1901500 | 0.12637366 |
| Q7TPV4 | MBB1A_MOUSE | Mybbp1a | 152.037 | 1344 | 36 | 19 | 17 | 253380000 | 132180000 | 1.2E+08 | 3128100 | 1631900 | 1496200 | 0.125233241 |
| Q9CZX8 | RS19_MOUSE | Rps19 | 16.085 | 145 | 14 | 7 | 7 | 1919000000 | 1000900000 | 9.2E+08 | 159920000 | 83405000 | 7.7E+07 | 0.124543208 |
| Q60972 | RBBP4_MOUSE | Rbbp4 | 47.656 | 425 | 12 | 6 | 6 | 123790000 | 64525000 | 5.9E+07 | 7281800 | 3795600 | 3486200 | 0.122677889 |
| P14115 | RL27A_MOUSE | Rpl27a | 16.605 | 148 | 8 | 4 | 4 | 189460000 | 98568000 | 9.1E+07 | 23683000 | 12321000 | 1.1E+07 | 0.116966034 |
| Q9Z2U0 | PSA7_MOUSE | Psma7 | 27.855 | 248 | 14 | 7 | 7 | 231590000 | 120450000 | 1.1E+08 | 14474000 | 7528200 | 6946200 | 0.116056248 |
| Q91VK1 | 5MP1_MOUSE | Bzw2 | 48.063 | 419 | 7 | 3 | 4 | 26351000 | 13700000 | 1.3E+07 | 850020 | 441920 | 408100 | 0.114924466 |
| P62267 | RS23_MOUSE | Rps23 | 15.808 | 143 | 10 | 5 | 5 | 927300000 | 482070000 | 4.5E+08 | 115910000 | 60259000 | 5.6E+07 | 0.114691845 |
| Q61753 | SERA_MOUSE | Phgdh | 56.586 | 533 | 38 | 21 | 17 | 1239000000 | 643790000 | 6E+08 | 49558000 | 25752000 | 2.4E+07 | 0.113288359 |
| Q810D6 | GRWD1_MOUSE | Grwd1 | 49.224 | 446 | 4 | 2 | 2 | 28943000 | 15035000 | 1.4E+07 | 1447100 | 751750 | 695390 | 0.112409896 |
| Q8VE22 | RT23_MOUSE | Mrps23 | 20.348 | 177 | 6 | 3 | 3 | 42423000 | 22036000 | 2E+07 | 3856600 | 2003300 | 1853300 | 0.112212873 |
| Q99020 | ROAA_MOUSE | Hnrnpab | 30.831 | 285 | 15 | 7 | 8 | 988960000 | 512840000 | 4.8E+08 | 70640000 | 36631000 | 3.4E+07 | 0.10718356 |
| O09061 | PSB1_MOUSE | Psmb1 | 26.372 | 240 | 12 | 7 | 5 | 158340000 | 81993000 | 7.6E+07 | 11310000 | 5856600 | 5453600 | 0.102853695 |
| P10852 | 4F2_MOUSE | Slc3a2 | 58.337 | 526 | 20 | 9 | 11 | 134410000 | 69503000 | 6.5E+07 | 4335800 | 2242000 | 2093700 | 0.098723402 |
| O89023 | TPP1_MOUSE | Tpp1 | 61.342 | 562 | 2 | 1 | 1 | 21742000 | 11232000 | 1.1E+07 | 905910 | 468010 | 437890 | 0.095989447 |
| Q9CXY6 | ILF2_MOUSE | Ilf2 | 43.062 | 390 | 19 | 10 | 9 | 289230000 | 149390000 | 1.4E+08 | 13773000 | 7113700 | 6659200 | 0.095306489 |
| Q9CWX2 | CIA30_MOUSE | Ndufaf1 | 37.81 | 328 | 12 | 6 | 6 | 75915000 | 39201000 | 3.7E+07 | 3163100 | 1633400 | 1529700 | 0.094599448 |
| Q9Z1Q9 | SYVC_MOUSE | Vars1 | 140.215 | 1263 | 24 | 11 | 13 | 207810000 | 107290000 | 1E+08 | 3056100 | 1577800 | 1478300 | 0.094033039 |
| Q9CQZ6 | NDUB3_MOUSE | Ndufb3 | 11.692 | 104 | 2 | 1 | 1 | 6574300 | 3393200 | 3181100 | 1314900 | 678630 | 636220 | 0.093120742 |
| Q8R3C0 | MCMBP_MOUSE | Mcmbp | 72.891 | 642 | 11 | 5 | 6 | 68038000 | 35053000 | 3.3E+07 | 1838900 | 947380 | 891490 | 0.087727817 |
| Q9WVG6 | CARM1_MOUSE | Carm1 | 65.854 | 608 | 20 | 9 | 11 | 176680000 | 90791000 | 8.6E+07 | 6543800 | 3362600 | 3181200 | 0.080042324 |
| P20664 | PRI1_MOUSE | Prim1 | 49.295 | 417 | 4 | 2 | 2 | 11040000 | 5671300 | 5368500 | 306660 | 157540 | 149130 | 0.079160429 |
| P24547 | IMDH2_MOUSE | Impdh2 | 55.815 | 514 | 4 | 2 | 2 | 15442000 | 7930200 | 7512200 | 571940 | 293710 | 278230 | 0.078121778 |
| Q99P72 | RTN4_MOUSE | Rtn4 | 126.613 | 1162 | 10 | 5 | 5 | 62268000 | 31959000 | 3E+07 | 1220900 | 626650 | 594290 | 0.076476014 |
| P84244 | H33_MOUSE | H3-3b | 15.328 | 136 | 2 | 1 | 1 | 36895000 | 18930000 | 1.8E+07 | 6149200 | 3155000 | 2994100 | 0.075485476 |
| Q61210 | ARHG1_MOUSE | Arhgef1 | 102.805 | 920 | 7 | 4 | 3 | 11933000 | 6122500 | 5810700 | 238670 | 122450 | 116210 | 0.075408897 |
| P30416 | FKBP4_MOUSE | Fkbp4 | 51.572 | 458 | 11 | 6 | 5 | 76184000 | 39058000 | 3.7E+07 | 2308600 | 1183600 | 1125000 | 0.073188191 |
| Q9D880 | TIM50_MOUSE | Timm50 | 39.776 | 353 | 14 | 7 | 7 | 416280000 | 213250000 | 2E+08 | 23127000 | 11847000 | 1.1E+07 | 0.070852824 |
| P62838 | UB2D2_MOUSE | Ube2d2 | 16.735 | 147 | 4 | 2 | 2 | 22741000 | 11642000 | 1.1E+07 | 4548200 | 2328500 | 2219700 | 0.068909225 |
| Q8K078 | SO4A1_MOUSE | Slco4a1 | 77.669 | 723 | 6 | 3 | 3 | 29411000 | 15056000 | 1.4E+07 | 1014200 | 519170 | 495010 | 0.068785203 |
| O88544 | CSN4_MOUSE | Cops4 | 46.285 | 406 | 11 | 5 | 6 | 48171000 | 24650000 | 2.4E+07 | 1720400 | 880340 | 840040 | 0.067638249 |
| Q9JJZ2 | TBA8_MOUSE | Tuba8 | 50.052 | 449 | 2 | 1 | 1 | 30166000 | 15429000 | 1.5E+07 | 1311600 | 670840 | 640720 | 0.066201694 |
| Q9D0L7 | ARM10_MOUSE | Armc10 | 33.311 | 306 | 6 | 3 | 3 | 21987000 | 11239000 | 1.1E+07 | 1832200 | 936620 | 895610 | 0.064579685 |
| P62264 | RS14_MOUSE | Rps14 | 16.273 | 151 | 18 | 9 | 9 | 1907200000 | 974180000 | 9.3E+08 | 317860000 | 162360000 | 1.6E+08 | 0.062357673 |
| P51912 | AAAT_MOUSE | Slc1a5 | 58.483 | 553 | 14 | 7 | 7 | 135620000 | 69195000 | 6.6E+07 | 6164400 | 3145200 | 3019200 | 0.059006629 |
| Q99KI3 | EMC3_MOUSE | Emc3 | 29.98 | 261 | 5 | 2 | 3 | 8916300 | 4542600 | 4373800 | 636880 | 324470 | 312410 | 0.054631022 |
| P53026 | RL10A_MOUSE | Rpl10a | 24.916 | 217 | 26 | 13 | 13 | 1378700000 | 702370000 | 6.8E+08 | 106050000 | 54029000 | 5.2E+07 | 0.054610536 |
| P51660 | DHB4_MOUSE | Hsd17b4 | 79.482 | 735 | 9 | 5 | 4 | 42681000 | 21725000 | 2.1E+07 | 948460 | 482780 | 465680 | 0.05199281 |
| O54692 | ZW10_MOUSE | Zw10 | 88.063 | 779 | 2 | 1 | 1 | 11032000 | 5612100 | 5419400 | 225130 | 114530 | 110600 | 0.050407581 |
| Q8CFE6 | S38A2_MOUSE | Slc38a2 | 55.503 | 504 | 6 | 3 | 3 | 47812000 | 24264000 | 2.4E+07 | 3677800 | 1866500 | 1811400 | 0.04321287 |
| Q9CPR8 | NSE3_MOUSE | Nsmce3 | 31.46 | 279 | 2 | 1 | 1 | 5194400 | 2635900 | 2558500 | 259720 | 131790 | 127930 | 0.042997405 |
| O35972 | RM23_MOUSE | Mrpl23 | 17.122 | 146 | 7 | 3 | 4 | 24973000 | 12669000 | 1.2E+07 | 2497300 | 1266900 | 1230400 | 0.042175245 |
| Q60749 | KHDR1_MOUSE | Khdrbs1 | 48.371 | 443 | 7 | 4 | 3 | 83467000 | 42343000 | 4.1E+07 | 3974600 | 2016300 | 1958300 | 0.04214289 |
| Q99PM9 | UCK2_MOUSE | Uck2 | 29.404 | 261 | 13 | 6 | 7 | 138090000 | 70018000 | 6.8E+07 | 8630800 | 4376100 | 4254700 | 0.040600776 |
| P57759 | ERP29_MOUSE | Erp29 | 28.823 | 262 | 10 | 5 | 5 | 186460000 | 94473000 | 9.2E+07 | 14343000 | 7267200 | 7076000 | 0.038456401 |
| Q9Z0M5 | LICH_MOUSE | Lipa | 45.325 | 397 | 2 | 1 | 1 | 5893500 | 2982900 | 2910600 | 280640 | 142040 | 138600 | 0.035399028 |
| Q9D1M0 | SEC13_MOUSE | Sec13 | 35.566 | 322 | 2 | 1 | 1 | 6741700 | 3409900 | 3331800 | 518600 | 262300 | 256290 | 0.033427629 |
| Q922B2 | SYDC_MOUSE | Dars1 | 57.147 | 501 | 8 | 4 | 4 | 41019000 | 20728000 | 2E+07 | 1051800 | 531480 | 520290 | 0.030740954 |
| Q99N96 | RM01_MOUSE | Mrpl1 | 37.597 | 336 | 6 | 3 | 3 | 29826000 | 15067000 | 1.5E+07 | 1355700 | 684870 | 670880 | 0.029797215 |
| Q8K0C9 | GMDS_MOUSE | Gmds | 41.985 | 372 | 4 | 2 | 2 | 8166300 | 4120700 | 4045600 | 272210 | 137360 | 134850 | 0.026535751 |
| O35887 | CALU_MOUSE | Calu | 37.064 | 315 | 24 | 12 | 12 | 272430000 | 137460000 | 1.3E+08 | 16025000 | 8085900 | 7939600 | 0.026373092 |
| P68373 | TBA1C_MOUSE | Tuba1c | 49.909 | 449 | 2 | 1 | 1 | 76370000 | 38514000 | 3.8E+07 | 3320400 | 1674500 | 1645900 | 0.024860988 |
| O55234 | PSB5_MOUSE | Psmb5 | 28.532 | 264 | 9 | 4 | 5 | 75646000 | 38143000 | 3.8E+07 | 4449800 | 2243700 | 2206100 | 0.024373843 |
| P11983 | TCPA_MOUSE | Tcp1 | 60.449 | 556 | 57 | 28 | 29 | 1377000000 | 694190000 | 6.8E+08 | 39343000 | 19834000 | 2E+07 | 0.023804139 |
| Q9CQW9 | IFM3_MOUSE | Ifitm3 | 14.954 | 137 | 14 | 7 | 7 | 441220000 | 222420000 | 2.2E+08 | 110300000 | 55605000 | 5.5E+07 | 0.023739721 |
| Q80U30 | CL16A_MOUSE | Clec16a | 116.232 | 1036 | 2 | 1 | 1 | 37024000 | 18663000 | 1.8E+07 | 787740 | 397080 | 390660 | 0.023536277 |
| P56480 | ATPB_MOUSE | Atp5f1b | 56.3 | 529 | 75 | 38 | 37 | 4806400000 | 2422600000 | 2.4E+09 | 160210000 | 80754000 | 7.9E+07 | 0.023353541 |
| P08030 | APT_MOUSE | Aprt | 19.724 | 180 | 6 | 3 | 3 | 30170000 | 15200000 | 1.5E+07 | 2155000 | 1085700 | 1069300 | 0.021900733 |
| P29758 | OAT_MOUSE | Oat | 48.355 | 439 | 28 | 13 | 15 | 518000000 | 260860000 | 2.6E+08 | 19185000 | 9661500 | 9523800 | 0.02072169 |
| O35643 | AP1B1_MOUSE | Ap1b1 | 103.935 | 943 | 10 | 5 | 5 | 44822000 | 22527000 | 2.2E+07 | 914730 | 459740 | 454990 | 0.014999709 |
| O35864 | CSN5_MOUSE | Cops5 | 37.549 | 334 | 8 | 4 | 4 | 47444000 | 23835000 | 2.4E+07 | 2372200 | 1191700 | 1180500 | 0.01374469 |
| Q9D5V5 | CUL5_MOUSE | Cul5 | 90.974 | 780 | 38 | 17 | 21 | 388000000 | 194830000 | 1.9E+08 | 7185200 | 3608000 | 3577200 | 0.012344785 |
| P56382 | ATP5E_MOUSE | Atp5f1e | 5.838 | 52 | 2 | 1 | 1 | 56252000 | 28246000 | 2.8E+07 | 14063000 | 7061500 | 7001500 | 0.012310635 |
| Q8BMF4 | ODP2_MOUSE | Dlat | 67.942 | 642 | 35 | 15 | 20 | 629270000 | 315740000 | 3.1E+08 | 17480000 | 8770400 | 8709200 | 0.010133549 |
| Q60864 | STIP1_MOUSE | Stip1 | 62.582 | 543 | 7 | 3 | 4 | 48254000 | 24211000 | 2.4E+07 | 1176900 | 590500 | 586420 | 0.010045747 |
| Q9CQN6 | TM14C_MOUSE | Tmem14c | 11.642 | 114 | 2 | 1 | 1 | 12332000 | 6177000 | 6155000 | 2055300 | 1029500 | 1025800 | 0.005147474 |
| Q9CQJ2 | PIHD1_MOUSE | Pih1d1 | 32.209 | 290 | 4 | 2 | 2 | 9157100 | 4586600 | 4570500 | 481950 | 241400 | 240550 | 0.005073094 |
| Q9D937 | CK098_MOUSE |  | 14.099 | 123 | 2 | 1 | 1 | 41655000 | 20863000 | 2.1E+07 | 5950700 | 2980400 | 2970300 | 0.004918087 |
| P14131 | RS16_MOUSE | Rps16 | 16.445 | 146 | 20 | 10 | 10 | 1393200000 | 697760000 | 7E+08 | 116100000 | 58146000 | 5.8E+07 | 0.004763356 |
| Q3UA16 | SPC25_MOUSE | Spc25 | 26.457 | 226 | 2 | 1 | 1 | 6331600 | 3169400 | 3162200 | 527630 | 264110 | 263520 | 0.003281132 |
| Q9D6K7 | TTC33_MOUSE | Ttc33 | 29.371 | 262 | 2 | 1 | 1 | 4118900 | 2060700 | 2058100 | 274590 | 137380 | 137210 | 0.001821408 |
| Q91V92 | ACLY_MOUSE | Acly | 119.728 | 1091 | 15 | 7 | 8 | 106340000 | 53155000 | 5.3E+07 | 1661600 | 830550 | 831030 | -0.00084113 |
| Q68FD5 | CLH1_MOUSE | Cltc | 191.557 | 1675 | 110 | 53 | 57 | 2417400000 | 1207300000 | 1.2E+09 | 24667000 | 12320000 | 1.2E+07 | -0.00334206 |
| Q8CH09 | SUGP2_MOUSE | Sugp2 | 118.103 | 1067 | 2 | 1 | 1 | 11520000 | 5747300 | 5773000 | 182860 | 91228 | 91634 | -0.00643687 |
| Q8R323 | RFC3_MOUSE | Rfc3 | 40.526 | 356 | 11 | 6 | 5 | 85350000 | 42527000 | 4.3E+07 | 3161100 | 1575100 | 1586000 | -0.01000678 |
| P43274 | H14_MOUSE | H1-4 | 21.977 | 219 | 18 | 9 | 9 | 3369000000 | 1678600000 | 1.7E+09 | 280750000 | 139880000 | 1.4E+08 | -0.01010619 |
| Q6PD26 | PIGS_MOUSE | Pigs | 61.711 | 555 | 13 | 6 | 7 | 66151000 | 32933000 | 3.3E+07 | 2067200 | 1029200 | 1038100 | -0.01243127 |
| Q6ZWV7 | RL35_MOUSE | Rpl35 | 14.553 | 123 | 6 | 3 | 3 | 397250000 | 197610000 | 2E+08 | 99312000 | 49402000 | 5E+07 | -0.01474485 |
| Q8BWL5 | RBMS3_MOUSE | Rbms3 | 46.927 | 431 | 4 | 2 | 2 | 20398000 | 10145000 | 1E+07 | 1133200 | 563610 | 569590 | -0.01527724 |
| Q8BKE6 | CP20A_MOUSE | Cyp20a1 | 52.149 | 462 | 10 | 5 | 5 | 44627000 | 22190000 | 2.2E+07 | 1487600 | 739670 | 747900 | -0.01597012 |
| O35638 | STAG2_MOUSE | Stag2 | 141.281 | 1231 | 2 | 1 | 1 | 4549600 | 2261800 | 2287700 | 68933 | 34270 | 34663 | -0.01642651 |
| Q8CBE3 | WDR37_MOUSE | Wdr37 | 55.046 | 496 | 2 | 1 | 1 | 4348100 | 2155800 | 2192300 | 144940 | 71861 | 73075 | -0.02422189 |
| Q80WJ7 | LYRIC_MOUSE | Mtdh | 63.846 | 579 | 16 | 9 | 7 | 127770000 | 63265000 | 6.5E+07 | 4914400 | 2433300 | 2481100 | -0.02807051 |
| Q8BMC4 | NOP9_MOUSE | Nop9 | 70.046 | 636 | 12 | 5 | 7 | 45337000 | 22384000 | 2.3E+07 | 1462500 | 722050 | 740420 | -0.03621486 |
| P62281 | RS11_MOUSE | Rps11 | 18.431 | 158 | 16 | 7 | 9 | 739040000 | 364820000 | 3.7E+08 | 73904000 | 36482000 | 3.7E+07 | -0.03666329 |
| P15864 | H12_MOUSE | H1-2 | 21.267 | 212 | 4 | 2 | 2 | 17141000 | 8456700 | 8684400 | 1558300 | 768790 | 789490 | -0.03833138 |
| Q9CYN2 | SPCS2_MOUSE | Spcs2 | 24.978 | 226 | 7 | 4 | 3 | 40176000 | 19816000 | 2E+07 | 2869700 | 1415400 | 1454300 | -0.03907179 |
| P40142 | TKT_MOUSE | Tkt | 67.63 | 623 | 48 | 25 | 23 | 867230000 | 426930000 | 4.4E+08 | 25507000 | 12557000 | 1.3E+07 | -0.0444873 |
| P97461 | RS5_MOUSE | Rps5 | 22.889 | 204 | 7 | 3 | 4 | 1103900000 | 543300000 | 5.6E+08 | 110390000 | 54330000 | 5.6E+07 | -0.04524843 |
| Q9CWZ7 | SNAG_MOUSE | Napg | 34.732 | 312 | 2 | 1 | 1 | 6215500 | 3051900 | 3163600 | 310780 | 152590 | 158180 | -0.05185951 |
| P97372 | PSME2_MOUSE | Psme2 | 27.057 | 239 | 9 | 5 | 4 | 48930000 | 23983000 | 2.5E+07 | 2718300 | 1332400 | 1385900 | -0.0568542 |
| Q9JKV1 | ADRM1_MOUSE | Adrm1 | 42.06 | 407 | 4 | 2 | 2 | 33879000 | 16601000 | 1.7E+07 | 2823300 | 1383400 | 1439800 | -0.05766608 |
| E9PVA8 | GCN1_MOUSE | Gcn1 | 293.021 | 2671 | 7 | 3 | 4 | 20656000 | 10118000 | 1.1E+07 | 118030 | 57815 | 60219 | -0.05867694 |
| P15532 | NDKA_MOUSE | Nme1 | 17.208 | 152 | 4 | 2 | 2 | 43677000 | 21390000 | 2.2E+07 | 3359700 | 1645400 | 1714400 | -0.05926595 |
| Q9CR21 | ACPM_MOUSE | Ndufab1 | 17.37 | 156 | 7 | 3 | 4 | 50065000 | 24507000 | 2.6E+07 | 5562700 | 2723000 | 2839700 | -0.06052461 |
| P70295 | AUP1_MOUSE | Aup1 | 46.121 | 410 | 6 | 3 | 3 | 46722000 | 22869000 | 2.4E+07 | 2031400 | 994290 | 1037100 | -0.06077744 |
| Q9JJA4 | WDR12_MOUSE | Wdr12 | 47.347 | 423 | 6 | 3 | 3 | 48834000 | 23886000 | 2.5E+07 | 2123200 | 1038500 | 1084700 | -0.06275889 |
| O88712 | CTBP1_MOUSE | Ctbp1 | 47.745 | 441 | 6 | 3 | 3 | 36264000 | 17723000 | 1.9E+07 | 1576700 | 770560 | 806120 | -0.06509623 |
| Q9D2G2 | ODO2_MOUSE | Dlst | 48.995 | 454 | 17 | 9 | 8 | 200750000 | 98054000 | 1E+08 | 7721200 | 3771300 | 3949900 | -0.06678779 |
| P97390 | VPS45_MOUSE | Vps45 | 65.053 | 570 | 4 | 2 | 2 | 8818800 | 4302900 | 4515800 | 232070 | 113230 | 118840 | -0.06967228 |
| Q3UX10 | TBAL3_MOUSE | Tubal3 | 49.988 | 446 | 2 | 1 | 1 | 14576000 | 7110100 | 7466300 | 607350 | 296260 | 311100 | -0.07052363 |
| Q91V04 | TRAM1_MOUSE | Tram1 | 43.039 | 374 | 16 | 7 | 9 | 214910000 | 104760000 | 1.1E+08 | 16532000 | 8058200 | 8473700 | -0.0725125 |
| Q8C1Q6 | SMIM4_MOUSE | Smim4 | 9.746 | 80 | 3 | 1 | 2 | 4072800 | 0 | 4072800 | 1357600 | 0 | 1357600 | -0.07376869 |
| P10126 | EF1A1_MOUSE | Eef1a1 | 50.114 | 462 | 66 | 35 | 31 | 1.313E+10 | 6397000000 | 6.7E+09 | 525190000 | 255880000 | 2.7E+08 | -0.07381113 |
| Q91VR2 | ATPG_MOUSE | Atp5f1c | 32.886 | 298 | 10 | 5 | 5 | 286750000 | 139580000 | 1.5E+08 | 17922000 | 8723600 | 9198000 | -0.07639138 |
| Q9D0J4 | ARL2_MOUSE | Arl2 | 20.864 | 184 | 5 | 2 | 3 | 18476000 | 8993300 | 9483000 | 1847600 | 899330 | 948300 | -0.07649294 |
| Q9JHU9 | INO1_MOUSE | Isyna1 | 60.932 | 557 | 10 | 5 | 5 | 91401000 | 44457000 | 4.7E+07 | 3656100 | 1778300 | 1877800 | -0.07856091 |
| Q9Z204 | HNRPC_MOUSE | Hnrnpc | 34.385 | 313 | 10 | 4 | 6 | 75340000 | 36630000 | 3.9E+07 | 3424600 | 1665000 | 1759600 | -0.07968061 |
| Q99JB2 | STML2_MOUSE | Stoml2 | 38.385 | 353 | 7 | 3 | 4 | 47203000 | 22945000 | 2.4E+07 | 2360200 | 1147300 | 1212900 | -0.0802808 |
| A2A432 | CUL4B_MOUSE | Cul4b | 110.699 | 970 | 4 | 2 | 2 | 15442000 | 7501600 | 7940200 | 241280 | 117210 | 124070 | -0.08197701 |
| Q60865 | CAPR1_MOUSE | Caprin1 | 78.169 | 707 | 10 | 5 | 5 | 167370000 | 81241000 | 8.6E+07 | 7276800 | 3532200 | 3744500 | -0.08420733 |
| P97470 | PP4C_MOUSE | Ppp4c | 35.08 | 307 | 2 | 1 | 1 | 4667800 | 2261100 | 2406700 | 259320 | 125620 | 133710 | -0.09003152 |
| Q6A0A2 | LAR4B_MOUSE | Larp4b | 81.627 | 741 | 9 | 4 | 5 | 28118000 | 13602000 | 1.5E+07 | 685800 | 331750 | 354060 | -0.09382517 |
| E9Q3S4 | M3K19_MOUSE | Map3k19 | 146.412 | 1311 | 2 | 1 | 1 | 35559000 | 17195000 | 1.8E+07 | 439000 | 212280 | 226720 | -0.09489122 |
| Q3UVL4 | VPS51_MOUSE | Vps51 | 86.187 | 782 | 2 | 1 | 1 | 3491500 | 1688200 | 1803300 | 81197 | 39260 | 41937 | -0.0951536 |
| Q8BWW4 | LARP4_MOUSE | Larp4 | 79.763 | 719 | 13 | 6 | 7 | 81659000 | 39457000 | 4.2E+07 | 2474500 | 1195700 | 1278800 | -0.0970301 |
| Q3TXS7 | PSMD1_MOUSE | Psmd1 | 105.73 | 953 | 35 | 18 | 17 | 345660000 | 166940000 | 1.8E+08 | 6777700 | 3273200 | 3504500 | -0.09845214 |
| Q8VDT9 | RM50_MOUSE | Mrpl50 | 18.213 | 159 | 8 | 4 | 4 | 84514000 | 40800000 | 4.4E+07 | 7683100 | 3709100 | 3973900 | -0.09949324 |
| Q8BFY9 | TNPO1_MOUSE | Tnpo1 | 102.357 | 898 | 13 | 6 | 7 | 53911000 | 26019000 | 2.8E+07 | 1198000 | 578190 | 619840 | -0.10033759 |
| Q9CZ30 | OLA1_MOUSE | Ola1 | 44.73 | 396 | 2 | 1 | 1 | 3751500 | 1806400 | 1945100 | 133980 | 64516 | 69466 | -0.10672694 |
| Q8VDI7 | UBAC1_MOUSE | Ubac1 | 45.531 | 409 | 2 | 1 | 1 | 4228200 | 2035200 | 2193000 | 192190 | 92508 | 99683 | -0.10773524 |
| P60867 | RS20_MOUSE | Rps20 | 13.373 | 119 | 10 | 5 | 5 | 998590000 | 480480000 | 5.2E+08 | 166430000 | 80081000 | 8.6E+07 | -0.10878205 |
| P97370 | AT1B3_MOUSE | Atp1b3 | 31.776 | 278 | 14 | 7 | 7 | 179150000 | 86130000 | 9.3E+07 | 11943000 | 5742000 | 6201400 | -0.11105613 |
| Q9ERD8 | PARVG_MOUSE | Parvg | 37.603 | 331 | 6 | 3 | 3 | 21514000 | 10331000 | 1.1E+07 | 1195200 | 573930 | 621290 | -0.11432736 |
| Q920Q6 | MSI2H_MOUSE | Msi2 | 36.939 | 346 | 5 | 2 | 3 | 14308000 | 6868200 | 7440000 | 1100600 | 528320 | 572310 | -0.11537057 |
| Q921H8 | THIKA_MOUSE | Acaa1a | 43.953 | 424 | 11 | 5 | 6 | 73137000 | 35057000 | 3.8E+07 | 3179900 | 1524200 | 1655600 | -0.11929305 |
| Q9ESZ8 | GTF2I_MOUSE | Gtf2i | 112.265 | 998 | 8 | 4 | 4 | 34111000 | 16297000 | 1.8E+07 | 559190 | 267160 | 292040 | -0.12840509 |
| Q9D338 | RM19_MOUSE | Mrpl19 | 33.578 | 292 | 10 | 5 | 5 | 67799000 | 32379000 | 3.5E+07 | 3081800 | 1471800 | 1610000 | -0.12950578 |
| P47915 | RL29_MOUSE | Rpl29 | 17.587 | 160 | 2 | 1 | 1 | 362350000 | 173040000 | 1.9E+08 | 60391000 | 28840000 | 3.2E+07 | -0.12956884 |
| Q8BU88 | RM22_MOUSE | Mrpl22 | 23.805 | 206 | 4 | 2 | 2 | 17334000 | 8245600 | 9088600 | 963010 | 458090 | 504920 | -0.1404336 |
| P62869 | ELOB_MOUSE | Elob | 13.17 | 118 | 17 | 8 | 9 | 807120000 | 383010000 | 4.2E+08 | 73374000 | 34819000 | 3.9E+07 | -0.14705644 |
| Q60692 | PSB6_MOUSE | Psmb6 | 25.379 | 238 | 6 | 3 | 3 | 102260000 | 48522000 | 5.4E+07 | 9296400 | 4411000 | 4885300 | -0.14733046 |
| Q9D0W5 | PPIL1_MOUSE | Ppil1 | 18.237 | 166 | 6 | 3 | 3 | 51400000 | 24373000 | 2.7E+07 | 5140000 | 2437300 | 2702800 | -0.14917092 |
| P28574 | MAX_MOUSE | Max | 18.245 | 160 | 6 | 3 | 3 | 49029000 | 23212000 | 2.6E+07 | 4457200 | 2110200 | 2347000 | -0.15345053 |
| Q9JKR6 | HYOU1_MOUSE | Hyou1 | 111.181 | 999 | 23 | 11 | 12 | 112230000 | 53122000 | 5.9E+07 | 2040500 | 965860 | 1074600 | -0.15394631 |
| P45952 | ACADM_MOUSE | Acadm | 46.481 | 421 | 12 | 7 | 5 | 73558000 | 34786000 | 3.9E+07 | 3343500 | 1581200 | 1762400 | -0.15650836 |
| P24288 | BCAT1_MOUSE | Bcat1 | 42.791 | 386 | 7 | 4 | 3 | 59915000 | 28313000 | 3.2E+07 | 3328600 | 1572900 | 1755700 | -0.15855124 |
| Q8R010 | AIMP2_MOUSE | Aimp2 | 35.378 | 320 | 6 | 3 | 3 | 31871000 | 15058000 | 1.7E+07 | 1874800 | 885790 | 989000 | -0.15904701 |
| Q921F2 | TADBP_MOUSE | Tardbp | 44.548 | 414 | 5 | 2 | 3 | 52398000 | 24749000 | 2.8E+07 | 3082300 | 1455800 | 1626400 | -0.15985707 |
| Q9D6M3 | GHC1_MOUSE | Slc25a22 | 34.67 | 323 | 3 | 1 | 2 | 4247000 | 2004700 | 2242300 | 223530 | 105510 | 118020 | -0.16159296 |
| P47802 | MTX1_MOUSE | Mtx1 | 35.624 | 317 | 2 | 1 | 1 | 5914600 | 2791800 | 3122800 | 295730 | 139590 | 156140 | -0.16164458 |
| P35979 | RL12_MOUSE | Rpl12 | 17.805 | 165 | 14 | 7 | 7 | 3287300000 | 1551300000 | 1.7E+09 | 298840000 | 141030000 | 1.6E+08 | -0.16228924 |
| P59759 | MRTFB_MOUSE | Mrtfb | 117.547 | 1080 | 2 | 1 | 1 | 12183000 | 5740500 | 6442900 | 297150 | 140010 | 157140 | -0.1665338 |
| P07356 | ANXA2_MOUSE | Anxa2 | 38.676 | 339 | 6 | 3 | 3 | 19025000 | 8961600 | 1E+07 | 761010 | 358470 | 402550 | -0.16737559 |
| Q8K1M6 | DNM1L_MOUSE | Dnm1l | 82.658 | 742 | 2 | 1 | 1 | 6604700 | 3111000 | 3493700 | 122310 | 57611 | 64699 | -0.16737734 |
| P28650 | PURA1_MOUSE | Adss1 | 50.254 | 457 | 2 | 1 | 1 | 2705700 | 1273800 | 1432000 | 96633 | 45492 | 51142 | -0.16889272 |
| Q9Z2I8 | SUCB2_MOUSE | Suclg2 | 46.84 | 433 | 15 | 7 | 8 | 101280000 | 47655000 | 5.4E+07 | 3267100 | 1537300 | 1729800 | -0.17025125 |
| Q9WV55 | VAPA_MOUSE | Vapa | 27.855 | 249 | 10 | 4 | 6 | 202670000 | 95348000 | 1.1E+08 | 11260000 | 5297100 | 5962400 | -0.17064438 |
| Q9DCZ4 | MIC26_MOUSE | Apoo | 22.604 | 198 | 2 | 1 | 1 | 2798800 | 1314700 | 1484000 | 233230 | 109560 | 123670 | -0.17475746 |
| Q8CGC7 | SYEP_MOUSE | Eprs1 | 170.079 | 1512 | 13 | 6 | 7 | 70971000 | 33282000 | 3.8E+07 | 702680 | 329530 | 373160 | -0.17940139 |
| P50544 | ACADV_MOUSE | Acadvl | 70.876 | 656 | 2 | 1 | 1 | 4424900 | 2074800 | 2350100 | 98331 | 46107 | 52225 | -0.17974987 |
| Q61881 | MCM7_MOUSE | Mcm7 | 81.211 | 719 | 19 | 9 | 10 | 87875000 | 41195000 | 4.7E+07 | 1910300 | 895550 | 1014800 | -0.18030441 |
| Q9Z130 | HNRDL_MOUSE | Hnrnpdl | 33.559 | 301 | 3 | 1 | 2 | 13577000 | 6364800 | 7212700 | 905160 | 424320 | 480840 | -0.18042424 |
| P80314 | TCPB_MOUSE | Cct2 | 57.477 | 535 | 56 | 28 | 28 | 1461200000 | 684220000 | 7.8E+08 | 40588000 | 19006000 | 2.2E+07 | -0.18334291 |
| Q8C3R1 | BRAT1_MOUSE | Brat1 | 89.086 | 822 | 2 | 1 | 1 | 2665500 | 1246300 | 1419300 | 63465 | 29673 | 33792 | -0.18752818 |
| Q33DR3 | DLP1_MOUSE | Pdss2 | 43.98 | 401 | 9 | 4 | 5 | 55029000 | 25708000 | 2.9E+07 | 1897500 | 886480 | 1011100 | -0.18971693 |
| P53395 | ODB2_MOUSE | Dbt | 53.247 | 482 | 10 | 4 | 6 | 52759000 | 24637000 | 2.8E+07 | 1648700 | 769910 | 878800 | -0.19087261 |
| Q91YD3 | DCP1A_MOUSE | Dcp1a | 65.219 | 602 | 6 | 3 | 3 | 27475000 | 12821000 | 1.5E+07 | 981260 | 457880 | 523380 | -0.19288418 |
| P18242 | CATD_MOUSE | Ctsd | 44.954 | 410 | 3 | 1 | 2 | 18848000 | 8789500 | 1E+07 | 942410 | 439470 | 502940 | -0.19463389 |
| Q9D6R2 | IDH3A_MOUSE | Idh3a | 39.639 | 366 | 4 | 2 | 2 | 55812000 | 26025000 | 3E+07 | 2536900 | 1182900 | 1354000 | -0.19478467 |
| Q9D1H8 | RM53_MOUSE | Mrpl53 | 12.738 | 118 | 2 | 1 | 1 | 9360500 | 4361300 | 4999200 | 1040100 | 484590 | 555470 | -0.19693901 |
| Q9CXT8 | MPPB_MOUSE | Pmpcb | 54.614 | 489 | 8 | 4 | 4 | 50638000 | 23585000 | 2.7E+07 | 2025500 | 943410 | 1082100 | -0.19791899 |
| P07607 | TYSY_MOUSE | Tyms | 34.958 | 307 | 2 | 1 | 1 | 3917000 | 1824200 | 2092700 | 217610 | 101350 | 116260 | -0.1981016 |
| Q61699 | HS105_MOUSE | Hsph1 | 96.407 | 858 | 23 | 11 | 12 | 171060000 | 79600000 | 9.1E+07 | 3110100 | 1447300 | 1662800 | -0.20030939 |
| Q8K2A7 | INT10_MOUSE | Ints10 | 82.02 | 710 | 5 | 2 | 3 | 22538000 | 10484000 | 1.2E+07 | 479530 | 223060 | 256470 | -0.20132271 |
| Q9ERN0 | SCAM2_MOUSE | Scamp2 | 36.465 | 329 | 4 | 2 | 2 | 8535600 | 3969900 | 4565700 | 853560 | 396990 | 456570 | -0.2017334 |
| P26638 | SYSC_MOUSE | Sars1 | 58.389 | 512 | 6 | 3 | 3 | 21895000 | 10182000 | 1.2E+07 | 729830 | 339400 | 390430 | -0.20208966 |
| Q62465 | VAT1_MOUSE | Vat1 | 43.097 | 406 | 10 | 5 | 5 | 37183000 | 17270000 | 2E+07 | 1957000 | 908920 | 1048100 | -0.2054425 |
| Q62186 | SSRD_MOUSE | Ssr4 | 18.937 | 172 | 12 | 5 | 7 | 394680000 | 183250000 | 2.1E+08 | 49335000 | 22906000 | 2.6E+07 | -0.2063669 |
| P61514 | RL37A_MOUSE | Rpl37a | 10.275 | 92 | 7 | 3 | 4 | 510090000 | 236820000 | 2.7E+08 | 85015000 | 39470000 | 4.6E+07 | -0.20653616 |
| O55126 | NIPS2_MOUSE | Nipsnap2 | 32.933 | 281 | 6 | 3 | 3 | 69164000 | 32103000 | 3.7E+07 | 3640200 | 1689600 | 1950600 | -0.20719369 |
| Q9CQR2 | RS21_MOUSE | Rps21 | 9.141 | 83 | 10 | 5 | 5 | 265370000 | 123050000 | 1.4E+08 | 37910000 | 17578000 | 2E+07 | -0.20989376 |
| P32233 | DRG1_MOUSE | Drg1 | 40.512 | 367 | 14 | 7 | 7 | 97554000 | 45232000 | 5.2E+07 | 3902200 | 1809300 | 2092900 | -0.2100739 |
| Q8K273 | EMC5_MOUSE | Mmgt1 | 14.677 | 131 | 2 | 1 | 1 | 11812000 | 5474900 | 6336700 | 1968600 | 912480 | 1056100 | -0.2108991 |
| O08583 | THOC4_MOUSE | Alyref | 26.94 | 255 | 18 | 9 | 9 | 551250000 | 255420000 | 3E+08 | 36750000 | 17028000 | 2E+07 | -0.21194563 |
| Q9CQ43 | DUT_MOUSE | Dut | 17.385 | 162 | 16 | 9 | 7 | 420910000 | 194870000 | 2.3E+08 | 38265000 | 17715000 | 2.1E+07 | -0.21406609 |
| O08784 | TCOF_MOUSE | Tcof1 | 135.001 | 1320 | 7 | 3 | 4 | 86461000 | 40023000 | 4.6E+07 | 1184400 | 548260 | 636130 | -0.21444546 |
| Q9CWK3 | CD2B2_MOUSE | Cd2bp2 | 37.694 | 342 | 5 | 2 | 3 | 15756000 | 7279200 | 8476800 | 1212000 | 559940 | 652060 | -0.21973985 |
| Q9CQN7 | RM41_MOUSE | Mrpl41 | 15.262 | 135 | 8 | 4 | 4 | 45123000 | 20846000 | 2.4E+07 | 5013700 | 2316200 | 2697500 | -0.21981957 |
| Q3THW5 | H2AV_MOUSE | H2az2 | 13.509 | 128 | 2 | 1 | 1 | 23291000 | 10760000 | 1.3E+07 | 3881800 | 1793300 | 2088500 | -0.21982347 |
| Q921T2 | TOIP1_MOUSE | Tor1aip1 | 66.781 | 595 | 9 | 4 | 5 | 56415000 | 26035000 | 3E+07 | 1659300 | 765730 | 893530 | -0.22266946 |
| Q9WTZ1 | RBX2_MOUSE | Rnf7 | 12.707 | 113 | 4 | 2 | 2 | 17508000 | 8077000 | 9431100 | 2918000 | 1346200 | 1571800 | -0.22360651 |
| Q99N93 | RM16_MOUSE | Mrpl16 | 28.804 | 251 | 6 | 3 | 3 | 44045000 | 20317000 | 2.4E+07 | 3146100 | 1451200 | 1694800 | -0.22390311 |
| Q922J9 | FACR1_MOUSE | Far1 | 59.435 | 515 | 21 | 10 | 11 | 208920000 | 96302000 | 1.1E+08 | 6528900 | 3009400 | 3519400 | -0.22582539 |
| Q62193 | RFA2_MOUSE | Rpa2 | 29.718 | 270 | 18 | 9 | 9 | 322020000 | 148380000 | 1.7E+08 | 20126000 | 9274100 | 1.1E+07 | -0.22680268 |
| P60334 | CDO1_MOUSE | Cdo1 | 23.026 | 200 | 4 | 2 | 2 | 90931000 | 41893000 | 4.9E+07 | 9093100 | 4189300 | 4903800 | -0.22719094 |
| O35387 | HAX1_MOUSE | Hax1 | 31.654 | 280 | 21 | 10 | 11 | 308770000 | 142120000 | 1.7E+08 | 17154000 | 7895600 | 9258000 | -0.22962515 |
| P84104 | SRSF3_MOUSE | Srsf3 | 19.33 | 164 | 6 | 3 | 3 | 68955000 | 31732000 | 3.7E+07 | 7661600 | 3525800 | 4135800 | -0.23021712 |
| P51859 | HDGF_MOUSE | Hdgf | 26.269 | 237 | 4 | 2 | 2 | 23643000 | 10880000 | 1.3E+07 | 1477700 | 679980 | 797700 | -0.23028892 |
| Q6P5F9 | XPO1_MOUSE | Xpo1 | 123.093 | 1071 | 22 | 10 | 12 | 89738000 | 41282000 | 4.8E+07 | 1547200 | 711760 | 835460 | -0.23116245 |
| P26516 | PSMD7_MOUSE | Psmd7 | 36.54 | 321 | 18 | 9 | 9 | 161330000 | 74212000 | 8.7E+07 | 10755000 | 4947500 | 5807800 | -0.23130179 |
| Q9D8N0 | EF1G_MOUSE | Eef1g | 50.061 | 437 | 36 | 18 | 18 | 1398000000 | 641100000 | 7.6E+08 | 53770000 | 24658000 | 2.9E+07 | -0.23957236 |
| Q68FL6 | SYMC_MOUSE | Mars1 | 101.431 | 902 | 38 | 18 | 20 | 330880000 | 151440000 | 1.8E+08 | 6617700 | 3028900 | 3588800 | -0.24475521 |
| Q923T9 | KCC2G_MOUSE | Camk2g | 59.607 | 529 | 11 | 6 | 5 | 63540000 | 29040000 | 3.4E+07 | 2269300 | 1037200 | 1232100 | -0.24851309 |
| Q9QZD9 | EIF3I_MOUSE | Eif3i | 36.461 | 325 | 18 | 8 | 10 | 326820000 | 149360000 | 1.8E+08 | 14856000 | 6789000 | 8066600 | -0.24870004 |
| Q9Z127 | LAT1_MOUSE | Slc7a5 | 55.872 | 512 | 4 | 2 | 2 | 19209000 | 8766500 | 1E+07 | 1067200 | 487030 | 580160 | -0.25246335 |
| Q8BHG9 | CGBP1_MOUSE | Cggbp1 | 18.761 | 167 | 2 | 1 | 1 | 13694000 | 6246200 | 7447600 | 1141100 | 520520 | 620630 | -0.25379683 |
| P50580 | PA2G4_MOUSE | Pa2g4 | 43.699 | 394 | 32 | 15 | 17 | 738610000 | 336840000 | 4E+08 | 30775000 | 14035000 | 1.7E+07 | -0.25430637 |
| Q99J62 | RFC4_MOUSE | Rfc4 | 39.867 | 364 | 18 | 9 | 9 | 116770000 | 53251000 | 6.4E+07 | 4865400 | 2218800 | 2646600 | -0.25437958 |
| Q61249 | IGBP1_MOUSE | Igbp1 | 38.971 | 340 | 6 | 3 | 3 | 21638000 | 9867000 | 1.2E+07 | 983570 | 448500 | 535070 | -0.25467603 |
| Q9CPR1 | RWDD4_MOUSE | Rwdd4 | 21.138 | 188 | 4 | 2 | 2 | 17328000 | 7901000 | 9426700 | 2475400 | 1128700 | 1346700 | -0.25471755 |
| Q9Z2U1 | PSA5_MOUSE | Psma5 | 26.411 | 241 | 21 | 11 | 10 | 649620000 | 295490000 | 3.5E+08 | 54135000 | 24625000 | 3E+07 | -0.26116976 |
| Q9ES46 | PARVB_MOUSE | Parvb | 41.669 | 365 | 2 | 1 | 1 | 19494000 | 8867000 | 1.1E+07 | 974680 | 443350 | 531330 | -0.2612164 |
| Q80YQ2 | MED23_MOUSE | Med23 | 156.087 | 1367 | 2 | 1 | 1 | 3621500 | 1643600 | 1977800 | 47032 | 21346 | 25686 | -0.26703731 |
| Q3TBW2 | RM10_MOUSE | Mrpl10 | 29.396 | 262 | 6 | 3 | 3 | 30450000 | 13803000 | 1.7E+07 | 1602600 | 726450 | 876170 | -0.27028035 |
| Q3UGC7 | EI3JA_MOUSE | Eif3j1 | 29.344 | 261 | 12 | 6 | 6 | 67077000 | 30399000 | 3.7E+07 | 4192300 | 1899900 | 2292400 | -0.27093044 |
| Q9EPL8 | IPO7_MOUSE | Ipo7 | 119.486 | 1038 | 20 | 10 | 10 | 132950000 | 60216000 | 7.3E+07 | 2828700 | 1281200 | 1547600 | -0.27250288 |
| Q01768 | NDKB_MOUSE | Nme2 | 17.363 | 152 | 23 | 12 | 11 | 587730000 | 266080000 | 3.2E+08 | 41980000 | 19006000 | 2.3E+07 | -0.27363161 |
| Q61425 | HCDH_MOUSE | Hadh | 34.464 | 314 | 5 | 2 | 3 | 21691000 | 9819000 | 1.2E+07 | 1141600 | 516790 | 624830 | -0.27391499 |
| Q99N89 | RM43_MOUSE | Mrpl43 | 20.203 | 183 | 6 | 3 | 3 | 63748000 | 28834000 | 3.5E+07 | 4903700 | 2218000 | 2685700 | -0.27603466 |
| P17742 | PPIA_MOUSE | Ppia | 17.971 | 164 | 9 | 4 | 5 | 101920000 | 46065000 | 5.6E+07 | 7280300 | 3290400 | 3990000 | -0.27814456 |
| P62830 | RL23_MOUSE | Rpl23 | 14.865 | 140 | 13 | 7 | 6 | 478860000 | 216390000 | 2.6E+08 | 53207000 | 24043000 | 2.9E+07 | -0.27857367 |
| Q8BML9 | SYQ_MOUSE | Qars1 | 87.677 | 775 | 26 | 14 | 12 | 203540000 | 91806000 | 1.1E+08 | 3990900 | 1800100 | 2190800 | -0.28335626 |
| Q60787 | LCP2_MOUSE | Lcp2 | 60.238 | 533 | 4 | 2 | 2 | 14200000 | 6401900 | 7797800 | 507130 | 228640 | 278490 | -0.28456701 |
| Q9Z2D8 | MBD3_MOUSE | Mbd3 | 32.168 | 285 | 6 | 3 | 3 | 25995000 | 11717000 | 1.4E+07 | 1444200 | 650920 | 793250 | -0.28529171 |
| P11440 | CDK1_MOUSE | Cdk1 | 34.107 | 297 | 24 | 12 | 12 | 473150000 | 212950000 | 2.6E+08 | 22531000 | 10141000 | 1.2E+07 | -0.28910623 |
| Q6PB66 | LPPRC_MOUSE | Lrpprc | 156.615 | 1392 | 37 | 18 | 19 | 256770000 | 115390000 | 1.4E+08 | 2517300 | 1131200 | 1386100 | -0.29305985 |
| Q9DCW4 | ETFB_MOUSE | Etfb | 27.623 | 255 | 11 | 5 | 6 | 85499000 | 38401000 | 4.7E+07 | 6107100 | 2742900 | 3364100 | -0.29452192 |
| P48453 | PP2BB_MOUSE | Ppp3cb | 59.174 | 525 | 6 | 3 | 3 | 16284000 | 7310700 | 8973200 | 603110 | 270770 | 332340 | -0.29561302 |
| P97300 | NPTN_MOUSE | Nptn | 44.373 | 397 | 5 | 2 | 3 | 53907000 | 24198000 | 3E+07 | 2450300 | 1099900 | 1350400 | -0.29601223 |
| Q921Y0 | MOB1A_MOUSE | Mob1a | 25.08 | 216 | 2 | 1 | 1 | 7320600 | 3280000 | 4040600 | 732060 | 328000 | 404060 | -0.30087372 |
| Q6PDM2 | SRSF1_MOUSE | Srsf1 | 27.745 | 248 | 13 | 6 | 7 | 157840000 | 70634000 | 8.7E+07 | 10523000 | 4709000 | 5814000 | -0.30413077 |
| P80316 | TCPE_MOUSE | Cct5 | 59.624 | 541 | 51 | 24 | 27 | 799220000 | 357540000 | 4.4E+08 | 21601000 | 9663300 | 1.2E+07 | -0.30489685 |
| P26443 | DHE3_MOUSE | Glud1 | 61.337 | 558 | 37 | 18 | 19 | 608490000 | 272100000 | 3.4E+08 | 16446000 | 7354000 | 9091700 | -0.30599786 |
| Q8VI93 | OAS3_MOUSE | Oas3 | 126.333 | 1138 | 7 | 3 | 4 | 36803000 | 16451000 | 2E+07 | 584170 | 261130 | 323040 | -0.3069244 |
| Q9CZN7 | GLYM_MOUSE | Shmt2 | 55.759 | 504 | 62 | 31 | 31 | 6779300000 | 3026700000 | 3.8E+09 | 218690000 | 97637000 | 1.2E+08 | -0.31010639 |
| Q8BU30 | SYIC_MOUSE | Iars1 | 144.271 | 1262 | 22 | 10 | 12 | 142610000 | 63573000 | 7.9E+07 | 2128600 | 948850 | 1179700 | -0.31420528 |
| Q9CPV1 | SKA1_MOUSE | Ska1 | 29.445 | 254 | 2 | 1 | 1 | 4440600 | 1974400 | 2466200 | 246700 | 109690 | 137010 | -0.3208755 |
| Q6P9P6 | KIF11_MOUSE | Kif11 | 118.027 | 1052 | 8 | 4 | 4 | 25388000 | 11270000 | 1.4E+07 | 338500 | 150270 | 188230 | -0.32504821 |
| Q8R0X7 | SGPL1_MOUSE | Sgpl1 | 63.677 | 568 | 5 | 2 | 3 | 17216000 | 7630900 | 9584800 | 521690 | 231240 | 290450 | -0.32889511 |
| P20108 | PRDX3_MOUSE | Prdx3 | 28.127 | 257 | 17 | 8 | 9 | 433350000 | 192050000 | 2.4E+08 | 25491000 | 11297000 | 1.4E+07 | -0.32934595 |
| P63330 | PP2AA_MOUSE | Ppp2ca | 35.608 | 309 | 23 | 11 | 12 | 241540000 | 107040000 | 1.3E+08 | 13419000 | 5946900 | 7471900 | -0.32945615 |
| P17426 | AP2A1_MOUSE | Ap2a1 | 107.664 | 977 | 18 | 9 | 9 | 125060000 | 55293000 | 7E+07 | 2084300 | 921560 | 1162700 | -0.33534455 |
| Q8R1Q8 | DC1L1_MOUSE | Dync1li1 | 56.614 | 523 | 17 | 8 | 9 | 103650000 | 45746000 | 5.8E+07 | 3140800 | 1386200 | 1754500 | -0.33991775 |
| Q9BDB7 | IF44L_MOUSE | Ifi44l | 49.68 | 447 | 12 | 6 | 6 | 166060000 | 73198000 | 9.3E+07 | 7548000 | 3327200 | 4220800 | -0.34322198 |
| P43275 | H11_MOUSE | H1-1 | 21.785 | 213 | 3 | 1 | 2 | 200180000 | 87989000 | 1.1E+08 | 25023000 | 10999000 | 1.4E+07 | -0.35054901 |
| Q61598 | GDIB_MOUSE | Gdi2 | 50.537 | 445 | 4 | 2 | 2 | 13997000 | 6146500 | 7850600 | 482660 | 211950 | 270710 | -0.35303779 |
| Q99L45 | IF2B_MOUSE | Eif2s2 | 38.092 | 331 | 22 | 11 | 11 | 288030000 | 126380000 | 1.6E+08 | 13092000 | 5744500 | 7347900 | -0.35510534 |
| Q99M31 | HSP7E_MOUSE | Hspa14 | 54.65 | 509 | 20 | 10 | 10 | 157170000 | 68957000 | 8.8E+07 | 4911700 | 2154900 | 2756800 | -0.35535969 |
| P38647 | GRP75_MOUSE | Hspa9 | 73.461 | 679 | 86 | 40 | 46 | 7635600000 | 3343700000 | 4.3E+09 | 173540000 | 75994000 | 9.8E+07 | -0.36013743 |
| Q8VI75 | IPO4_MOUSE | Ipo4 | 119.275 | 1082 | 10 | 5 | 5 | 48100000 | 21021000 | 2.7E+07 | 981640 | 428990 | 552650 | -0.36539644 |
| Q99M08 | CD003_MOUSE |  | 7.404 | 65 | 2 | 1 | 1 | 12273000 | 5361900 | 6911600 | 3068400 | 1340500 | 1727900 | -0.36627541 |
| Q9D7N3 | RT09_MOUSE | Mrps9 | 44.929 | 390 | 12 | 6 | 6 | 51198000 | 22352000 | 2.9E+07 | 1706600 | 745060 | 961550 | -0.36801736 |
| P52480 | KPYM_MOUSE | Pkm | 57.845 | 531 | 125 | 59 | 66 | 1.9919E+10 | 8694400000 | 1.1E+10 | 524190000 | 228800000 | 3E+08 | -0.36855707 |
| Q62448 | IF4G2_MOUSE | Eif4g2 | 102.106 | 906 | 8 | 4 | 4 | 28599000 | 12458000 | 1.6E+07 | 510690 | 222460 | 288230 | -0.37365748 |
| Q99KE1 | MAOM_MOUSE | Me2 | 65.799 | 589 | 9 | 4 | 5 | 87060000 | 37864000 | 4.9E+07 | 2638200 | 1147400 | 1490800 | -0.37771419 |
| Q9D964 | GATM_MOUSE | Gatm | 48.297 | 423 | 16 | 8 | 8 | 143010000 | 62175000 | 8.1E+07 | 4767000 | 2072500 | 2694500 | -0.37862764 |
| Q9EQH3 | VPS35_MOUSE | Vps35 | 91.713 | 796 | 22 | 11 | 11 | 150910000 | 65526000 | 8.5E+07 | 3143900 | 1365100 | 1778800 | -0.3818307 |
| P83882 | RL36A_MOUSE | Rpl36a | 12.441 | 106 | 2 | 1 | 1 | 31088000 | 13492000 | 1.8E+07 | 5181300 | 2248600 | 2932600 | -0.38314328 |
| Q8CGY8 | OGT1_MOUSE | Ogt | 116.952 | 1046 | 21 | 8 | 13 | 92763000 | 40211000 | 5.3E+07 | 1750200 | 758700 | 991540 | -0.38615546 |
| Q8JZQ2 | AFG32_MOUSE | Afg3l2 | 89.519 | 802 | 6 | 2 | 4 | 14194000 | 6152300 | 8041800 | 267810 | 116080 | 151730 | -0.3863926 |
| Q9D0M1 | KPRA_MOUSE | Prpsap1 | 39.432 | 356 | 3 | 1 | 2 | 4247400 | 1838100 | 2409300 | 176980 | 76589 | 100390 | -0.39039879 |
| Q8BQM4 | HEAT3_MOUSE | Heatr3 | 74.305 | 679 | 4 | 2 | 2 | 11185000 | 4839000 | 6346400 | 319580 | 138260 | 181330 | -0.39122951 |
| Q9CZU3 | MTREX_MOUSE | Mtrex | 117.636 | 1040 | 23 | 10 | 13 | 92715000 | 40083000 | 5.3E+07 | 1495400 | 646500 | 848900 | -0.39294973 |
| O35609 | SCAM3_MOUSE | Scamp3 | 38.458 | 349 | 7 | 3 | 4 | 51698000 | 22340000 | 2.9E+07 | 3231100 | 1396200 | 1834900 | -0.39417364 |
| Q9DB77 | QCR2_MOUSE | Uqcrc2 | 48.235 | 453 | 20 | 10 | 10 | 342780000 | 148020000 | 1.9E+08 | 15581000 | 6728200 | 8852900 | -0.39590528 |
| Q9ET30 | TM9S3_MOUSE | Tm9sf3 | 67.545 | 587 | 6 | 3 | 3 | 22429000 | 9677600 | 1.3E+07 | 1019500 | 439890 | 579630 | -0.39800232 |
| P63028 | TCTP_MOUSE | Tpt1 | 19.462 | 172 | 2 | 1 | 1 | 14865000 | 6398100 | 8467200 | 2477500 | 1066300 | 1411200 | -0.40424143 |
| Q8BVE3 | VATH_MOUSE | Atp6v1h | 55.855 | 483 | 8 | 3 | 5 | 36232000 | 15590000 | 2.1E+07 | 1341900 | 577420 | 764500 | -0.40496183 |
| P51174 | ACADL_MOUSE | Acadl | 47.908 | 430 | 19 | 10 | 9 | 167070000 | 71853000 | 9.5E+07 | 6961400 | 2993900 | 3967500 | -0.40621624 |
| Q9DBG3 | AP2B1_MOUSE | Ap2b1 | 104.583 | 937 | 35 | 16 | 19 | 363340000 | 156220000 | 2.1E+08 | 7569500 | 3254600 | 4314900 | -0.4068877 |
| Q8JZM0 | TFB1M_MOUSE | Tfb1m | 38.962 | 345 | 2 | 1 | 1 | 9248200 | 3975600 | 5272600 | 402100 | 172850 | 229240 | -0.40734194 |
| Q80VD1 | FA98B_MOUSE | Fam98b | 45.349 | 429 | 12 | 6 | 6 | 103410000 | 44304000 | 5.9E+07 | 3565700 | 1527700 | 2038000 | -0.41576999 |
| P43277 | H13_MOUSE | H1-3 | 22.1 | 221 | 10 | 5 | 5 | 691290000 | 295670000 | 4E+08 | 57607000 | 24639000 | 3.3E+07 | -0.42012749 |
| P67984 | RL22_MOUSE | Rpl22 | 14.759 | 128 | 6 | 3 | 3 | 600690000 | 256650000 | 3.4E+08 | 100110000 | 42775000 | 5.7E+07 | -0.42277405 |
| O88543 | CSN3_MOUSE | Cops3 | 47.832 | 423 | 2 | 1 | 1 | 12331000 | 5262000 | 7068700 | 456690 | 194890 | 261800 | -0.42583367 |
| Q8BG51 | MIRO1_MOUSE | Rhot1 | 72.242 | 631 | 2 | 1 | 1 | 10360000 | 4419500 | 5940700 | 345340 | 147320 | 198020 | -0.42674978 |
| Q0VGB7 | PP4R2_MOUSE | Ppp4r2 | 46.479 | 417 | 2 | 1 | 1 | 7438700 | 3172900 | 4265800 | 297550 | 126920 | 170630 | -0.42701427 |
| Q9DCL9 | PUR6_MOUSE | Paics | 47.006 | 425 | 45 | 22 | 23 | 2450500000 | 1044700000 | 1.4E+09 | 90760000 | 38693000 | 5.2E+07 | -0.42830265 |
| O70435 | PSA3_MOUSE | Psma3 | 28.405 | 255 | 14 | 7 | 7 | 130530000 | 55540000 | 7.5E+07 | 8702000 | 3702700 | 4999300 | -0.43317105 |
| Q05144 | RAC2_MOUSE | Rac2 | 21.441 | 192 | 11 | 5 | 6 | 267060000 | 113500000 | 1.5E+08 | 26706000 | 11350000 | 1.5E+07 | -0.43611017 |
| Q80UW8 | RPAB1_MOUSE | Polr2e | 24.57 | 210 | 5 | 2 | 3 | 36539000 | 15494000 | 2.1E+07 | 2810700 | 1191800 | 1618900 | -0.44176786 |
| Q9D0B6 | PBDC1_MOUSE | Pbdc1 | 22.223 | 198 | 14 | 7 | 7 | 348840000 | 147880000 | 2E+08 | 26834000 | 11376000 | 1.5E+07 | -0.44248142 |
| Q8C0C7 | SYFA_MOUSE | Farsa | 57.599 | 508 | 6 | 3 | 3 | 24086000 | 10209000 | 1.4E+07 | 802850 | 340300 | 462550 | -0.44285416 |
| Q9DBT5 | AMPD2_MOUSE | Ampd2 | 92.024 | 798 | 6 | 2 | 4 | 26545000 | 11244000 | 1.5E+07 | 603300 | 255550 | 347750 | -0.44447058 |
| Q9EQP2 | EHD4_MOUSE | Ehd4 | 61.481 | 541 | 25 | 13 | 12 | 227370000 | 96242000 | 1.3E+08 | 7105500 | 3007600 | 4097900 | -0.44625926 |
| P00493 | HPRT_MOUSE | Hprt1 | 24.57 | 218 | 22 | 11 | 11 | 467450000 | 197400000 | 2.7E+08 | 35958000 | 15184000 | 2.1E+07 | -0.45210456 |
| Q8BH73 | QPCTL_MOUSE | Qpctl | 42.692 | 383 | 6 | 3 | 3 | 20112000 | 8488200 | 1.2E+07 | 1547100 | 652940 | 894130 | -0.45357605 |
| Q9DC61 | MPPA_MOUSE | Pmpca | 58.279 | 524 | 26 | 11 | 15 | 219540000 | 92641000 | 1.3E+08 | 6272500 | 2646900 | 3625700 | -0.45396934 |
| P42125 | ECI1_MOUSE | Eci1 | 32.25 | 289 | 14 | 7 | 7 | 111050000 | 46854000 | 6.4E+07 | 6169600 | 2603000 | 3566600 | -0.45437861 |
| P63017 | HSP7C_MOUSE | Hspa8 | 70.871 | 646 | 81 | 39 | 42 | 1.1336E+10 | 4781500000 | 6.6E+09 | 306370000 | 129230000 | 1.8E+08 | -0.45493441 |
| P39054 | DYN2_MOUSE | Dnm2 | 98.145 | 870 | 14 | 6 | 8 | 42676000 | 17999000 | 2.5E+07 | 723320 | 305070 | 418250 | -0.45525026 |
| P16045 | LEG1_MOUSE | Lgals1 | 14.866 | 135 | 4 | 2 | 2 | 28977000 | 12205000 | 1.7E+07 | 2897700 | 1220500 | 1677100 | -0.45849642 |
| P16254 | SRP14_MOUSE | Srp14 | 12.51 | 110 | 2 | 1 | 1 | 5026600 | 2116700 | 2909900 | 837770 | 352780 | 484990 | -0.45915276 |
| Q91WN1 | DNJC9_MOUSE | Dnajc9 | 30.059 | 259 | 8 | 3 | 5 | 51274000 | 21575000 | 3E+07 | 2848500 | 1198600 | 1649900 | -0.46100522 |
| P62852 | RS25_MOUSE | Rps25 | 13.742 | 125 | 10 | 5 | 5 | 2341000000 | 983680000 | 1.4E+09 | 390170000 | 163950000 | 2.3E+08 | -0.46458494 |
| Q9EP72 | EMC7_MOUSE | Emc7 | 26.31 | 241 | 4 | 2 | 2 | 46769000 | 19646000 | 2.7E+07 | 3597600 | 1511200 | 2086400 | -0.46528116 |
| Q922R8 | PDIA6_MOUSE | Pdia6 | 48.1 | 440 | 23 | 10 | 13 | 1361500000 | 571320000 | 7.9E+08 | 54461000 | 22853000 | 3.2E+07 | -0.46791881 |
| Q8R574 | KPRB_MOUSE | Prpsap2 | 40.881 | 369 | 4 | 2 | 2 | 7255000 | 3043900 | 4211200 | 279040 | 117070 | 161970 | -0.46831043 |
| P22892 | AP1G1_MOUSE | Ap1g1 | 91.35 | 822 | 6 | 3 | 3 | 14793000 | 6205200 | 8587300 | 328720 | 137890 | 190830 | -0.46872688 |
| Q9CY58 | PAIRB_MOUSE | Serbp1 | 44.714 | 407 | 25 | 11 | 14 | 462010000 | 193740000 | 2.7E+08 | 22000000 | 9225700 | 1.3E+07 | -0.46956388 |
| P70406 | UCP2_MOUSE | Ucp2 | 33.374 | 309 | 3 | 1 | 2 | 9297000 | 3898600 | 5398500 | 516500 | 216590 | 299910 | -0.46960246 |
| P05064 | ALDOA_MOUSE | Aldoa | 39.356 | 364 | 53 | 25 | 28 | 4012900000 | 1674500000 | 2.3E+09 | 167200000 | 69769000 | 9.7E+07 | -0.48179136 |
| Q9CQN1 | TRAP1_MOUSE | Trap1 | 80.209 | 706 | 8 | 3 | 5 | 49606000 | 20690000 | 2.9E+07 | 953960 | 397880 | 556080 | -0.48293435 |
| Q9CQ65 | MTAP_MOUSE | Mtap | 31.062 | 283 | 30 | 15 | 15 | 708990000 | 294490000 | 4.1E+08 | 37315000 | 15500000 | 2.2E+07 | -0.49315346 |
| Q921G8 | GCP2_MOUSE | Tubgcp2 | 103.223 | 905 | 9 | 4 | 5 | 27439000 | 11371000 | 1.6E+07 | 457320 | 189520 | 267800 | -0.49883123 |
| O88456 | CPNS1_MOUSE | Capns1 | 28.406 | 268 | 1 | 0 | 1 | 2456200 | 0 | 2456200 | 188940 | 0 | 188940 | -0.50317662 |
| Q8BYL4 | SYYM_MOUSE | Yars2 | 52.597 | 472 | 5 | 2 | 3 | 20247000 | 8366400 | 1.2E+07 | 723110 | 298800 | 424310 | -0.50597739 |
| Q8CI71 | VPS50_MOUSE | Vps50 | 111.174 | 964 | 4 | 2 | 2 | 6949600 | 2870400 | 4079200 | 119820 | 49490 | 70330 | -0.50703445 |
| Q8BRF7 | SCFD1_MOUSE | Scfd1 | 72.323 | 639 | 5 | 2 | 3 | 16688000 | 6888400 | 9800000 | 439170 | 181270 | 257900 | -0.50861283 |
| P40124 | CAP1_MOUSE | Cap1 | 51.565 | 474 | 39 | 19 | 20 | 1087600000 | 448660000 | 6.4E+08 | 33989000 | 14020000 | 2E+07 | -0.51014821 |
| Q9D6K5 | SYJ2B_MOUSE | Synj2bp | 15.815 | 145 | 3 | 1 | 2 | 20088000 | 8272200 | 1.2E+07 | 2232000 | 919130 | 1312900 | -0.51439876 |
| O35129 | PHB2_MOUSE | Phb2 | 33.296 | 299 | 28 | 13 | 15 | 836060000 | 344220000 | 4.9E+08 | 32156000 | 13239000 | 1.9E+07 | -0.51485815 |
| Q9D3D9 | ATPD_MOUSE | Atp5f1d | 17.6 | 168 | 6 | 3 | 3 | 232190000 | 95509000 | 1.4E+08 | 33170000 | 13644000 | 2E+07 | -0.51709356 |
| P46471 | PRS7_MOUSE | Psmc2 | 48.648 | 433 | 24 | 11 | 13 | 209030000 | 85922000 | 1.2E+08 | 6532200 | 2685100 | 3847100 | -0.51884847 |
| Q7TMB8 | CYFP1_MOUSE | Cyfip1 | 145.241 | 1253 | 19 | 9 | 10 | 63037000 | 25904000 | 3.7E+07 | 863520 | 354850 | 508680 | -0.51952699 |
| Q9D0I8 | MRT4_MOUSE | Mrto4 | 27.546 | 239 | 17 | 8 | 9 | 182940000 | 75067000 | 1.1E+08 | 10761000 | 4415700 | 6345700 | -0.52317669 |
| Q8QZY1 | EIF3L_MOUSE | Eif3l | 66.613 | 564 | 41 | 19 | 22 | 608110000 | 249450000 | 3.6E+08 | 19003000 | 7795300 | 1.1E+07 | -0.52386619 |
| Q8CGP2 | H2B1P_MOUSE | Hist1h2bp | 13.992 | 126 | 11 | 5 | 6 | 564690000 | 231370000 | 3.3E+08 | 51335000 | 21033000 | 3E+07 | -0.52670607 |
| P05202 | AATM_MOUSE | Got2 | 47.411 | 430 | 48 | 24 | 24 | 1686600000 | 689150000 | 1E+09 | 54407000 | 22231000 | 3.2E+07 | -0.53345542 |
| Q99K74 | MED24_MOUSE | Med24 | 109.985 | 987 | 2 | 1 | 1 | 5294600 | 2154900 | 3139700 | 96266 | 39180 | 57086 | -0.54300579 |
| Q9CZW5 | TOM70_MOUSE | Tomm70 | 67.59 | 611 | 21 | 10 | 11 | 326750000 | 132840000 | 1.9E+08 | 9610300 | 3907100 | 5703300 | -0.54569758 |
| Q99J09 | MEP50_MOUSE | Wdr77 | 36.943 | 342 | 8 | 3 | 5 | 99661000 | 40442000 | 5.9E+07 | 7666200 | 3110900 | 4555300 | -0.55020578 |
| Q9CQC6 | 5MP2_MOUSE | Bzw1 | 48.043 | 419 | 2 | 1 | 1 | 6606200 | 2677500 | 3928600 | 220210 | 89251 | 130950 | -0.55312871 |
| Q61316 | HSP74_MOUSE | Hspa4 | 94.133 | 841 | 52 | 24 | 28 | 791160000 | 320340000 | 4.7E+08 | 13880000 | 5620000 | 8260000 | -0.55557165 |
| P63038 | CH60_MOUSE | Hspd1 | 60.955 | 573 | 77 | 38 | 39 | 3772900000 | 1526400000 | 2.2E+09 | 104800000 | 42400000 | 6.2E+07 | -0.55754599 |
| P14206 | RSSA_MOUSE | Rpsa | 32.838 | 295 | 27 | 14 | 13 | 1390800000 | 562260000 | 8.3E+08 | 92722000 | 37484000 | 5.5E+07 | -0.55938617 |
| Q9JIF0 | ANM1_MOUSE | Prmt1 | 42.436 | 371 | 6 | 2 | 4 | 25395000 | 10256000 | 1.5E+07 | 1209300 | 488380 | 720920 | -0.56180174 |
| P35601 | RFC1_MOUSE | Rfc1 | 125.985 | 1131 | 4 | 2 | 2 | 50126000 | 20199000 | 3E+07 | 737150 | 297050 | 440100 | -0.56716379 |
| P05977 | MYL1_MOUSE | Myl1 | 20.595 | 188 | 6 | 1 | 5 | 178950000 | 72085000 | 1.1E+08 | 14912000 | 6007100 | 8905300 | -0.56795093 |
| P85094 | ISC2A_MOUSE | Isoc2a | 22.417 | 206 | 3 | 1 | 2 | 7103300 | 2860500 | 4242800 | 710330 | 286050 | 424280 | -0.56874933 |
| P50431 | GLYC_MOUSE | Shmt1 | 52.601 | 478 | 10 | 5 | 5 | 65803000 | 26486000 | 3.9E+07 | 2437100 | 980950 | 1456200 | -0.56992326 |
| P54116 | STOM_MOUSE | Stom | 31.375 | 284 | 10 | 4 | 6 | 98756000 | 39735000 | 5.9E+07 | 5809200 | 2337300 | 3471900 | -0.57084246 |
| Q8BKZ9 | ODPX_MOUSE | Pdhx | 53.999 | 501 | 9 | 4 | 5 | 45108000 | 18138000 | 2.7E+07 | 1611000 | 647780 | 963220 | -0.57234014 |
| Q91WK2 | EIF3H_MOUSE | Eif3h | 39.832 | 352 | 19 | 9 | 10 | 244410000 | 97632000 | 1.5E+08 | 10184000 | 4068000 | 6115800 | -0.58822941 |
| Q7TNV0 | DEK_MOUSE | Dek | 43.159 | 380 | 6 | 3 | 3 | 36926000 | 14730000 | 2.2E+07 | 1605500 | 640440 | 965040 | -0.59154228 |
| Q922Q4 | P5CR2_MOUSE | Pycr2 | 33.659 | 320 | 15 | 7 | 8 | 149170000 | 59377000 | 9E+07 | 8287400 | 3298700 | 4988700 | -0.59676304 |
| Q8BH95 | ECHM_MOUSE | Echs1 | 31.474 | 290 | 16 | 8 | 8 | 211030000 | 83981000 | 1.3E+08 | 11724000 | 4665600 | 7058200 | -0.5972615 |
| P80318 | TCPG_MOUSE | Cct3 | 60.63 | 545 | 60 | 28 | 32 | 1641500000 | 652910000 | 9.9E+08 | 43196000 | 17182000 | 2.6E+07 | -0.5984298 |
| P62196 | PRS8_MOUSE | Psmc5 | 45.626 | 406 | 28 | 12 | 16 | 169200000 | 67184000 | 1E+08 | 6043000 | 2399400 | 3643600 | -0.60266241 |
| Q8VCN5 | CGL_MOUSE | Cth | 43.567 | 398 | 2 | 1 | 1 | 6922700 | 2746900 | 4175800 | 300990 | 119430 | 181560 | -0.60424823 |
| Q9CZ13 | QCR1_MOUSE | Uqcrc1 | 52.852 | 480 | 26 | 13 | 13 | 283110000 | 112310000 | 1.7E+08 | 12869000 | 5105200 | 7763400 | -0.60473712 |
| O89053 | COR1A_MOUSE | Coro1a | 50.989 | 461 | 22 | 10 | 12 | 464750000 | 184330000 | 2.8E+08 | 19365000 | 7680400 | 1.2E+07 | -0.6053498 |
| Q9R0A0 | PEX14_MOUSE | Pex14 | 41.208 | 376 | 4 | 2 | 2 | 16715000 | 6625100 | 1E+07 | 983250 | 389710 | 593540 | -0.60691204 |
| Q9CR00 | PSMD9_MOUSE | Psmd9 | 24.72 | 222 | 4 | 2 | 2 | 23134000 | 9165700 | 1.4E+07 | 1652400 | 654690 | 997740 | -0.60780849 |
| Q9R1P0 | PSA4_MOUSE | Psma4 | 29.471 | 261 | 6 | 2 | 4 | 98817000 | 39142000 | 6E+07 | 7058400 | 2795900 | 4262500 | -0.60840919 |
| P31230 | AIMP1_MOUSE | Aimp1 | 33.997 | 310 | 2 | 1 | 1 | 14074000 | 5564100 | 8509800 | 740730 | 292850 | 447880 | -0.61297688 |
| O70251 | EF1B_MOUSE | Eef1b | 24.694 | 225 | 17 | 8 | 9 | 1628400000 | 642600000 | 9.9E+08 | 108560000 | 42840000 | 6.6E+07 | -0.61731546 |
| Q9R1P1 | PSB3_MOUSE | Psmb3 | 22.965 | 205 | 10 | 3 | 7 | 152440000 | 60094000 | 9.2E+07 | 16938000 | 6677200 | 1E+07 | -0.61979727 |
| Q8CII2 | CD123_MOUSE | Cdc123 | 38.816 | 336 | 5 | 2 | 3 | 11605000 | 4570600 | 7034800 | 644740 | 253920 | 390820 | -0.62212584 |
| Q6P6M7 | SPCS_MOUSE | Sepsecs | 55.326 | 504 | 2 | 1 | 1 | 35993000 | 14173000 | 2.2E+07 | 1124800 | 442900 | 681890 | -0.62257205 |
| Q8CGP6 | H2A1H_MOUSE | H2ac12 | 13.95 | 128 | 4 | 2 | 2 | 476690000 | 187470000 | 2.9E+08 | 68099000 | 26781000 | 4.1E+07 | -0.62550757 |
| Q99KJ8 | DCTN2_MOUSE | Dctn2 | 44.117 | 402 | 14 | 6 | 8 | 109850000 | 43188000 | 6.7E+07 | 4225100 | 1661100 | 2564100 | -0.62632066 |
| Q9CR68 | UCRI_MOUSE | Uqcrfs1 | 29.368 | 274 | 8 | 4 | 4 | 61216000 | 24017000 | 3.7E+07 | 3826000 | 1501100 | 2324900 | -0.63120789 |
| Q9CZ91 | SRFB1_MOUSE | Srfbp1 | 48.746 | 441 | 3 | 1 | 2 | 37381000 | 14664000 | 2.3E+07 | 1437700 | 564000 | 873720 | -0.63149363 |
| Q9CR51 | VATG1_MOUSE | Atp6v1g1 | 13.724 | 118 | 4 | 2 | 2 | 29794000 | 11649000 | 1.8E+07 | 4965700 | 1941400 | 3024300 | -0.63944545 |
| Q2YDW2 | MSTO1_MOUSE | Msto1 | 61.23 | 556 | 5 | 2 | 3 | 13262000 | 5181100 | 8080900 | 378910 | 148030 | 230880 | -0.64125755 |
| Q9CPW3 | RM54_MOUSE | Mrpl54 | 15.422 | 135 | 2 | 1 | 1 | 6295900 | 2458700 | 3837200 | 786990 | 307330 | 479660 | -0.64215825 |
| Q9D1I6 | RM14_MOUSE | Mrpl14 | 15.874 | 145 | 2 | 1 | 1 | 18246000 | 7121600 | 1.1E+07 | 1824600 | 712160 | 1112400 | -0.64340234 |
| P06151 | LDHA_MOUSE | Ldha | 36.499 | 332 | 46 | 24 | 22 | 5370900000 | 2095100000 | 3.3E+09 | 233520000 | 91091000 | 1.4E+08 | -0.64482817 |
| Q07417 | ACADS_MOUSE | Acads | 44.89 | 412 | 4 | 1 | 3 | 11199000 | 4363500 | 6836000 | 533310 | 207780 | 325520 | -0.6476666 |
| P11157 | RIR2_MOUSE | Rrm2 | 45.096 | 390 | 15 | 7 | 8 | 122040000 | 47391000 | 7.5E+07 | 4693800 | 1822700 | 2871100 | -0.65550983 |
| P70349 | HINT1_MOUSE | Hint1 | 13.777 | 126 | 6 | 3 | 3 | 70463000 | 27330000 | 4.3E+07 | 8807800 | 3416200 | 5391600 | -0.6583066 |
| P01942 | HBA_MOUSE | Hba | 15.085 | 142 | 7 | 3 | 4 | 66057000 | 25616000 | 4E+07 | 8257200 | 3202000 | 5055200 | -0.65877346 |
| Q924T2 | RT02_MOUSE | Mrps2 | 32.313 | 291 | 4 | 2 | 2 | 20591000 | 7963300 | 1.3E+07 | 1211300 | 468430 | 742830 | -0.66518785 |
| P80315 | TCPD_MOUSE | Cct4 | 58.066 | 539 | 38 | 18 | 20 | 1323100000 | 511590000 | 8.1E+08 | 34818000 | 13463000 | 2.1E+07 | -0.66558525 |
| Q8CDN6 | TXNL1_MOUSE | Txnl1 | 32.237 | 289 | 2 | 1 | 1 | 6189400 | 2392300 | 3797100 | 412620 | 159490 | 253140 | -0.66649968 |
| P62814 | VATB2_MOUSE | Atp6v1b2 | 56.551 | 511 | 27 | 13 | 14 | 193290000 | 74369000 | 1.2E+08 | 6665100 | 2564400 | 4100700 | -0.67721809 |
| Q9D051 | ODPB_MOUSE | Pdhb | 38.937 | 359 | 21 | 10 | 11 | 229420000 | 88105000 | 1.4E+08 | 10925000 | 4195500 | 6729500 | -0.68166985 |
| Q8CEE7 | RDH13_MOUSE | Rdh13 | 36.464 | 334 | 7 | 3 | 4 | 32327000 | 12398000 | 2E+07 | 1469400 | 563570 | 905850 | -0.68476191 |
| P58281 | OPA1_MOUSE | Opa1 | 111.339 | 960 | 14 | 6 | 8 | 45321000 | 17364000 | 2.8E+07 | 697250 | 267150 | 430100 | -0.68711023 |
| P61164 | ACTZ_MOUSE | Actr1a | 42.614 | 376 | 12 | 5 | 7 | 85537000 | 32757000 | 5.3E+07 | 3564100 | 1364900 | 2199200 | -0.68821545 |
| Q9ES74 | NEK7_MOUSE | Nek7 | 34.537 | 302 | 2 | 1 | 1 | 6166400 | 2360800 | 3805700 | 324550 | 124250 | 200300 | -0.68888601 |
| P67871 | CSK2B_MOUSE | Csnk2b | 24.942 | 215 | 12 | 6 | 6 | 150140000 | 57260000 | 9.3E+07 | 13649000 | 5205500 | 8443300 | -0.69777817 |
| Q64442 | DHSO_MOUSE | Sord | 38.249 | 357 | 4 | 2 | 2 | 9511800 | 3621500 | 5890300 | 432350 | 164610 | 267740 | -0.70175374 |
| P47738 | ALDH2_MOUSE | Aldh2 | 56.538 | 519 | 36 | 16 | 20 | 826340000 | 314250000 | 5.1E+08 | 30605000 | 11639000 | 1.9E+07 | -0.70445647 |
| Q9DBL1 | ACDSB_MOUSE | Acadsb | 47.874 | 432 | 4 | 1 | 3 | 14545000 | 5498800 | 9046700 | 559440 | 211490 | 347950 | -0.71827482 |
| Q9WVK4 | EHD1_MOUSE | Ehd1 | 60.603 | 534 | 50 | 23 | 27 | 863310000 | 326060000 | 5.4E+08 | 26161000 | 9880700 | 1.6E+07 | -0.72045611 |
| P42932 | TCPQ_MOUSE | Cct8 | 59.555 | 548 | 65 | 33 | 32 | 2585600000 | 974750000 | 1.6E+09 | 64640000 | 24369000 | 4E+07 | -0.72476278 |
| Q8VDM4 | PSMD2_MOUSE | Psmd2 | 100.203 | 908 | 47 | 22 | 25 | 513470000 | 193480000 | 3.2E+08 | 9008200 | 3394300 | 5613900 | -0.72584238 |
| Q14C51 | PTCD3_MOUSE | Ptcd3 | 77.796 | 685 | 17 | 8 | 9 | 92061000 | 34678000 | 5.7E+07 | 2001300 | 753870 | 1247500 | -0.7266027 |
| P49722 | PSA2_MOUSE | Psma2 | 25.927 | 234 | 16 | 7 | 9 | 262420000 | 98403000 | 1.6E+08 | 16401000 | 6150200 | 1E+07 | -0.73700958 |
| Q91V64 | ISOC1_MOUSE | Isoc1 | 32.033 | 297 | 10 | 5 | 5 | 81681000 | 30623000 | 5.1E+07 | 5105100 | 1913900 | 3191100 | -0.7375214 |
| Q9DBR1 | XRN2_MOUSE | Xrn2 | 108.687 | 951 | 2 | 1 | 1 | 9249000 | 3451200 | 5797800 | 162260 | 60547 | 101720 | -0.74840749 |
| Q9JI11 | STK4_MOUSE | Stk4 | 55.541 | 487 | 13 | 6 | 7 | 102740000 | 38315000 | 6.4E+07 | 3314200 | 1236000 | 2078300 | -0.74973372 |
| Q9CXW2 | RT22_MOUSE | Mrps22 | 41.192 | 359 | 8 | 2 | 6 | 35739000 | 13305000 | 2.2E+07 | 1374600 | 511740 | 862850 | -0.75371837 |
| Q9WVA2 | TIM8A_MOUSE | Timm8a1 | 11.042 | 97 | 2 | 1 | 1 | 44377000 | 16485000 | 2.8E+07 | 7396100 | 2747500 | 4648700 | -0.7586975 |
| P50516 | VATA_MOUSE | Atp6v1a | 68.326 | 617 | 35 | 16 | 19 | 308210000 | 114390000 | 1.9E+08 | 8110900 | 3010300 | 5100600 | -0.76075651 |
| P70315 | WASP_MOUSE | Was | 54.192 | 520 | 4 | 2 | 2 | 18476000 | 6855000 | 1.2E+07 | 879810 | 326430 | 553380 | -0.76150565 |
| Q8BKC5 | IPO5_MOUSE | Ipo5 | 123.591 | 1097 | 14 | 5 | 9 | 88589000 | 32665000 | 5.6E+07 | 1554200 | 573060 | 981130 | -0.77572191 |
| Q8C0M9 | ASGL1_MOUSE | Asrgl1 | 33.95 | 326 | 17 | 8 | 9 | 247470000 | 91168000 | 1.6E+08 | 15467000 | 5698000 | 9769200 | -0.77781065 |
| Q9WUP7 | UCHL5_MOUSE | Uchl5 | 37.617 | 329 | 12 | 5 | 7 | 67555000 | 24872000 | 4.3E+07 | 3377800 | 1243600 | 2134200 | -0.77913906 |
| P17182 | ENOA_MOUSE | Eno1 | 47.141 | 434 | 54 | 25 | 29 | 2712800000 | 998090000 | 1.7E+09 | 96884000 | 35646000 | 6.1E+07 | -0.78071437 |
| P57776 | EF1D_MOUSE | Eef1d | 31.293 | 281 | 31 | 15 | 16 | 1080600000 | 397360000 | 6.8E+08 | 51459000 | 18922000 | 3.3E+07 | -0.78203025 |
| P35700 | PRDX1_MOUSE | Prdx1 | 22.177 | 199 | 22 | 10 | 12 | 1376800000 | 504970000 | 8.7E+08 | 91785000 | 33665000 | 5.8E+07 | -0.78779952 |
| Q9CQE6 | ASF1A_MOUSE | Asf1a | 22.943 | 204 | 2 | 1 | 1 | 9557300 | 3498900 | 6058300 | 1194700 | 437370 | 757290 | -0.79201159 |
| Q61599 | GDIR2_MOUSE | Arhgdib | 22.851 | 200 | 6 | 3 | 3 | 61824000 | 22630000 | 3.9E+07 | 4416000 | 1616400 | 2799600 | -0.79239623 |
| Q9QYI3 | DNJC7_MOUSE | Dnajc7 | 56.476 | 494 | 6 | 2 | 4 | 33200000 | 12129000 | 2.1E+07 | 922220 | 336930 | 585290 | -0.79679817 |
| Q8R035 | ICT1_MOUSE | Mrpl58 | 23.477 | 206 | 9 | 3 | 6 | 39948000 | 14587000 | 2.5E+07 | 2663200 | 972490 | 1690700 | -0.79792843 |
| P47758 | SRPRB_MOUSE | Srprb | 29.579 | 269 | 22 | 10 | 12 | 238770000 | 86980000 | 1.5E+08 | 11939000 | 4349000 | 7589600 | -0.80332113 |
| Q9CYR0 | SSBP_MOUSE | Ssbp1 | 17.319 | 152 | 2 | 1 | 1 | 13113000 | 4769500 | 8343800 | 1192100 | 433590 | 758520 | -0.80686654 |
| Q9Z1X4 | ILF3_MOUSE | Ilf3 | 96.021 | 898 | 9 | 3 | 6 | 38995000 | 14183000 | 2.5E+07 | 928460 | 337690 | 590770 | -0.80687531 |
| Q9D868 | PPIH_MOUSE | Ppih | 20.464 | 188 | 9 | 4 | 5 | 97720000 | 35514000 | 6.2E+07 | 6980000 | 2536700 | 4443300 | -0.80866588 |
| Q9DCD0 | 6PGD_MOUSE | Pgd | 53.247 | 483 | 16 | 8 | 8 | 177020000 | 64323000 | 1.1E+08 | 5710400 | 2074900 | 3635400 | -0.80908092 |
| O08807 | PRDX4_MOUSE | Prdx4 | 31.053 | 274 | 6 | 3 | 3 | 289980000 | 105190000 | 1.8E+08 | 17058000 | 6187900 | 1.1E+07 | -0.81288913 |
| Q9DCH4 | EIF3F_MOUSE | Eif3f | 37.984 | 361 | 21 | 10 | 11 | 363050000 | 131500000 | 2.3E+08 | 22691000 | 8219000 | 1.4E+07 | -0.81626096 |
| Q8C263 | SKA3_MOUSE | Ska3 | 45.37 | 411 | 2 | 1 | 1 | 7025000 | 2541700 | 4483300 | 305440 | 110510 | 194930 | -0.81876728 |
| P17751 | TPIS_MOUSE | Tpi1 | 26.713 | 249 | 29 | 14 | 15 | 1485200000 | 537330000 | 9.5E+08 | 99015000 | 35822000 | 6.3E+07 | -0.81892648 |
| P55258 | RAB8A_MOUSE | Rab8a | 23.668 | 207 | 5 | 2 | 3 | 11352000 | 4092600 | 7259000 | 667740 | 240740 | 427000 | -0.82675315 |
| Q8K2Z4 | CND1_MOUSE | Ncapd2 | 155.665 | 1392 | 8 | 4 | 4 | 18285000 | 6586100 | 1.2E+07 | 222990 | 80319 | 142670 | -0.82888889 |
| P70460 | VASP_MOUSE | Vasp | 39.667 | 375 | 18 | 8 | 10 | 147330000 | 52807000 | 9.5E+07 | 8666400 | 3106300 | 5560100 | -0.83990571 |
| O54950 | AAKG1_MOUSE | Prkag1 | 37.52 | 330 | 11 | 5 | 6 | 121550000 | 43484000 | 7.8E+07 | 5525000 | 1976500 | 3548500 | -0.84422817 |
| Q9CZ04 | CSN7A_MOUSE | Cops7a | 30.224 | 275 | 3 | 1 | 2 | 6699200 | 2385500 | 4313700 | 418700 | 149090 | 269610 | -0.85463416 |
| P54071 | IDHP_MOUSE | Idh2 | 50.906 | 452 | 35 | 15 | 20 | 469470000 | 167130000 | 3E+08 | 14671000 | 5222900 | 9447900 | -0.85515342 |
| Q9DBJ1 | PGAM1_MOUSE | Pgam1 | 28.832 | 254 | 19 | 9 | 10 | 289170000 | 102850000 | 1.9E+08 | 19278000 | 6856900 | 1.2E+07 | -0.85724075 |
| P04184 | KITH_MOUSE | Tk1 | 25.776 | 233 | 5 | 2 | 3 | 41454000 | 14667000 | 2.7E+07 | 2763600 | 977780 | 1785800 | -0.8689592 |
| Q9CY57 | CHTOP_MOUSE | Chtop | 26.585 | 249 | 4 | 2 | 2 | 15437000 | 5439400 | 9998000 | 908080 | 319960 | 588120 | -0.87819201 |
| P06745 | G6PI_MOUSE | Gpi | 62.767 | 558 | 32 | 15 | 17 | 553020000 | 194410000 | 3.6E+08 | 16758000 | 5891100 | 1.1E+07 | -0.88331329 |
| Q61206 | PA1B2_MOUSE | Pafah1b2 | 25.581 | 229 | 4 | 2 | 2 | 44093000 | 15445000 | 2.9E+07 | 4899200 | 1716200 | 3183000 | -0.89124419 |
| Q9Z1Z2 | STRAP_MOUSE | Strap | 38.442 | 350 | 42 | 20 | 22 | 1374400000 | 480800000 | 8.9E+08 | 57265000 | 20033000 | 3.7E+07 | -0.89412771 |
| P80313 | TCPH_MOUSE | Cct7 | 59.652 | 544 | 49 | 24 | 25 | 1412300000 | 489810000 | 9.2E+08 | 42798000 | 14843000 | 2.8E+07 | -0.91335796 |
| Q99PT1 | GDIR1_MOUSE | Arhgdia | 23.407 | 204 | 16 | 8 | 8 | 276280000 | 95784000 | 1.8E+08 | 18419000 | 6385600 | 1.2E+07 | -0.91414225 |
| P45376 | ALDR_MOUSE | Akr1b1 | 35.732 | 316 | 2 | 1 | 1 | 5243900 | 1815800 | 3428100 | 249710 | 86468 | 163240 | -0.91680389 |
| Q9CPU0 | LGUL_MOUSE | Glo1 | 20.81 | 184 | 3 | 1 | 2 | 9191900 | 3165200 | 6026600 | 835620 | 287750 | 547870 | -0.92904765 |
| P53612 | PGTB2_MOUSE | Rabggtb | 37.803 | 339 | 7 | 4 | 3 | 31084000 | 10681000 | 2E+07 | 1726900 | 593410 | 1133500 | -0.93366386 |
| Q9D9Z5 | DDA1_MOUSE | Dda1 | 11.753 | 102 | 7 | 3 | 4 | 39266000 | 13462000 | 2.6E+07 | 4362900 | 1495800 | 2867100 | -0.93870196 |
| Q9R0P3 | ESTD_MOUSE | Esd | 31.32 | 282 | 14 | 7 | 7 | 175120000 | 59998000 | 1.2E+08 | 10945000 | 3749900 | 7195200 | -0.94015218 |
| Q8BIQ5 | CSTF2_MOUSE | Cstf2 | 61.341 | 580 | 3 | 1 | 2 | 7684500 | 2622800 | 5061700 | 307380 | 104910 | 202470 | -0.9485142 |
| P70168 | IMB1_MOUSE | Kpnb1 | 97.184 | 876 | 46 | 21 | 25 | 792230000 | 270300000 | 5.2E+08 | 18005000 | 6143200 | 1.2E+07 | -0.94929482 |
| Q9JLQ0 | CD2AP_MOUSE | Cd2ap | 70.45 | 637 | 13 | 6 | 7 | 69959000 | 23812000 | 4.6E+07 | 1706300 | 580790 | 1125500 | -0.95454806 |
| Q9CQJ6 | DENR_MOUSE | Denr | 22.166 | 198 | 5 | 2 | 3 | 26137000 | 8861300 | 1.7E+07 | 2904100 | 984590 | 1919500 | -0.96317895 |
| Q6PHZ2 | KCC2D_MOUSE | Camk2d | 56.369 | 499 | 4 | 2 | 2 | 24751000 | 8382100 | 1.6E+07 | 853480 | 289040 | 564440 | -0.96558255 |
| Q6ZQ38 | CAND1_MOUSE | Cand1 | 136.332 | 1230 | 42 | 19 | 23 | 365960000 | 123900000 | 2.4E+08 | 5462100 | 1849300 | 3612800 | -0.96618851 |
| O88396 | GRPE2_MOUSE | Grpel2 | 25.03 | 224 | 3 | 1 | 2 | 8988600 | 3019000 | 5969700 | 642050 | 215640 | 426400 | -0.98358768 |
| Q9D8V0 | HM13_MOUSE | Hm13 | 41.748 | 378 | 4 | 2 | 2 | 15785000 | 5292400 | 1E+07 | 928500 | 311320 | 617180 | -0.9872957 |
| Q6IRU5 | CLCB_MOUSE | Cltb | 25.172 | 229 | 7 | 3 | 4 | 61930000 | 20733000 | 4.1E+07 | 6193000 | 2073300 | 4119700 | -0.9906104 |
| O88844 | IDHC_MOUSE | Idh1 | 46.674 | 414 | 6 | 2 | 4 | 28143000 | 9420000 | 1.9E+07 | 907830 | 303870 | 603960 | -0.99101265 |
| Q8R395 | COMD5_MOUSE | Commd5 | 24.493 | 224 | 3 | 1 | 2 | 10078000 | 3371500 | 6706400 | 719850 | 240820 | 479030 | -0.99214794 |
| Q6P8X1 | SNX6_MOUSE | Snx6 | 46.649 | 406 | 4 | 2 | 2 | 11458000 | 3818700 | 7638900 | 409200 | 136380 | 272820 | -1.00028332 |
| P05201 | AATC_MOUSE | Got1 | 46.248 | 413 | 17 | 7 | 10 | 88469000 | 29356000 | 5.9E+07 | 3276600 | 1087300 | 2189400 | -1.00982004 |
| Q8QZT1 | THIL_MOUSE | Acat1 | 44.816 | 424 | 22 | 11 | 11 | 516440000 | 171070000 | 3.5E+08 | 21518000 | 7128000 | 1.4E+07 | -1.01351422 |
| P08249 | MDHM_MOUSE | Mdh2 | 35.611 | 338 | 31 | 15 | 16 | 871660000 | 288480000 | 5.8E+08 | 36319000 | 12020000 | 2.4E+07 | -1.01546994 |
| Q9JJT9 | PHAX_MOUSE | Phax | 43.247 | 385 | 3 | 1 | 2 | 10614000 | 3508800 | 7104800 | 482440 | 159490 | 322950 | -1.01781632 |
| P11352 | GPX1_MOUSE | Gpx1 | 22.329 | 201 | 5 | 2 | 3 | 16834000 | 5552500 | 1.1E+07 | 1202400 | 396610 | 805830 | -1.02281345 |
| Q64433 | CH10_MOUSE | Hspe1 | 10.963 | 102 | 11 | 5 | 6 | 515400000 | 169850000 | 3.5E+08 | 51540000 | 16985000 | 3.5E+07 | -1.02463326 |
| P63085 | MK01_MOUSE | Mapk1 | 41.276 | 358 | 2 | 1 | 1 | 10358000 | 3411200 | 6946800 | 431580 | 142130 | 289450 | -1.02606922 |
| Q9WV80 | SNX1_MOUSE | Snx1 | 58.952 | 522 | 11 | 5 | 6 | 38289000 | 12564000 | 2.6E+07 | 1320300 | 433230 | 887090 | -1.03393131 |
| P61202 | CSN2_MOUSE | Cops2 | 51.597 | 443 | 11 | 4 | 7 | 69580000 | 22677000 | 4.7E+07 | 2783200 | 907090 | 1876100 | -1.04845041 |
| O70591 | PFD2_MOUSE | Pfdn2 | 16.534 | 154 | 8 | 4 | 4 | 82262000 | 26797000 | 5.5E+07 | 6855200 | 2233100 | 4622100 | -1.04950618 |
| Q9CZU6 | CISY_MOUSE | Cs | 51.737 | 464 | 19 | 9 | 10 | 395960000 | 128970000 | 2.7E+08 | 18855000 | 6141500 | 1.3E+07 | -1.04975019 |
| Q8BMK4 | CKAP4_MOUSE | Ckap4 | 63.692 | 575 | 31 | 14 | 17 | 343730000 | 111770000 | 2.3E+08 | 8593300 | 2794400 | 5799000 | -1.05334303 |
| P31996 | CD68_MOUSE | Cd68 | 34.818 | 326 | 5 | 3 | 2 | 33793000 | 10967000 | 2.3E+07 | 3072100 | 997020 | 2075100 | -1.05750913 |
| Q9D706 | RPAP3_MOUSE | Rpap3 | 74.096 | 660 | 8 | 3 | 5 | 25486000 | 8268800 | 1.7E+07 | 606820 | 196880 | 409940 | -1.0580839 |
| O35344 | IMA4_MOUSE | Kpna3 | 57.773 | 521 | 16 | 7 | 9 | 223440000 | 72496000 | 1.5E+08 | 11172000 | 3624800 | 7547400 | -1.05809746 |
| Q9WTM5 | RUVB2_MOUSE | Ruvbl2 | 51.113 | 463 | 39 | 19 | 20 | 922250000 | 298840000 | 6.2E+08 | 31802000 | 10305000 | 2.1E+07 | -1.06080803 |
| Q99LD4 | CSN1_MOUSE | Gps1 | 53.442 | 471 | 5 | 2 | 3 | 27038000 | 8713200 | 1.8E+07 | 1039900 | 335120 | 704810 | -1.07253863 |
| P30285 | CDK4_MOUSE | Cdk4 | 33.751 | 303 | 9 | 4 | 5 | 67452000 | 21726000 | 4.6E+07 | 3372600 | 1086300 | 2286300 | -1.07359214 |
| Q9CXW3 | CYBP_MOUSE | Cacybp | 26.51 | 229 | 13 | 6 | 7 | 263010000 | 84237000 | 1.8E+08 | 16438000 | 5264800 | 1.1E+07 | -1.08557869 |
| P48758 | CBR1_MOUSE | Cbr1 | 30.641 | 277 | 9 | 4 | 5 | 63334000 | 20284000 | 4.3E+07 | 3333400 | 1067600 | 2265800 | -1.08567106 |
| Q6P069 | SORCN_MOUSE | Sri | 21.627 | 198 | 2 | 1 | 1 | 2257600 | 722690 | 1535000 | 173660 | 55591 | 118070 | -1.08678982 |
| P62827 | RAN_MOUSE | Ran | 24.423 | 216 | 2 | 1 | 1 | 21673000 | 6933700 | 1.5E+07 | 1806100 | 577810 | 1228300 | -1.08794132 |
| Q9CY73 | RM44_MOUSE | Mrpl44 | 37.527 | 333 | 5 | 2 | 3 | 16462000 | 5262000 | 1.1E+07 | 783890 | 250570 | 533320 | -1.08981558 |
| P46061 | RAGP1_MOUSE | Rangap1 | 63.531 | 589 | 17 | 8 | 9 | 93336000 | 29772000 | 6.4E+07 | 2828400 | 902190 | 1926200 | -1.09425377 |
| P60122 | RUVB1_MOUSE | Ruvbl1 | 50.214 | 456 | 35 | 17 | 18 | 893410000 | 283400000 | 6.1E+08 | 37225000 | 11808000 | 2.5E+07 | -1.10599313 |
| Q3TC46 | PATL1_MOUSE | Patl1 | 86.77 | 770 | 1 | 0 | 1 | 2380600 | 0 | 2380600 | 54104 | 0 | 54104 | -1.11318841 |
| P97807 | FUMH_MOUSE | Fh | 54.357 | 507 | 26 | 12 | 14 | 619350000 | 194560000 | 4.2E+08 | 19355000 | 6079900 | 1.3E+07 | -1.12656863 |
| P54822 | PUR8_MOUSE | Adsl | 54.866 | 484 | 15 | 7 | 8 | 102370000 | 31974000 | 7E+07 | 3302400 | 1031400 | 2271000 | -1.13869668 |
| Q8BJ71 | NUP93_MOUSE | Nup93 | 93.281 | 819 | 12 | 5 | 7 | 46752000 | 14595000 | 3.2E+07 | 834860 | 260620 | 574240 | -1.13970347 |
| P08071 | TRFL_MOUSE | Ltf | 77.838 | 707 | 1 | 0 | 1 | 2953300 | 0 | 2953300 | 59067 | 0 | 59067 | -1.14042803 |
| P12787 | COX5A_MOUSE | Cox5a | 16.101 | 146 | 5 | 2 | 3 | 89090000 | 27657000 | 6.1E+07 | 7424200 | 2304800 | 5119400 | -1.15136916 |
| Q3TIR3 | RIC8A_MOUSE | Ric8a | 59.847 | 530 | 7 | 3 | 4 | 291650000 | 89800000 | 2E+08 | 9114200 | 2806200 | 6307900 | -1.16849624 |
| Q03147 | CDK7_MOUSE | Cdk7 | 38.968 | 346 | 4 | 2 | 2 | 14996000 | 4606100 | 1E+07 | 749800 | 230300 | 519500 | -1.17357802 |
| Q9D1B9 | RM28_MOUSE | Mrpl28 | 30.17 | 257 | 5 | 2 | 3 | 29953000 | 9151400 | 2.1E+07 | 1497700 | 457570 | 1040100 | -1.18465787 |
| Q9CQT2 | RBM7_MOUSE | Rbm7 | 30.148 | 265 | 5 | 2 | 3 | 20635000 | 6299000 | 1.4E+07 | 1473900 | 449930 | 1024000 | -1.18644783 |
| Q8CG71 | P3H2_MOUSE | P3h2 | 80.154 | 703 | 2 | 1 | 1 | 213580000 | 65100000 | 1.5E+08 | 4854000 | 1479500 | 3374500 | -1.18953917 |
| D3Z7P3 | GLSK_MOUSE | Gls | 73.964 | 674 | 11 | 5 | 6 | 84321000 | 25667000 | 5.9E+07 | 2007600 | 611120 | 1396500 | -1.19231482 |
| O54825 | BYST_MOUSE | Bysl | 49.784 | 436 | 9 | 4 | 5 | 73213000 | 22255000 | 5.1E+07 | 2614700 | 794810 | 1819900 | -1.19517916 |
| Q99MD9 | NASP_MOUSE | Nasp | 83.954 | 773 | 5 | 2 | 3 | 16824000 | 5104900 | 1.2E+07 | 391260 | 118720 | 272540 | -1.19889486 |
| Q9JIG7 | CCD22_MOUSE | Ccdc22 | 70.844 | 627 | 7 | 3 | 4 | 20901000 | 6306200 | 1.5E+07 | 497640 | 150150 | 347490 | -1.21063138 |
| Q9JLJ2 | AL9A1_MOUSE | Aldh9a1 | 53.515 | 494 | 10 | 4 | 6 | 62539000 | 18863000 | 4.4E+07 | 2156500 | 650440 | 1506100 | -1.21128159 |
| P61290 | PSME3_MOUSE | Psme3 | 29.506 | 254 | 12 | 6 | 6 | 62916000 | 18884000 | 4.4E+07 | 3932300 | 1180300 | 2752000 | -1.22138799 |
| P28063 | PSB8_MOUSE | Psmb8 | 30.26 | 276 | 7 | 3 | 4 | 107110000 | 32041000 | 7.5E+07 | 5355600 | 1602100 | 3753600 | -1.22835574 |
| Q9CWJ9 | PUR9_MOUSE | Atic | 64.217 | 592 | 11 | 5 | 6 | 39875000 | 11915000 | 2.8E+07 | 1107600 | 330970 | 776670 | -1.23058541 |
| Q9D819 | IPYR_MOUSE | Ppa1 | 32.667 | 289 | 9 | 4 | 5 | 99891000 | 29824000 | 7E+07 | 4994600 | 1491200 | 3503400 | -1.23226136 |
| P46664 | PURA2_MOUSE | Adss2 | 50.021 | 456 | 1 | 0 | 1 | 1558900 | 0 | 1558900 | 59959 | 0 | 59959 | -1.23464952 |
| Q62083 | PICK1_MOUSE | Pick1 | 46.597 | 416 | 5 | 2 | 3 | 21868000 | 6490200 | 1.5E+07 | 874720 | 259610 | 615110 | -1.24453304 |
| Q8BMJ3 | IF1AX_MOUSE | Eif1ax | 16.46 | 144 | 3 | 1 | 2 | 9496900 | 2812200 | 6684700 | 949690 | 281220 | 668470 | -1.24916361 |
| Q9CQE2 | TM223_MOUSE | Tmem223 | 21.857 | 199 | 1 | 0 | 1 | 2499600 | 0 | 2499600 | 208300 | 0 | 208300 | -1.25669732 |
| Q99J87 | DHX58_MOUSE | Dhx58 | 76.709 | 678 | 4 | 2 | 2 | 8973800 | 2640900 | 6332900 | 190930 | 56189 | 134740 | -1.26183663 |
| Q9JHI5 | IVD_MOUSE | Ivd | 46.325 | 424 | 3 | 1 | 2 | 9054100 | 2648100 | 6406000 | 377250 | 110340 | 266920 | -1.27446619 |
| Q99LC5 | ETFA_MOUSE | Etfa | 35.009 | 333 | 13 | 6 | 7 | 218130000 | 63732000 | 1.5E+08 | 9484100 | 2770900 | 6713100 | -1.27658291 |
| Q80UG5 | SEPT9_MOUSE | Septin9 | 65.575 | 583 | 9 | 4 | 5 | 62951000 | 18383000 | 4.5E+07 | 1464000 | 427520 | 1036500 | -1.277636 |
| P25976 | UBF1_MOUSE | Ubtf | 89.509 | 765 | 5 | 2 | 3 | 19593000 | 5715000 | 1.4E+07 | 416870 | 121600 | 295280 | -1.27997427 |
| Q9QY36 | NAA10_MOUSE | Naa10 | 26.52 | 235 | 6 | 3 | 3 | 59917000 | 17424000 | 4.2E+07 | 3744800 | 1089000 | 2655800 | -1.28614934 |
| P52293 | IMA1_MOUSE | Kpna2 | 57.928 | 529 | 26 | 12 | 14 | 275320000 | 79867000 | 2E+08 | 9832900 | 2852400 | 6980500 | -1.29112816 |
| P16546 | SPTN1_MOUSE | Sptan1 | 284.597 | 2472 | 71 | 28 | 43 | 390000000 | 113080000 | 2.8E+08 | 2052600 | 595130 | 1457500 | -1.29212546 |
| Q99LX0 | PARK7_MOUSE | Park7 | 20.021 | 189 | 7 | 3 | 4 | 46197000 | 13354000 | 3.3E+07 | 3553600 | 1027200 | 2526400 | -1.2983579 |
| P28474 | ADHX_MOUSE | Adh5 | 39.548 | 374 | 2 | 0 | 2 | 5050900 | 0 | 5050900 | 229590 | 0 | 229590 | -1.30108763 |
| Q99K85 | SERC_MOUSE | Psat1 | 40.473 | 370 | 19 | 8 | 11 | 200170000 | 57511000 | 1.4E+08 | 7149000 | 2054000 | 5095000 | -1.31067105 |
| Q9ER88 | RT29_MOUSE | Dap3 | 44.699 | 391 | 16 | 7 | 9 | 109350000 | 31329000 | 7.8E+07 | 4373900 | 1253200 | 3120700 | -1.31630829 |
| Q9CYG7 | TOM34_MOUSE | Tomm34 | 34.278 | 309 | 3 | 1 | 2 | 4692700 | 1335200 | 3357500 | 213310 | 60690 | 152620 | -1.33033154 |
| Q80UM3 | NAA15_MOUSE | Naa15 | 100.961 | 865 | 1 | 0 | 1 | 2230900 | 0 | 2230900 | 35983 | 0 | 35983 | -1.33589757 |
| Q9DC70 | NDUS7_MOUSE | Ndufs7 | 24.683 | 224 | 3 | 1 | 2 | 18470000 | 5229200 | 1.3E+07 | 1319300 | 373520 | 945740 | -1.34024097 |
| P09411 | PGK1_MOUSE | Pgk1 | 44.55 | 417 | 10 | 4 | 6 | 95798000 | 26927000 | 6.9E+07 | 3090200 | 868610 | 2221600 | -1.35484312 |
| O35685 | NUDC_MOUSE | Nudc | 38.358 | 332 | 16 | 7 | 9 | 169160000 | 47333000 | 1.2E+08 | 7354600 | 2058000 | 5296700 | -1.36383274 |
| Q62422 | OSTF1_MOUSE | Ostf1 | 23.783 | 215 | 4 | 2 | 2 | 15569000 | 4354100 | 1.1E+07 | 1197600 | 334930 | 862670 | -1.36498317 |
| P14733 | LMNB1_MOUSE | Lmnb1 | 66.786 | 588 | 8 | 3 | 5 | 40678000 | 11346000 | 2.9E+07 | 1043000 | 290920 | 752120 | -1.37034086 |
| P13541 | MYH3_MOUSE | Myh3 | 223.791 | 1940 | 1 | 0 | 1 | 4211600 | 0 | 4211600 | 33162 | 0 | 33162 | -1.37279501 |
| Q9CSU0 | RPR1B_MOUSE | Rprd1b | 36.884 | 326 | 5 | 2 | 3 | 12874000 | 3572200 | 9301900 | 804630 | 223260 | 581370 | -1.38071257 |
| Q9JMH6 | TRXR1_MOUSE | Txnrd1 | 67.084 | 613 | 13 | 5 | 8 | 98982000 | 27426000 | 7.2E+07 | 2749500 | 761830 | 1987700 | -1.38352852 |
| Q921G6 | LRCH4_MOUSE | Lrch4 | 73.163 | 680 | 4 | 2 | 2 | 26728000 | 7388700 | 1.9E+07 | 763650 | 211100 | 552550 | -1.38812074 |
| P09671 | SODM_MOUSE | Sod2 | 24.603 | 222 | 10 | 5 | 5 | 210850000 | 57932000 | 1.5E+08 | 17570000 | 4827700 | 1.3E+07 | -1.40025038 |
| O08585 | CLCA_MOUSE | Clta | 25.604 | 235 | 11 | 4 | 7 | 309990000 | 84691000 | 2.3E+08 | 30999000 | 8469100 | 2.3E+07 | -1.41156674 |
| Q8K2Q0 | COMD9_MOUSE | Commd9 | 21.85 | 198 | 1 | 0 | 1 | 2880500 | 0 | 2880500 | 221580 | 0 | 221580 | -1.42465103 |
| P70195 | PSB7_MOUSE | Psmb7 | 29.891 | 277 | 9 | 4 | 5 | 98914000 | 26650000 | 7.2E+07 | 8242800 | 2220800 | 6022000 | -1.43914158 |
| P97371 | PSME1_MOUSE | Psme1 | 28.673 | 249 | 17 | 7 | 10 | 253400000 | 68107000 | 1.9E+08 | 14078000 | 3783700 | 1E+07 | -1.44391003 |
| Q5NCE8 | MRS2_MOUSE | Mrs2 | 49.299 | 434 | 2 | 1 | 1 | 6599300 | 1771400 | 4827900 | 235690 | 63264 | 172420 | -1.44650577 |
| P17047 | LAMP2_MOUSE | Lamp2 | 45.681 | 415 | 3 | 1 | 2 | 14964000 | 3927200 | 1.1E+07 | 680200 | 178510 | 501690 | -1.49077511 |
| Q61171 | PRDX2_MOUSE | Prdx2 | 21.779 | 198 | 8 | 3 | 5 | 137610000 | 36081000 | 1E+08 | 10585000 | 2775400 | 7809600 | -1.49245275 |
| Q9JHK4 | PGTA_MOUSE | Rabggta | 64.989 | 567 | 11 | 5 | 6 | 37044000 | 9527400 | 2.8E+07 | 841910 | 216530 | 625380 | -1.53016873 |
| P62482 | KCAB2_MOUSE | Kcnab2 | 41.021 | 367 | 11 | 4 | 7 | 65892000 | 16934000 | 4.9E+07 | 3137700 | 806390 | 2331300 | -1.53162183 |
| Q9D0E1 | HNRPM_MOUSE | Hnrnpm | 77.649 | 729 | 10 | 4 | 6 | 42126000 | 10816000 | 3.1E+07 | 765930 | 196660 | 569270 | -1.53345645 |
| Q61189 | ICLN_MOUSE | Clns1a | 26.021 | 236 | 2 | 1 | 1 | 7005600 | 1794200 | 5211400 | 636870 | 163110 | 473760 | -1.53833028 |
| Q60809 | CNOT7_MOUSE | Cnot7 | 32.718 | 285 | 3 | 1 | 2 | 13734000 | 3507700 | 1E+07 | 858410 | 219230 | 639170 | -1.54378574 |
| Q60780 | GAS7_MOUSE | Gas7 | 48.174 | 421 | 8 | 3 | 5 | 35896000 | 9079700 | 2.7E+07 | 1435800 | 363190 | 1072600 | -1.56237752 |
| Q9CWY8 | RNH2A_MOUSE | Rnaseh2a | 33.513 | 301 | 6 | 3 | 3 | 17680000 | 4402700 | 1.3E+07 | 1105000 | 275170 | 829830 | -1.59246875 |
| Q9D6J6 | NDUV2_MOUSE | Ndufv2 | 27.285 | 248 | 3 | 1 | 2 | 10853000 | 2680400 | 8172500 | 723530 | 178690 | 544840 | -1.60832916 |
| G5E870 | TRIPC_MOUSE | Trip12 | 224.128 | 2025 | 5 | 1 | 4 | 11965000 | 2936500 | 9028800 | 104050 | 25535 | 78511 | -1.62043662 |
| Q9EQI8 | RM46_MOUSE | Mrpl46 | 32.132 | 283 | 5 | 2 | 3 | 29334000 | 7195000 | 2.2E+07 | 1396800 | 342620 | 1054200 | -1.62152347 |
| Q3UMB9 | WASC4_MOUSE | Washc4 | 136.37 | 1173 | 1 | 0 | 1 | 2614000 | 0 | 2614000 | 36305 | 0 | 36305 | -1.62756394 |
| Q61152 | PTN18_MOUSE | Ptpn18 | 50.202 | 453 | 2 | 1 | 1 | 9677200 | 2342500 | 7334700 | 302410 | 73205 | 229210 | -1.64668891 |
| Q8K4M5 | COMD1_MOUSE | Commd1 | 20.996 | 188 | 3 | 1 | 2 | 12451000 | 3012500 | 9438200 | 1778700 | 430360 | 1348300 | -1.6475505 |
| Q64737 | PUR2_MOUSE | Gart | 107.503 | 1010 | 3 | 1 | 2 | 8194500 | 1980800 | 6213700 | 154610 | 37374 | 117240 | -1.64936937 |
| Q9D083 | SPC24_MOUSE | Spc24 | 23.416 | 201 | 1 | 0 | 1 | 2734900 | 0 | 2734900 | 210370 | 0 | 210370 | -1.65899967 |
| Q9CZX9 | EMC4_MOUSE | Emc4 | 20.117 | 183 | 5 | 2 | 3 | 25550000 | 6070500 | 1.9E+07 | 2322700 | 551860 | 1770800 | -1.68203236 |
| Q80UN1 | KCTD9_MOUSE | Kctd9 | 36.994 | 339 | 4 | 1 | 3 | 17508000 | 4157500 | 1.3E+07 | 761210 | 180760 | 580450 | -1.68305157 |
| Q91YM4 | FAKD4_MOUSE | Tbrg4 | 71.513 | 630 | 4 | 1 | 3 | 13130000 | 3117900 | 1E+07 | 345520 | 82051 | 263470 | -1.68308363 |
| Q6KAR6 | EXOC3_MOUSE | Exoc3 | 86.455 | 755 | 1 | 0 | 1 | 2833400 | 0 | 2833400 | 67462 | 0 | 67462 | -1.6970639 |
| Q9D172 | GAL3A_MOUSE | Gatd3 | 28.09 | 266 | 2 | 1 | 1 | 25448000 | 5996400 | 1.9E+07 | 1590500 | 374780 | 1215700 | -1.6976758 |
| O35343 | IMA3_MOUSE | Kpna4 | 57.923 | 521 | 4 | 2 | 2 | 37900000 | 8929000 | 2.9E+07 | 1895000 | 446450 | 1448500 | -1.69803897 |
| Q99LP6 | GRPE1_MOUSE | Grpel1 | 24.307 | 217 | 10 | 4 | 6 | 121540000 | 28534000 | 9.3E+07 | 7149600 | 1678500 | 5471200 | -1.70470383 |
| Q99PU8 | DHX30_MOUSE | Dhx30 | 136.668 | 1217 | 3 | 1 | 2 | 6162600 | 1435000 | 4727700 | 72502 | 16882 | 55620 | -1.72008775 |
| Q9WUD1 | CHIP_MOUSE | Stub1 | 34.909 | 304 | 7 | 3 | 4 | 71033000 | 16489000 | 5.5E+07 | 2959700 | 687020 | 2272700 | -1.7259166 |
| Q9EPK6 | SIL1_MOUSE | Sil1 | 52.43 | 465 | 11 | 5 | 6 | 55089000 | 12583000 | 4.3E+07 | 2295400 | 524280 | 1771100 | -1.75622452 |
| Q01730 | RSU1_MOUSE | Rsu1 | 31.55 | 277 | 5 | 2 | 3 | 29972000 | 6813700 | 2.3E+07 | 1763100 | 400810 | 1362200 | -1.76500033 |
| O35465 | FKBP8_MOUSE | Fkbp8 | 43.529 | 402 | 6 | 2 | 4 | 34033000 | 7604600 | 2.6E+07 | 1620600 | 362120 | 1258500 | -1.79712298 |
| Q8R429 | AT2A1_MOUSE | Atp2a1 | 109.425 | 994 | 1 | 0 | 1 | 5854100 | 0 | 5854100 | 114790 | 0 | 114790 | -1.79944405 |
| P50247 | SAHH_MOUSE | Ahcy | 47.688 | 432 | 25 | 12 | 13 | 221680000 | 48782000 | 1.7E+08 | 8526200 | 1876200 | 6650000 | -1.82551706 |
| P38060 | HMGCL_MOUSE | Hmgcl | 34.239 | 325 | 3 | 1 | 2 | 11905000 | 2574600 | 9330800 | 661410 | 143030 | 518380 | -1.85765247 |
| Q9CR41 | HYPK_MOUSE | Hypk | 13.651 | 121 | 6 | 2 | 4 | 52917000 | 11426000 | 4.1E+07 | 8819500 | 1904300 | 6915300 | -1.86051276 |
| Q3TIV5 | ZC3HF_MOUSE | Zc3h15 | 48.327 | 426 | 1 | 0 | 1 | 9274900 | 0 | 9274900 | 319830 | 0 | 319830 | -1.86941149 |
| P14152 | MDHC_MOUSE | Mdh1 | 36.511 | 334 | 6 | 2 | 4 | 52871000 | 11356000 | 4.2E+07 | 2517700 | 540770 | 1976900 | -1.87017794 |
| P34022 | RANG_MOUSE | Ranbp1 | 23.596 | 203 | 2 | 1 | 1 | 29805000 | 6370300 | 2.3E+07 | 2709500 | 579120 | 2130400 | -1.87917001 |
| Q8BYA0 | TBCD_MOUSE | Tbcd | 133.321 | 1196 | 3 | 1 | 2 | 10800000 | 2303200 | 8497000 | 168750 | 35987 | 132770 | -1.88331387 |
| O35345 | IMA7_MOUSE | Kpna6 | 59.964 | 536 | 10 | 4 | 6 | 28419000 | 5996800 | 2.2E+07 | 1136800 | 239870 | 896910 | -1.90271455 |
| P63280 | UBC9_MOUSE | Ube2i | 18.007 | 158 | 3 | 1 | 2 | 22370000 | 4708400 | 1.8E+07 | 3195700 | 672630 | 2523000 | -1.90725824 |
| O08709 | PRDX6_MOUSE | Prdx6 | 24.871 | 224 | 10 | 3 | 7 | 83304000 | 17298000 | 6.6E+07 | 5206500 | 1081200 | 4125400 | -1.93199193 |
| O88952 | LIN7C_MOUSE | Lin7c | 21.834 | 197 | 11 | 5 | 6 | 163730000 | 33945000 | 1.3E+08 | 10916000 | 2263000 | 8652600 | -1.93490824 |
| A6PWV5 | ARI3C_MOUSE | Arid3c | 43.633 | 409 | 1 | 0 | 1 | 3858400 | 0 | 3858400 | 192920 | 0 | 192920 | -1.9532854 |
| P97377 | CDK2_MOUSE | Cdk2 | 38.978 | 346 | 9 | 2 | 7 | 51720000 | 10614000 | 4.1E+07 | 2248700 | 461480 | 1787200 | -1.95338054 |
| Q91WG8 | GLCNE_MOUSE | Gne | 79.199 | 722 | 3 | 1 | 2 | 9031400 | 1830900 | 7200500 | 231570 | 46945 | 184630 | -1.97554409 |
| Q02819 | NUCB1_MOUSE | Nucb1 | 53.409 | 459 | 3 | 1 | 2 | 9998700 | 2013000 | 7985700 | 333290 | 67100 | 266190 | -1.9880717 |
| Q61166 | MARE1_MOUSE | Mapre1 | 30.016 | 268 | 13 | 5 | 8 | 141750000 | 28356000 | 1.1E+08 | 7460400 | 1492400 | 5968000 | -1.99956747 |
| Q91YI0 | ARLY_MOUSE | Asl | 51.739 | 464 | 1 | 0 | 1 | 4367900 | 0 | 4367900 | 145600 | 0 | 145600 | -2.00828824 |
| P34884 | MIF_MOUSE | Mif | 12.504 | 115 | 19 | 10 | 9 | 3341000000 | 632400000 | 2.7E+09 | 556830000 | 105400000 | 4.5E+08 | -2.09863808 |
| O88811 | STAM2_MOUSE | Stam2 | 57.455 | 523 | 6 | 2 | 4 | 23994000 | 4492700 | 2E+07 | 959770 | 179710 | 780060 | -2.11789347 |
| Q9JIH2 | NUP50_MOUSE | Nup50 | 49.485 | 466 | 1 | 0 | 1 | 5485100 | 0 | 5485100 | 182840 | 0 | 182840 | -2.12626562 |
| Q8BGZ4 | CDC23_MOUSE | Cdc23 | 68.562 | 597 | 3 | 1 | 2 | 7317300 | 1359500 | 5957800 | 215210 | 39985 | 175230 | -2.13170354 |
| Q9QUI0 | RHOA_MOUSE | Rhoa | 21.782 | 193 | 1 | 0 | 1 | 7578900 | 0 | 7578900 | 631580 | 0 | 631580 | -2.1361024 |
| P26151 | FCGR1_MOUSE | Fcgr1 | 44.888 | 404 | 11 | 5 | 6 | 222060000 | 40534000 | 1.8E+08 | 13062000 | 2384300 | 1.1E+07 | -2.16300353 |
| Q8CFQ3 | AQR_MOUSE | Aqr | 170.294 | 1481 | 3 | 1 | 2 | 14298000 | 2581600 | 1.2E+07 | 180990 | 32679 | 148310 | -2.18226584 |
| P08101 | FCGR2_MOUSE | Fcgr2 | 36.695 | 330 | 3 | 1 | 2 | 11386000 | 2045900 | 9340500 | 669790 | 120350 | 549440 | -2.19076415 |
| Q91VN4 | MIC25_MOUSE | Chchd6 | 29.852 | 273 | 4 | 1 | 3 | 13460000 | 2379800 | 1.1E+07 | 841220 | 148730 | 692490 | -2.21904564 |
| Q6ZPU9 | KBP_MOUSE | Kifbp | 71.052 | 617 | 2 | 0 | 2 | 4387700 | 0 | 4387700 | 118590 | 0 | 118590 | -2.22905347 |
| Q3U6N9 | CH033_MOUSE |  | 24.465 | 222 | 5 | 2 | 3 | 19487000 | 3415100 | 1.6E+07 | 1623900 | 284590 | 1339300 | -2.23454974 |
| P11438 | LAMP1_MOUSE | Lamp1 | 43.865 | 406 | 2 | 1 | 1 | 7463600 | 1300300 | 6163300 | 414640 | 72237 | 342400 | -2.2448585 |
| Q93092 | TALDO_MOUSE | Taldo1 | 37.387 | 337 | 4 | 1 | 3 | 30635000 | 5324800 | 2.5E+07 | 1276500 | 221870 | 1054600 | -2.24890826 |
| Q05915 | GCH1_MOUSE | Gch1 | 27.014 | 241 | 1 | 0 | 1 | 3574300 | 0 | 3574300 | 238290 | 0 | 238290 | -2.25041627 |
| Q9WUK4 | RFC2_MOUSE | Rfc2 | 38.725 | 349 | 13 | 5 | 8 | 160350000 | 27716000 | 1.3E+08 | 6414100 | 1108600 | 5305500 | -2.25872495 |
| Q9EQU5 | SET_MOUSE | Set | 33.378 | 289 | 8 | 3 | 5 | 58412000 | 10083000 | 4.8E+07 | 4867700 | 840270 | 4027400 | -2.2609642 |
| Q8R0J7 | VP37B_MOUSE | Vps37b | 31.056 | 285 | 3 | 1 | 2 | 8887900 | 1528400 | 7359500 | 634850 | 109170 | 525680 | -2.26758559 |
| Q63829 | COMD3_MOUSE | Commd3 | 22.037 | 195 | 1 | 0 | 1 | 5787600 | 0 | 5787600 | 482300 | 0 | 482300 | -2.27819391 |
| Q9CRD0 | OCAD1_MOUSE | Ociad1 | 27.61 | 247 | 3 | 1 | 2 | 16701000 | 2840000 | 1.4E+07 | 1192900 | 202860 | 990060 | -2.28706851 |
| Q9CYI0 | NJMU_MOUSE |  | 44.422 | 393 | 1 | 0 | 1 | 4104900 | 0 | 4104900 | 171040 | 0 | 171040 | -2.30398821 |
| P05063 | ALDOC_MOUSE | Aldoc | 39.395 | 363 | 1 | 0 | 1 | 5328800 | 0 | 5328800 | 222030 | 0 | 222030 | -2.32649793 |
| Q9DCC4 | P5CR3_MOUSE | Pycr3 | 28.721 | 274 | 3 | 1 | 2 | 20817000 | 3279000 | 1.8E+07 | 1601300 | 252230 | 1349100 | -2.41915643 |
| Q9CWN7 | CNO11_MOUSE | Cnot11 | 54.959 | 505 | 2 | 1 | 1 | 5035100 | 792830 | 4242300 | 179830 | 28315 | 151510 | -2.41976319 |
| Q9WTK3 | GPAA1_MOUSE | Gpaa1 | 67.949 | 621 | 2 | 0 | 2 | 8849800 | 0 | 8849800 | 384780 | 0 | 384780 | -2.42489319 |
| P51150 | RAB7A_MOUSE | Rab7a | 23.49 | 207 | 1 | 0 | 1 | 4522200 | 0 | 4522200 | 266010 | 0 | 266010 | -2.48063755 |
| Q9D8U8 | SNX5_MOUSE | Snx5 | 46.797 | 404 | 6 | 2 | 4 | 37788000 | 5715300 | 3.2E+07 | 1453400 | 219820 | 1233600 | -2.48845817 |
| Q9D2N9 | VP33A_MOUSE | Vps33a | 67.555 | 598 | 2 | 0 | 2 | 6330800 | 0 | 6330800 | 154410 | 0 | 154410 | -2.53985582 |
| Q3TAS6 | EMC10_MOUSE | Emc10 | 27.053 | 258 | 1 | 0 | 1 | 6943900 | 0 | 6943900 | 578660 | 0 | 578660 | -2.54958098 |
| Q8CIM5 | GPR84_MOUSE | Gpr84 | 43.717 | 396 | 1 | 0 | 1 | 4589700 | 0 | 4589700 | 269980 | 0 | 269980 | -2.5551233 |
| O88990 | ACTN3_MOUSE | Actn3 | 103.043 | 900 | 4 | 0 | 4 | 13391000 | 0 | 1.3E+07 | 230870 | 0 | 230870 | -2.62908194 |
| Q99P31 | HPBP1_MOUSE | Hspbp1 | 39.167 | 357 | 20 | 8 | 12 | 427220000 | 59252000 | 3.7E+08 | 21361000 | 2962600 | 1.8E+07 | -2.63465239 |
| P54728 | RD23B_MOUSE | Rad23b | 43.513 | 416 | 1 | 0 | 1 | 3388900 | 0 | 3388900 | 225930 | 0 | 225930 | -2.70177119 |
| Q8K1X4 | NCKPL_MOUSE | Nckap1l | 128.905 | 1134 | 12 | 4 | 8 | 50955000 | 6700500 | 4.4E+07 | 796170 | 104690 | 691480 | -2.7234998 |
| Q9CS42 | PRPS2_MOUSE | Prps2 | 34.786 | 318 | 1 | 0 | 1 | 7477700 | 0 | 7477700 | 393560 | 0 | 393560 | -2.82650001 |
| O88653 | LTOR3_MOUSE | Lamtor3 | 13.553 | 124 | 1 | 0 | 1 | 6181700 | 0 | 6181700 | 772710 | 0 | 772710 | -2.84098812 |
| P23198 | CBX3_MOUSE | Cbx3 | 20.855 | 183 | 1 | 0 | 1 | 8034200 | 0 | 8034200 | 892690 | 0 | 892690 | -2.87412111 |
| Q62384 | ZPR1_MOUSE | Zpr1 | 50.715 | 459 | 2 | 0 | 2 | 6645700 | 0 | 6645700 | 237340 | 0 | 237340 | -2.87460936 |
| Q9CQ18 | RNH2C_MOUSE | Rnaseh2c | 17.823 | 166 | 4 | 1 | 3 | 19638000 | 2303000 | 1.7E+07 | 1636500 | 191910 | 1444600 | -2.91210152 |
| O09111 | NDUBB_MOUSE | Ndufb11 | 17.444 | 151 | 1 | 0 | 1 | 5521000 | 0 | 5521000 | 552100 | 0 | 552100 | -2.94517732 |
| Q99L13 | 3HIDH_MOUSE | Hibadh | 35.44 | 335 | 1 | 0 | 1 | 4792700 | 0 | 4792700 | 319510 | 0 | 319510 | -2.96634608 |
| Q3USB7 | PLCL1_MOUSE | Plcl1 | 122.673 | 1096 | 1 | 0 | 1 | 7279500 | 0 | 7279500 | 119340 | 0 | 119340 | -2.97088082 |
| P51943 | CCNA2_MOUSE | Ccna2 | 47.269 | 422 | 4 | 2 | 2 | 18046000 | 1921700 | 1.6E+07 | 820270 | 87351 | 732920 | -3.06875465 |
| P24860 | CCNB1_MOUSE | Ccnb1 | 48.052 | 430 | 1 | 0 | 1 | 8413500 | 0 | 8413500 | 400640 | 0 | 400640 | -3.08788473 |
| Q9ESK3 | CAN10_MOUSE | Capn10 | 74.596 | 666 | 2 | 1 | 1 | 18351000 | 1888700 | 1.6E+07 | 436920 | 44969 | 391960 | -3.12367415 |
| Q8CDG5 | CRERF_MOUSE | Crebrf | 72.598 | 640 | 2 | 1 | 1 | 17180000 | 1708100 | 1.5E+07 | 490870 | 48803 | 442070 | -3.17919536 |
| P17897 | LYZ1_MOUSE | Lyz1 | 16.794 | 148 | 1 | 0 | 1 | 19766000 | 0 | 2E+07 | 1796900 | 0 | 1796900 | -3.17959819 |
| Q9D1A2 | CNDP2_MOUSE | Cndp2 | 52.767 | 475 | 4 | 1 | 3 | 13392000 | 1330800 | 1.2E+07 | 496020 | 49289 | 446730 | -3.18010346 |
| Q9JIB0 | MOG1_MOUSE | Rangrf | 20.413 | 185 | 2 | 0 | 2 | 10240000 | 0 | 1E+07 | 1462800 | 0 | 1462800 | -3.2020488 |
| Q9JI10 | STK3_MOUSE | Stk3 | 56.855 | 497 | 2 | 0 | 2 | 10285000 | 0 | 1E+07 | 331760 | 0 | 331760 | -3.29285181 |
| P07310 | KCRM_MOUSE | Ckm | 43.045 | 381 | 2 | 0 | 2 | 14093000 | 0 | 1.4E+07 | 563720 | 0 | 563720 | -3.34489879 |
| P26350 | PTMA_MOUSE | Ptma | 12.254 | 111 | 1 | 0 | 1 | 11110000 | 0 | 1.1E+07 | 3703400 | 0 | 3703400 | -3.35791033 |
| P70677 | CASP3_MOUSE | Casp3 | 31.475 | 277 | 2 | 0 | 2 | 13438000 | 0 | 1.3E+07 | 707250 | 0 | 707250 | -3.38624394 |
| P19157 | GSTP1_MOUSE | Gstp1 | 23.609 | 210 | 6 | 2 | 4 | 58991000 | 5115200 | 5.4E+07 | 4915900 | 426270 | 4489700 | -3.39678019 |
| Q61545 | EWS_MOUSE | Ewsr1 | 68.462 | 655 | 2 | 0 | 2 | 12680000 | 0 | 1.3E+07 | 551290 | 0 | 551290 | -3.55474501 |
| Q02257 | PLAK_MOUSE | Jup | 81.801 | 745 | 7 | 2 | 5 | 21564000 | 1554800 | 2E+07 | 458820 | 33082 | 425730 | -3.68592025 |
| Q9CPT5 | NOP16_MOUSE | Nop16 | 21.139 | 178 | 2 | 0 | 2 | 18768000 | 0 | 1.9E+07 | 1706200 | 0 | 1706200 | -3.71436168 |
| E9Q557 | DESP_MOUSE | Dsp | 332.912 | 2883 | 7 | 2 | 5 | 22337000 | 1520900 | 2.1E+07 | 108430 | 7383.1 | 101050 | -3.77469566 |
| Q9WUB3 | PYGM_MOUSE | Pygm | 97.286 | 842 | 4 | 0 | 4 | 12887000 | 0 | 1.3E+07 | 226090 | 0 | 226090 | -3.81671356 |
| Q9QY76 | VAPB_MOUSE | Vapb | 26.946 | 243 | 3 | 1 | 2 | 14331000 | 0 | 1.4E+07 | 842980 | 0 | 842980 | -3.89830685 |
| Q99JR5 | TINAL_MOUSE | Tinagl1 | 52.665 | 466 | 2 | 1 | 1 | 32591000 | 1972600 | 3.1E+07 | 1417000 | 85767 | 1331200 | -3.9562097 |
| P60766 | CDC42_MOUSE | Cdc42 | 21.259 | 191 | 2 | 0 | 2 | 19921000 | 0 | 2E+07 | 2213400 | 0 | 2213400 | -4.1851919 |
| Q91Z83 | MYH7_MOUSE | Myh7 | 222.879 | 1935 | 1 | 0 | 1 | 9452000 | 0 | 9452000 | 75616 | 0 | 75616 | -4.28585922 |
| O88545 | CSN6_MOUSE | Cops6 | 35.88 | 324 | 4 | 1 | 3 | 24557000 | 1157600 | 2.3E+07 | 1754000 | 82682 | 1671400 | -4.33723814 |
| P08228 | SODC_MOUSE | Sod1 | 15.943 | 154 | 1 | 0 | 1 | 18664000 | 0 | 1.9E+07 | 1696700 | 0 | 1696700 | -4.37995718 |
| P97457 | MYL11_MOUSE | Myl11 | 18.955 | 169 | 3 | 0 | 3 | 28659000 | 0 | 2.9E+07 | 2388300 | 0 | 2388300 | -5.03799326 |
| P13542 | MYH8_MOUSE | Myh8 | 222.708 | 1937 | 3 | 0 | 3 | 61297000 | 0 | 6.1E+07 | 486490 | 0 | 486490 | -5.33166684 |
| Q5SX40 | MYH1_MOUSE | Myh1 | 223.342 | 1942 | 4 | 0 | 4 | 81540000 | 0 | 8.2E+07 | 642050 | 0 | 642050 | -6.67788126 |
| Q80VP2 | SPAT7_MOUSE | Spata7 | 65.655 | 582 | 1 | 0 | 1 | 139770000 | 0 | 1.4E+08 | 3583900 | 0 | 3583900 | -7.3925628 |
| Q5SX39 | MYH4_MOUSE | Myh4 | 222.859 | 1939 | 33 | 2 | 31 | 361970000 | 1656200 | 3.6E+08 | 2850200 | 13041 | 2837100 | -7.76521798 |
