# Supplementary material for: TRIP13 promotes the expansion and immunosuppression of CD4+Foxp3+ regulatory T cells by sustaining HAT1 stability
Source: Cell Death Dis. 2026 Jan 14;17(1):32. doi: 10.1038/s41419-025-08214-7 (PMC12804696; doi:10.1038/s41419-025-08214-7)
Supplement: Supplementary file 3 — Raw data of Western Blot [file 41419_2025_8214_MOESM3_ESM.pdf]

Raw data of WB in Figure 1B and 1D

A

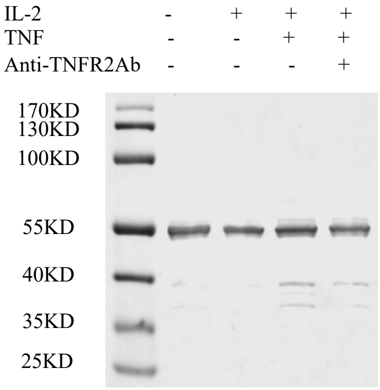

TRIP13

B

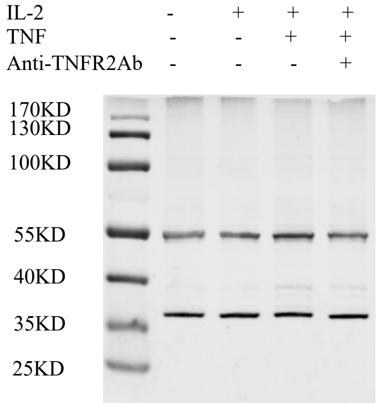

GAPDH

C

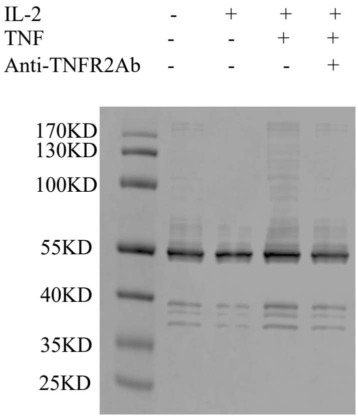

TRIP13

D

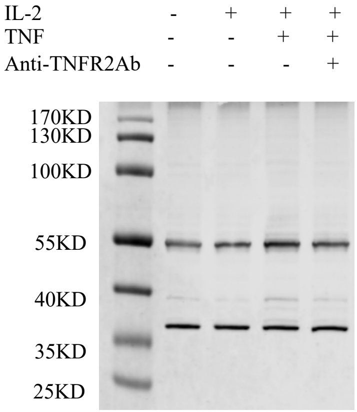

GAPDH

Raw data of WB in Figure 3

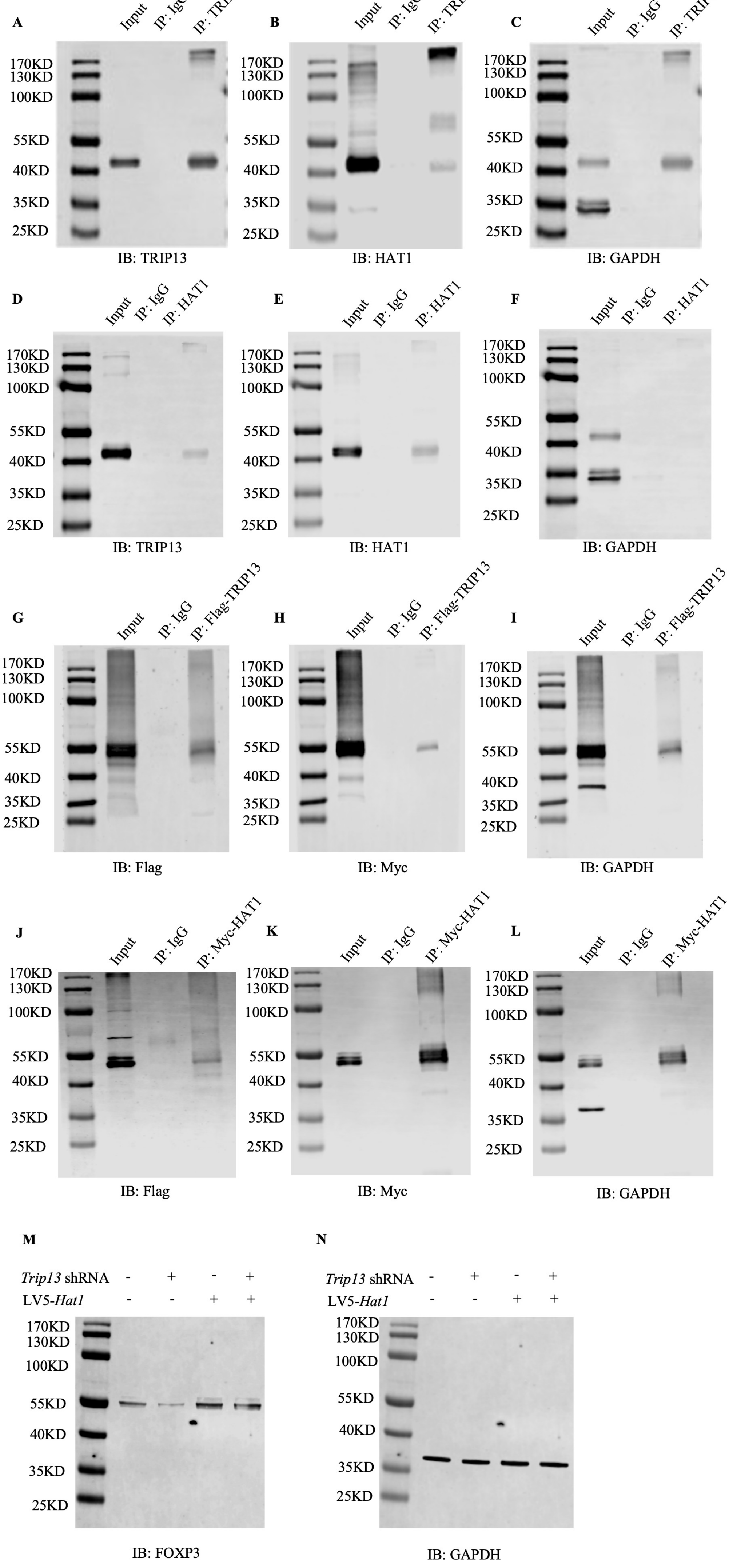

Raw data of WB in Figure 4

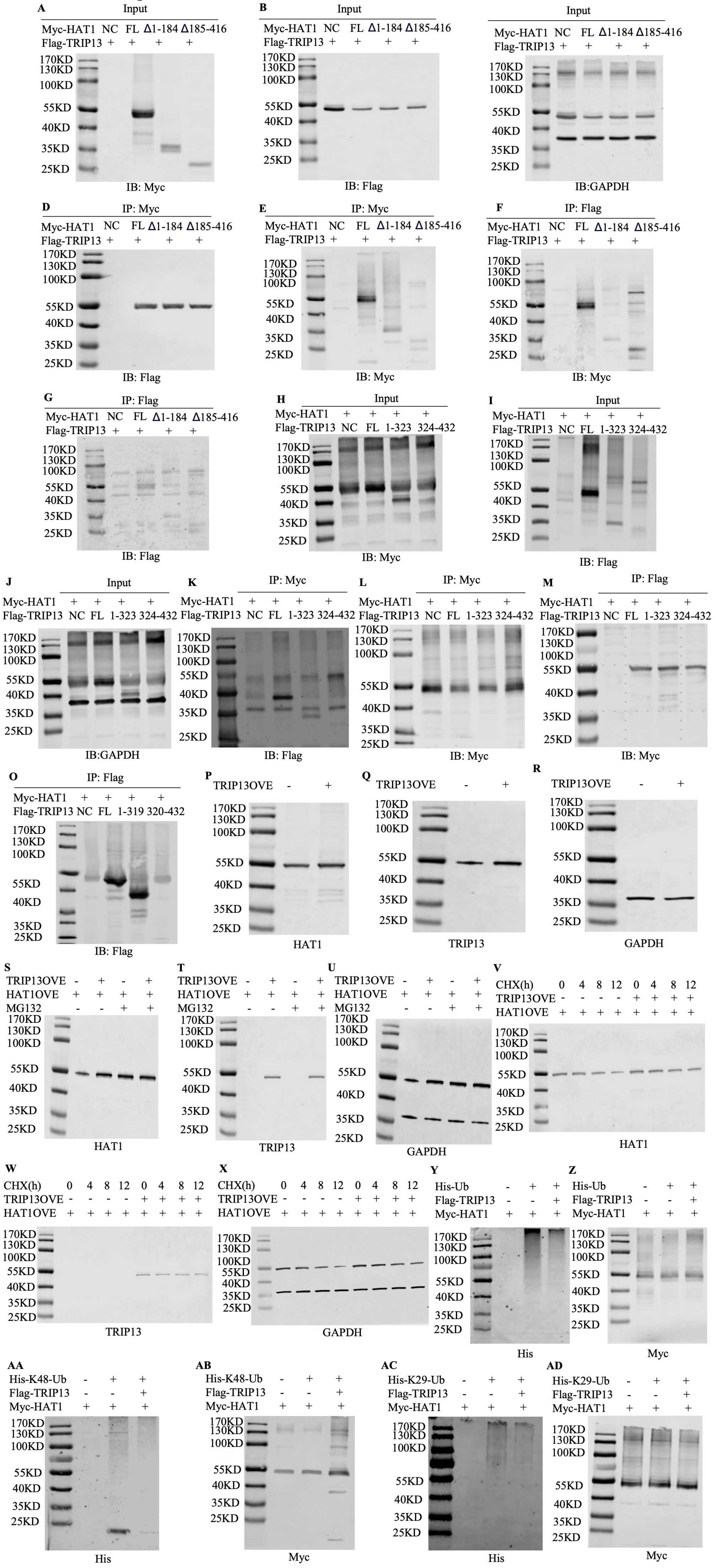

Raw data of WB in Figure 5

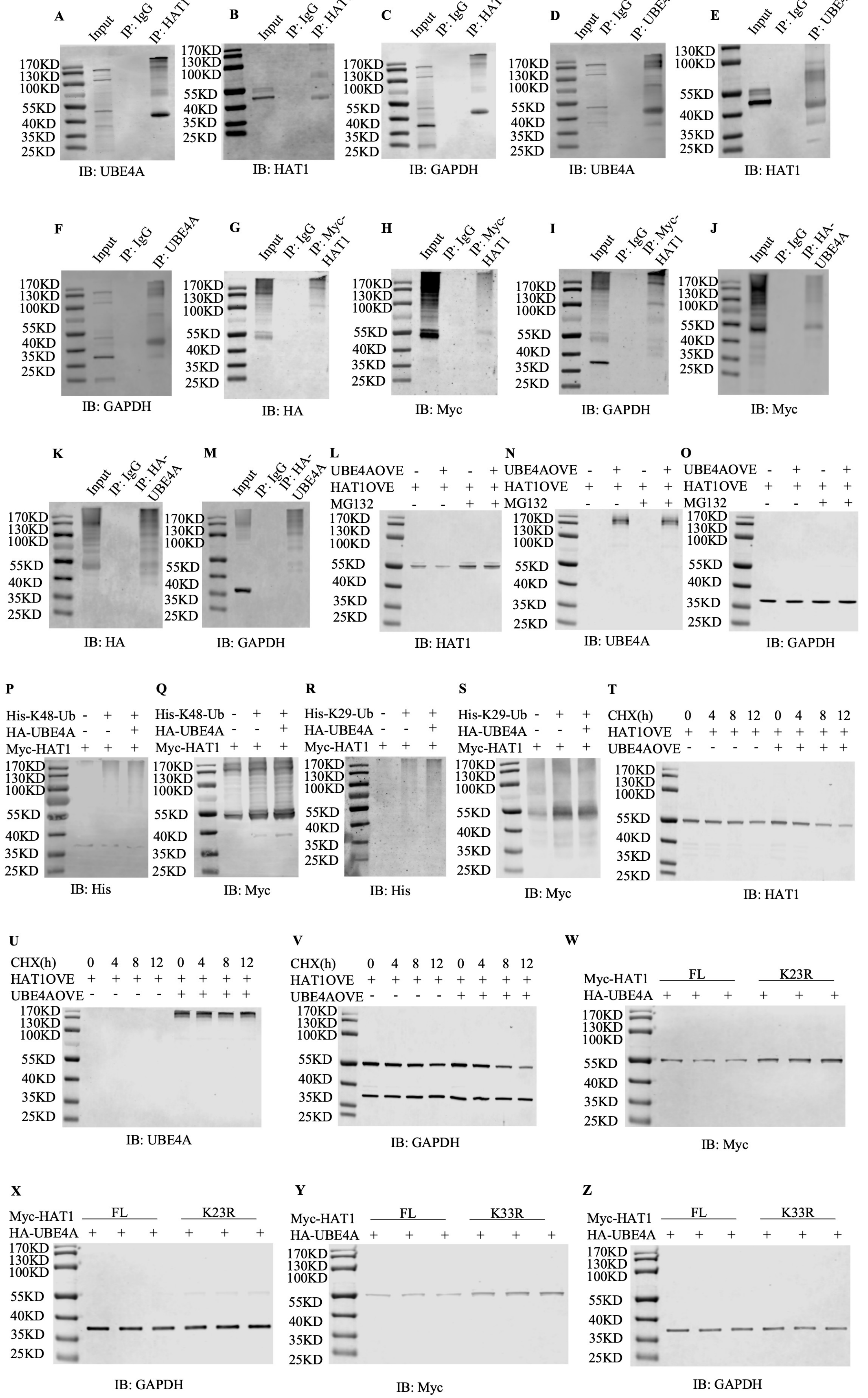

Raw data of WB in Figure 6

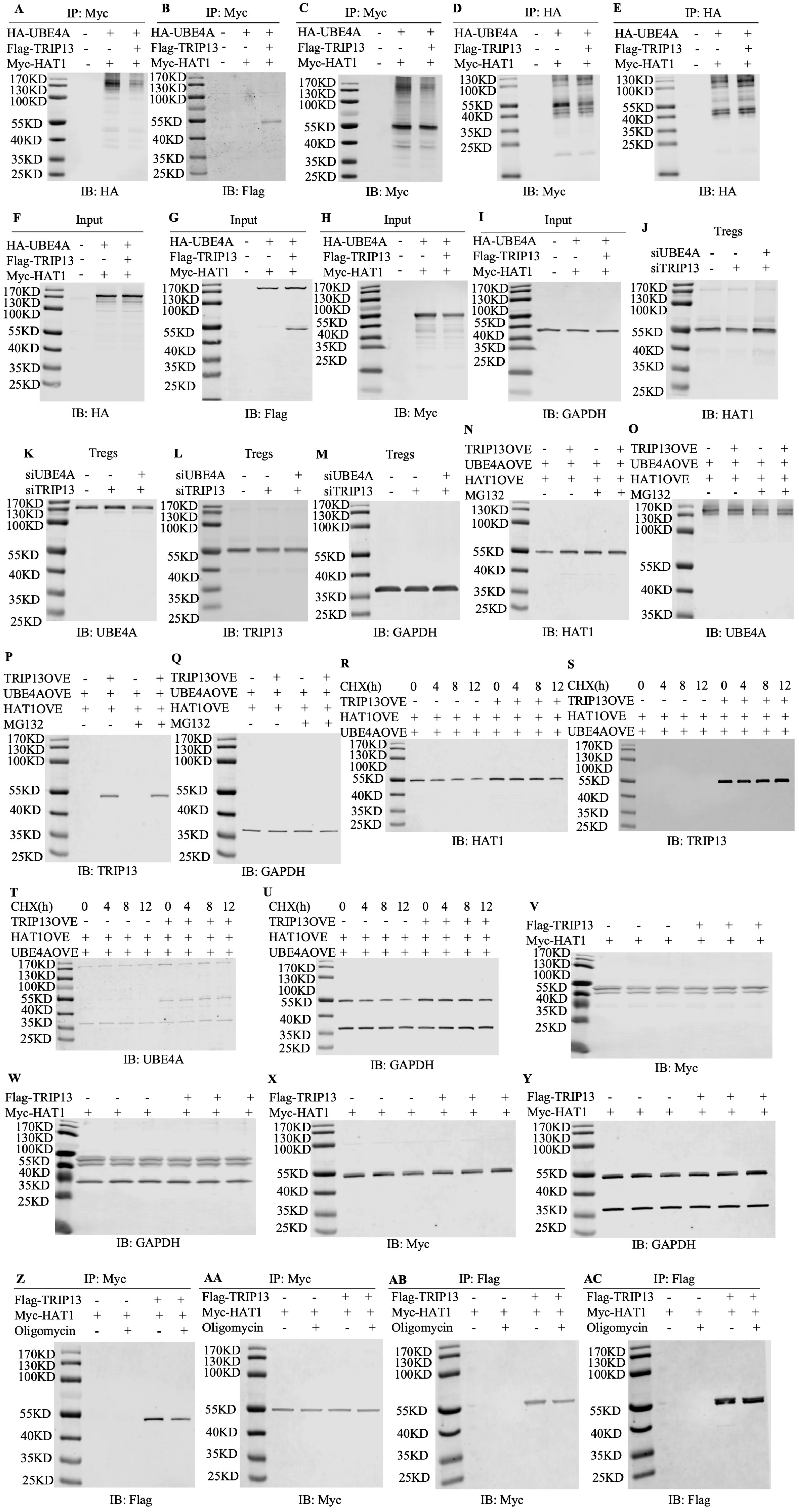

Raw data of WB in Figure S7

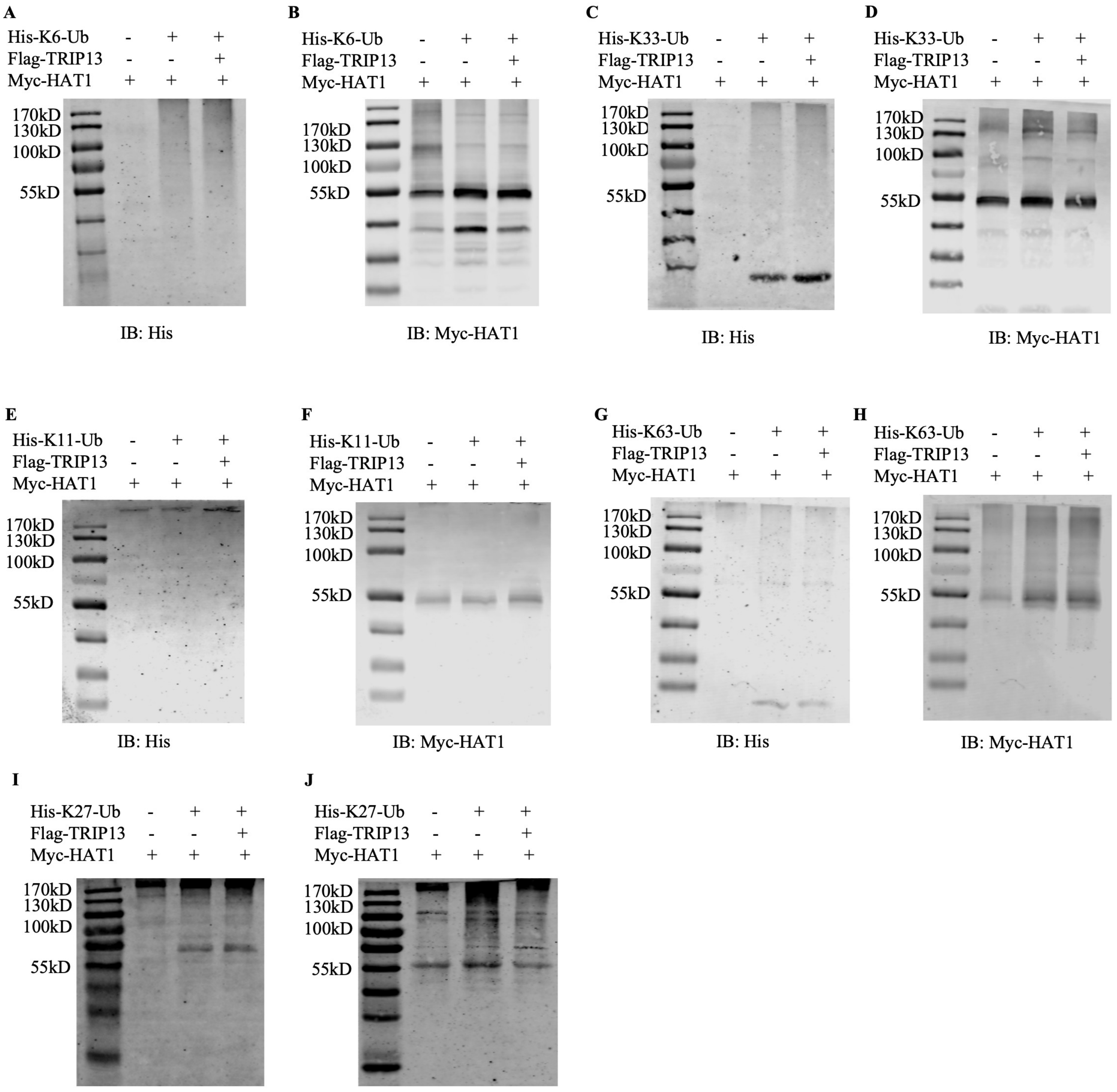

Raw data of WB in Figure S8

A

Myc-HAT1 FL FL FL K6R K6R K6R
HA-UBE4A + + + + + +

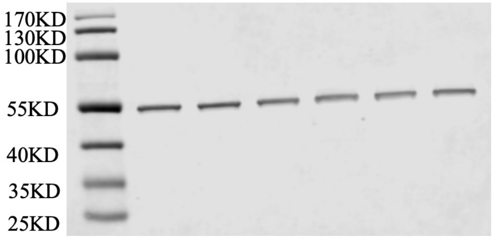

IB: Myc

Myc-HAT1 FL FL FL K6R K6R K6R
HA-UBE4A + + + + + +

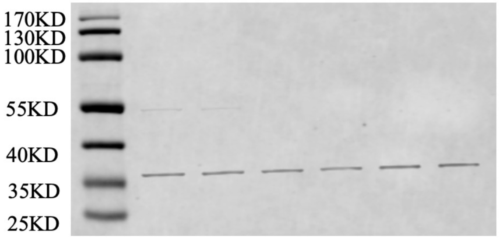

IB: GAPDH

C

Myc-HAT1 FL FL FL K12R K12R K12R
HA-UBE4A + + + + + +

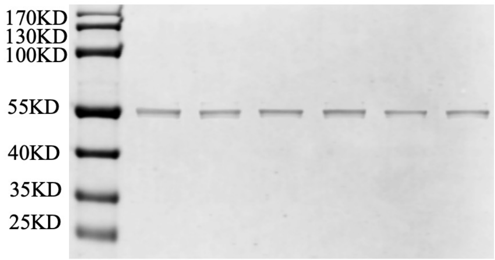

IB: Myc

Myc-HAT1 FL FL FL K12R K12R K12R
HA-UBE4A + + + + + +

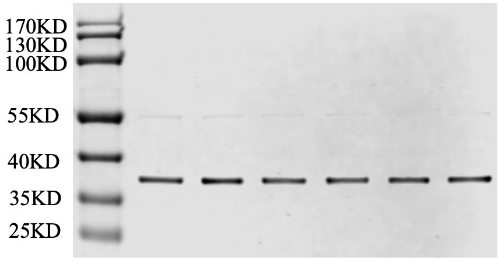

IB: GAPDH

E

Myc-HAT1 FL FL FL K67R K67R K67R
HA-UBE4A + + + + + +

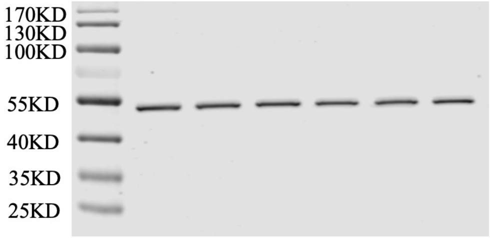

IB: Myc

Myc-HAT1 FL FL FL K67R K67R K67R
HA-UBE4A + + + + + +

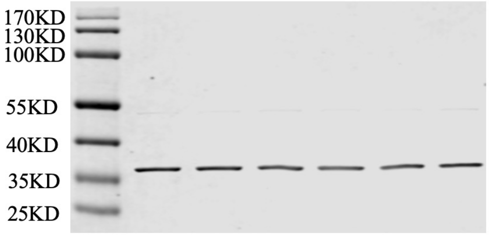

IB: GAPDH

G

Myc-HAT1 FL FL FL K107R K107R K107R
HA-UBE4A + + + + + +

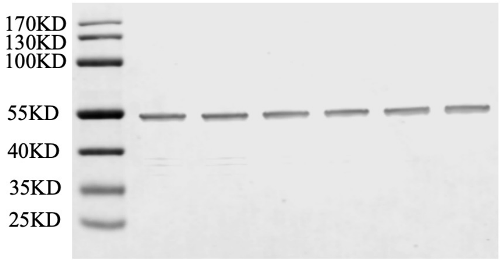

IB: Myc

Myc-HAT1 FL FL FL K107R K107R K107R
HA-UBE4A + + + + + +

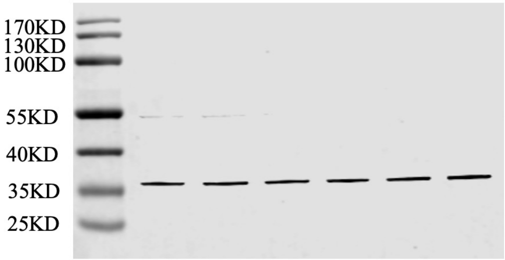

IB: GAPDH

Raw data of WB in Figure S9

A

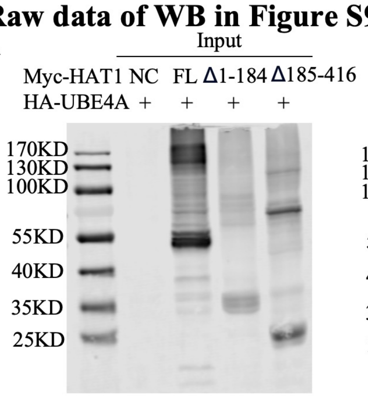

IB: Myc

B

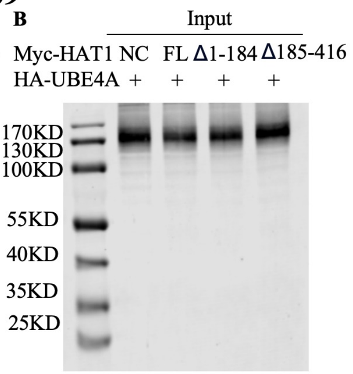

IB: HA

C

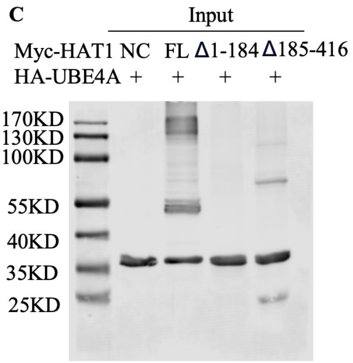

IB:GAPDH

D

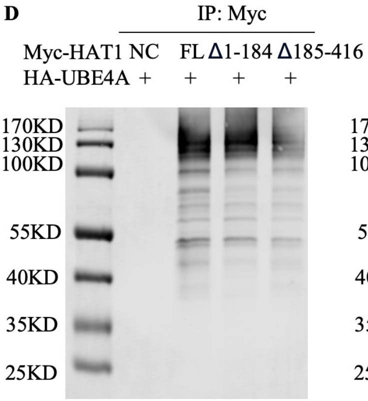

IB: HA

E

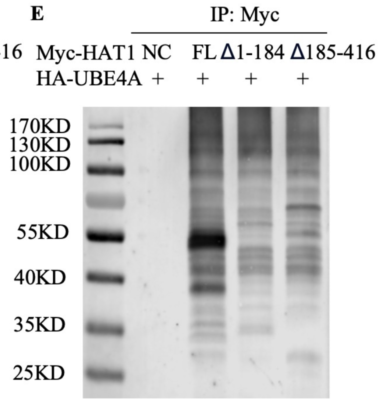

IB: Myc

F

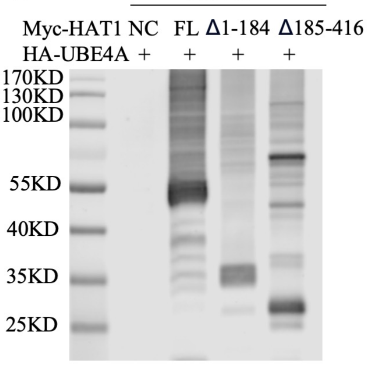

IB: Myc

G

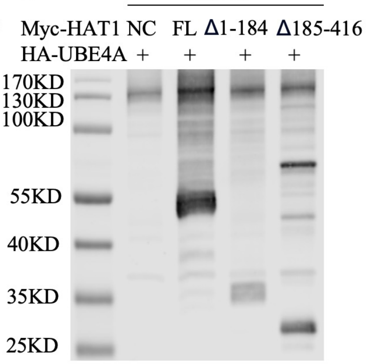

IB: HA
